# Light-Driven Iron-Mediated Thiotrifluoromethylation of Alkenes Using CF_3_CO_2_H

**DOI:** 10.1021/acs.orglett.6c02069

**Published:** 2026-06-09

**Authors:** Ying-Hui Zhou, Ting Zhou, Chi Wai Cheung, Jun-An Ma

**Affiliations:** † Department of Chemistry, State Key Laboratory of Synthetic Biology, 12605 Tianjin University, Tianjin 300072, People’s Republic of China; ‡ State Key Laboratory of Synthetic Chemistry and Department of Chemistry, 26451 The Chinese University of Hong Kong, Shatin, New Territories Hong Kong 999077, People’s Republic of China

## Abstract

Fluorinated thioethers are important motifs in medicinal and agrochemical chemistry, but modular synthesis of structurally complex derivatives remains challenging. Herein we report a photoinduced iron­(III)-mediated thiotrifluoromethylation of alkenes using trifluoroacetic acid as an inexpensive CF_3_ radical source and *S*-aryl benzenesulfonothioates as arylthio donors. Under purple-light irradiation, simple iron­(III) salts enable a three-component reaction that affords diverse aryl 3,3,3-trifluoropropyl sulfides with broad scope and good functional group tolerance. The products are readily elaborated and applied to late-stage modification of complex molecules, providing a simple and scalable route to fluorinated sulfur-containing scaffolds.

## Graphical abstract

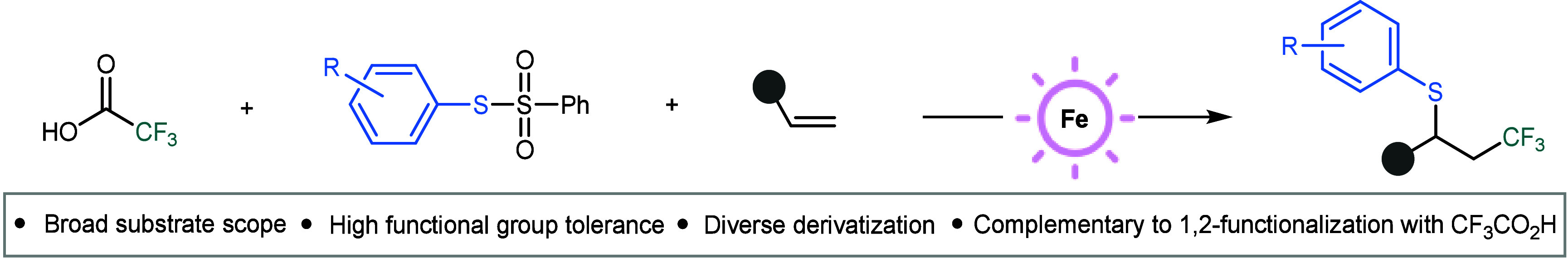

Fluorinated thioethers, particularly aryl 3,3,3-trifluoropropyl sulfides, have emerged as important motifs in pharmaceuticals and agrochemicals due to their unique physicochemical properties, including enhanced lipophilicity, metabolic stability, and membrane permeability^1^ (Figure 1A). These compounds function as critical pharmacophores in serotonin receptor agonists, estrogen receptor ligands, nuclear receptor modulators, insecticides, and antibacterial agents for plant protection.^2^ The development of structurally diverse aryl trifluoropropyl sulfides is thus crucial for advancing drug discovery and agrochemical innovation. In this context, modular and efficient strategies to access structurally complex aryl trifluoropropyl sulfides from simple and readily available building blocks are highly desirable. One particularly attractive route involves three-component alkene difunctionalization using a trifluoromethyl (CF_3_) source, an alkene **4**, and an arylthio precursor^3–6^ (Figure 1B). This strategy enables rapid diversification and streamlines the synthesis of trifluoropropyl thioethers **5**. However, prior methods have largely relied on expensive and prefunctionalized trifluoromethylating reagents, such as sodium trifluoromethanesulfinate (**1**),^3,4^ (trifluoromethyl)­sulfonylbenzene (**2**),^5^ or *S*-aryl trifluoromethanesulfonothioate (**3**),^6^ often requiring noble-metal photocatalysts or copper complexes in the presence of electrophilic or nucleophilic thiolating agents. While effective, these systems are limited by high cost, multistep reagent synthesis, and less favorable scalability.

**Figure 1:**
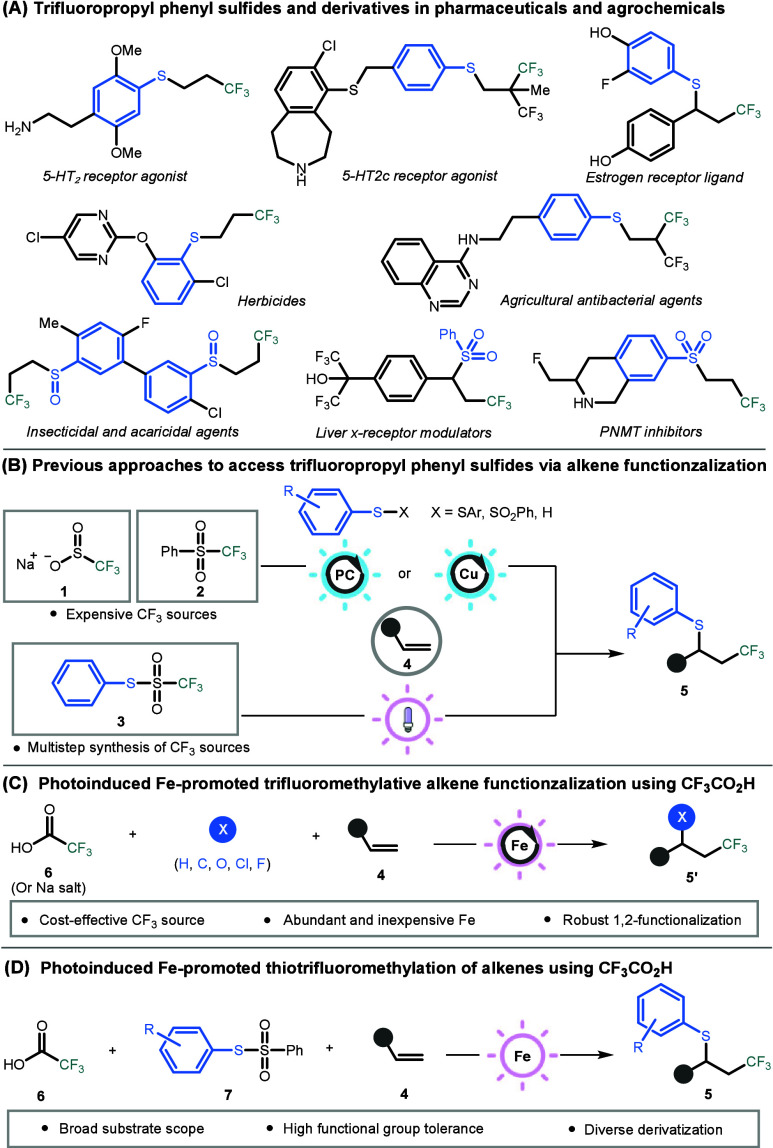
Development of a photoinduced iron-mediated thiotrifluoromethylation of alkenes.

Recently, photoinduced ligand-to-metal charge transfer (LMCT) from carboxylate ions to high-valent Fe­(III) species has emerged as a powerful strategy for generating alkyl radicals under visible light irradiation.^7^ This reactivity mode enables facile catalytic decarboxylative alkylation of aliphatic carboxylic acids without the need for prefunctionalized redox-active groups.^8^ Notably, this approach has also been extended to the direct decarboxylation of trifluoroacetic acid (CF_3_CO_2_H, **6**) or its salts, providing an efficient and bench-stable method for accessing CF_3_ radicals as a cost-effective alternative to conventional CF_3_ sources.^9–14^ The resulting radicals readily engage in hydro-,^9^ carbo-,^10^ and heteroatom-trifluoromethylation^11–13^ of alkenes via 1,2-difunctionalization, furnishing structurally diverse trifluoropropyl thioether products **5′** under mild, sustainable conditions (Figure 1C).

Inspired by the versatility of the Fe-LMCT decarboxylative strategy, we envisioned extending this approach to sulfur-based substrates for the direct synthesis of trifluoropropyl thioethers. Herein, we report a light-driven Fe­(III)-promoted thiotrifluoromethylation of alkenes using CF_3_CO_2_H as a radical precursor and *S*-phenyl benzenesulfonothioate **7** as an arylthio radical source (Figure 1D). This three-component protocol proceeds under visible-light irradiation with simple iron salts, affording a broad array of 3,3,3-trifluoropropyl aryl sulfides **5** in good efficiency and functional group compatibility. The resulting fluorinated thioethers offer valuable handles for further derivatization and expansion into medicinally relevant space. This modular approach thus provides a streamlined route to fluorinated sulfur-containing scaffolds, enabling accelerated development of new fluorinated drugs and agrochemicals.

We commenced our investigation by evaluating various iron salts, bases, oxidants, and additives under purple LED irradiation to promote the three-component coupling of 4-phenylbut-1-ene (**4a**), trifluoroacetic acid (**6**), and *S*-phenyl benzenesulfonothioate (**7a**) in acetonitrile (Table S1, Supporting Information). When 29 mol % of basic ferric acetate ([Fe_3_O­(OAc)_6_(H_2_O)_3_]­OAc, ∼0.87 equiv. Fe) was employed with Cs_2_CO_3_ (0.5 equiv) as the base, the reaction proceeded smoothly, affording the desired phenyl 3,3,3-trifluoropropyl sulfide **8** in 70% yield (Entry 1). Iron­(III) salts such as FeCl_3_·6H_2_O and Fe­(OTf)_3_ delivered only modest yields (Entries 2 and 3). Screening of inorganic and organic bases revealed 1,4-diazabicyclo[2.2.2]­octane (DABCO) as the optimal choice, further promoting the reaction to give compound **8** in 74% yield (Entries 4–6). The addition of di-*tert*-butyl peroxide (DTBP) as an oxidant proved beneficial to the thiotrifluoromethylation process, increasing the yield to 78% (Entries 7–10). Notably, the inclusion of a tridentate nitrogen ligand, bis­(2-pyridylmethyl)­amine (DPA, **L1**), further enhanced the yield to 88% (Entries 11–14). However, reducing the loading of basic ferric acetate to 14 mol % or 5 mol % led to a significant drop in product yield (Entries 15 and 16), which represents a current limitation of this thiotrifluoromethylation protocol. We hypothesized that thio-based side-products derived from *S*-phenyl benzenesulfonothioate may irreversibly coordinate to the Fe species, forming off-cycle complexes and thereby hindering catalytic turnover. Lowering the CF_3_CO_2_H loading to 4.0 or 6.0 equiv., or further increasing it to 10 equiv., decreased the yield to 53–60% (Entries 17–19). These results suggested that 8.0 equiv. provides an optimal balance for generating sufficient LMCT-active Fe­(III)–trifluoroacetate species, while further acid addition may perturb CF_3_CO_2_^–^ availability, **L1** coordination, and iron speciation. Control experiments confirmed that both basic ferric acetate and purple light irradiation are essential for promoting this decarboxylative transformation (Entries 20 and 21). Collectively, these results established basic ferric acetate, **L1**, DABCO, and DTBP as the optimal combination for the thiotrifluoromethylation of alkenes (Entry 11).

With the optimized conditions in hand, we next explored the substrate scope with respect to alkenes (**4a**–**4ab**, Scheme 1). The photoinduced, iron­(III)-mediated thiotrifluoromethylation protocol demonstrated broad generality, accommodating a wide range of alkene substrates to furnish structurally diverse 3,3,3-trifluoropropyl aryl sulfides **8**–**35**. Aryl-substituted alkene (**8**), aryl alkenyl ether (**9**), alkenyl benzoate (**10**), aryl alkenoate (**11**), amide-derived alkene (**12**), and imide-coordinated alkene (**13**) all underwent smooth transformation, affording the corresponding trifluoropropyl aryl sulfides. Alkenes with varied chain lengths also reacted successfully, providing the corresponding homologated trifluoropropyl thioethers **14**–**22**. A diverse array of electron-donating and electron-withdrawing groups was well tolerated on the aryl alkenyl ether substrates, including *tert*-butyl (**23**), methyl (**24**), methylthio (**25**), difluoromethoxy (**26**), trifluoromethoxy (**27**), acetyl (**28**), benzoyl (**29**), trifluoromethyl (**30**), nitrile (**31**), and aldehyde (**32**) groups. These functional fluorinated thioethers are crucial for modulating the physiological properties of bioactive molecules and serve as useful handles for further synthetic transformations. Furthermore, heterocycles such as furan (**33**) and thiophene (**34**), along with biphenyl motifs (**35**), were successfully incorporated into the fluorinated thioether scaffolds. Notably, longer-chain products such as **14** and **17** were obtained in lower yields than their shorter-chain analogues **8** and **16**, likely due in part to the lower solubility of the corresponding longer-chain alkenes in polar MeCN, which may reduce the effective reaction efficiency. Collectively, this modular and scalable protocol offers an efficient strategy for accessing structurally diverse aryl trifluoropropyl sulfides.

**Scheme 1:**
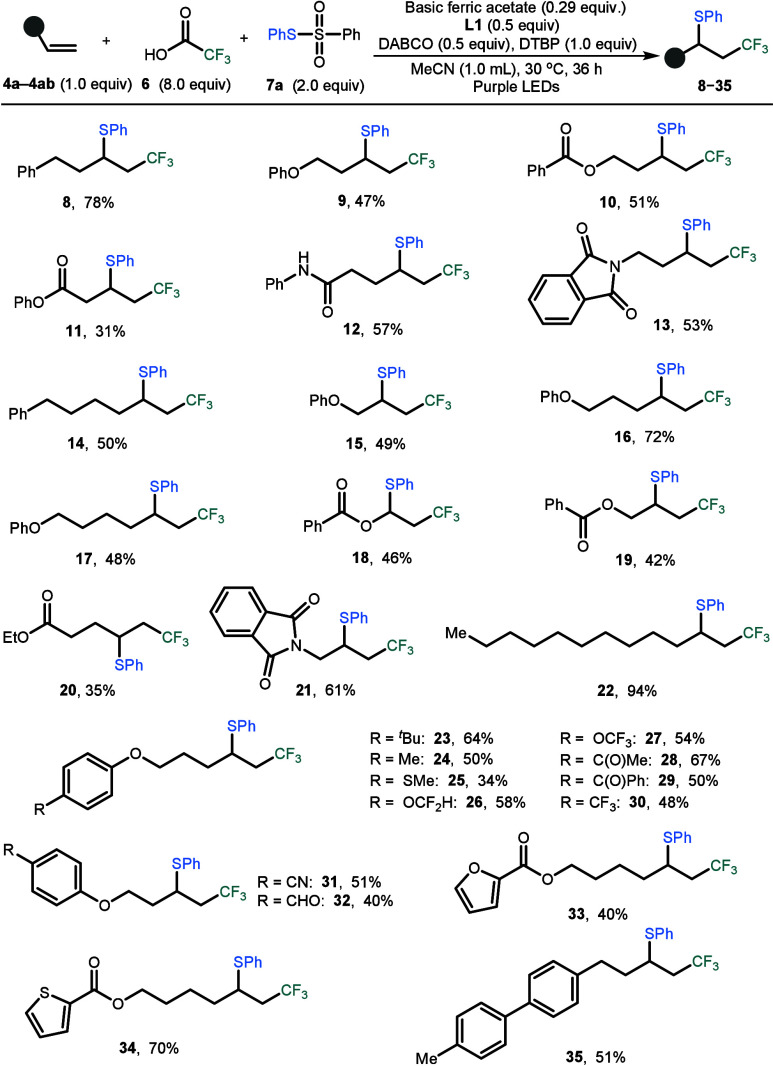
Scope of Alkenes^a^

a Reaction conditions: Alkene (**4a**–**4ab**, 0.10 mmol), CF_3_CO_2_H (**6**, 0.80 mmol), PhSSO_2_Ph (**7a**, 0.20 mmol), [Fe_3_O­(OAc)_6_(H_2_O)_3_]­OAc (0.029 mmol), DPA (**L1**, 0.05 mmol), DABCO (0.05 mmol), DTBP (0.10 mmol), MeCN (1.0 mL), purple LEDs (390 nm), ∼30 °C, argon atm, 36 h. Isolated yields are reported.

Next, we examined the scope of *S*-aryl benzenesulfonothioates (**7b**–**7t**) under the optimized reaction conditions (Scheme 2). A broad range of para-substituted aryl sulfonothioates bearing electron-donating groups (e.g., methoxy (**36**), *tert*-butyl (**37**)) and electron-withdrawing groups (e.g., fluoro (**38**), bromo (**39**), chloro (**40**), ester (**41**), and nitro (**42**)) on the aryl ring were well tolerated, affording the desired para-functionalized aryl trifluoropropyl sulfide products (**36**–**42**) in moderate to excellent yields. The position of the substituents had minimal impact on reactivity, with both meta- (**43**–**45**) and ortho-substituted (**46**–**51**) arenes delivering the target products efficiently. Pendant groups on the aromatic rings—including *tert*-butyl (**37**), fluoro (**38**, **43**, **48**), bromo (**39**), chloro (**40**, **49**), ester (**41**, **50**), nitro (**42**, **45**), trifluoromethyl (**44**), methyl (**47**), and amide (**51**) substituents—serve as versatile handles for drug design by enabling further chemical transformations and tuning physicochemical properties. Notably, *S*-aryl sulfonothioates bearing poly- and heteroaromatic motifs such as naphthyl (**52**), thienyl (**53**), and pyridyl (**54**) groups were also well tolerated, further highlighting the broad substrate scope and generality of this transformation.

**Scheme 2:**
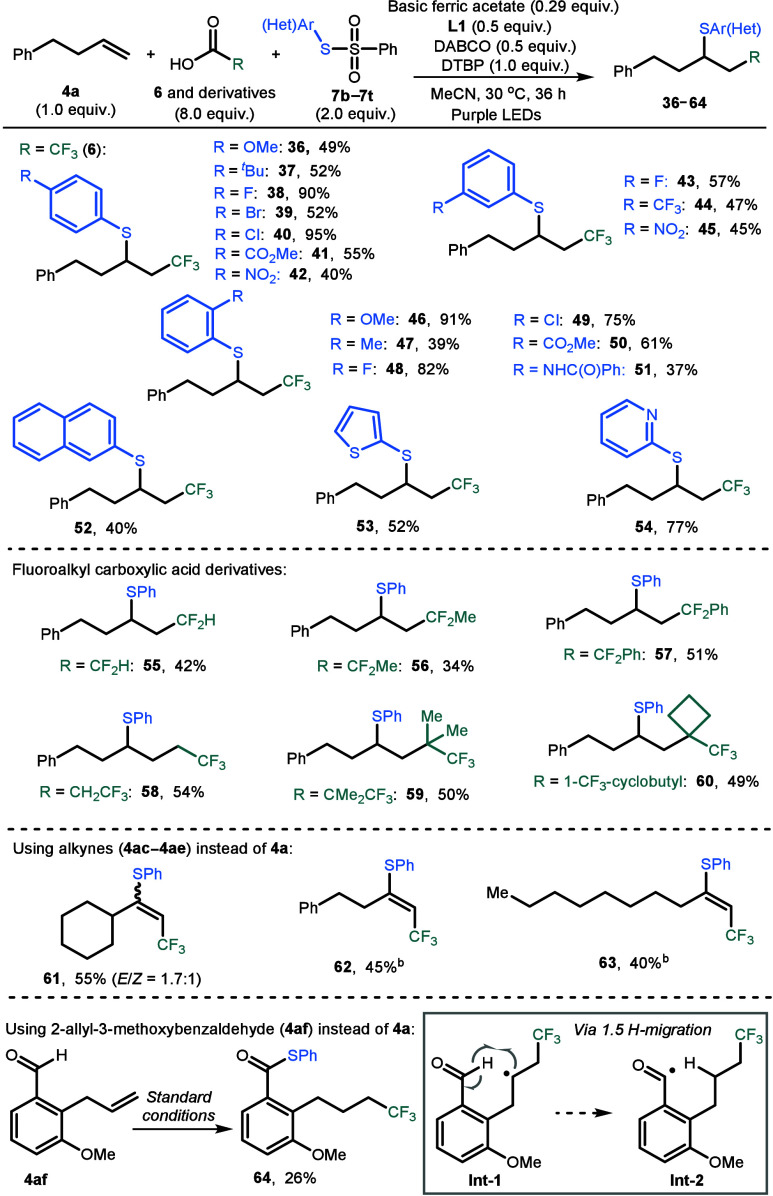
Scope of *S*-Aryl Benzenesulfonothioates, Fluorinated Aliphatic Acids, and Unsaturated Substrates^a^

a Reaction conditions: Alkene or alkyne (**4a**, **4ac**–**4af**, 0.10 mmol), CF_3_CO_2_H and derivatives (**6**, 0.80 mmol), ArSSO_2_Ph (**7b**–**7t**, 0.20 mmol), [Fe_3_O­(OAc)_6_(H_2_O)_3_]­OAc (0.029 mmol), DPA (**L1** **,** 0.05 mmol), DABCO (0.05 mmol), DTBP (0.10 mmol), MeCN (1.0 mL), purple LEDs (390 nm), ∼30 °C, argon atm, 36 h; isolated yields are reported.
b A trace amount of *Z*-isomer is detected by GC-MS analysis.

Furthermore, we explored the reactivity of other fluorinated aliphatic acids in this three-component coupling reaction. In addition to trifluoroacetic acid, difluoroacetic acid, 2,2-difluoropropionic acid and difluorophenylacetic acid were successfully incorporated, affording the corresponding aryl 3,3-difluoropropyl sulfides (**55**–**57**) in synthetically useful yields. Higher homologues bearing trifluoromethyl groups, including 3,3,3-trifluoropropionic acid, 2,2-dimethyl-3,3,3-trifluoropropionic acid, and (1-trifluoromethyl)­cyclobutanecarboxylic acid, were also viable reaction partners, delivering structurally more complex 4,4,4-trifluoropropyl sulfide products (**58**–**60**). Alkynes (**4ac**–**4ae**) were likewise competent substrates, undergoing thiotrifluoromethylation to furnish phenyl trifluoropropenyl sulfides (**61**–**63**). Notably, when 2-allyl-3-methoxybenzaldehyde (**4af**) was employed, a rearranged product, namely a trifluorobutyl-substituted *S*-phenyl benzothioate (**64**), was isolated. This outcome is consistent with a 1,5-hydrogen atom transfer of the trifluoroalkyl radical intermediate (**Int-1**), generating an acyl radical (**Int-2**) that subsequently undergoes C–S bond formation. Collectively, these results underscore the utility of this transformation for the efficient incorporation of structurally diverse fluoroalkyl groups and unsaturated compounds into complex thioether architectures.

To demonstrate the practicality and utility of the developed thiotrifluoromethylation strategy, several downstream applications were explored (Scheme 3). First, a large-scale reaction was successfully conducted using 4-phenylbut-1-ene, trifluoroacetic acid, and *S*-phenyl benzenesulfonothioate, affording the desired product **8** in 69% yield (1.07 g), which is comparable to the yield obtained on the 0.10 mmol scale (78%, Scheme 1). This result highlights the scalability of the protocol (Scheme 3A). The synthetic versatility of the method was further demonstrated through the late-stage functionalization of structurally complex drug molecules, natural products, and functional materials bearing alkene moieties (Scheme 3B). A variety of trifluoropropyl thioether-containing derivatives were synthesized, including functionalized analogues of probenecid (**65**), indomethacin (**66**), menthol (**67**), flavone (**68**), oestrone (**69**), and a liquid crystal building block (**70**). Finally, the derivatization of the aryl trifluoropropyl thioether motif was explored using compound **8** as a representative substrate (Scheme 3C). Oxidation of **8** with *m*-chloroperoxybenzoic acid furnished sulfone **71**, while treatment with hydrogen peroxide and chlorotrimethylsilane yielded sulfoxide **72**. Additionally, a one-pot sequence involving ammonium carbamate and PhI­(OAc)_2_ enabled access to sulfoximine **73**. These transformations showcase the chemical versatility of fluorinated thioether scaffolds, enabling diverse functionalizations for potential applications in pharmaceuticals and materials science. The structure of compound **71** was unambiguously confirmed by single-crystal X-ray diffraction analysis, which further verified the regioselective addition of the CF_3_ group to the terminal alkenyl carbon, followed by installation of the thio group at the internal alkenyl carbon.

**Scheme 3:**
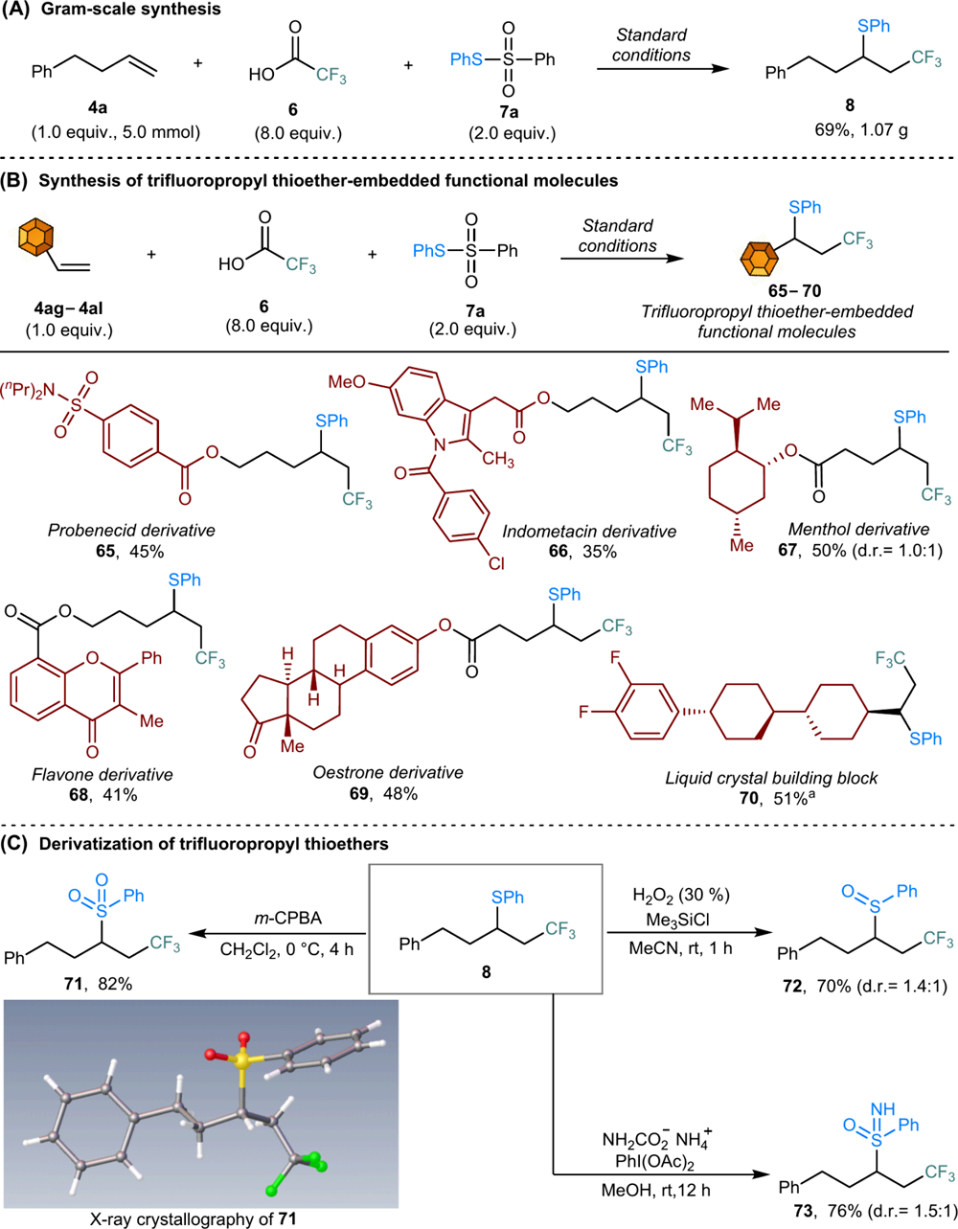
Synthetic Utility

a The α-stereogenic carbon of the thiotrifluoroalkyl moiety is racemic.

To elucidate the mechanism of the thiotrifluoromethylation reaction, a radical-trapping experiment was conducted in the presence of excess 2,2,6,6-tetramethylpiperidine-1-oxyl (TEMPO) under the standard conditions (Figure S6, Supporting Information). The reaction was markedly suppressed, delivering only a trace amount of the trifluoropropyl aryl sulfide product **8**. Meanwhile, several TEMPO–radical adducts (**S1**–**S3**), arising from the trifluoromethyl radical (**Rad-i**), trifluoroalkyl radical (**Rad-ii**), and phenylsulfonyl radical (**Rad-iii**), were detected by high-resolution mass spectrometry (HRMS). These observations strongly support a radical pathway. Specifically, CF_3_CO_2_H undergoes Fe-promoted decarboxylative oxidation to generate **Rad-i**, which subsequently adds to alkene **4a** to form **Rad-ii**. The resulting **Rad-ii** then reacts with *S*-phenyl benzenesulfonothioate (**7a**) to furnish product **8**, concomitant with the release of **Rad-iii**. Furthermore, *tert*-butyl benzenesulfonate (**S4**) was identified by HRMS analysis as a coproduct in the model reaction (Figure S7A, Supporting Information). This finding provides additional evidence for the generation of **Rad-iii**, which is likely trapped by DTBP, thereby forming **S4**.

Based on control experiments and literature precedents, we propose the following mechanism for the thiotrifluoromethylation reaction (Figure S8, Supporting Information). Trifluoroacetic acid (CF_3_CO_2_H, **6**) is initially deprotonated by DABCO to generate the trifluoroacetate anion (**6′**), which then undergoes ligand substitution with basic ferric acetate to form MeCN-coordinated^11^ and **L1**-ligated^15^ Fe­(III) trifluoroacetate complexes, **Int-3** and **Int-4**. These complexes feature coordinated trifluoroacetate anions that are further stabilized by hydrogen-bonding interactions with CF_3_CO_2_H.^11,15^ **Int-3** may further react with **L1** to afford **Int-4**. Both complexes were detected by HRMS analysis (Figure S7B, Supporting Information), supporting their formation under the reaction conditions. Upon photoexcitation of **Int-3** and **Int-4** to **Int-5** and **Int-6**, respectively, this coordination environment facilitates LMCT from the trifluoroacetate ligand to the Fe­(III) center, leading to the formation of the trifluoroacetate radical (**6·**) and the corresponding Fe­(II) species, **Int-7** and **Int-8**. The trifluoroacetate radical then rapidly undergoes decarboxylation to generate the CF_3_ radical (**Rad-i**), which adds to the terminal carbon of alkene substrate **4** to give the 3,3,3-trifluoropropyl radical intermediate **Rad-ii**. This carbon-centered radical subsequently reacts with *S*-aryl benzenesulfonothioate **7** to furnish the desired aryl 3,3,3-trifluoropropyl sulfide product **5**, accompanied by the release of the phenylsulfonyl radical (**Rad-iii**). DTBP is proposed to play dual roles in this transformation. First, DTBP may promote the oxidation of the Fe­(II) species **Int-7** and **Int-8**, thereby regenerating the LMCT-active Fe­(III) complexes **Int-3** and **Int-4** and potentially mitigating catalyst deactivation caused by arylthio-containing byproducts. Second, DTBP may intercept **Rad-iii** to form *tert*-butyl benzenesulfonate (**S4**), generating a *tert*-butoxy radical (*t*-BuO·) in the process. The resulting *t*-BuO· may further facilitate the reoxidation of low-valent iron species, thereby sustaining efficient radical turnover.

In summary, we have developed a light-driven, iron­(III)-promoted thiotrifluoromethylation of alkenes using CF_3_CO_2_H as an inexpensive CF_3_ radical source and *S*-aryl benzenesulfonothioates as arylthio donors. This method provides a modular and economical route to aryl 3,3,3-trifluoropropyl sulfides via iron-mediated LMCT activation of trifluoroacetate, avoiding prefunctionalized CF_3_ reagents and noble-metal catalysts. Its broad scope, scalability, and downstream derivatization highlight the synthetic utility of this protocol and the potential value of the resulting fluorinated thioethers.

## Supplementary Material



## Data Availability

The data underlying this study are available in the published article and its Supporting Information.

## Notes

Experimental details, supplementary figures and schemes, spectral data of all compounds, and X-ray crystal data (PDF)
