# Supplementary material for: Light-Driven Iron-Mediated Thiotrifluoromethylation of Alkenes Using CF3CO2H
Source: Org Lett. 2026 Jun 9;28(24):7857–62. doi: 10.1021/acs.orglett.6c02069 (PMC13288890; doi:10.1021/acs.orglett.6c02069)
Supplement: Supplementary file 1 [file ol6c02069_si_001.pdf]

Supporting Information

Light-Driven Iron-Mediated Thiotrifluoromethylation of Alkenes Using CF_3CO_2H

Ying-Hui Zhou[1], Ting Zhou[1], Chi Wai Cheung[2]*, Jun-An Ma[1]*

[1]Department of Chemistry, State Key Laboratory of Synthetic Biology, Tianjin University, Tianjin 300072, P. R. of China

[2]State Key Laboratory of Synthetic Chemistry and Department of Chemistry, The Chinese University of Hong Kong, Shatin, New Territories, Hong Kong 999077, P. R. of China

*Corresponding E-mails: cw.cheung@cuhk.edu.hk (C.W.C.); majun_an68@tju.edu.cn (J.-A.M.)

Table of contents

General Considerations	S2
General Analytical Information	S2
General Reagent Information	S2
General Manipulation Considerations	S3
Supporting Results	S4
Synthesis of Starting Materials	S4
Optimization of Reaction Conditions	S6
Mechanistic Study	S9
Substrate Scope Study	S15
Diverse Functionalization of Phenyl(4,4,4-trifluoro-1-phenylbutan-2-yl)sulfane	S47
X-ray Crystallographic Analysis	S50
NMR Spectra	S52
References	S188

General Considerations

General Analytical Information. ^{1}H, ^{13}C and ^{19}F NMR spectra were recorded on Bruker AV 500 MHz spectrometer at 500 MHz (^{1}H NMR), 126 MHz (^{13}C NMR), and 471 MHz (^{19}F NMR, comp. pulse decoupling or no decoupling). Chemical shifts (δ) are reported in parts per million (ppm). All ^{1}H NMR spectra were referenced to tetramethylsilane (TMS, 0 ppm) or residual proton signals of d_1-chloroform ($CDCl_3$, 7.26 ppm). All ^{13}C NMR spectra were reported relative to the residual carbon signals of $CDCl_3$ (77.16 ppm) and were obtained with ^{1}H decoupling. All ^{19}F NMR spectra were referenced to trichlorofluoromethane ($CFCl_3$, 0 ppm). Coupling constants (J) are reported in hertz (Hz). Multiplicity was indicated as follows: s (singlet), d (doublet), t (triplet), q (quartet), p (pentet), dd (doublet of doublets) and m (multiplet). High resolution mass spectrometry (HRMS) spectra were obtained on a Bruker micrOTOF-QII instrument. GC-MS analyses were performed on a Shimadzu QP-2010 SE instrument. X-ray structural analysis was conducted on a Bruker APEX-II CCD instrument. Thin-layer chromatography (TLC) was performed on precoated GF254 silica gel plates (Qingdao Marine Chemical Inc.) and compounds were visualized under UV light (254 nm). Flash column chromatography for purification of compounds was carried out using silica gel (200–300 mesh, Qingdao Marine Chemical Inc.). The melting point was determined using an SGW®X-4A Melting Point Apparatus with Microscope.

General Reagent Information. Unless otherwise noted, all commercially available materials were used as received without further purification. All known starting materials were synthesized according to the literature procedures. Anhydrous acetonitrile (MeCN) and dichloromethane (CH_2Cl_2) were purchased from Energy Chemical and stored over activated 3Å molecular sieves. 4-Phenyl-1-butene (**4a**, 98% purity), trifluoroacetic acid (CF_3CO_2H, **6**, 99% purity) and di-*tert*-butyl peroxide (DTBP, 97% purity) were purchased from Meryer. DTBP was stored in the refrigerator at 4 °C. Triethylenediamine (DABCO, 98% purity), *S*-phenyl benzenesulfonothioate (**7a**, 97% purity) and bis(2-pyridylmethyl)amine (DPA, **L1**, 97% purity) were

purchased from Bidepharm. Specifically, basic ferric acetate ($[Fe_3O(OAc)_6(H_2O)_3]OAc$, CAS No. 10450-55-2) was purchased from Meryer.

General Manipulation Considerations. Unless otherwise noted, all photochemical reactions were performed in oven-dried vials (4 mL). Flash column chromatography for purification of compounds was carried out using silica gel (200–300 mesh, Qingdao Marine Chemical Inc.). Preparative thin-layer chromatography (PTLC) for purification of compounds was carried out using preparative TLC (Rushan Hailan Experimental Equipment Inc.). Thin-layer chromatography (TLC) was performed on precoated GF254 silica gel plates (Qingdao Marine Chemical Inc.), and compounds were visualized under UV light (254 nm). The eluents used for column chromatography, PTLC and TLC are reported as volume ratios (*v/v*). Dichloromethane (CH_2Cl_2) was used to elute the products from the silica gel during PTLC purification. Yields reported in this study refer to isolated yields unless otherwise noted. All new starting materials and products obtained from the photochemical reactions were characterized by ^1H, ^{13}C and ^{19}F NMR spectroscopy (where applicable) and high-resolution mass spectrometry (HRMS). The Kessil LED (390 nm, 40 W) setup for photocatalytic reactions was purchased from Taobao.

Supporting Results

Synthesis of Starting Materials

(i) Alkenes

Substrates **4a**, **4k**–**4o**, **4ab**, **4af** and **4al** were purchased from commercial sources and used without further purification. Other substrates were prepared according to previously reported literature procedures (Figure S1).[1-3]

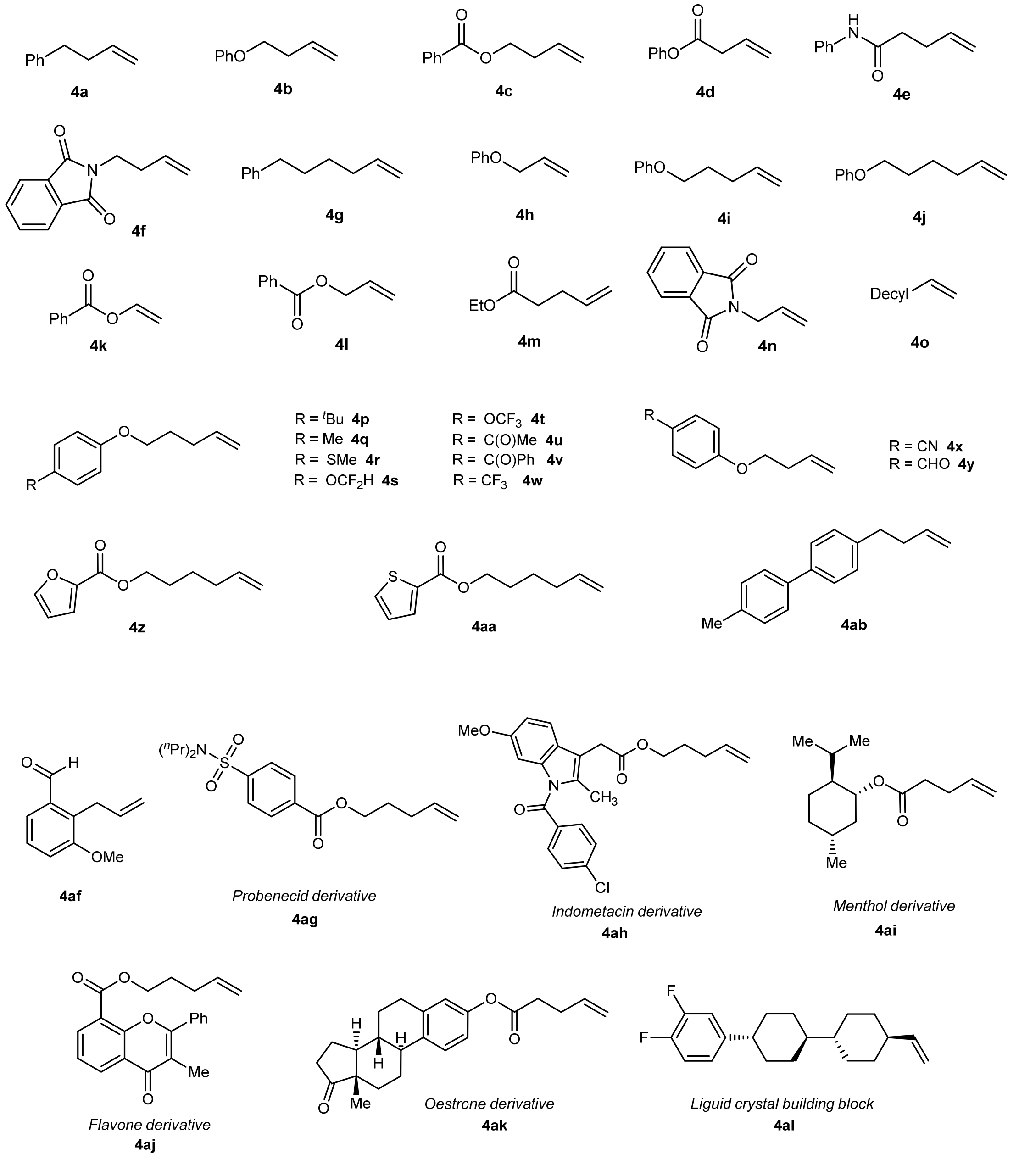

Figure S1. Alkene substrates used in the reaction.

(ii) Alkynes

All substrates were purchased from commercial sources and used without further purification (Figure S2).

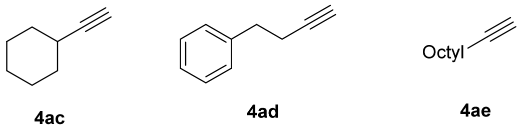

Figure S2. Alkyne substrates used in the reaction.

(iii) *S*-aryl arenesulfonothioates

All substrates were prepared according to the procedures reported in the literature (Figure S3).[2,4]

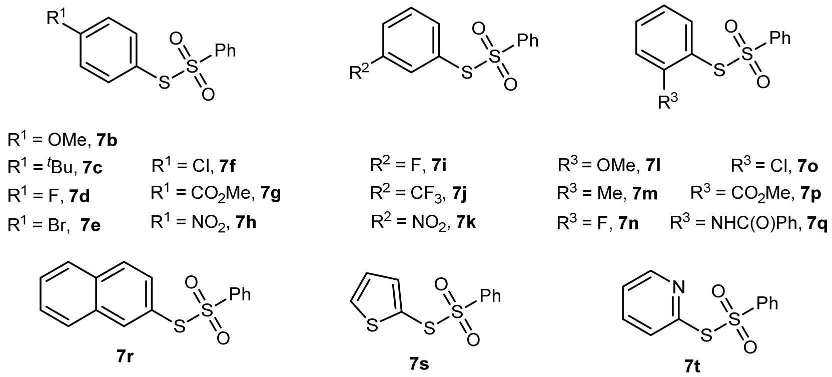

Figure S3. Sulfur reagents used in the reaction.

(iv) Fluorinated carboxylic acids

All substrates were purchased from commercial sources and used without further purification (Figure S4).

Other derivatives:

CF_2HCOOH $CF_2MeCOOH$ $CF_2PhCOOH$ CF_3CH_2COOH CF_3CMe_2COOH

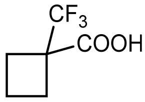

Figure S4. Fluorinated carboxylic acids used in the reaction.

Optimization of Reaction Conditions

Table S1. Optimization of iron-mediated thiotrifluoromethylation of Alkenes[a]

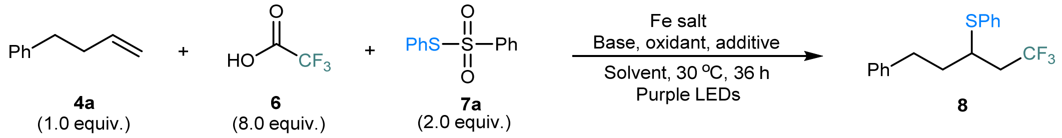

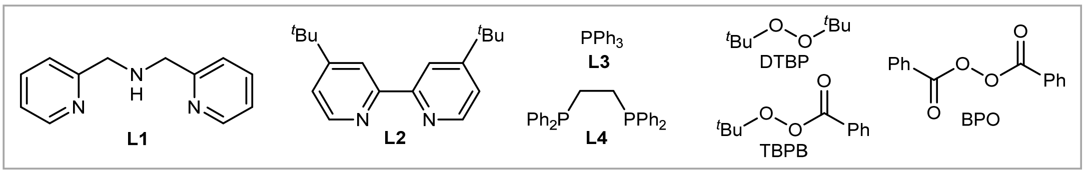

Entry	4a:6:7a (equiv.)	Fe salt (equiv.)	Base (equiv.)	Oxidant (equiv.)	Additive (equiv.)	Yield/%[b]
1	1:8:2	basic ferric acetate (0.29)	Cs_2CO_3 (0.5)	none	none	70
2	1:8:2	$FeCl_3\cdot6H_2O$ (1)	Cs_2CO_3 (0.5)	none	none	30
3	1:8:2	$Fe(OTf)_3$ (1)	Cs_2CO_3 (0.5)	none	none	41
4	1:8:2	basic ferric acetate (0.29)	K_2CO_3(0.5)	none	none	66
5	1:8:2	basic ferric acetate (0.29)	DBU (0.5)	none	none	67
6	1:8:2	basic ferric acetate (0.29)	DABCO (0.5)	none	none	74
7	1:8:2	basic ferric acetate (0.29)	DABCO (0.5)	$K_2S_2O_8$(1)	none	65
8	1:8:2	basic ferric acetate (0.29)	DABCO (0.5)	DTBP (1)	none	78
9	1:8:2	basic ferric acetate (0.29)	DABCO (0.5)	TBPB (1)	none	42
10	1:8:2	basic ferric acetate (0.29)	DABCO (0.5)	BPO (1)	none	51
11	1:8:2	basic ferric acetate (0.29)	DABCO (0.5)	DTBP(1)	**L1** (0.5)	88 (78)[c]
12	1:8:2	basic ferric acetate (0.29)	DABCO (0.5)	DTBP(1)	**L2** (0.5)	65
13	1:8:2	basic ferric acetate (0.29)	DABCO (0.5)	DTBP(1)	**L3** (0.5)	65
14	1:8:2	basic ferric acetate (0.29)	DABCO (0.5)	DTBP(1)	**L4** (0.5)	72
15	1:8:2	basic ferric acetate (0.14)	DABCO (0.5)	DTBP(1)	**L1** (0.5)	57
16	1:8:2	basic ferric acetate (0.05)	DABCO (0.5)	DTBP(1)	**L1** (0.5)	30
17	1:4:2	basic ferric acetate (0.29)	DABCO (0.5)	DTBP(1)	**L1** (0.5)	53
18	1:6:2	basic ferric acetate (0.29)	DABCO (0.5)	DTBP(1)	**L1** (0.5)	56
19	1:10:2	basic ferric acetate (0.29)	DABCO (0.5)	DTBP(1)	**L1** (0.5)	60
20	1:8:2	basic ferric acetate (0)	DABCO (0.5)	DTBP(1)	**L1** (0.5)	0
21	1:8:2	basic ferric acetate (0.29)	DABCO (0.5)	DTBP(1)	**L1** (0.5)	0[d]

[a] Reaction conditions: Alkene **4a** (0.10 mmol), CF_3CO_2H (**6**, 0.80 mmol), $PhSSO_2Ph$ (**7a**, 0.20 mmol), Fe salt ($FeCl_3\cdot6H_2O$, 1.0 equiv. 0.1 mmol, basic ferric acetate, $[Fe_3O(OAc)_6(H_2O)_3]OAc$, 0.29 equiv., 0.029 mmol), base (0.05 mmol), oxidant (0.10 mmol), additive (0.05 mmol), MeCN (1.0 mL), purple LEDs (390 nm), ~30 °C, argon atm, 36 h. [b] ^{19}F NMR yield using trifluoromethoxybenzene as an internal standard. [c] Isolated yield. [d] No purple LED irradiation was applied and the reaction is performed at room temperature under dark conditions.

General Procedure for Optimizations of Reaction Conditions of Iron-Mediated Thiotrifluoromethylation of Alkenes (Table S1):

An oven-dried, transparent 4 mL standard borosilicate glass vial equipped with a magnetic stir bar was sequentially charged with the *S*-phenyl benzenesulfonothioate (**7a**, 2.0 equiv., 0.20 mmol) and Fe salt ($FeCl_3 \cdot 6H_2O$ or $Fe(OTf)_3$: 1.0 equiv., 0.10 mmol; $[Fe_3O(OAc)_6(H_2O)_3]OAc$: 0.29 equiv., 0.029 mmol). When a solid base (0.5 equiv., 0.05 mmol), oxidant (1.0 equiv., 0.10 mmol), or additive (0.5 equiv., 0.05 mmol) was used, it was also added at this stage. The vial and septum screw cap were degassed three times in the transfer chamber of an argon-filled glovebox and then transferred into the main chamber. Inside the glovebox, anhydrous MeCN (1.0 mL) was added via syringe, and the vial was sealed with the septum screw cap before being removed from the glovebox. Subsequently, the 4-phenyl-1-butene (**4a**, 1.0 equiv., 0.10 mmol), liquid base, if applicable (0.5 equiv., 0.05 mmol), liquid oxidant, if applicable (1.0 equiv., 0.10 mmol), liquid additive, if applicable (0.5 equiv., 0.05 mmol), and trifluoroacetic acid (CF_3CO_2H, **6**, 8.0 equiv., 0.80 mmol) were added via microsyringe. The vial was further sealed with Parafilm, and the reaction mixture was vigorously stirred and irradiated with 40 W purple Kessil LEDs (λ = 390 nm) for 36 h at an ambient temperature of approximately 30 °C with fan cooling. After completion, the reaction mixture was diluted with ethyl acetate (~100 mL) and washed with water (~50 mL × 4). The organic layer was dried over anhydrous Na_2SO_4, filtered, and concentrated in vacuo. The residue was purified by preparative thin-layer chromatography using a mixture of petroleum ether and ethyl acetate as the eluent to afford the desired product **8**. Typically, 4–5 reaction vials were irradiated simultaneously, with the distance between the light source and the vials maintained at approximately 8 cm. No optical filters were used. Further details of the experimental setup are provided in Figure S5.

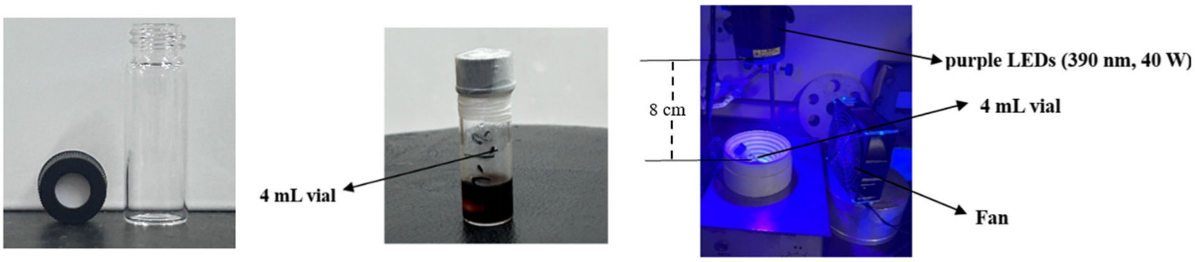

(A) **(B)** **(C)**

Figure S5. Experimental setup for the photochemical reaction. (A) A 4 mL borosilicate glass vial and septum screw cap used as the reaction vessel. (B) The sealed reaction vial after setup and additional sealing with Parafilm. (C) Irradiation of the vigorously stirred reaction mixture using 40 W purple Kessil LEDs.

Mechanistic Study

Radical Trap Experiment, Co-product Detection, and Iron species detection

(i) TEMPO-Radical Trapping Experiment

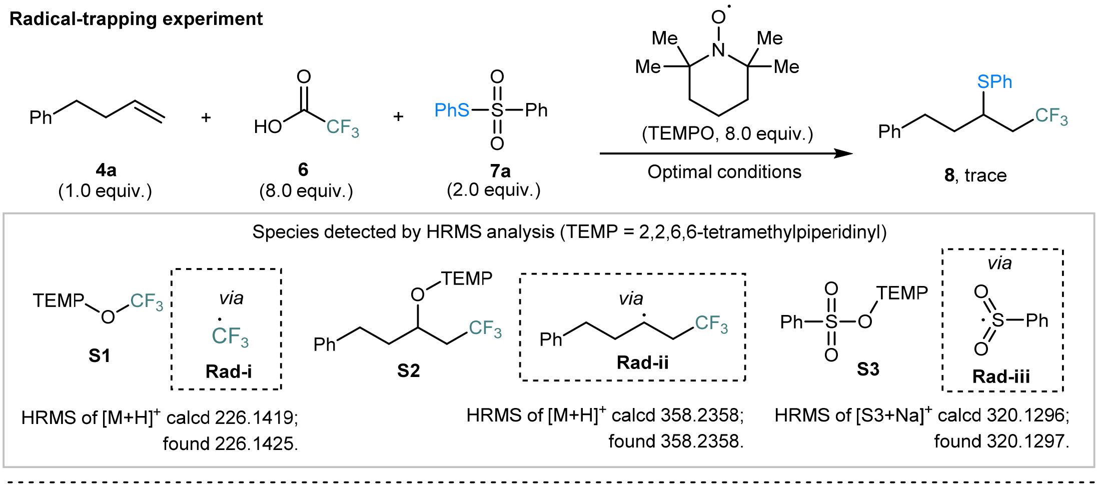

Figure S6. Identification of radical species using TEMPO as the radical trap.

Procedure for TEMPO-radical trap experiment

An oven-dried, transparent 4 mL standard borosilicate glass vial equipped with a magnetic stir bar was sequentially charged with *S*-phenyl benzenesulfonothioate (**7a**, 2.0 equiv., 0.20 mmol), basic ferric acetate ($[Fe_3O(OAc)_6(H_2O)_3]OAc$, 0.29 equiv., 0.029 mmol), triethylenediamine (DABCO, 0.5 equiv., 0.05 mmol), and 2,2,6,6-tetramethylpiperidine-1-oxyl (TEMPO, 8.0 equiv., 0.80 mmol). The vial and septum screw cap were degassed three times in the transfer chamber of an argon-filled glovebox and then transferred into the main chamber. Inside the glovebox, anhydrous MeCN (1.0 mL) was added via syringe, and the vial was sealed with the septum screw cap before being removed from the glovebox. Subsequently, 4-phenyl-1-butene (**4a**, 1.0 equiv., 0.10 mmol), di-*tert*-butyl peroxide (DTBP, 1.0 equiv., 0.10 mmol), bis(2-pyridylmethyl)amine (DPA, **L1**, 0.5 equiv., 0.05 mmol), and trifluoroacetic acid (CF_3CO_2H, **6**, 8.0 equiv., 0.80 mmol) were added via microsyringe. The vial was further sealed with Parafilm, and the reaction mixture was vigorously stirred and irradiated

with 40 W purple Kessil LEDs (λ = 390 nm) for 36 h at an ambient temperature of approximately 30 °C with fan cooling. The distance between the light source and the vial was maintained at approximately 8 cm, and no optical filters were used. Only a trace amount of product **8** was detected by TLC and GC-MS analysis, while the TEMPO-trapped adducts **S1–S3** were identified by HRMS analysis.

HRMS results:

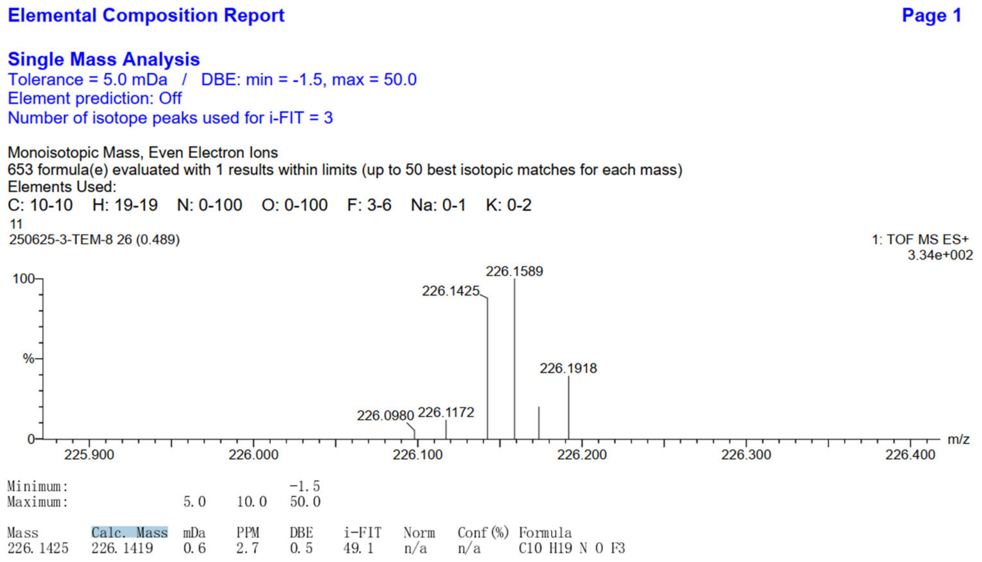

HRMS of [**S1**+H]$^+$ calcd 226.1419; found 226.1425

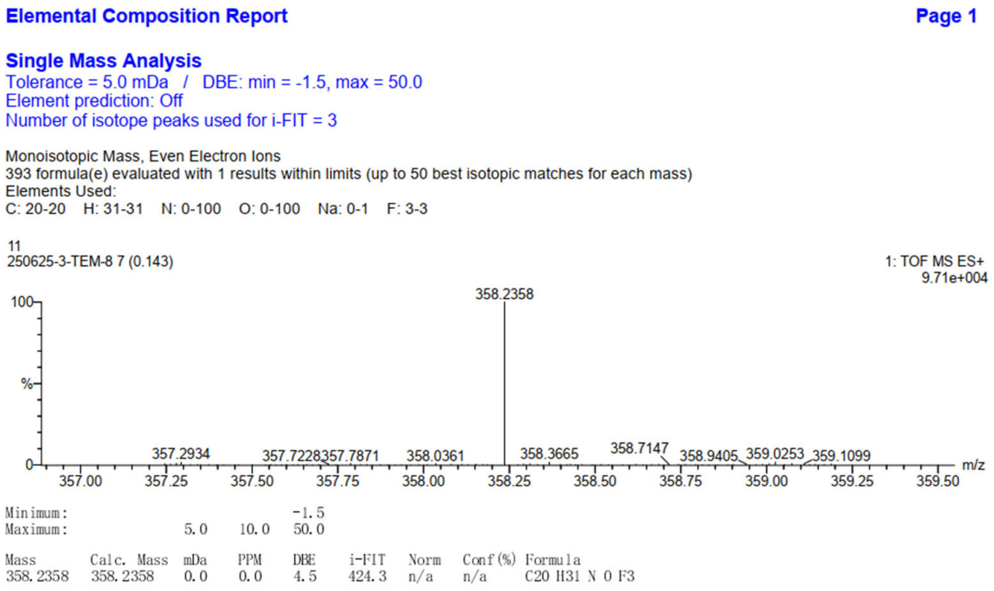

HRMS of [**S2**+H]$^+$ calcd 358.2358; found 358.2358

Elemental Composition Report Page 1

Single Mass Analysis
Tolerance = 5.0 mDa / DBE: min = -1.5, max = 50.0
Element prediction: Off
Number of isotope peaks used for i-FIT = 3

Monoisotopic Mass, Even Electron Ions
1266 formula(e) evaluated with 1 results within limits (up to 50 best isotopic matches for each mass)
Elements Used:
C: 15-15 H: 23-23 N: 0-100 O: 0-100 Na: 0-1 S: 1-9
11
250625-3-TEM-8 7 (0.143) 1: TOF MS ES+ 3.30e+002

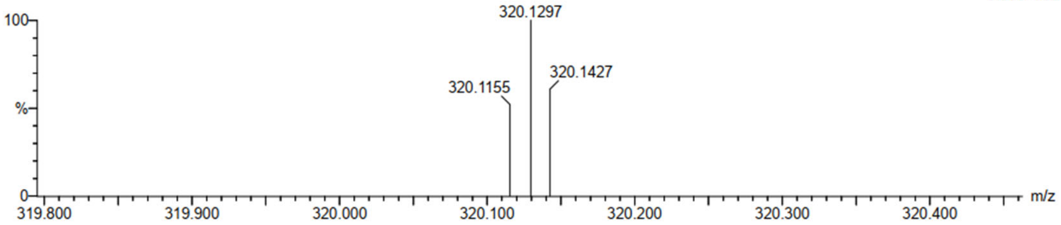

Minimum:				-1.5				
Maximum:		5.0	10.0	50.0				
Mass	Calc. Mass	mDa	PPM	DBE	i-FIT	Norm	Conf(%)	Formula
320.1297	320.1296	0.1	0.3	4.5	31.1	n/a	n/a	C15 H23 N O3 Na S

HRMS of [**S3**+Na]$^+$ calcd 320.1296; found 320.1297

(ii) Organic Co-product and Iron Species Detection

(A) Probing the formation of organic co-products

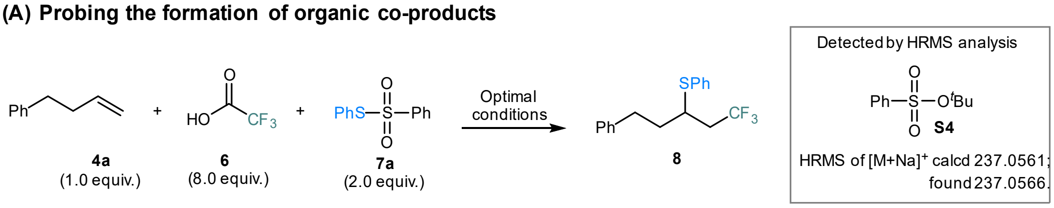

(B) Probing Fe species

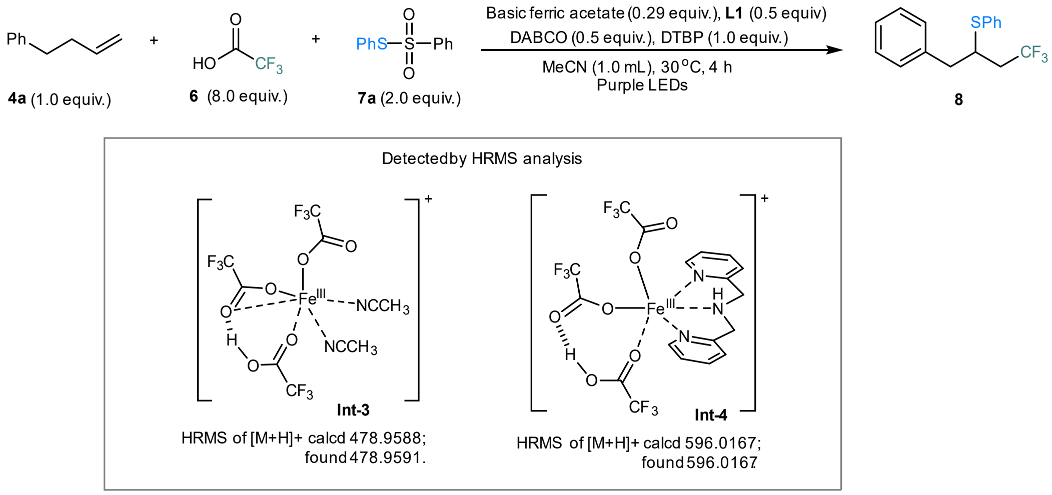

Figure S7. (A) Probing the formation of organic co-products in the model reaction. **(B)** Probing Fe species in the model reaction.

HRMS results:

Elemental Composition Report

Page 1

Single Mass Analysis
Tolerance = 5.0 mDa / DBE: min = -1.5, max = 50.0
Element prediction: Off
Number of isotope peaks used for i-FIT = 3

Monoisotopic Mass, Even Electron Ions
437 formula(e) evaluated with 1 results within limits (up to 50 best isotopic matches for each mass)
Elements Used:
C: 10-10 H: 14-14 N: 0-24 O: 0-100 Na: 0-1 S: 1-4
47
260117-2-3so 18 (0.119)

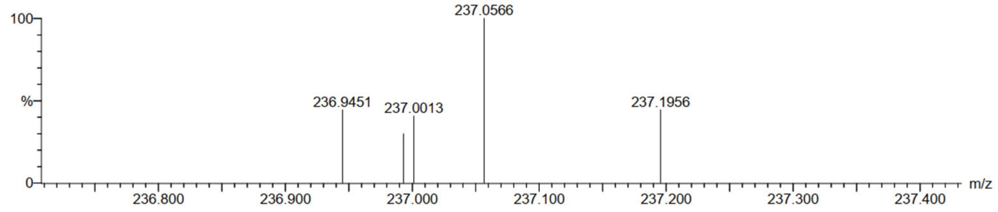

Minimum:				-1.5				
Maximum:		5.0	10.0	50.0				

Mass	Calc. Mass	mDa	PPM	DBE	i-FIT	Norm	Conf(%)	Formula
237.0566	237.0561	0.5	2.1	3.5	20.2	n/a	n/a	C10 H14 O3 Na S

HRMS of [**S4**+Na]$^{+}$ calcd 237.0561; found 237.0566

Elemental Composition Report

Page 1

Single Mass Analysis
Tolerance = 50.0 PPM / DBE: min = -100.0, max = 500.0
Element prediction: Off
Number of isotope peaks used for i-FIT = 3

Monoisotopic Mass, Even Electron Ions
9 formula(e) evaluated with 1 results within limits (all results (up to 1000) for each mass)
Elements Used:
C: 10-24 H: 0-200 N: 2-13 O: 6-6 F: 9-9 Fe: 1-1
3-P
260416-2-ZYH-4H-1 7 (0.085)

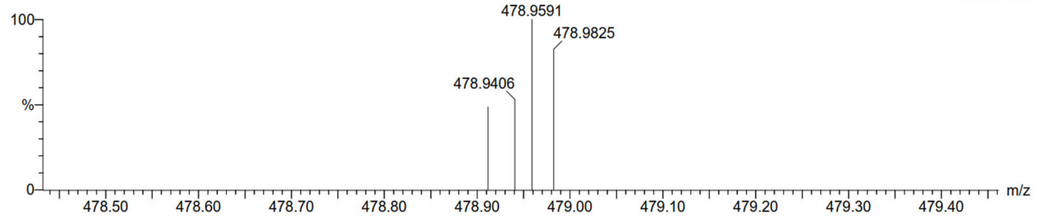

Minimum:				-100.0				
Maximum:		5.0	50.0	500.0				

Mass	Calc. Mass	mDa	PPM	DBE	i-FIT	Norm	Conf(%)	Formula
478.9591	478.9588	0.3	0.6	3.5	19.0	n/a	n/a	C10 H8 N2 O6 F9 Fe

HRMS of [**Int-3**+H]$^{+}$ calcd 478.9588; found 478.9591

Elemental Composition Report **Page 1**

Single Mass Analysis
Tolerance = 50.0 PPM / DBE: min = -100.0, max = 500.0
Element prediction: Off
Number of isotope peaks used for i-FIT = 3

Monoisotopic Mass, Even Electron Ions
131 formula(e) evaluated with 1 results within limits (all results (up to 1000) for each mass)
Elements Used:
C: 18-18 H: 0-200 N: 2-13 O: 6-66 Fe: 1-1 F: 9-9

3-P
260416-2-ZYH-4H-1 19 (0.196) 1: TOF MS ES+
9.22e+002

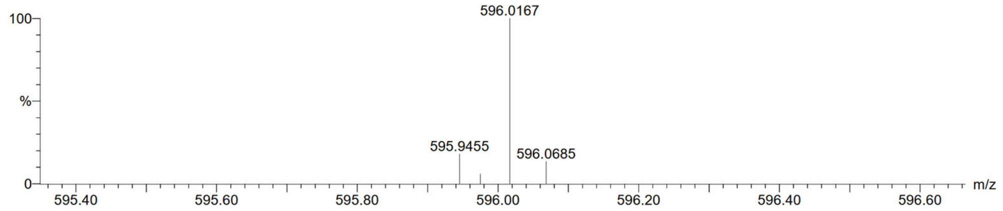

	Mass	Calc. Mass	mDa	PPM	DBE	i-FIT	Norm	Conf(%)	Formula
Minimum:					-100.0				
Maximum:			5.0	50.0	500.0				
	596.0167	596.0167	0.0	0.0	8.5	17.8	n/a	n/a	C18 H15 N3 O6 Fe F9

HRMS of [**Int-4**+H]$^+$ calcd 596.0167; found 596.0167

(iii) Proposed Mechanism of the Iron-Mediated Thiotrifluoromethylation of alkenes

Based on control experiments and literature precedents, we propose the following mechanism for the thiotrifluoromethylation reaction (Figure S8). Trifluoroacetic acid (CF_3CO_2H, **6**) is initially deprotonated by DABCO to generate the trifluoroacetate anion (**6′**), which then undergoes ligand substitution with ferric acetate to form MeCN-coordinated and **L1**-ligated Fe(III) trifluoroacetate complexes, **Int-3** and **Int-4**. These complexes feature coordinated trifluoroacetate anions that are further stabilized by hydrogen-bonding interactions with CF_3CO_2H. **Int-3** may further react with **L1** to afford **Int-4**. Both complexes were detected by HRMS analysis, supporting their formation under the reaction conditions. Upon photoexcitation of **Int-3** and **Int-4** to **Int-5** and **Int-6**, respectively, this coordination environment facilitates LMCT from the trifluoroacetate ligand to the Fe(III) center, leading to the formation of the trifluoroacetate radical (**6·**) and the corresponding Fe(II) species, **Int-7** and **Int-8**. The trifluoroacetate radical then rapidly undergoes decarboxylation to generate the CF_3 radical (**Rad-i**), which adds to the terminal carbon of alkene substrate **4** to give the 3,3,3-trifluoropropyl radical intermediate **Rad-ii**. This carbon-centered radical

subsequently reacts with *S*-aryl benzenesulfonothioate **7** to furnish the desired aryl 3,3,3-trifluoropropyl sulfide product **5**, accompanied by release of the phenylsulfonyl radical (**Rad-iii**). DTBP is proposed to play dual roles in this transformation. First, DTBP may promote the oxidation of the Fe(II) species **Int-7** and **Int-8**, thereby regenerating the LMCT-active Fe(III) complexes **Int-3** and **Int-4** and potentially mitigating catalyst deactivation caused by arylthio-containing byproducts. Second, DTBP may intercept **Rad-iii** to form *tert*-butyl benzenesulfonate (**S4**), generating a *tert*-butoxy radical (*t*-BuO·) in the process. The resulting *t*-BuO· may further facilitate the reoxidation of low-valent iron species, thereby sustaining efficient radical turnover.

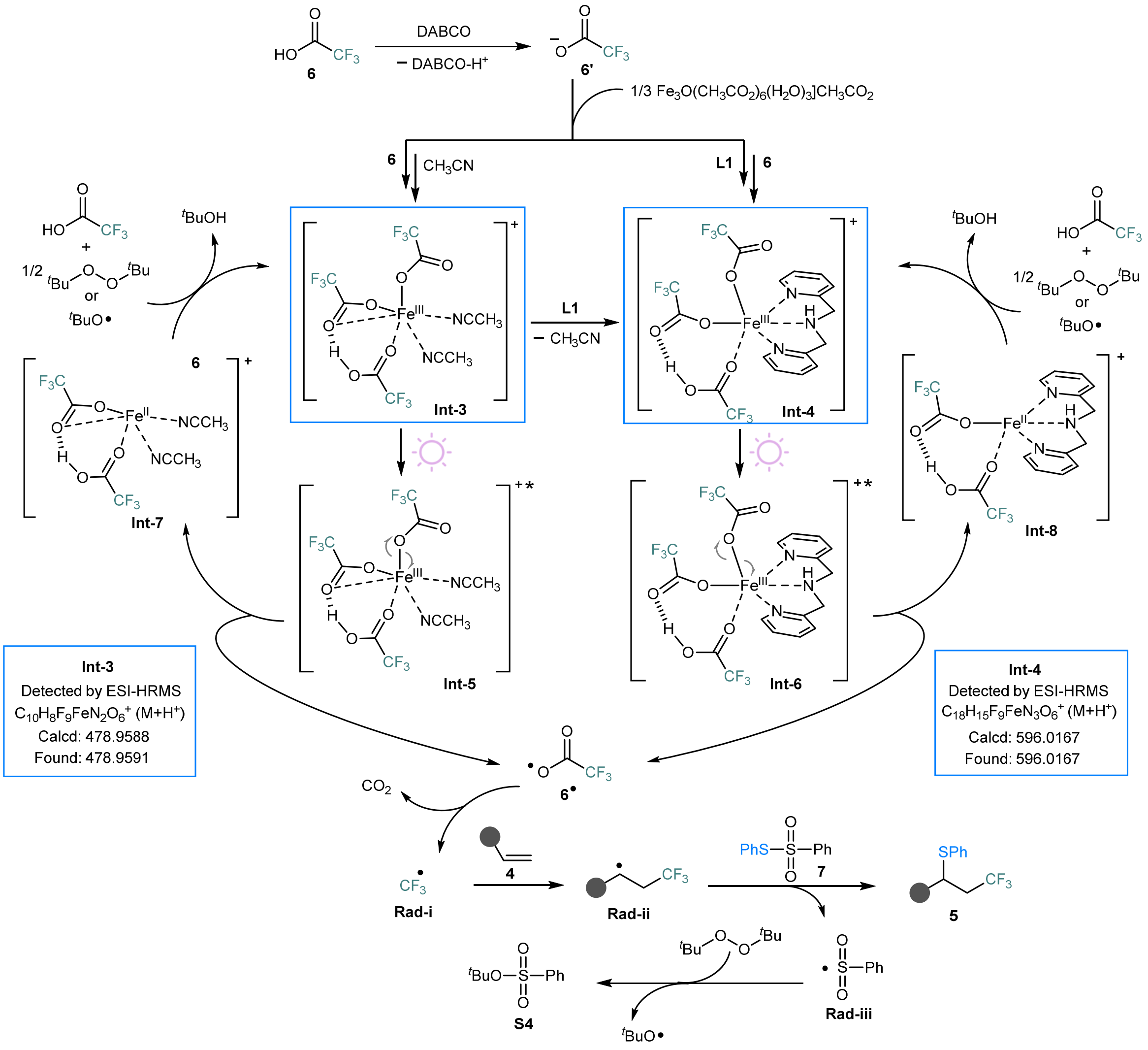

Figure S8. Proposed mechanism of the iron-mediated thiotrifluoromethylation of alkenes.

Substrate Scope Study

General Procedure for Iron-mediated thiotrifluoromethylation of alkenes (General Procedure A)

An oven-dried 4 mL standard borosilicate glass vial equipped with a magnetic stir bar was sequentially charged with the *S*-aryl arenesulfonothioate (**7a**–**7t**, 2.0 equiv., 0.20 mmol), basic ferric acetate ($[Fe_3O(OAc)_6(H_2O)_3]OAc$, 0.29 equiv., 0.029 mmol), and triethylenediamine (DABCO, 0.5 equiv., 0.05 mmol). The vial and septum screw cap were degassed three times in the transfer chamber of an argon-filled glovebox and then transferred into the main chamber. Inside the glovebox, anhydrous acetonitrile (MeCN, 1.0 mL) was added via syringe, and the vial was sealed with the septum screw cap before being removed from the glovebox. Subsequently, the alkene (**4a**–**4ab**, **4af**–**4al**, 1.0 equiv., 0.10 mmol) or alkyne (**4ac**–**4ae**, 1.0 equiv., 0.10 mmol), di-*tert*-butyl peroxide (DTBP, 1.0 equiv., 0.10 mmol), bis(2-pyridylmethyl)amine (DPA, **L1**, 0.5 equiv., 0.05 mmol), and trifluoroacetic acid (**6**) or other fluorinated carboxylic acids (8.0 equiv., 0.80 mmol) were added via microsyringe. The vial was further sealed with Parafilm, and the reaction mixture was vigorously stirred and irradiated with 40 W purple Kessil LEDs (λ = 390 nm) for 36 h at an ambient temperature of approximately 30 °C with fan cooling. The distance between the light source and the vial was maintained at approximately 8 cm, and no optical filters were used. After completion, the reaction mixture was diluted with ethyl acetate (~20 mL) and washed with water (~20 mL × 4). The organic layer was dried over anhydrous Na_2SO_4, filtered, and concentrated in vacuo. The residue was purified by preparative thin-layer chromatography using a mixture of petroleum ether and ethyl acetate as the eluent to afford the desired products **8**–**70** (The experimental setup is shown in **Figure S5**).

Phenyl(1,1,1-trifluoro-5-phenylpentan-3-yl)sulfane (8)

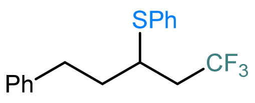

(a) 0.1 mmol scale: Using the General Procedure A, the title compound was obtained as yellow oil by preparative TLC using

petroleum ether as the eluent; R_f = 0.6 (petroleum ether); 24.1 mg, 78% yield; **^{1}H NMR** (500 MHz, $CDCl_3$) δ 7.42 – 7.37 (m, 2H), 7.33 – 7.23 (m, 5H), 7.22 – 7.14 (m, 3H), 3.36 – 3.25 (m, 1H), 2.96 – 2.88 (m, 1H), 2.86 – 2.77 (m, 1H), 2.51 – 2.41 (m, 1H), 2.40 – 2.27 (m, 1H), 2.15 – 2.05 (m, 1H), 1.93 – 1.79 (m, 1H). **^{13}C NMR** (126 MHz, $CDCl_3$) δ 141.0, 133.3, 132.9, 129.3, 128.6, 128.5, 127.9, 126.2, 126.1 (q, J = 278.1 Hz), 41.8 (q, J = 2.4 Hz), 39.2 (q, J = 27.5 Hz), 35.4, 32.6. **^{19}F NMR** (471 MHz, $CDCl_3$) δ –63.22 (t, J = 10.8 Hz). **HRMS** (ESI-TOF) *m/z*: $[M+H]^+$ Calcd for $C_{17}H_{18}F_3S^+$ 311.1081; Found 311.1083.

(b) 5.0 mmol scale (Gram-scale synthesis):

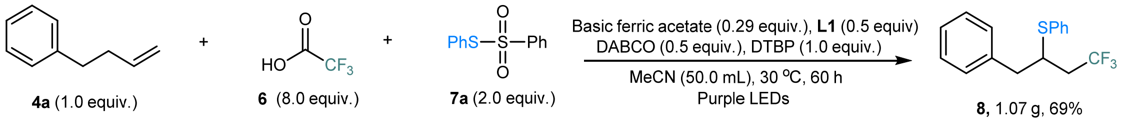

An oven-dried, transparent 100 mL standard borosilicate glass Schlenk tube equipped with a magnetic stir bar was sequentially charged with *S*-phenyl benzenesulfonothioate (**7a**, 2.0 equiv., 10.0 mmol), basic ferric acetate ($[Fe_3O(OAc)_6(H_2O)_3]OAc$, 0.29 equiv., 1.45 mmol), and triethylenediamine (DABCO, 0.5 equiv., 2.5 mmol). The Schlenk tube and rubber septum were degassed three times in the transfer chamber of an argon-filled glovebox and then transferred into the main chamber. The Schlenk tube was sealed with the rubber septum and then removed from the glovebox. Subsequently, an argon-filled balloon was attached to the sidearm to maintain a positive argon pressure, and anhydrous MeCN (50 mL), 4-phenyl-1-butene (**4a**, 1.0 equiv., 5.0 mmol), di-*tert*-butyl peroxide (DTBP, 1.0 equiv., 5.0 mmol), bis(2-pyridylmethyl)amine (DPA, **L1**, 0.5 equiv., 2.5 mmol), and trifluoroacetic acid (CF_3CO_2H, **6**, 8.0 equiv., 40 mmol) were added via syringe. The top of the Schlenk tube was further sealed with Parafilm, and the reaction mixture was vigorously stirred and irradiated with 40 W purple Kessil LEDs (λ = 390 nm) for 60 h at an ambient temperature of approximately 30 °C with fan cooling. The distance between the light source and the Schlenk tube was maintained at approximately 5 cm, and no optical filters were used. After completion, the reaction mixture was diluted with ethyl acetate (~100 mL) and washed with water (~50 mL ×

4). The organic layer was dried over anhydrous Na_2SO_4, filtered, and concentrated in vacuo. The residue was purified by flash column chromatography using petroleum ether/ethyl acetate as the eluent to afford product **8** (1.07 g, 69%). The experimental setup is shown in Figure S9.

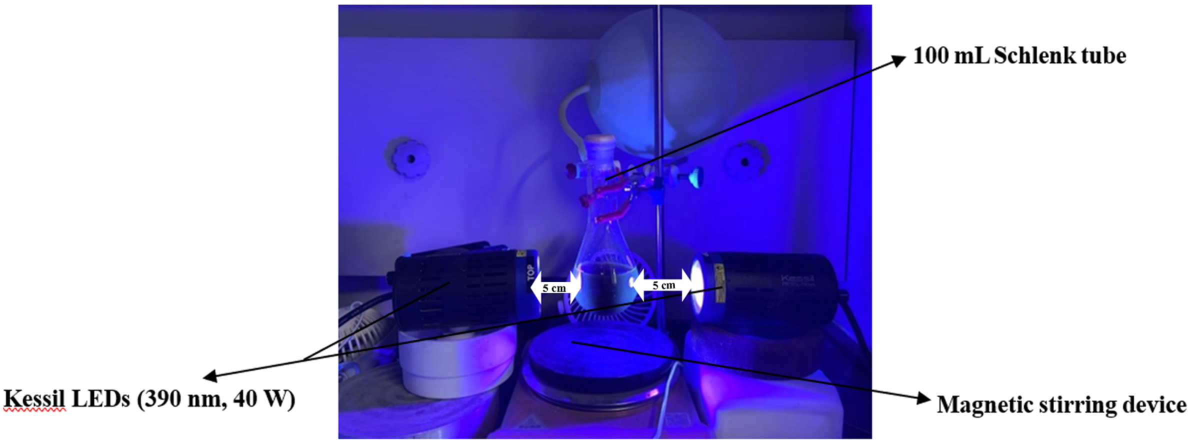

Figure S9. The experimental setup for gram-scale synthesis

Phenyl(1,1,1-trifluoro-5-phenoxypentan-3-yl)sulfane (9)

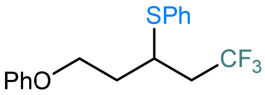

Using the General Procedure A, the title compound was obtained as yellow oil by preparative TLC using petroleum ether as the eluent; R_f = 0.6 (petroleum ether); 15.3 mg, 47% yield. ^{1}H NMR (500 MHz, $CDCl_3$) δ 7.47 – 7.40 (m, 2H), 7.37 – 7.23 (m, 5H), 6.99 – 6.89 (m, 3H), 4.29 – 4.23 (m, 1H), 4.22 – 4.16 (m, 1H), 3.66 – 3.59 (m, 1H), 2.61 – 2.50 (m, 1H), 2.49 – 2.40 (m, 1H), 2.38 – 2.29 (m, 1H), 2.07 – 1.94 (m, 1H). **^{13}C NMR** (126 MHz, $CDCl_3$) δ 158.8, 133.13, 133.06, 129.6, 129.4, 128.0, 126.1 (q, J = 278.2 Hz), 121.1, 114.7, 64.8, 39.8 (q, J = 2.6 Hz), 39.7 (q, J = 27.8 Hz), 33.7. **^{19}F NMR** (471 MHz, $CDCl_3$) δ –63.16 (t, J = 10.8 Hz). **HRMS** (ESI-TOF) *m/z*: $[M+H]^+$ Calcd for $C_{17}H_{18}OF_3S^+$ 327.1030; Found 327.1037.

5,5,5-trifluoro-3-(phenylthio)pentyl benzoate (10)

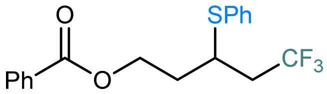

Using the General Procedure A, the title compound was obtained as yellow oil by preparative TLC using petroleum ether as the eluent; R_f = 0.5 (petroleum ether); 18.0 mg, 51%

yield. **^{1}H NMR** (500 MHz, $CDCl_3$) δ 8.05 – 7.97 (m, 2H), 7.61 – 7.54 (m, 1H), 7.48 – 7.41 (m, 4H), 7.36 – 7.28 (m, 3H), 4.66 – 4.48 (m, 2H), 3.58 – 3.41 (m, 1H), 2.62 – 2.50 (m, 1H), 2.49 – 2.36 (m, 1H), 2.34 – 2.26 (m, 1H), 2.07 – 1.91 (m, 1H). **^{13}C NMR** (126 MHz, $CDCl_3$) δ 166.5, 133.4, 133.2, 132.8, 130.1, 129.7, 129.4, 128.6, 128.3, 126.0 (q, J = 278.3 Hz), 62.1, 39.9 (q, J = 2.4 Hz), 39.7 (q, J = 27.6 Hz), 33.0. **^{19}F NMR** (471 MHz, $CDCl_3$) δ –63.28 (t, J = 10.7 Hz). **HRMS** (ESI-TOF) *m/z*: $[M+Na]^+$ Calcd for $C_{18}H_{17}O_2F_3NaS^+$ 377.0799; Found 377.0801.

Phenyl 5,5,5-trifluoro-3-(phenylthio)pentanoate (11)

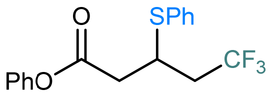

Using the General Procedure A, the title compound was obtained as yellow oil by preparative TLC using petroleum ether/EtOAc (20:1) as the eluent; R_f = 0.6 (petroleum ether/EtOAc = 20:1); 10.5 mg, 31% yield. **^{1}H NMR** (500 MHz, $CDCl_3$) δ 7.58 – 7.48 (m, 2H), 7.43 – 7.31 (m, 5H), 7.30 – 7.18 (m, 1H), 7.16 – 7.08 (m, 2H), 3.83 – 3.77 (m, 1H), 3.09 – 2.96 (m, 1H), 2.92 – 2.81 (m, 1H), 2.66 – 2.47 (m, 2H). **^{13}C NMR** (126 MHz, $CDCl_3$) δ 169.2, 150.6, 134.2, 131.9, 129.7, 129.5, 128.8, 126.2, 125.9 (q, J = 278.1 Hz), 121.7, 39.3, 38.9 (q, J = 2.4 Hz), 38.8 (q, J = 28.0 Hz). **^{19}F NMR** (471 MHz, $CDCl_3$) δ –63.38 (t, J = 10.6 Hz). **HRMS** (ESI-TOF) *m/z*: $[M+H]^+$ Calcd for $C_{17}H_{16}O_2F_3S^+$ 341.0823; Found 341.0824.

6,6,6-trifluoro-N-phenyl-4-(phenylthio)hexanamide (12)

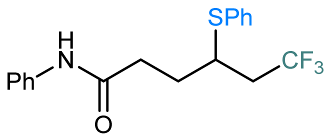

Using the General Procedure A, the title compound was obtained as yellow oil by preparative TLC using petroleum ether/EtOAc (5:1) as the eluent; R_f = 0.6 (petroleum ether/EtOAc = 5:1); 20.1 mg, 57% yield. **^{1}H NMR** (500 MHz, $CDCl_3$) δ 7.53 – 7.44 (m, 3H), 7.44 – 7.38 (m, 2H), 7.36 – 7.26 (m, 5H), 7.14 – 7.05 (m, 1H), 3.46 – 3.31 (m, 1H), 2.70 – 2.58 (m, 2H), 2.53 – 2.40 (m, 1H), 2.37 – 2.23 (m, 2H), 1.95 – 1.83 (m, 1H). **^{13}C NMR** (126 MHz, $CDCl_3$) δ 170.3, 137.9, 133.1, 132.7, 129.4, 129.1, 128.2, 126.0 (q, J = 278.3 Hz), 124.5, 120.1, 42.1 (q, J = 2.4 Hz), 39.6 (q, J = 27.7 Hz), 34.4,

29.5. **^{19}F NMR** (471 MHz, $CDCl_3$) δ –63.16 (t, J = 10.6 Hz). **HRMS** (ESI-TOF) *m/z*: $[M+Na]^+$ Calcd for $C_{18}H_{18}NOF_3NaS^+$ 376.0959; Found 376.0963.

2-(5,5,5-trifluoro-3-(phenylthio)pentyl)isoindoline-1,3-dione (13)

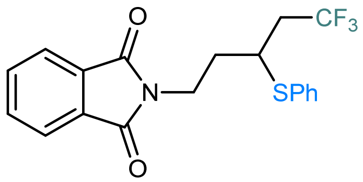

Using the General Procedure A, the title compound was obtained as yellow oil by preparative TLC using petroleum ether/EtOAc (5:1) as the eluent; R_f = 0.6 (petroleum ether/EtOAc = 5:1); 20.1 mg, 53% yield. **^{1}H NMR** (500 MHz, $CDCl_3$) δ 7.90 – 7.79 (m, 2H), 7.78 – 7.69 (m, 2H), 7.55 – 7.47 (m, 2H), 7.37 – 7.29 (m, 3H), 4.08 – 3.98 (m, 1H), 3.96 – 3.85 (m, 1H), 3.36 – 3.27 (m, 1H), 2.56 – 2.40 (m, 1H), 2.40 – 2.28 (m, 1H), 2.20 – 2.09 (m, 1H), 1.97 – 1.86 (m, 1H). **^{13}C NMR** (126 MHz, $CDCl_3$) δ 168.4, 134.14, 134.06, 132.2, 132.1, 129.4, 128.5, 125.9 (q, J = 278.2 Hz), 123.4, 40.6 (q, J = 3.1 Hz), 39.3 (q, J = 27.7 Hz), 35.8, 32.4. **^{19}F NMR** (471 MHz, $CDCl_3$) δ –63.29 (t, J = 10.5 Hz). **HRMS** (ESI-TOF) *m/z*: $[M+H]^+$ Calcd for $C_{19}H_{17}NO_2F_3S^+$ 380.0932; Found 380.0933.

Phenyl(1,1,1-trifluoro-7-phenylheptan-3-yl)sulfane (14)

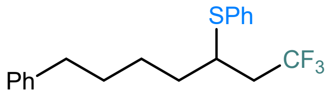

Using the General Procedure A, the title compound was obtained as yellow oil by preparative TLC using petroleum ether as the eluent; R_f= 0.7 (petroleum ether); 16.9 mg, 50% yield. **^{1}H NMR** (500 MHz, $CDCl_3$) δ 7.43 – 7.38 (m, 2H), 7.36 – 7.27 (m, 5H), 7.22 – 7.16 (m, 3H), 3.42 – 3.17 (m, 1H), 2.70 – 2.55 (m, 2H), 2.48 – 2.39 (m, 1H), 2.36 – 2.26 (m, 1H), 1.86 – 1.76 (m, 1H), 1.69 – 1.58 (m, 4H), 1.58 – 1.51 (m, 1H). **^{13}C NMR** (126 MHz, $CDCl_3$) δ 142.5, 133.6, 132.9, 129.3, 128.52, 128.45, 127.8, 126.2 (q, J = 278.3 Hz), 125.9, 42.5 (q, J = 2.1 Hz), 39.3 (q, J = 27.5 Hz), 35.8, 33.8, 31.2, 26.1. **^{19}F NMR** (471 MHz, $CDCl_3$) δ –63.37 (t, J = 10.9 Hz). **HRMS** (ESI-TOF) *m/z*: $[M+H]^+$ Calcd for $C_{19}H_{22}F_3S^+$ 339.1394; Found 339.1393.

Phenyl(4,4,4-trifluoro-1-phenoxybutan-2-yl)sulfane (15)

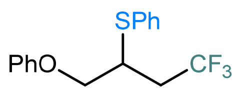

Using the General Procedure A, the title compound was obtained as yellow oil by preparative TLC using petroleum ether as the eluent; R_f= 0.8 (petroleum ether); 15.3 mg, 49% yield. **^{1}H NMR** (500 MHz, $CDCl_3$) δ 7.55 – 7.44 (m, 2H), 7.40 – 7.26 (m, 5H), 7.02 – 6.93 (m, 1H), 6.91 – 6.83 (m, 2H), 4.23 – 4.12 (m, 1H), 4.08 – 3.95 (m, 1H), 3.67 – 3.57 (m, 1H), 2.92 – 2.71 (m, 1H), 2.51 – 2.25 (m, 1H). **^{13}C NMR** (126 MHz, $CDCl_3$) δ 158.3, 133.4, 132.8, 129.7, 129.4, 128.4, 126.2 (q, J = 277.6 Hz), 121.5, 114.8, 69.0, 41.8 (q, J = 2.5 Hz), 35.8 (q, J = 28.8 Hz). **^{19}F NMR** (471 MHz, $CDCl_3$) δ –63.44 (t, J = 10.7 Hz). **HRMS** (ESI-TOF) *m/z*: $[M+Na]^+$ Calcd for $C_{16}H_{15}OF_3NaS^+$ 335.0693; Found 335.0695.

Phenyl(1,1,1-trifluoro-6-phenoxyhexan-3-yl)sulfane (16)

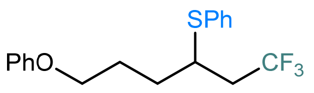

Using the General Procedure A, the title compound was obtained as yellow oil by preparative TLC using petroleum ether as the eluent; R_f= 0.8 (petroleum ether); 24.5 mg, 72% yield. **^{1}H NMR** (500 MHz, $CDCl_3$) δ 7.48 – 7.41 (m, 2H), 7.38 – 7.27 (m, 5H), 6.99 – 6.93 (m, 1H), 6.93 – 6.87 (m, 2H), 4.06 – 3.93 (m, 2H), 3.44 – 3.35 (m, 1H), 2.54 – 2.42 (m, 1H), 2.41 – 2.31 (m, 1H), 2.17 – 2.06 (m, 1H), 2.04 – 1.96 (m, 2H), 1.84 – 1.71 (m, 1H). **^{13}C NMR** (126 MHz, $CDCl_3$) δ 159.0, 133.21, 133.16, 129.6, 129.3, 128.0, 126.2 (q, J = 278.5 Hz), 120.8, 114.6, 67.3, 42.4 (q, J = 2.8 Hz), 39.3 (q, J = 27.6 Hz), 30.5, 26.4. **^{19}F NMR** (471 MHz, $CDCl_3$) δ –63.36 (t, J = 10.9 Hz). **HRMS** (ESI-TOF) *m/z*: $[M+Na]^+$ Calcd for $C_{18}H_{19}OF_3NaS^+$ 363.1006; Found 363.1008.

Phenyl(1,1,1-trifluoro-7-phenoxyheptan-3-yl)sulfane (17)

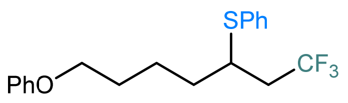

Using the General Procedure A, the title compound was obtained as yellow oil by preparative TLC using petroleum ether as the eluent; R_f= 0.7 (petroleum ether); 17.0 mg, 48% yield. **^{1}H NMR** (500 MHz, $CDCl_3$) δ 7.45 – 7.40 (m, 2H), 7.35 – 7.27 (m, 5H), 6.99 – 6.85 (m, 3H), 4.06 – 3.91 (m, 2H), 3.46 – 3.20 (m, 1H), 2.59 – 2.40 (m, 1H), 2.40 – 2.27 (m, 1H), 1.96 – 1.78 (m, 4H), 1.73 – 1.61 (m, 2H). **^{13}C NMR** (126

MHz, $CDCl_3$) δ 159.1, 133.4, 133.0, 129.6, 129.3, 127.9, 126.2 (q, J = 278.3 Hz), 120.8, 114.6, 67.5, 42.5 (q, J = 2.4 Hz), 39.3 (q, J = 27.5 Hz), 33.5, 29.0, 23.2. **^{19}F NMR** (471 MHz, $CDCl_3$) δ –63.35 (t, J = 10.8 Hz). **HRMS** (ESI-TOF) *m/z*: $[M+Na]^+$ Calcd for $C_{19}H_{21}OF_3NaS^+$ 377.1163; Found 377.1168.

3,3,3-trifluoro-1-(phenylthio)propyl benzoate (18)

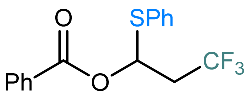

Using the General Procedure A, the title compound was obtained as yellow oil by preparative TLC using petroleum ether as the eluent; R_f = 0.5 (petroleum ether); 15.0 mg, 46% yield. **^{1}H NMR** (500 MHz, $CDCl_3$) δ 8.07 – 8.01 (m, 2H), 7.65 – 7.58 (m, 1H), 7.58 – 7.51 (m, 2H), 7.51 – 7.45 (m, 2H), 7.42 – 7.33 (m, 3H), 6.59 – 6.49 (m, 1H), 2.77 – 2.66 (m, 2H). **^{13}C NMR** (126 MHz, $CDCl_3$) δ 164.7, 135.5, 133.7, 130.0, 129.6, 129.4, 129.34, 129.29, 128.7, 125.0 (q, J = 278.0 Hz), 73.2 (q, J = 3.1 Hz), 39.5 (q, J = 29.2 Hz). **^{19}F NMR** (471 MHz, $CDCl_3$) δ –63.97 (t, J = 10.2 Hz). **HRMS** (ESI-TOF) *m/z*: $[M+Na]^+$ Calcd for $C_{16}H_{13}O_2F_3NaS^+$ 349.0486; Found 349.0483.

4,4,4-trifluoro-2-(phenylthio)butyl benzoate (19)

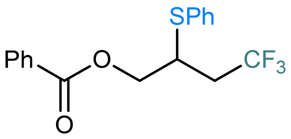

Using the General Procedure A, the title compound was obtained as yellow oil by preparative TLC using petroleum ether as the eluent; R_f = 0.6 (petroleum ether); 14.3 mg, 42% yield. **^{1}H NMR** (500 MHz, $CDCl_3$) δ 8.04 – 7.98 (m, 2H), 7.62 – 7.55 (m, 1H), 7.55 – 7.49 (m, 2H), 7.49 – 7.42 (m, 2H), 7.40 – 7.30 (m, 3H), 4.54 – 4.47 (m, 1H), 4.46 – 4.38 (m, 1H), 3.73 – 3.62 (m, 1H), 2.69 – 2.58 (m, 1H), 2.55 – 2.45 (m, 1H). **^{13}C NMR** (126 MHz, $CDCl_3$) δ 166.2, 133.6, 133.5, 132.2, 129.8, 129.6, 129.5, 128.62, 128.59, 126.0 (q, J = 278.0 Hz), 65.6, 41.3 (q, J = 2.5 Hz), 36.2 (q, J = 28.9 Hz). **^{19}F NMR** (471 MHz, $CDCl_3$) δ –63.58 (t, J = 10.6 Hz). **HRMS** (ESI-TOF) *m/z*: $[M+H]^+$ Calcd for $C_{17}H_{16}O_2F_3S^+$ 341.0823; Found 341.0827.

Ethyl 6,6,6-trifluoro-4-(phenylthio)hexanoate (20)

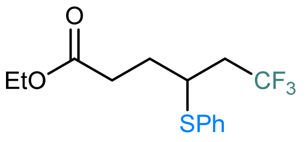

Using the General Procedure A, the title compound was obtained as yellow oil by preparative TLC using petroleum ether as the eluent; R_f= 0.6 (petroleum ether); 10.7 mg, 35% yield. **^{1}H NMR** (500 MHz, $CDCl_3$) δ 7.45 – 7.38 (m, 2H), 7.36 – 7.29 (m, 3H), 4.14 (q, J = 7.1 Hz, 2H), 3.47 – 3.22 (m, 1H), 2.69 – 2.54 (m, 2H), 2.53 – 2.43 (m, 1H), 2.36 – 2.23 (m, 1H), 2.23 – 2.11 (m, 1H), 1.89 – 1.75 (m, 1H), 1.26 (t, J = 7.0 Hz, 3H). **^{13}C NMR** (126 MHz, $CDCl_3$) δ 172.9, 133.3, 132.8, 129.4, 128.2, 126.0 (q, J = 278.6 Hz), 60.7, 42.1 (q, J = 2.4 Hz), 39.6 (q, J = 27.5 Hz), 31.4, 29.0, 14.3. **^{19}F NMR** (471 MHz, $CDCl_3$) δ –63.38 (t, J = 10.8 Hz). **HRMS** (ESI-TOF) *m/z*: $[M+Na]^+$ Calcd for $C_{14}H_{17}O_2F_3NaS^+$ 329.0799; Found 329.0803.

2-(4,4,4-trifluoro-2-(phenylthio)butyl)isoindoline-1,3-dione (21)

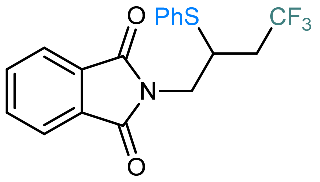

Using the General Procedure A, the title compound was obtained as yellow oil by preparative TLC using petroleum ether/EtOAc (15:1) as the eluent; R_f = 0.5 (petroleum ether/EtOAc = 15:1); 22.3 mg, 61% yield. **^{1}H NMR** (500 MHz, $CDCl_3$) δ 7.85 – 7.78 (m, 2H), 7.76 – 7.68 (m, 2H), 7.49 – 7.42 (m, 2H), 7.28 – 7.15 (m, 3H), 4.09 – 3.64 (m, 3H), 2.55 – 2.21 (m, 2H). **^{13}C NMR** (126 MHz, $CDCl_3$) δ 168.2, 134.3, 132.7, 132.3, 131.8, 129.3, 128.0, 125.9 (q, J = 277.7 Hz), 123.6, 42.2, 40.4 (q, J = 2.0 Hz), 37.4 (q, J = 29.0 Hz). **^{19}F NMR** (471 MHz, $CDCl_3$) δ –63.50 (t, J = 10.7 Hz). **HRMS** (ESI-TOF) *m/z*: $[M+H]^+$ Calcd for $C_{18}H_{15}NO_2F_3S^+$ 366.0776; Found 366.0779.

Phenyl(1,1,1-trifluorotridecan-3-yl)sulfane (22)

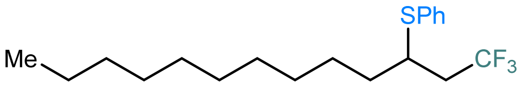

Using the General Procedure A, the title compound was obtained as yellow oil by preparative TLC using petroleum ether as the eluent; R_f=0.7 (petroleum ether); 32.5 mg, 94% yield. **^{1}H NMR** (500 MHz, $CDCl_3$) δ 7.44 – 7.38 (m, 2H), 7.35 – 7.27 (m, 3H), 3.36 – 3.29 (m, 1H), 2.48 – 2.38 (m, 1H), 2.37 – 2.26 (m, 1H), 1.82 – 1.72 (m, 1H), 1.62 – 1.54 (m, 1H), 1.52 – 1.45 (m, 1H), 1.31 – 1.20 (m, 15H), 0.89 (t, J = 6.8

Hz, 3H). **^{13}C NMR** (126 MHz, $CDCl_3$) δ 133.8, 132.8, 129.3, 127.7, 126.2 (q, J = 278.1 Hz), 42.5 (q, J = 2.4 Hz), 39.3 (q, J = 27.4 Hz), 33.9, 32.1, 29.74, 29.70, 29.6, 29.5, 29.4, 26.5, 22.8, 14.3. **^{19}F NMR** (471 MHz, $CDCl_3$) δ –63.43 (t, J = 11.0 Hz). **HRMS** (ESI-TOF) *m/z*: $[M+H]^+$ Calcd for $C_{19}H_{30}F_3S^+$ 347.2020; Found 347.2024.

(6-(4-(*tert*-butyl)phenoxy)-1,1,1-trifluorohexan-3-yl)(phenyl)sulfane (23)

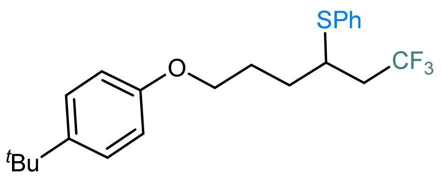

Using the General Procedure A, the title compound was obtained as yellow oil by preparative TLC using petroleum ether as the eluent; R_f =0.5 (petroleum ether); 25.3 mg, 64% yield. **^{1}H NMR** (500 MHz, $CDCl_3$) δ 7.50 – 7.42 (m, 2H), 7.39 – 7.27 (m, 5H), 6.88 – 6.81 (m, 2H), 4.02 – 3.94 (m, 2H), 3.49 – 3.31 (m, 1H), 2.55 – 2.32 (m, 2H), 2.17 – 2.10 (m, 1H), 2.06 – 1.95 (m, 2H), 1.83 – 1.73 (m, 1H), 1.32 (s, 9H). **^{13}C NMR** (126 MHz, $CDCl_3$) δ 156.7, 143.5, 133.23, 133.18, 129.3, 128.0, 126.4, 126.2 (q, J = 278.2 Hz), 114.1, 67.4, 42.4 (q, J = 2.6 Hz), 39.3 (q, J = 27.5 Hz), 34.2, 31.7, 30.6, 26.4. **^{19}F NMR** (471 MHz, $CDCl_3$) δ –63.31 (t, J = 10.8 Hz). **HRMS** (ESI-TOF) *m/z*: $[M+Na]^+$ Calcd for $C_{22}H_{27}OF_3NaS^+$ 419.1632; Found 419.1629.

Phenyl(1,1,1-trifluoro-6-(p-tolyloxy)hexan-3-yl)sulfane (24)

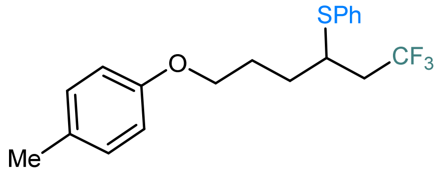

Using the General Procedure A, the title compound was obtained as yellow oil by preparative TLC using petroleum ether as the eluent; R_f = 0.6 (petroleum ether); 17.7 mg, 50% yield. **^{1}H NMR** (500 MHz, $CDCl_3$) δ 7.49 – 7.41 (m, 2H), 7.39 – 7.29 (m, 3H), 7.13 – 7.04 (m, 2H), 6.86 – 6.72 (m, 2H), 4.04 – 3.77 (m, 2H), 3.46 – 3.34 (m, 1H), 2.56 – 2.41 (m, 1H), 2.39 – 2.32 (m, 1H), 2.30 (s, 3H), 2.17 – 2.06 (m, 1H), 2.05 – 1.96 (m, 2H), 1.81 – 1.69 (m, 1H). **^{13}C NMR** (126 MHz, $CDCl_3$) δ 156.9, 133.2, 133.1, 130.1, 130.0, 129.3, 128.0, 126.2 (q, J = 278.1 Hz), 114.5, 67.4, 42.4 (q, J = 2.3 Hz), 39.3 (q, J = 27.6 Hz), 30.5, 26.4, 20.6. **^{19}F NMR** (471 MHz, $CDCl_3$) δ –63.34 (t, J = 10.9 Hz). **HRMS** (ESI-TOF)

m/z: [M+Na]$^+$ Calcd for $C_{19}H_{21}OF_3NaS^+$ 377.1163; Found 377.1165.

Methyl(4-((6,6,6-trifluoro-4-(phenylthio)hexyl)oxy)phenyl)sulfane (25)

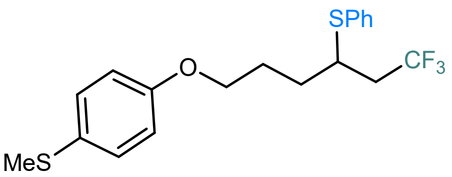

Using the General Procedure A, the title compound was obtained as yellow oil by preparative TLC using petroleum ether as the eluent; R_f= 0.5 (petroleum ether); 13.1 mg, 34% yield. **^{1}H NMR** (500 MHz, $CDCl_3$) δ 7.47 – 7.40 (m, 2H), 7.36 – 7.29 (m, 3H), 7.28 – 7.23 (m, 2H), 6.87 – 6.79 (m, 2H), 4.06 – 3.87 (m, 2H), 3.48 – 3.27 (m, 1H), 2.54 – 2.46 (m, 1H), 2.44 (s, 3H), 2.39 – 2.27 (m, 1H), 2.18 – 2.03 (m, 1H), 2.03 – 1.91 (m, 2H), 1.84 – 1.66 (m, 1H). **^{13}C NMR** (126 MHz, $CDCl_3$) δ 157.6, 133.2, 133.1, 130.3, 129.3, 129.0, 128.0, 126.1 (q, J = 278.4 Hz), 115.3, 67.5, 42.4 (q, J = 2.4 Hz), 39.3 (q, J = 27.6 Hz), 30.5, 26.3, 18.2. **^{19}F NMR** (471 MHz, $CDCl_3$) δ –63.36 (t, J = 11.1 Hz). **HRMS** (ESI-TOF) *m/z*: [M+Na]$^+$ Calcd for $C_{19}H_{21}OF_3NaS_2^+$ 409.0884; Found 409.0882.

(6-(4-(difluoromethoxy)phenoxy)-1,1,1-trifluorohexan-3-yl)(phenyl)sulfane (26)

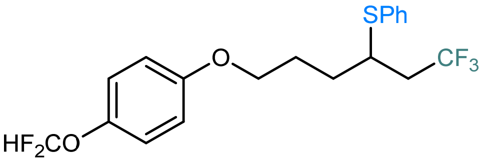

Using the General Procedure A, the title compound was obtained as yellow oil by preparative TLC using petroleum ether as the eluent; R_f= 0.5 (petroleum ether); 23.5 mg, 58% yield. **^{1}H NMR** (500 MHz, $CDCl_3$) δ 7.48 – 7.40 (m, 2H), 7.37 – 7.29 (m, 3H), 7.09 – 7.03 (m, 2H), 6.87 – 6.82 (m, 2H), 6.42 (t, J = 74.4 Hz, 1H), 3.96 (t, J = 5.8 Hz, 2H), 3.44 – 3.34 (m, 1H), 2.54 – 2.45 (m, 1H), 2.39 – 2.29 (m, 1H), 2.15 – 2.08 (m, 1H), 2.03 – 1.95 (m, 2H), 1.78 – 1.70 (m, 1H). **^{13}C NMR** (126 MHz, $CDCl_3$) δ 156.6, 144.7 (t, J = 3.0 Hz), 133.2, 133.1, 129.4, 128.1, 126.1 (q, J = 278.3 Hz), 121.5, 116.4 (t, J = 259.4 Hz), 115.4, 67.8, 42.3 (q, J = 2.4 Hz), 39.3 (q, J = 27.5 Hz), 30.4, 26.3. **^{19}F NMR** (471 MHz, $CDCl_3$) δ –63.37 (t, J = 10.7 Hz), –80.38 (d, J = 74.4 Hz). **HRMS** (ESI-TOF) *m/z*: [M+Na]$^+$ Calcd for $C_{19}H_{19}O_2F_5NaS^+$ 429.0924; Found 429.0931.

Phenyl(1,1,1-trifluoro-6-(4-(trifluoromethoxy)phenoxy)hexan-3-yl)sulfane (27)

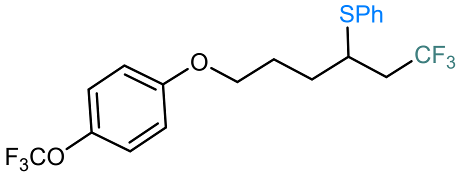

Using the General Procedure A, the title compound was obtained as yellow oil by preparative TLC using petroleum ether as the eluent; R_f = 0.7 (petroleum ether); 22.8 mg, 54% yield. **^{1}H NMR** (500 MHz, $CDCl_3$) δ 7.46 – 7.41 (m, 2H), 7.36 – 7.29 (m, 3H), 7.18 – 7.11 (m, 2H), 6.90 – 6.83 (m, 2H), 4.04 – 3.93 (m, 2H), 3.47 – 3.36 (m, 1H), 2.57 – 2.30 (m, 2H), 2.18 – 2.07 (m, 1H), 2.04 – 1.94 (m, 2H), 1.82 – 1.70 (m, 1H). **^{13}C NMR** (126 MHz, $CDCl_3$) δ 157.5, 142.9 (q, J = 2.2 Hz), 133.15, 133.13, 129.4, 128.1, 126.1 (q, J = 278.1 Hz), 122.6, 120.7 (q, J = 256.0 Hz), 115.3, 67.8, 42.4 (q, J = 2.5 Hz), 39.3 (q, J = 27.5 Hz), 30.4, 26.3. **^{19}F NMR** (471 MHz, $CDCl_3$) δ –58.39, –63.39 (t, J = 10.8 Hz). **HRMS** (ESI-TOF) *m/z*: $[M+Na]^+$ Calcd for $C_{19}H_{18}O_2SF_6Na^+$ 447.0829; Found 447.0824.

1-(4-((6,6,6-trifluoro-4-(phenylthio)hexyl)oxy)phenyl)ethan-1-one (28)

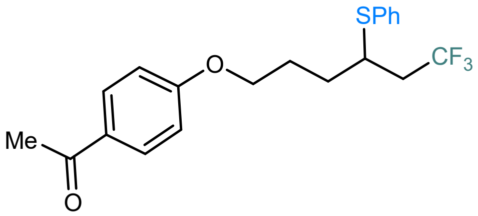

Using the General Procedure A, the title compound was obtained as yellow oil by preparative TLC using petroleum ether/EtOAc (5:1) as the eluent; R_f = 0.6 (petroleum ether/EtOAc = 5:1); 25.6 mg; 67% yield. **^{1}H NMR** (500 MHz, $CDCl_3$) δ 7.97 – 7.90 (m, 2H), 7.46 – 7.39 (m, 2H), 7.37 – 7.29 (m, 3H), 6.95 – 6.88 (m, 2H), 4.10 – 3.98 (m, 2H), 3.44 – 3.36 (m, 1H), 2.56 (s, 3H), 2.53 – 2.45 (m, 1H), 2.40 – 2.29 (m, 1H), 2.20 – 2.09 (m, 1H), 2.07 – 1.93 (m, 2H), 1.81 – 1.70 (m, 1H). **^{13}C NMR** (126 MHz, $CDCl_3$) δ 196.9, 162.9, 133.12, 133.06, 130.7, 130.5, 129.4, 128.1, 126.1 (q, J = 278.0 Hz), 114.2, 67.6, 42.3 (q, J = 2.6 Hz), 39.3 (q, J = 27.6 Hz), 30.3, 26.5, 26.1. **^{19}F NMR** (471 MHz, $CDCl_3$) δ –63.37 (t, J = 11.0 Hz). **HRMS** (ESI-TOF) *m/z*: $[M+H]^+$ Calcd for $C_{20}H_{22}O_2F_3S^+$ 383.1293; Found 383.1298.

Phenyl(4-((6,6,6-trifluoro-4-(phenylthio)hexyl)oxy)phenyl)methanone (29)

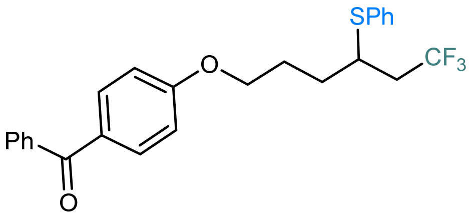

Using the General Procedure A, the title compound was obtained as yellow oil by preparative TLC using petroleum ether as the eluent; R_f = 0.4 (petroleum ether); 22.2 mg, 50% yield. **^{1}H NMR** (500 MHz, $CDCl_3$) δ 7.88 – 7.80 (m, 2H), 7.79 – 7.71 (m, 2H), 7.63 – 7.53 (m, 1H), 7.52 – 7.40 (m, 4H), 7.38 – 7.29 (m, 3H), 7.00 – 6.86 (m, 2H), 4.15 – 3.98 (m, 2H), 3.47 – 3.31 (m, 1H), 2.57 – 2.44 (m, 1H), 2.41 – 2.28 (m, 1H), 2.23 – 2.12 (m, 1H), 2.08 – 1.97 (m, 2H), 1.87 – 1.67 (m, 1H). **^{13}C NMR** (126 MHz, $CDCl_3$) δ 195.6, 162.6, 138.4, 133.10, 133.05, 132.7, 132.0, 130.2, 129.8, 129.3, 128.3, 128.1, 126.1 (q, J = 278.5 Hz), 114.1, 67.6, 42.3 (q, J = 2.4 Hz), 39.3 (q, J = 27.6 Hz), 30.3, 26.1. **^{19}F NMR** (471 MHz, $CDCl_3$) δ –63.35 (t, J = 10.8 Hz). **HRMS** (ESI-TOF) *m/z*: $[M+H]^+$ Calcd for $C_{25}H_{24}O_2F_3S^+$ 445.1449; Found 445.1454.

Phenyl(1,1,1-trifluoro-6-(4-(trifluoromethyl)phenoxy)hexan-3-yl)sulfane (30)

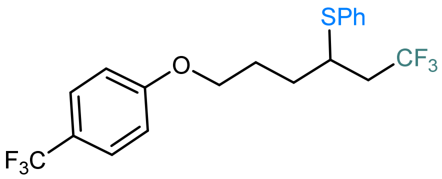

Using the General Procedure A, the title compound was obtained as yellow oil by preparative TLC using petroleum ether as the eluent; R_f = 0.7 (petroleum ether); 19.6 mg, 48% yield. **^{1}H NMR** (500 MHz, $CDCl_3$) δ 7.58 – 7.48 (m, 2H), 7.47 – 7.41 (m, 2H), 7.38 – 7.28 (m, 3H), 6.97 – 6.89 (m, 2H), 4.09 – 3.93 (m, 2H), 3.44 – 3.32 (m, 1H), 2.56 – 2.43 (m, 1H), 2.42 – 2.29 (m, 1H), 2.20 – 2.10 (m, 1H), 2.07 – 1.96 (m, 2H), 1.81 – 1.71 (m, 1H). **^{13}C NMR** (126 MHz, $CDCl_3$) δ 161.4, 133.13, 133.11, 129.4, 128.1, 127.0 (q, J = 3.8 Hz), 126.1 (q, J = 278.2 Hz), 124.6 (q, J = 270.9 Hz), 123.0 (q, J = 32.6 Hz), 114.5, 67.6, 42.3 (q, J = 2.0 Hz), 39.3 (q, J = 27.6 Hz), 30.4, 26.2. **^{19}F NMR** (471 MHz, $CDCl_3$) δ –61.43, –63.38 (t, J = 10.8 Hz). **HRMS** (ESI-TOF) *m/z*: $[M+Na]^+$ Calcd for $C_{19}H_{18}OF_6NaS^+$ 431.0880; Found 431.0879.

4-((5,5,5-trifluoro-3-(phenylthio)pentyl)oxy)benzonitrile (31)

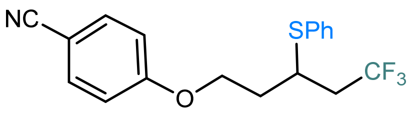

Using the General Procedure A, the title compound was obtained as yellow oil by preparative TLC using petroleum ether/EtOAc (15:1) as the eluent; R_f = 0.6 (petroleum ether/EtOAc = 15:1); 17.9 mg, 51% yield. **^{1}H NMR** (500 MHz, $CDCl_3$) δ 7.62 – 7.51 (m, 2H), 7.43 – 7.35 (m, 2H), 7.34 – 7.23 (m, 3H), 6.99 – 6.84 (m, 2H), 4.35 – 4.26 (m, 1H), 4.27 – 4.12 (m, 1H), 3.63 – 3.53 (m, 1H), 2.64 – 2.50 (m, 1H), 2.47 – 2.30 (m, 2H), 2.08 – 1.91 (m, 1H). **^{13}C NMR** (126 MHz, $CDCl_3$) δ 162.0, 134.1, 133.0, 132.8, 129.4, 128.2, 126.0 (q, J = 278.5 Hz), 119.3, 115.3, 104.3, 65.2, 39.8 (q, J = 27.6 Hz), 39.6 (q, J = 2.6 Hz), 33.4. **^{19}F NMR** (471 MHz, $CDCl_3$) δ –63.20 (t, J = 10.6 Hz). **HRMS** (ESI-TOF) *m/z*: $[M+H]^+$ Calcd for $C_{18}H_{17}NOF_3S^+$ 352.0983; Found 352.0982.

4-((5,5,5-trifluoro-3-(phenylthio)pentyl)oxy)benzaldehyde (32)

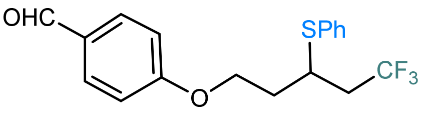

Using the General Procedure A, the title compound was obtained as yellow oil by preparative TLC using petroleum ether/EtOAc (15:1) as the eluent; R_f = 0.6 (petroleum ether/EtOAc = 15:1); 14.2 mg, 40% yield. **^{1}H NMR** (500 MHz, $CDCl_3$) δ 9.89 (s, 1H), 7.91 – 7.80 (m, 2H), 7.46 – 7.37 (m, 2H), 7.34 – 7.24 (m, 3H), 7.05 – 6.95 (m, 2H), 4.39 – 4.31 (m, 1H), 4.29 – 4.23 (m, 1H), 3.64 – 3.56 (m, 1H), 2.63 – 2.52 (m, 1H), 2.49 – 2.32 (m, 2H), 2.07 – 1.96 (m, 1H). **^{13}C NMR** (126 MHz, $CDCl_3$) δ 190.9, 163.7, 133.0, 132.8, 132.1, 130.2, 129.4, 128.2, 126.0 (q, J = 278.4 Hz), 114.9, 65.2, 39.8 (q, J = 27.8 Hz), 39.7 (q, J = 2.4 Hz), 33.5. **^{19}F NMR** (471 MHz, $CDCl_3$) δ –63.20 (t, J = 10.6 Hz). **HRMS** (ESI-TOF) *m/z*: $[M+H]^+$ Calcd for $C_{18}H_{18}O_2F_3S^+$ 355.0980; Found 355.0984.

7,7,7-trifluoro-5-(phenylthio)heptyl furan-2-carboxylate (33)

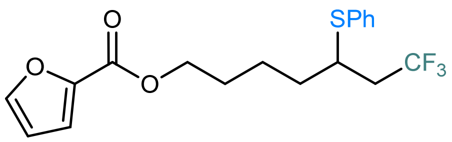

Using the General Procedure A, the title compound was obtained as yellow oil by preparative TLC using petroleum ether/EtOAc

(20:1) as the eluent; R_f= 0.7 (petroleum ether/EtOAc = 20:1); 14.8 mg, 40% yield. **^{1}H NMR** (500 MHz, $CDCl_3$) δ 7.62 – 7.53 (m, 1H), 7.44 – 7.39 (m, 2H), 7.37 – 7.27 (m, 3H), 7.20 – 7.16 (m, 1H), 6.53 – 6.48 (m, 1H), 4.36 – 4.24 (m, 2H), 3.38 – 3.27 (m, 1H), 2.50 – 2.38 (m, 1H), 2.37 – 2.25 (m, 1H), 1.82 – 1.72 (m, 4H), 1.69 – 1.57 (m, 2H). **^{13}C NMR** (126 MHz, $CDCl_3$) δ 158.9, 146.4, 144.9, 136.7, 133.0, 132.7 (q, J = 278.1 Hz), 129.3, 128.0, 118.0, 112.0, 64.7, 42.5 (q, J = 2.6 Hz), 39.3 (q, J = 27.5 Hz), 33.4, 28.5, 23.0. **^{19}F NMR** (471 MHz, $CDCl_3$) δ –63.38 (t, J = 11.1 Hz). **HRMS** (ESI-TOF) *m/z*: $[M+Na]^+$ Calcd for $C_{18}H_{19}O_3NaSF_3^+$ 395.0905; Found 395.0908.

7,7,7-trifluoro-5-(phenylthio)heptyl thiophene-2-carboxylate (34)

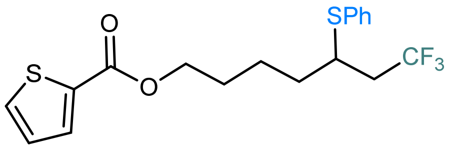

Using the General Procedure A, the title compound was obtained as yellow oil by preparative TLC using petroleum ether as the eluent; R_f= 0.4 (petroleum ether); 27.1 mg; 70% yield. **^{1}H NMR** (500 MHz, $CDCl_3$) δ 7.85 – 7.73 (m, 1H), 7.61 – 7.50 (m, 1H), 7.47 – 7.36 (m, 2H), 7.35 – 7.25 (m, 3H), 7.15 – 7.04 (m, 1H), 4.35 – 4.22 (m, 2H), 3.39 – 3.26 (m, 1H), 2.54 – 2.25 (m, 2H), 1.81 – 1.66 (m, 6H). **^{13}C NMR** (126 MHz, $CDCl_3$) δ 162.3, 134.0, 133.5, 133.3, 133.0, 132.4, 129.3, 127.85, 127.94, 126.1 (q, J = 278.5 Hz), 64.8, 42.4 (q, J = 2.5 Hz), 39.2 (q, J = 27.5 Hz), 33.4, 28.5, 23.0. **^{19}F NMR** (471 MHz, $CDCl_3$) δ –63.36 (t, J = 10.9 Hz). **HRMS** (ESI-TOF) *m/z*: $[M+Na]^+$ Calcd for $C_{18}H_{19}O_2S_2F_3Na^+$ 411.0676 Found 411.0680.

Phenyl(1,1,1-trifluoro-5-(4'-methyl-[1,1'-biphenyl]-4-yl)pentan-3-yl)sulfane (35)

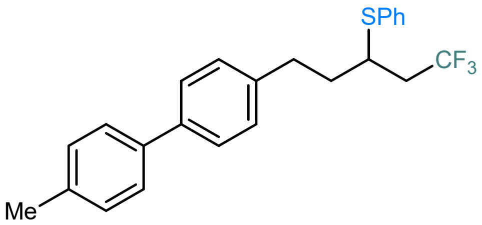

Using the General Procedure A, the title compound was obtained as a white solid by preparative TLC using petroleum ether/EtOAc (20:1) as the eluent; R_f = 0.4 (petroleum ether/EtOAc = 20:1); 20.4 mg, 51% yield. **^{1}H NMR** (500 MHz, $CDCl_3$) δ 7.54 – 7.45 (m, 4H), 7.43 – 7.38 (m, 2H), 7.34 – 7.27 (m,

3H), 7.27 – 7.19 (m, 4H), 3.40 – 3.28 (m, 1H), 3.01 – 2.91 (m, 1H), 2.90 – 2.81 (m, 1H), 2.53 – 2.44 (m, 2H), 2.39 (s, 3H), 2.22 – 2.08 (m, 1H), 2.00 – 1.77 (m, 1H). **^{13}C NMR** (126 MHz, $CDCl_3$) δ 139.8, 139.2, 138.2, 137.0, 133.3, 133.0, 129.6, 129.4, 129.0, 128.0, 127.2, 127.0, 126.1 (q, J = 278.3 Hz), 42.0 (q, J = 2.2 Hz), 39.4 (q, J = 27.5 Hz), 35.4, 32.3, 21.2. **^{19}F NMR** (471 MHz, $CDCl_3$) δ –63.20 (t, J = 11.0 Hz). **HRMS** (ESI-TOF) *m/z*: $[M+H]^+$ Calcd for $C_{24}H_{24}F_3S^+$ 401.1551 Found 401.1552. M.p.: 64–65 °C.

(4-methoxyphenyl)(1,1,1-trifluoro-5-phenylpentan-3-yl)sulfane (36)

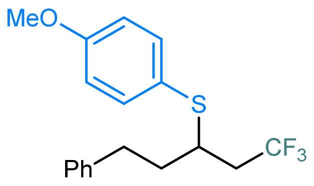

Using the General Procedure A, the title compound was obtained as yellow oil by preparative TLC using petroleum ether/EtOAc (15:1) as the eluent; R_f = 0.8 (petroleum ether/EtOAc = 15:1); 16.7 mg, 49% yield. **^{1}H NMR** (500 MHz, $CDCl_3$) δ 7.42 – 7.36 (m, 2H), 7.34 – 7.28 (m, 2H), 7.24 – 7.17 (m, 3H), 6.93 – 6.82 (m, 2H), 3.82 (s, 3H), 3.19 – 3.09 (m, 1H), 3.00 – 2.91 (m, 1H), 2.88 – 2.79 (m, 1H), 2.54 – 2.38 (m, 1H), 2.35 – 2.24 (m, 1H), 2.10 – 2.01 (m, 1H), 1.88 – 1.79 (m, 1H). **^{13}C NMR** (126 MHz, $CDCl_3$) δ 160.2, 141.2, 136.4, 128.63, 128.58, 126.23, 126.21 (q, J = 278.3 Hz), 123.0, 114.9, 55.5, 42.9 (q, J = 2.4 Hz), 39.2 (q, J = 27.5 Hz), 35.3, 32.7. **^{19}F NMR** (471 MHz, $CDCl_3$) δ –63.16 (t, J = 10.9 Hz). **HRMS** (ESI-TOF) *m/z*: $[M+H]^+$ Calcd for $C_{18}H_{20}OF_3S^+$ 341.1187; Found 341.1192.

(4-(*tert*-butyl)phenyl)(1,1,1-trifluoro-5-phenylpentan-3-yl)sulfane (37)

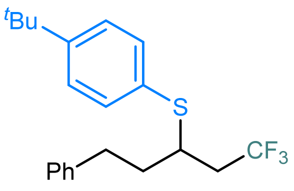

Using the General Procedure A, the title compound was obtained as yellow oil by preparative TLC using petroleum ether/EtOAc (20:1) as the eluent; R_f = 0.7 (petroleum ether/EtOAc = 20:1); 19.0 mg, 52% yield. **^{1}H NMR** (500 MHz, $CDCl_3$) δ 7.35 – 7.32 (m, 3H), 7.31 – 7.22 (m, 3H), 7.23 – 7.16 (m, 3H), 3.31 – 3.21 (m, 1H), 2.98 – 2.90 (m, 1H), 2.87 – 2.78 (m, 1H), 2.54 – 2.43 (m, 1H), 2.38 –

2.28 (m, 1H), 2.16 – 2.05 (m, 1H), 1.93 – 1.81 (m, 1H), 1.31 (s, 9H). **^{13}C NMR** (126 MHz, $CDCl_3$) δ 151.4, 141.2, 133.2, 129.5, 128.61, 128.60, 126.4, 126.24, 126.19 (q, J = 278.5 Hz), 42.0 (q, J = 2.4 Hz), 39.4 (q, J = 27.5 Hz), 35.4, 34.8, 32.7, 31.4. **^{19}F NMR** (471 MHz, $CDCl_3$) δ –63.16 (t, J = 10.7 Hz). **HRMS** (ESI-TOF) *m/z*: $[M+H]^+$ Calcd for $C_{21}H_{26}F_3S^+$ 367.1707 Found 367.1710.

(4-fluorophenyl)(1,1,1-trifluoro-5-phenylpentan-3-yl)sulfane (38)

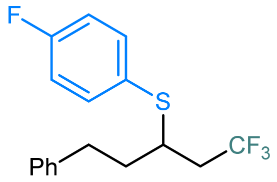

Using the General Procedure A, the title compound was obtained as yellow oil by preparative TLC using petroleum ether/EtOAc (15:1) as the eluent; R_f = 0.7 (petroleum ether/EtOAc = 15:1); 29.5 mg, 90% yield. **^{1}H NMR** (500 MHz, $CDCl_3$) δ 7.45 – 7.34 (m, 2H), 7.34 – 7.25 (m, 2H), 7.24 – 7.13 (m, 3H), 7.05 – 6.98 (m, 2H), 3.19 (m, 1H), 2.97 – 2.75 (m, 2H), 2.52 – 2.25 (m, 2H), 2.13 – 1.76 (m, 2H). **^{13}C NMR** (126 MHz, $CDCl_3$) δ 162.9 (d, J = 248.7 Hz), 140.9, 135.9 (d, J = 8.3 Hz), 128.7, 128.5, 128.1 (d, J = 3.5 Hz), 126.3, 126.1 (q, J = 278.1 Hz), 116.5 (d, J = 21.9 Hz), 42.7 (q, J = 3.0 Hz), 39.2 (q, J = 27.7 Hz), 35.4, 32.6. **^{19}F NMR** (471 MHz, $CDCl_3$) δ –63.24 (t, J = 11.0 Hz), –112.82. **HRMS** (ESI-TOF) *m/z*: $[M+H]^+$ Calcd for $C_{17}H_{17}F_4S^+$ 329.0987; Found 329.0978.

(4-bromophenyl)(1,1,1-trifluoro-5-phenylpentan-3-yl)sulfane (39)

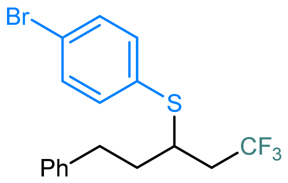

Using the General Procedure A, the title compound was obtained as yellow oil by preparative TLC using petroleum ether/EtOAc (20:1) as the eluent; R_f =0.8 (petroleum ether/EtOAc = 20:1); 20.2 mg, 52% yield. **^{1}H NMR** (500 MHz, $CDCl_3$) δ 7.48 – 7.39 (m, 2H), 7.34 – 7.26 (m, 2H), 7.26 – 7.12 (m, 5H), 3.34 – 3.20 (m, 1H), 2.96 – 2.86 (m, 1H), 2.86 – 2.77 (m, 1H), 2.49 – 2.27 (m, 2H), 2.18 – 2.05 (m, 1H), 1.94 – 1.83 (m, 1H). **^{13}C NMR** (126 MHz, $CDCl_3$) δ 140.8, 134.3, 132.6, 132.5, 128.7, 128.5, 126.4, 126.0 (q, J = 278.1 Hz), 122.2, 42.0 (q, J = 2.5 Hz), 39.3 (q, J = 27.6 Hz), 35.4, 32.6. **^{19}F NMR** (471 MHz, $CDCl_3$) δ –63.29 (t, J = 11.0 Hz). **HRMS**

(ESI-TOF) *m/z*: $[M+K]^+$ Calcd for $C_{17}H_{16}F_3SBrK^+$ 426.9745; Found 426.9743.

(4-chlorophenyl)(1,1,1-trifluoro-5-phenylpentan-3-yl)sulfane (40)

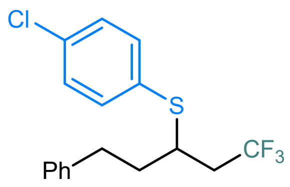

Using the General Procedure A, the title compound was obtained as yellow oil by preparative TLC using petroleum ether/EtOAc (20:1) as the eluent; R_f = 0.8 (petroleum ether/EtOAc = 20:1); 32.6 mg, 95% yield. **^{1}H NMR** (500 MHz, $CDCl_3$) δ 7.33 – 7.26 (m, 6H), 7.24 – 7.14 (m, 3H), 3.31 – 3.22 (m, 1H), 2.95 – 2.86 (m, 1H), 2.85 – 2.76 (m, 1H), 2.50 – 2.27 (m, 2H), 2.15 – 2.05 (m, 1H), 1.94 – 1.76 (m, 1H). **^{13}C NMR** (126 MHz, $CDCl_3$) δ 140.8, 134.2, 131.9, 129.5, 128.7, 128.6, 126.4, 126.0 (q, J = 278.5 Hz), 42.2 (q, J = 2.5 Hz), 39.3 (q, J = 27.7 Hz), 35.4, 32.6. **^{19}F NMR** (471 MHz, $CDCl_3$) δ –63.30 (t, J = 10.8 Hz). **HRMS** (ESI-TOF) *m/z*: $[M+Na]^+$ Calcd for $C_{17}H_{16}F_3SClNa^+$ 367.0511; Found 367.0514.

Methyl 4-((1,1,1-trifluoro-5-phenylpentan-3-yl)thio)benzoate (41)

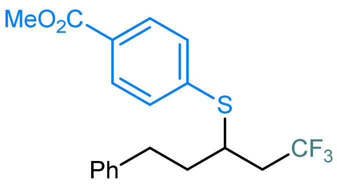

Using the General Procedure A, the title compound was obtained as yellow oil by preparative TLC using petroleum ether/EtOAc (20:1) as the eluent; R_f = 0.5 (petroleum ether/EtOAc = 20:1); 20.2 mg, 55% yield. **^{1}H NMR** (500 MHz, $CDCl_3$) δ 7.99 – 7.88 (m, 2H), 7.35 – 7.14 (m, 7H), 3.91 (s, 3H), 3.51 – 3.43 (m, 1H), 2.94 – 2.86 (m, 1H), 2.86 – 2.76 (m, 1H), 2.52 – 2.37 (m, 2H), 2.22 – 2.13 (m, 1H), 1.97 – 1.88 (m, 1H). **^{13}C NMR** (126 MHz, $CDCl_3$) δ 166.7, 140.8, 140.6, 130.4, 129.8, 128.7, 128.6, 128.6, 126.4, 125.9 (q, J = 278.1 Hz), 52.3, 40.6 (q, J = 2.4 Hz), 39.3 (q, J = 27.7 Hz), 35.6, 32.7. **^{19}F NMR** (471 MHz, $CDCl_3$) δ –63.40 (t, J = 10.7 Hz). **HRMS** (ESI-TOF) *m/z*: $[M+H]^+$ Calcd for $C_{19}H_{20}O_2F_3S^+$ 369.1136 Found 369.1138.

(4-nitrophenyl)(1,1,1-trifluoro-5-phenylpentan-3-yl)sulfane (42)

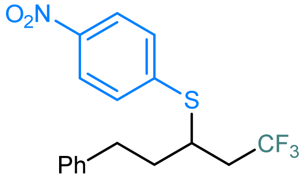

Using the General Procedure A, the title compound was obtained as yellow oil by preparative TLC using petroleum ether/EtOAc (20:1) as the eluent; R_f = 0.5 (petroleum ether/EtOAc = 20:1); 14.2 mg, 40% yield. **^{1}H NMR** (500 MHz, CDCl3) *δ* 8.20 – 8.03 (m, 2H), 7.35 – 7.28 (m, 4H), 7.27 – 7.23 (m, 1H), 7.20 – 7.12 (m, 2H), 3.61 – 3.40 (m, 1H), 2.97 – 2.73 (m, 2H), 2.63 – 2.30 (m, 2H), 2.30 – 2.16 (m, 1H), 2.04 – 1.89 (m, 1H). **^{13}C NMR** (126 MHz, $CDCl_3$) *δ* 146.1, 144.6, 140.2, 128.83, 128.81, 128.6, 126.6, 125.8 (q, *J* = 278.0 Hz), 124.3, 40.1 (q, *J* = 2.5 Hz), 39.3 (q, *J* = 28.0 Hz), 35.7, 32.6. **^{19}F NMR** (471 MHz, $CDCl_3$) *δ* –63.44 (t, *J* = 10.7 Hz). **HRMS** (ESI-TOF) *m/z*: $[M+H]^+$ Calcd for $C_{17}H_{17}NO_2F_3S^+$ 356.0932; Found 356.0938.

(3-fluorophenyl)(1,1,1-trifluoro-5-phenylpentan-3-yl)sulfane (43)

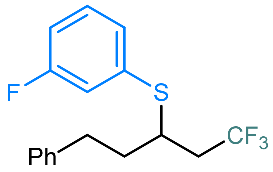

Using the General Procedure A, the title compound was obtained as yellow oil by preparative TLC using petroleum ether as the eluent; R_f=0.5 (petroleum ether); 18.6 mg, 57% yield; **^{1}H NMR** (500 MHz, $CDCl_3$) δ 7.25 – 7.06 (m, 7H), 7.04 – 6.99 (m, 1H), 6.95 – 6.88 (m, 1H), 3.34 – 3.23 (m, 1H), 2.90 – 2.72 (m, 2H), 2.45 – 2.29 (m, 2H), 2.13 – 2.04 (m, 1H), 1.89 – 1.79 (m, 1H). **^{13}C NMR** (126 MHz, $CDCl_3$) δ 162.9 (d, *J* = 249.4 Hz), 140.8, 136.0 (d, *J* = 7.7 Hz), 130.6 (d, *J* = 8.6 Hz), 128.7, 128.6, 127.7 (d, *J* = 3.1 Hz), 126.4, 126.0 (q, *J* = 278.5 Hz), 118.9 (d, *J* = 22.4 Hz), 114.8 (d, *J* = 21.1 Hz), 41.7 (q, *J* = 2.4 Hz), 39.3 (q, *J* = 27.7 Hz), 35.5, 32.6. **^{19}F NMR** (471 MHz, $CDCl_3$) δ –63.32 (t, *J* = 10.8 Hz), –111.58 – –111.68 (m). **HRMS** (ESI-TOF) *m/z*: $[M+H]^+$ Calcd for $C_{17}H_{17}F_4S^+$ 329.0987; Found 329.0980.

(1,1,1-trifluoro-5-phenylpentan-3-yl)(3-(trifluoromethyl)phenyl)sulfane (44)

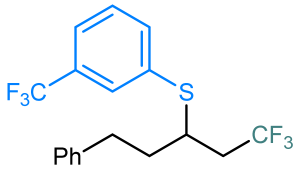

Using the General Procedure A, the title compound was obtained as yellow oil by preparative TLC using petroleum ether as the eluent; R_f= 0.5 (petroleum ether); 17.8 mg, 47% yield. **^{1}H NMR** (500 MHz, $CDCl_3$) *δ* 7.57 (s, 1H), 7.51 –

7.43 (m, 2H), 7.41 – 7.35 (m, 1H), 7.26 – 7.09 (m, 5H), 3.37 – 3.28 (m, 1H), 2.90 – 2.83 (m, 1H), 2.82 – 2.73 (m, 1H), 2.45 – 2.31 (m, 2H), 2.13 – 2.04 (m, 1H), 1.91 – 1.81 (m, 1H). **^{13}C NMR** (126 MHz, $CDCl_3$) δ 140.6, 135.3, 135.2, 131.8 (q, J = 32.5 Hz), 129.8, 128.7, 128.7 (q, J = 4.0 Hz), 128.5, 126.5, 125.9 (q, J = 278.6 Hz), 124.4 (q, J = 3.8 Hz), 123.8 (q, J = 272.7 Hz), 41.8 (q, J = 2.6 Hz), 39.4 (q, J = 27.8 Hz), 35.5, 32.6. **^{19}F NMR** (471 MHz, $CDCl_3$) δ –62.84, –63.40 (t, J = 10.7 Hz). **HRMS** (ESI-TOF) *m/z*: $[M+H]^+$ Calcd for $C_{18}H_{17}F_6S^+$ 379.0955; Found 379.0947.

(3-nitrophenyl)(1,1,1-trifluoro-5-phenylpentan-3-yl)sulfane (45)

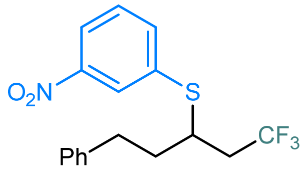

Using the General Procedure A, the title compound was obtained as yellow oil by preparative TLC using petroleum ether/EtOAc (15:1) as the eluent; R_f = 0.7 (petroleum ether/EtOAc = 15:1); 16.0 mg, 45% yield. **^{1}H NMR** (500 MHz, $CDCl_3$) δ 8.24 – 8.17 (m, 1H), 8.15 – 8.06 (m, 1H), 7.64 – 7.57 (m, 1H), 7.54 – 7.42 (m, 1H), 7.35 – 7.27 (m, 2H), 7.27 – 7.13 (m, 3H), 3.51 – 3.34 (m, 1H), 2.98 – 2.88 (m, 1H), 2.87 – 2.78 (m, 1H), 2.51 – 2.38 (m, 2H), 2.26 – 2.08 (m, 1H), 2.02 – 1.84 (m, 1H). **^{13}C NMR** (126 MHz, $CDCl_3$) δ 148.7, 140.4, 137.1, 136.9, 130.1, 128.8, 128.5, 126.5, 126.0, 125.8 (q, J = 278.4 Hz), 122.3, 41.9 (q, J = 2.5 Hz), 39.4 (q, J = 28.0 Hz), 35.6, 32.6. **^{19}F NMR** (471 MHz, $CDCl_3$) δ –63.33 (t, J = 10.5 Hz). **HRMS** (ESI-TOF) *m/z*: $[M+Na]^+$ Calcd for $C_{17}H_{16}NO_2F_3NaS^+$ 378.0752; Found 378.0748.

(2-methoxyphenyl)(1,1,1-trifluoro-5-phenylpentan-3-yl)sulfane (46)

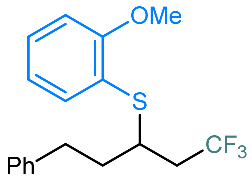

Using the General Procedure A, the title compound was obtained as yellow oil by preparative TLC using petroleum ether/EtOAc (15:1) as the eluent; R_f= 0.6 (petroleum ether/EtOAc = 15:1); 30.9 mg, 91% yield. **^{1}H NMR** (500 MHz, CDCl3) δ 7.41 – 7.36 (m, 1H), 7.33 – 7.27 (m, 3H), 7.23 – 7.17 (m, 3H), 6.97 – 6.88 (m, 2H), 3.86 (s, 3H), 3.57 – 3.52 (m, 1H), 2.99 – 2.79 (m, 2H), 2.53 – 2.30 (m, 2H), 2.21 – 1.84 (m, 2H). **^{13}C NMR** (126 MHz, $CDCl_3$) δ 159.2, 141.3, 134.2, 129.6, 128.6, 128.5, 127.4 (q, J = 278.1

Hz), 126.1, 121.3, 121.2, 111.2, 55.8, 39.8 (q, J = 2.1 Hz), 39.2 (q, J = 27.3 Hz), 35.7, 32.6. **^{19}F NMR** (471 MHz, $CDCl_3$) δ –63.45 (t, J = 11.0 Hz). **HRMS** (ESI-TOF) *m/z*: $[M+H]^+$ Calcd for $C_{18}H_{20}OF_3S^+$ 341.1187; Found 341.1186.

_o_-tolyl(1,1,1-trifluoro-5-phenylpentan-3-yl)sulfane (47)

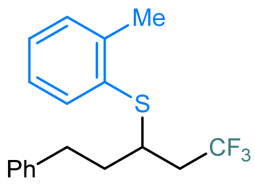

Using the General Procedure A, the title compound was obtained as yellow oil by preparative TLC using petroleum ether/EtOAc (20:1) as the eluent; R_f = 0.8 (petroleum ether/EtOAc = 20:1); 12.6 mg; 39% yield. **^{1}H NMR** (500 MHz, $CDCl_3$) δ 7.33 – 7.27 (m, 3H), 7.24 – 7.14 (m, 6H), 3.42 – 3.34 (m, 1H), 2.97 – 2.87 (m, 1H), 2.87 – 2.79 (m, 1H), 2.42 (s, 3H), 2.40 – 2.38 (m, 2H), 2.21 – 2.14 (m, 1H), 2.00 – 1.89 (m, 1H). **^{13}C NMR** (126 MHz, $CDCl_3$) δ 141.1, 140.2, 133.1, 132.2, 130.8, 128.6, 128.6, 127.7, 126.8, 126.3, 126.2 (q, J = 278.3 Hz), 41.0 (q, J = 2.5 Hz), 39.0 (q, J = 27.4 Hz), 35.6, 32.6, 20.9. **^{19}F NMR** (471 MHz, $CDCl_3$) δ –63.35 (t, J = 10.8 Hz). **HRMS** (ESI-TOF) *m/z*: $[M+H]^+$ Calcd for $C_{18}H_{20}F_3S^+$ 325.1238; Found 325.1241.

(2-fluorophenyl)(1,1,1-trifluoro-5-phenylpentan-3-yl)sulfane (48)

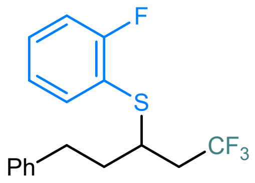

Using the General Procedure A, the title compound was obtained as yellow oil by preparative TLC using petroleum ether/EtOAc (20:1) as the eluent; R_f = 0.7 (petroleum ether/EtOAc = 20:1); 26.9 mg, 82% yield. **^{1}H NMR** (500 MHz, $CDCl_3$) δ 7.48 – 7.39 (m, 1H), 7.35 – 7.25 (m, 3H), 7.23 – 7.15 (m, 3H), 7.14 – 7.07 (m, 2H), 3.43 – 3.33 (m, 1H), 2.99 – 2.88 (m, 1H), 2.86 – 2.78 (m, 1H), 2.51 – 2.32 (m, 2H), 2.14 – 2.01 (m, 1H), 1.94 – 1.81 (m, 1H). **^{13}C NMR** (126 MHz, $CDCl_3$) δ 162.9 (d, J = 246.7 Hz), 141.1, 135.8, 130.6 (d, J = 8.1 Hz), 128.6 (d, J = 7.0 Hz), 126.3, 126.1 (q, J = 278.3 Hz), 124.9 (d, J = 3.9 Hz), 120.1 (d, J = 18.3 Hz), 116.3 (d, J = 23.2 Hz), 41.7 – 41.6 (m), 39.5 (q, J = 27.6 Hz), 35.8, 32.6. **^{19}F NMR** (471 MHz, $CDCl_3$) δ –63.37 (t, J = 10.9 Hz), –106.66 – –106.73 (m). **HRMS** (ESI-TOF) *m/z*: $[M+Na]^+$ Calcd for $C_{17}H_{16}F_4NaS^+$ 351.0807; Found 351.0808.

(2-chlorophenyl)(1,1,1-trifluoro-5-phenylpentan-3-yl)sulfane (49)

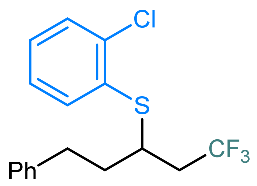

Using the General Procedure A, the title compound was obtained as yellow oil by preparative TLC using petroleum ether as the eluent; R_f = 0.7 (petroleum ether); 25.8 mg, 75% yield. **^{1}H NMR** (500 MHz, $CDCl_3$) δ 7.46 – 7.42 (m, 1H), 7.38 – 7.33 (m, 1H), 7.32 – 7.27 (m, 2H), 7.24 – 7.15 (m, 5H), 3.59 – 3.46 (m, 1H), 3.00 – 2.89 (m, 1H), 2.88 – 2.81 (m, 1H), 2.52 – 2.40 (m, 2H), 2.23 – 2.14 (m, 1H), 2.02 – 1.92 (m, 1H). **^{13}C NMR** (126 MHz, $CDCl_3$) δ 140.9, 136.6, 133.2, 132.7, 130.4, 128.7, 128.6, 127.5, 126.3, 126.0 (q, J = 275.5 Hz), 40.8 (q, J = 2.5 Hz), 39.1 (q, J = 27.7 Hz), 35.5, 32.6. **^{19}F NMR** (471 MHz, $CDCl_3$) δ –63.38 (t, J = 10.8 Hz). **HRMS** (ESI-TOF) *m/z*: $[M+Na]^+$ Calcd for $C_{17}H_{16}F_3SClNa^+$ 367.0511; Found 367.0507.

Methyl 2-((1,1,1-trifluoro-5-phenylpentan-3-yl)thio)benzoate (50)

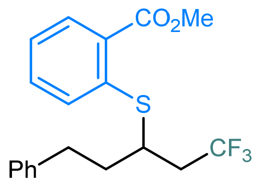

Using the General Procedure A, the title compound was obtained as yellow oil by preparative TLC using petroleum ether/EtOAc (10:1) as the eluent; R_f = 0.8 (petroleum ether/EtOAc = 10:1); 22.4 mg, 61% yield. **^{1}H NMR** (500 MHz, $CDCl_3$) δ 7.98 – 7.87 (m, 1H), 7.48 – 7.37 (m, 1H), 7.34 – 7.27 (m, 2H), 7.26 – 7.16 (m, 5H), 3.94 (s, 3H), 3.61 – 3.50 (m, 1H), 2.97 – 2.89 (m, 1H), 2.87 – 2.76 (m, 1H), 2.55 – 2.38 (m, 2H), 2.30 – 2.19 (m, 1H), 2.07 – 1.91 (m, 1H). **^{13}C NMR** (126 MHz, $CDCl_3$) δ 167.1, 140.9, 138.4, 132.6, 131.4, 130.2, 128.64, 128.61, 127.7, 126.3, 126.1 (q, J = 278.3 Hz), 125.3, 52.4, 39.0 (q, J = 27.6 Hz), 38.9 (q, J = 2.4 Hz), 35.3, 32.8. **^{19}F NMR** (471 MHz, $CDCl_3$) δ –63.47 (t, J = 10.8 Hz). **HRMS** (ESI-TOF) *m/z*: $[M+Na]^+$ Calcd for $C_{19}H_{19}O_2F_3NaS^+$ 391.0956; Found 391.0960.

***N*-(2-((1,1,1-trifluoro-5-phenylpentan-3-yl)thio)phenyl)benzamide (51)**

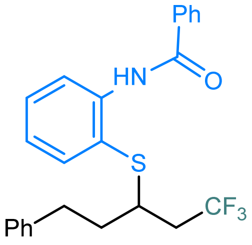

Using the General Procedure A, the title compound was obtained as yellow oil by preparative TLC using petroleum ether/EtOAc (15:1) as the eluent; R_f = 0.5 (petroleum ether/EtOAc = 15:1); 15.9 mg, 37% yield. **^{1}H NMR** (500 MHz, $CDCl_3$) δ 9.29 (s, 1H), 8.62 – 8.54 (m, 1H), 7.93 – 7.78 (m, 2H), 7.59 – 7.33 (m, 5H), 7.19 – 7.08 (m, 3H), 7.06 – 6.94 (m, 3H), 3.16 – 3.04 (m, 1H), 2.78 – 2.61 (m, 2H), 2.35 – 2.23 (m, 2H), 2.02 – 1.91 (m, 1H), 1.90 – 1.80 (m, 1H). **^{13}C NMR** (126 MHz, $CDCl_3$) δ 165.4, 140.7, 140.4, 136.6, 134.8, 132.3, 131.1, 129.1, 128.7, 128.3, 127.2, 126.4, 125.9 (q, J = 278.4 Hz), 124.4, 120.6, 120.3, 43.4 (q, J = 2.0 Hz), 38.5 (q, J = 28.0 Hz), 35.6, 32.5. **^{19}F NMR** (471 MHz, $CDCl_3$) δ –63.06 (t, J = 10.8 Hz). **HRMS** (ESI-TOF) *m/z*: $[M+Na]^+$ Calcd for $C_{24}H_{22}NOF_3SNa^+$ 452.1272; Found 452.1275.

Naphthalen-2-yl(1,1,1-trifluoro-5-phenylpentan-3-yl)sulfane (52)

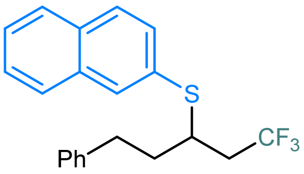

Using the General Procedure A, the title compound was obtained as yellow oil by preparative TLC using petroleum ether as the eluent; R_f = 0.6 (petroleum ether); 14.4 mg, 40% yield. **^{1}H NMR** (500 MHz, $CDCl_3$) δ 7.90 – 7.74 (m, 4H), 7.55 – 7.42 (m, 3H), 7.32 – 7.28 (m, 2H), 7.24 – 7.16 (m, 3H), 3.51 – 3.38 (m, 1H), 3.03 – 2.93 (m, 1H), 2.94 – 2.84 (m, 1H), 2.59 – 2.48 (m, 1H), 2.46 – 2.36 (m, 1H), 2.25 – 2.10 (m, 1H), 2.02 – 1.90 (m, 1H). **^{13}C NMR** (126 MHz, $CDCl_3$) δ 141.0, 133.8, 132.7, 131.7, 130.8, 129.8, 129.0, 128.7, 128.6, 127.9, 127.6, 126.9, 126.6, 126.3, 126.1 (q, J = 278.4 Hz), 41.8 (q, J = 2.4 Hz), 39.3 (q, J = 27.5 Hz), 35.5, 32.7. **^{19}F NMR** (471 MHz, $CDCl_3$) δ –63.23 (t, J = 10.7 Hz). **HRMS** (ESI-TOF) *m/z*: $[M+H]^+$ Calcd for $C_{21}H_{20}F_3S^+$ 361.1238; Found 361.1236.

2-((1,1,1-trifluoro-5-phenylpentan-3-yl)thio)thiophene (53)

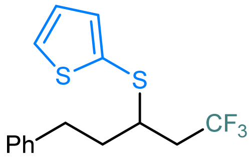

Using the General Procedure A, the title compound was obtained as yellow oil by preparative TLC using petroleum ether as the eluent; R_f = 0.6 (petroleum ether); 16.4 mg, 52% yield. **^{1}H**

NMR (500 MHz, $CDCl_3$) δ 7.47 – 7.41 (m, 1H), 7.32 – 7.27 (m, 2H), 7.24 – 7.18 (m, 4H), 7.10 – 6.98 (m, 1H), 3.08 – 2.95 (m, 2H), 2.87 – 2.76 (m, 1H), 2.61 – 2.46 (m, 1H), 2.37 – 2.21 (m, 1H), 2.08 – 1.95 (m, 1H), 1.90 – 1.76 (m, 1H). **^{13}C NMR** (126 MHz, $CDCl_3$) δ 141.0, 136.7, 131.3, 129.8, 128.7, 128.6, 128.0, 126.3, 126.1 (q, J = 278.5 Hz), 43.9 (q, J = 2.3 Hz), 39.3 (q, J = 27.6 Hz), 35.1, 32.8. **^{19}F NMR** (471 MHz, $CDCl_3$) δ –63.06 (t, J = 10.7 Hz). **HRMS** (ESI-TOF) *m/z*: $[M+H]^+$ Calcd for $C_{15}H_{16}F_3S_2^+$ 317.0646; Found 317.0643.

2-((1,1,1-trifluoro-5-phenylpentan-3-yl)thio)pyridine (54)

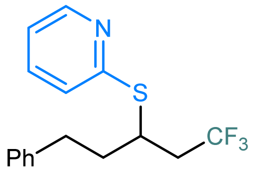

Using the General Procedure A, the title compound was obtained as yellow oil by preparative TLC using petroleum ether/EtOAc (20:1) as the eluent; R_f = 0.5 (petroleum ether/EtOAc = 20:1); 23.9 mg, 77% yield. **^{1}H NMR** (500 MHz, $CDCl_3$) δ 8.44 – 8.40 (m, 1H), 7.53 – 7.44 (m, 1H), 7.31 – 7.23 (m, 2H), 7.22 – 7.12 (m, 4H), 7.05 – 6.93 (m, 1H), 4.28 – 4.11 (m, 1H), 2.95 – 2.84 (m, 1H), 2.81 – 2.68 (m, 2H), 2.62 – 2.48 (m, 1H), 2.27 – 2.14 (m, 1H), 2.12 – 2.00 (m, 1H). **^{13}C NMR** (126 MHz, $CDCl_3$) δ 157.8, 149.7, 141.4, 136.3, 128.6, 126.3 (q, J = 278.5 Hz), 126.2, 122.9, 119.9, 39.3 (q, J = 27.4 Hz), 38.2 (q, J = 2.2 Hz), 35.5, 33.1. **^{19}F NMR** (471 MHz, $CDCl_3$) δ –62.89 (t, J = 10.9 Hz). **HRMS** (ESI-TOF) *m/z*: $[M+H]^+$ Calcd for $C_{16}H_{17}NF_3S^+$ 312.1034; Found 312.1037.

(1,1-difluoro-5-phenylpentan-3-yl)(phenyl)sulfane (55)

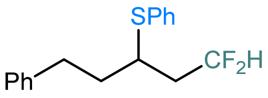

Using the General Procedure A, the title compound was obtained as yellow oil by preparative TLC using petroleum ether as the eluent; R_f = 0.6 (petroleum ether); 12.3 mg, 42% yield. **^{1}H NMR** (500 MHz, $CDCl_3$) δ 7.43 – 7.37 (m, 2H), 7.35 – 7.26 (m, 5H), 7.24 – 7.14 (m, 3H), 6.22 – 5.97 (m, 1H), 3.24 – 3.15 (m, 1H), 2.91 – 2.78 (m, 2H), 2.19 – 2.06 (m, 2H), 1.98 – 1.90 (m, 2H). **^{13}C NMR** (126 MHz, $CDCl_3$) δ 141.2, 133.5, 132.9, 129.3, 128.63, 128.58, 127.8, 126.2, 116.3 (t, J = 239.3 Hz), 42.9 (dd, J = 7.1, 4.3 Hz),

39.4 (t, J = 21.6 Hz), 36.8, 32.9. **^{19}F NMR** (471 MHz, $CDCl_3$) δ –115.29 (dddd, J = 286.4, 57.5, 15.9, 12.2 Hz), –116.87 (dddd, J = 285.4, 57.2, 24.3, 14.4 Hz). **HRMS** (ESI-TOF) *m/z*: $[M+Na]^+$ Calcd for $C_{17}H_{18}F_2SNa^+$ 315.0995; Found 315.0997.

(5,5-difluoro-1-phenylhexan-3-yl)(phenyl)sulfane (56)

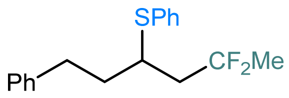

Using the General Procedure A, the title compound was obtained as yellow oil by preparative TLC using petroleum ether/EtOAc (20:1) as the eluent; R_f = 0.8 (petroleum ether/EtOAc = 20:1); 10.4 mg, 34% yield. **^{1}H NMR** (500 MHz, $CDCl_3$) δ 7.42 – 7.32 (m, 2H), 7.32 – 7.23 (m, 5H), 7.21 – 7.11 (m, 3H), 3.41 – 3.28 (m, 1H), 2.94 – 2.76 (m, 2H), 2.29 – 2.14 (m, 2H), 2.13 – 2.06 (m, 1H), 1.96 – 1.81 (m, 1H), 1.55 (t, J = 18.5 Hz, 3H). **^{13}C NMR** (126 MHz, $CDCl_3$) δ 141.5, 134.4, 132.3, 129.2, 128.6, 128.5, 127.4, 126.1, 123.7 (t, J = 239.4 Hz), 43.1 (t, J = 24.8 Hz), 42.4 (t, J = 3.5 Hz), 36.3, 32.7, 24.1 (t, J = 27.6 Hz). **^{19}F NMR** (471 MHz, $CDCl_3$) δ –86.29 (m, 1F), –90.32 (m, 1F). **HRMS** (ESI-TOF) *m/z*: $[M+H]^+$ Calcd for $C_{18}H_{21}F_2S^+$ 307.1332; Found 307.1335.

(1,1-difluoro-1,5-diphenylpentan-3-yl)(phenyl)sulfane (57)

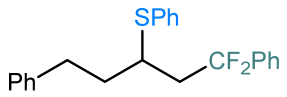

Using the General Procedure A, the title compound was obtained as yellow oil by preparative TLC using petroleum ether as the eluent; R_f= 0.5 (petroleum ether); 18.8 mg, 51% yield. **^{1}H NMR** (500 MHz, $CDCl_3$) δ 7.40 – 7.34 (m, 2H), 7.33 – 7.28 (m, 2H), 7.25 – 7.15 (m, 9H), 7.14 – 7.07 (m, 2H), 3.22 – 3.14 (m, 1H), 2.89 – 2.72 (m, 2H), 2.51 – 2.36 (m, 2H), 2.17 – 2.06 (m, 1H), 1.89 – 1.79 (m, 1H). **^{13}C NMR** (126 MHz, $CDCl_3$) δ 141.5, 136.8 (t, J = 26.4 Hz), 134.3, 132.0, 129.9 (t, J = 1.7 Hz), 129.1, 128.7, 128.6, 128.5, 127.2, 126.1, 125.0 (t, J = 6.2 Hz), 122.6 (t, J = 244.4 Hz), 44.5 (t, J = 26.9 Hz), 42.8 – 40.7 (m), 36.0, 32.8. **^{19}F NMR** (471 MHz, $CDCl_3$) δ –89.93 (ddd, J = 245.6, 17.7, 11.2 Hz), –96.55 (ddd, J = 245.6, 19.0, 16.7 Hz). **HRMS** (ESI-TOF) *m/z*: $[M+Na]^+$ Calcd for $C_{23}H_{22}F_2SNa^+$ 391.1308; Found 391.1310.

Phenyl(6,6,6-trifluoro-1-phenylhexan-3-yl)sulfane (58)

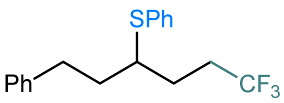

Using the General Procedure A, the title compound was obtained as yellow oil by preparative TLC using petroleum ether/EtOAc (20:1) as the eluent; R_f = 0.8 (petroleum ether/EtOAc = 20:1); 18.7 mg, 54% yield. **^{1}H NMR** (500 MHz, CDCl3) δ 7.40 – 7.36 (m, 2H), 7.33 – 7.27 (m, 5H), 7.23 – 7.20 (m, 1H), 7.19 – 7.16 (m, 2H), 3.07 – 2.99 (m, 1H), 2.92 – 2.77 (m, 2H), 2.43 – 2.21 (m, 2H), 1.93 – 1.83 (m, 3H), 1.81 – 1.72 (m, 1H). **^{13}C NMR** (126 MHz, CDCl3) δ 141.3, 133.8, 133.0, 129.2, 128.64, 128.58, 128.5 (q, J = 278.1 Hz), 127.6, 126.2, 47.6, 36.3, 33.1, 31.1 (q, J = 28.8 Hz), 26.9 (q, J = 2.7 Hz). **^{19}F NMR** (471 MHz, CDCl3) δ –66.07 (t, J = 10.9 Hz). **HRMS** (ESI-TOF) *m/z*: $[M+Na]^+$ Calcd for $C_{18}H_{19}F_3NaS^+$ 347.1057; Found 347.1054.

Phenyl(6,6,6-trifluoro-5,5-dimethyl-1-phenylhexan-3-yl)sulfane (59)

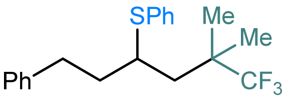

Using the General Procedure A, the title compound was obtained as a yellow viscous oil by preparative TLC using petroleum ether/EtOAc (20:1) as an eluent; R_f= 0.8 (petroleum ether/EtOAc = 20:1); 17.6 mg, 50% yield. **^{1}H NMR** (500 MHz, $CDCl_3$) δ 7.39 – 7.35 (m, 2H), 7.30 – 7.22 (m, 5H), 7.20 – 7.16 (m, 1H), 7.13 – 7.10 (m, 2H), 3.14 – 3.07 (m, 1H), 2.87 – 2.71 (m, 2H), 1.96 – 1.79 (m, 4H), 1.14 – 1.07 (m, 6H). **^{13}C NMR** (126 MHz, $CDCl_3$) δ 141.5, 134.9, 132.7, 129.5 (q, J = 282.7 Hz), 129.1, 128.6, 128.5, 127.4, 126.1, 43.8, 40.8 (q, J = 24.0 Hz), 40.2, 38.6, 32.9, 21.7 (q, J = 2.2 Hz), 20.5 (q, J = 2.0 Hz). **^{19}F NMR** (471 MHz, $CDCl_3$) δ –78.57. **HRMS** (ESI-TOF) *m/z*: $[M+H]^+$ Calcd for $C_{20}H_{24}F_3S^+$ 353.1551; Found 353.1553.

Phenyl(4-phenyl-1-(1-(trifluoromethyl)cyclobutyl)butan-2-yl)sulfane (60)

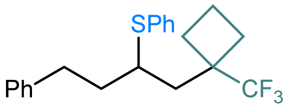

Using the General Procedure A, the title compound was obtained as a yellow viscous oil by preparative TLC using petroleum ether/EtOAc (20:1) as an eluent; R_f = 0.8 (petroleum ether/EtOAc = 20:1); 17.9 mg, 49% yield. **^{1}H NMR** (500 MHz, $CDCl_3$) δ 7.44 – 7.39 (m, 2H), 7.35 – 7.27 (m, 5H), 7.24 – 7.19 (m, 1H), 7.19 – 7.15 (m, 2H),

3.30 – 3.24 (m, 1H), 2.92 – 2.77 (m, 2H), 2.39 – 2.25 (m, 2H), 2.13 – 1.93 (m, 6H), 1.92 – 1.80 (m, 2H). **^{13}C NMR** (126 MHz, $CDCl_3$) δ 141.6, 134.9, 132.3, 129.2 (q, J = 280.4 Hz), 129.1, 128.6, 128.5, 127.2, 126.1, 45.3, 44.9 (q, J = 26.0 Hz), 40.3 (q, J = 2.3 Hz), 37.3, 33.1, 27.6 (q, J = 3.2 Hz), 26.7 (q, J = 3.1 Hz), 15.4. **^{19}F NMR** (471 MHz, $CDCl_3$) δ –76.36. **HRMS** (ESI-TOF) *m/z*: $[M+H]^+$ Calcd for $C_{21}H_{24}F_3S^+$ 365.1551; Found 365.1556.

(*E*)-(1-cyclohexyl-3,3,3-trifluoroprop-1-en-1-yl)(phenyl)sulfane (61)

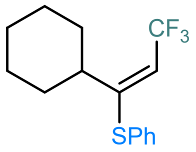

Using the General Procedure A, the title compound was obtained as colorless oil by preparative TLC using petroleum ether as the eluent; R_f = 0.8 (petroleum ether); 10.1 mg, 35% yield. The assignment of the *E* isomer was based on a comparison of its ^{1}H, ^{13}C, and ^{19}F NMR data with literature values.[5] **^{1}H NMR** (500 MHz, $CDCl_3$) δ 7.50 – 7.46 (m, 2H), 7.44 – 7.40 (m, 3H), 5.04 – 4.48 (m, 1H), 3.10 – 2.79 (m, 1H), 1.87 – 1.77 (m, 4H), 1.75 – 1.61 (m, 4H), 1.40 – 1.32 (m, 2H). **^{13}C NMR** (126 MHz, $CDCl_3$) δ 163.2 (q, J = 5.7 Hz), 136.1, 130.0, 129.9, 129.8, 123.8 (q, J = 271.0 Hz), 108.7 (q, J = 34.8 Hz), 42.3, 32.2, 26.4, 25.9. **^{19}F NMR** (471 MHz, $CDCl_3$) δ –54.86 (d, J = 8.6 Hz). **HRMS** (ESI-TOF) *m/z*: $[M+Na]^+$ Calcd for $C_{15}H_{17}F_3NaS^+$ 309.0901; Found 309.0904.

(*Z*)-(1-cyclohexyl-3,3,3-trifluoroprop-1-en-1-yl)(phenyl)sulfane (61')

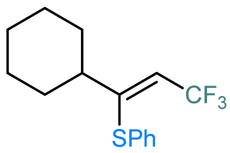

Using the General Procedure A, the title compound was obtained as colorless oil by preparative TLC using petroleum ether as the eluent; R_f = 0.7 (petroleum ether); 5.7 mg, 20% yield. The assignment of the *Z* isomer was based on a comparison of its ^{1}H, ^{13}C, and ^{19}F NMR data with literature values.[5] **^{1}H NMR** (500 MHz, $CDCl_3$) δ 7.42 – 7.37 (m, 2H), 7.36 – 7.31 (m, 3H), 5.81 – 5.74 (m, 1H), 1.95 – 1.84 (m, 3H), 1.76 – 1.66 (m, 2H), 1.62 – 1.53 (m, 2H), 1.18 – 1.07 (m, 2H), 1.05 – 0.94 (m, 2H). **^{13}C NMR** (126 MHz, $CDCl_3$) δ 156.2 (q, J = 5.3 Hz), 133.3, 132.0, 129.3, 128.4, 123.4 (q, J = 271.0 Hz), 116.7 (q, J = 34.6 Hz), 43.8, 33.2, 26.5, 26.1. **^{19}F NMR** (471 MHz, $CDCl_3$) δ –56.50 (d, J = 8.0

Hz). **HRMS** (ESI-TOF) *m/z*: $[M+Na]^+$ Calcd for $C_{15}H_{17}F_3NaS^+$ 309.0901; Found 309.0904.

(*E*)-phenyl(1,1,1-trifluoro-5-phenylpent-2-en-3-yl)sulfane (62)

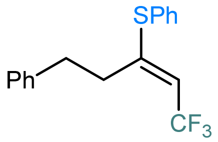

Using the General Procedure A, the title compound was obtained as colorless oil by preparative TLC using petroleum ether as the eluent; R_f = 0.6 (petroleum ether); 13.9 mg, 45% yield. The assignment of the *E* isomer was based on a comparison of its ^1H, ^{13}C, and ^{19}F NMR data with literature values[5]. **^{1}H NMR** (500 MHz, $CDCl_3$) δ 7.55 – 7.49 (m, 2H), 7.48 – 7.43 (m, 3H), 7.35 – 7.28 (m, 2H), 7.28 – 7.18 (m, 3H), 5.09 – 4.88 (m, 1H), 2.99 – 2.91 (m, 2H), 2.78 – 2.71 (m, 2H). **^{13}C NMR** (126 MHz, $CDCl_3$) δ 155.4 (q, *J* = 5.1 Hz), 140.7, 135.7, 130.1, 130.0, 129.6, 128.6, 128.6, 126.5, 123.5 (q, *J* = 271.0 Hz), 110.6 (q, *J* = 34.8 Hz), 36.0, 35.0. **^{19}F NMR** (471 MHz, $CDCl_3$) δ –55.70 (d, *J* = 8.3 Hz). **HRMS** (ESI-TOF) *m/z*: $[M+H]^+$ Calcd for $C_{17}H_{16}F_3S^+$ 309.0925; Found 309.0924. A trace amount of *Z* isomer was detected by GC-MS analysis.

(*E*)-phenyl(1,1,1-trifluoroundec-2-en-3-yl)sulfane (63)

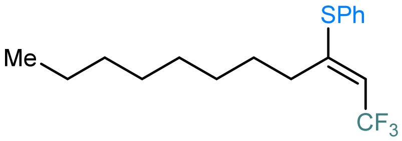

Using the General Procedure A, the title compound was obtained as colorless oil by preparative TLC using petroleum ether as the eluent; R_f = 0.6 (petroleum ether); 12.6 mg, 40% yield. The assignment of the *E* isomer was based on a comparison of its ^1H, ^{13}C, and ^{19}F NMR data with literature values[5]. **^{1}H NMR** (500 MHz, $CDCl_3$) δ 7.51 – 7.48 (m, 2H), 7.45 – 7.41 (m, 3H), 4.94 – 4.84 (m, 1H), 2.51 – 2.44 (m, 2H), 1.70 – 1.59 (m, 2H), 1.40 – 1.19 (m, 10H), 0.95 – 0.73 (m, 3H). **^{13}C NMR** (126 MHz, $CDCl_3$) δ 156.8 (q, *J* = 5.6 Hz), 135.7, 130.0, 129.93, 129.85, 123.6 (q, *J* = 270.9 Hz), 109.9 (q, *J* = 34.7 Hz), 32.8, 32.0, 29.6, 29.45, 29.35, 29.3, 22.8, 14.3. **^{19}F NMR** (471 MHz, $CDCl_3$) δ –55.49 (d, *J* = 8.7 Hz). **HRMS** (ESI-TOF) *m/z*: $[M+H]^+$ Calcd for $C_{17}H_{24}F_3S^+$ 317.1551; Found 317.1550. A trace amount of *Z* isomer was detected by GC-MS analysis.

S-phenyl 3-methoxy-2-(4,4,4-trifluorobutyl)benzothioate (64)

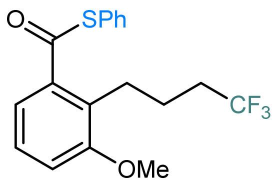

Using the General Procedure A, the title compound was obtained as yellow oil by preparative TLC using petroleum ether/EtOAc (15:1) as the eluent; R_f = 0.6 (petroleum ether/EtOAc = 15:1); 9.2 mg, 26% yield. **^{1}H NMR** (500 MHz, $CDCl_3$) δ 7.55 – 7.40 (m, 6H), 7.35 – 7.28 (m, 1H), 7.08 – 6.96 (m, 1H), 3.87 (s, 3H), 3.19 – 2.57 (m, 2H), 2.16 – 2.03 (m, 2H), 1.94 – 1.74 (m, 2H). **^{13}C NMR** (126 MHz, $CDCl_3$) δ 192.9, 158.1, 138.9, 134.9, 129.8, 129.5, 128.4, 128.3, 127.43 (q, J = 276.3 Hz), 127.41, 120.4, 113.8, 55.9, 33.7 (q, J = 28.3 Hz), 25.6, 22.3 (q, J = 3.0 Hz). **^{19}F NMR** (471 MHz, $CDCl_3$) δ –66.24 (t, J = 10.9 Hz). **HRMS** (ESI-TOF) *m/z*: $[M+H]^+$ Calcd for $C_{18}H_{18}O_2F_3S^+$ 355.0980; Found 355.0982.

6,6,6-trifluoro-4-(phenylthio)hexyl 4-(*N*,*N*-dipropylsulfamoyl)benzoate (65)

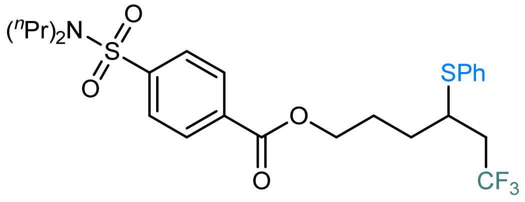

Using the General Procedure A, the title compound was obtained as yellow oil by preparative TLC using petroleum ether/EtOAc (10:1) as the eluent; R_f= 0.4 (petroleum ether/EtOAc = 10:1); 23.9 mg, 45% yield. **^{1}H NMR** (500 MHz, $CDCl_3$) δ 8.18 – 8.07 (m, 2H), 7.90 – 7.84 (m, 2H), 7.46 – 7.37 (m, 2H), 7.34 – 7.27 (m, 3H), 4.42 – 4.33 (m, 2H), 3.43 – 3.34 (m, 1H), 3.14 – 3.07 (m, 4H), 2.55 – 2.44 (m, 1H), 2.40 – 2.28 (m, 1H), 2.19 – 2.07 (m, 1H), 2.04 – 1.92 (m, 2H), 1.78 – 1.68 (m, 1H), 1.60 – 1.48 (m, 4H), 0.92 – 0.82 (m, 6H). **^{13}C NMR** (126 MHz, $CDCl_3$) δ 165.3, 144.4, 133.6, 133.1, 132.9, 130.3, 129.4, 128.2, 127.1, 126.1 (q, J = 278.1 Hz), 65.0, 50.0, 42.1 (q, J = 2.5 Hz), 39.2 (q, J = 27.6 Hz), 30.1, 25.6, 22.0, 11.3. **^{19}F NMR** (471 MHz, $CDCl_3$) δ –63.39 (t, J = 10.8 Hz). **HRMS** (ESI-TOF) *m/z*: $[M+Na]^+$ Calcd for $C_{25}H_{32}NO_4F_3NaS_2^+$ 554.1623; Found 554.1627.

6,6,6-trifluoro-4-(phenylthio)hexyl 2-(1-(4-chlorobenzoyl)-6-methoxy-2-methyl-1*H*-indol-3-yl)acetate (66)

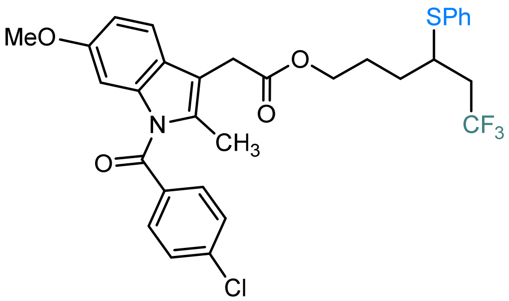

Using the General Procedure A, the title compound was obtained as yellow oil by preparative TLC using petroleum ether/EtOAc (5:1) as the eluent; R_f = 0.7 (petroleum ether/EtOAc = 5:1); 21.1 mg, 35% yield. **^{1}H NMR** (500 MHz, $CDCl_3$) δ 7.69 – 7.62 (m, 2H), 7.51 – 7.43 (m, 2H), 7.42 – 7.37 (m, 2H), 7.35 – 7.28 (m, 3H), 6.98 – 6.93 (m, 1H), 6.88 – 6.83 (m, 1H), 6.71 – 6.64 (m, 1H), 4.19 – 4.10 (m, 2H), 3.83 (s, 3H), 3.66 (s, 2H), 3.33 – 3.24 (m, 1H), 2.47 – 2.41 (m, 1H), 2.39 (s, 3H), 2.28 – 2.19 (m, 1H), 2.01 – 1.92 (m, 1H), 1.87 – 1.76 (m, 2H), 1.62 – 1.52 (m, 1H). **^{13}C NMR** (126 MHz, $CDCl_3$) δ 171.0, 168.4, 156.2, 139.4, 136.1, 134.0, 133.1, 133.0, 131.3, 130.9, 130.8, 129.4, 129.2, 128.1, 126.0 (q, J = 278.1 Hz), 115.1, 112.6, 111.7, 101.5, 64.5, 55.8, 42.2 (q, J = 2.3 Hz), 39.2 (q, J = 27.5 Hz), 30.5, 30.2, 25.7, 13.5. **^{19}F NMR** (471 MHz, $CDCl_3$) δ –63.38 (t, J = 10.8 Hz). **HRMS** (ESI-TOF) *m/z*: $[M+Na]^+$ Calcd for $C_{31}H_{29}NO_4F_3NaSCl^+$ 626.1356; Found 626.1358.

(1R,2S,5R)-2-isopropyl-5-methylcyclohexyl 6,6,6-trifluoro-4-(phenylthio)hexanoate (67)

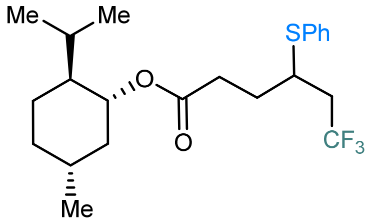

Using the General Procedure A, the title compound was obtained as colorless oil by preparative TLC using petroleum ether/EtOAc (20:1) as the eluent; R_f = 0.8 (petroleum ether/EtOAc = 20:1); 20.8 mg, 50% yield, The product was obtained as an inseparable mixture of isomers. The diastereomeric ratio was determined by ^{19}F NMR analysis of the inseparable mixture of isomers (*d.r.* = 1.0:1). The following NMR data represents the combined peaks of both components. **^{1}H NMR** (500 MHz, $CDCl_3$) δ 7.44 – 7.39 (m, 2H), 7.35 – 7.28 (m, 3H), 4.74 – 4.63 (m, 1H), 3.44 – 3.31 (m, 1H), 2.63 – 2.56 (m, 2H), 2.53 – 2.43 (m, 1H), 2.39 – 2.36 (m, 1H), 2.35 – 2.26 (m, 1H), 2.22 – 2.12 (m, 1H), 2.01 – 1.93 (m, 1H), 1.88 – 1.77 (m, 3H), 1.72 – 1.61 (m, 3H), 0.95 – 0.84 (m,

8H), 0.75 (d, J = 6.9 Hz, 3H). **^{13}C NMR** (126 MHz, $CDCl_3$) δ 172.41, 172.38, 133.32, 133.31, 133.0, 132.9, 129.4, 128.2, 126.1 (q, J = 278.4 Hz), 74.6, 47.21, 47.17, 42.3 (q, J = 2.4 Hz), 42.2 (q, J = 2.2 Hz), 41.1 (q, J = 5.2 Hz), 39.7 (q, J = 27.8 Hz), 34.5, 34.4, 31.8, 31.5, 29.3, 29.1, 26.53, 26.50, 23.7, 23.6, 22.2, 22.1, 20.89, 20.87, 16.50, 16.46. **^{19}F NMR** (471 MHz, comp. pulse decoupling, $CDCl_3$) δ –63.36 (d, J = 3.4 Hz). **HRMS** (ESI-TOF) *m/z*: $[M+H]^+$ Calcd for $C_{22}H_{32}O_2F_3S^+$ 417.2075; Found 417.2072.

6,6,6-trifluoro-4-(phenylthio)hexyl 3-methyl-4-oxo-2-phenyl-4*H*-chromene-8-carboxylate (68)

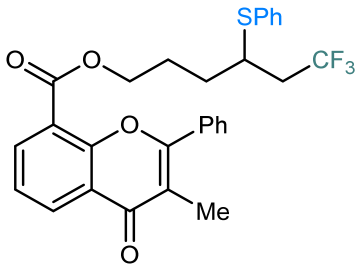

Using the General Procedure A, the title compound was obtained as yellow oil by preparative TLC using petroleum ether/EtOAc (5:1) as the eluent; R_f = 0.5 (petroleum ether/EtOAc = 5:1); 21.6 mg, 41% yield. **^{1}H NMR** (500 MHz, $CDCl_3$) δ 8.52 – 8.43 (m, 1H), 8.26 – 8.19 (m, 1H), 7.80 – 7.72 (m, 2H), 7.57 – 7.48 (m, 3H), 7.48 – 7.42 (m, 1H), 7.40 – 7.34 (m, 2H), 7.30 – 7.21 (m, 3H), 4.40 – 4.34 (m, 2H), 3.30 – 3.21 (m, 1H), 2.45 – 2.35 (m, 1H), 2.24 (s, 3H), 2.22 – 2.15 (m, 1H), 2.11 – 2.01 (m, 1H), 1.98 – 1.89 (m, 1H), 1.87 – 1.77 (m, 1H), 1.65 – 1.53 (m, 1H). **^{13}C NMR** (126 MHz, $CDCl_3$) δ 178.4, 164.5, 161.2, 154.6, 136.2, 133.1, 133.1, 132.9, 130.9, 130.6, 129.4, 129.3, 128.6, 128.1, 126.0 (q, J = 278.5 Hz), 124.1, 123.4, 120.7, 117.8, 65.0, 42.0 (q, J = 2.5 Hz), 39.1 (q, J = 27.7 Hz), 30.1, 25.6, 11.9. **^{19}F NMR** (471 MHz, $CDCl_3$) δ –63.40 (t, J = 11.1 Hz). **HRMS** (ESI-TOF) *m/z*: $[M+H]^+$ Calcd for $C_{29}H_{26}O_4F_3S^+$ 527.1504; Found 527.1508.

(8*R*,9*S*,13*S*,14*S*)-13-methyl-17-oxo-7,8,9,11,12,13,14,15,16,17-decahydro-6*H*-cyclopenta[*a*]phenanthren-3-yl 6,6,6-trifluoro-4-(phenylthio)hexanoate (69)

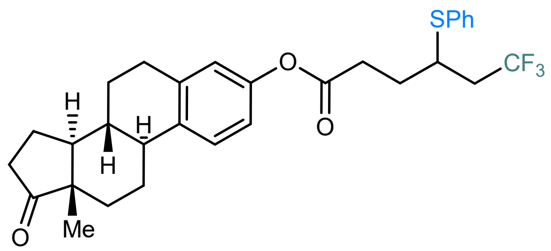

Using the General Procedure A, the title compound was obtained as yellow oil by preparative TLC using petroleum ether/EtOAc (5:1) as the eluent; R_f=0.5 (petroleum ether/EtOAc = 5:1); 25.4 mg, 48% yield. **^{1}H NMR** (500 MHz, $CDCl_3$) δ 7.41 – 7.35 (m, 2H), 7.30 – 7.16 (m, 4H), 6.79 – 6.74 (m, 1H), 6.73 – 6.70 (m, 1H), 3.42 – 3.34 (m, 1H), 2.86 – 2.76 (m, 2H), 2.52 – 2.38 (m, 2H), 2.35 – 2.29 (m, 1H), 2.28 – 2.16 (m, 2H), 2.13 – 2.01 (m, 1H), 2.01 – 1.79 (m, 4H), 1.63 – 1.31 (m, 8H), 1.23 – 1.11 (m, 1H), 0.83 (s, 3H). **^{13}C NMR** (126 MHz, $CDCl_3$) δ 171.7, 148.5, 138.2, 137.6, 133.3, 132.7, 129.4, 128.3, 126.5, 126.0 (q, J = 278.2 Hz), 121.6, 118.8, 50.5, 48.1, 44.2, 42.1 (q, J = 2.3 Hz), 39.7 (q, J = 27.7 Hz), 38.1, 36.0, 31.6, 31.5, 29.5, 28.9, 26.4, 25.9, 21.7, 13.9. **^{19}F NMR** (471 MHz, $CDCl_3$) δ –63.31 (t, J = 10.8 Hz). **HRMS** (ESI-TOF) *m/z*: $[M+H]^+$ Calcd for $C_{30}H_{34}O_3F_3S^+$ 531.2181; Found 531.2182.

(1-(4'-(3,4-difluorophenyl)-[1,1'-bi(cyclohexan)]-4-yl)-3,3,3-trifluoropropyl)(phenyl)sulfane (70)

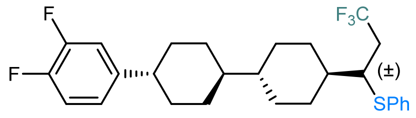

Using the General Procedure A, the title compound was obtained as colorless oil by preparative TLC using petroleum ether/EtOAc (15:1) as the eluent; R_f=0.5 (petroleum ether/EtOAc = 15:1); 24.6 mg, 51% yield. **^{1}H NMR** (500 MHz, $CDCl_3$) δ 7.43 – 7.39 (m, 2H), 7.34 – 7.29 (m, 2H), 7.28 – 7.24 (m, 1H), 7.08 – 6.95 (m, 2H), 6.91 – 6.87 (m, 1H), 3.34 – 3.27 (m, 1H), 2.59 – 2.47 (m, 1H), 2.44 – 2.30 (m, 2H), 1.94 – 1.78 (m, 8H), 1.75 – 1.66 (m, 1H), 1.54 – 1.43 (m, 1H), 1.40 – 1.30 (m, 2H), 1.23 – 0.96 (m, 7H). **^{13}C NMR** (126 MHz, $CDCl_3$) δ 150.3 (dd, J = 246.6, 12.6 Hz), 148.7 (dd, J = 245.0, 12.9 Hz), 144.9 (dd, J = 5.0, 3.8 Hz), 135.3, 132.0, 129.3, 127.4, 126.4 (q, J = 278.3 Hz), 122.6 (dd, J = 6.0, 3.4 Hz), 116.9 (d, J = 16.8 Hz), 115.5(d, J = 16.7 Hz), 49.2 (q, J = 2.3 Hz), 43.9, 43.1, 42.7, 41.5, 37.2 (q, J = 27.4 Hz), 34.6, 30.7, 30.28, 30.27, 29.9, 29.8, 28.3. **^{19}F NMR** (471 MHz, $CDCl_3$) δ –63.61 (t, 3F),

–138.64 (m, 1F), –142.51 (m, 1F). **HRMS** (ESI-TOF) *m/z*: $[M+H]^+$ Calcd for $C_{27}H_{32}F_5S^+$ 483.2145; Found 483.2145.

Diverse Functionalization of Phenyl(4,4,4-trifluoro-1-phenylbutan-2-yl)sulfane.[3,6]

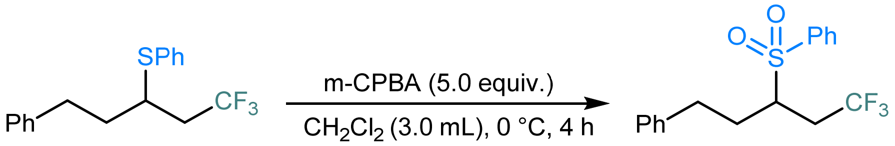

8 (1.0 equiv.) **71**, 82%

According to the procedures reported in the literature,[3] an oven-dried vial (4 mL) equipped with a magnetic stir bar was charged with **8** (0.2 mmol, 1.0 equiv.), DCM (3.0 mL), and *m*-CPBA (1.0 mmol, 5.0 equiv.). Then the resulting reaction mixture was stirred at 0 °C for 4 h. After completion, the reaction mixture was then washed with saturated aqueous $NaHCO_3$ (~10 mL × 3) to remove residual *m*-CPBA and the corresponding acid byproduct. The organic phase was dried over anhydrous Na_2SO_4, and the solvent was removed *in vacuo*. The residue was purified by silica gel column chromatography (petroleum ether/ethyl acetate = 15:1 to 4:1) to afford compound **71** as a white solid (82% yield).

(5,5,5-trifluoro-3-(phenylsulfonyl)pentyl)benzene (71): The title compound was obtained as a white solid by preparative TLC using petroleum ether/EtOAc (15:1) as the eluent; 56.1 mg, 82% yield; R_f=0.5 (petroleum ether/EtOAc = 15:1); **^{1}H NMR** (500 MHz, $CDCl_3$) δ 7.83 – 7.74 (m, 2H), 7.64 – 7.55 (m, 1H), 7.52 – 7.43 (m, 2H), 7.20 – 7.05 (m, 3H), 7.02 – 6.92 (m, 2H), 3.26 – 3.09 (m, 1H), 2.80 – 2.67 (m, 2H), 2.63 – 2.48 (m, 1H), 2.41 – 2.25 (m, 1H), 2.21 – 2.08 (m, 1H), 2.01 – 1.90 (m, 1H). **^{13}C NMR** (126 MHz, $CDCl_3$) δ 140.0, 136.8, 134.4, 129.6, 128.9, 128.7, 128.4, 126.5, 125.7 (q, J = 277.4 Hz), 58.1 (q, J = 2.3 Hz), 32.8 (q, J = 30.2 Hz), 32.3, 30.3. **^{19}F NMR** (471 MHz, $CDCl_3$) δ –63.45 (t, J = 10.5 Hz). **HRMS** (ESI-TOF) *m/z*: $[M+Na]^+$ Calcd for $C_{17}H_{17}O_2F_3NaS^+$ 365.0799; Found 365.0803. M.p.: 75–76 °C.

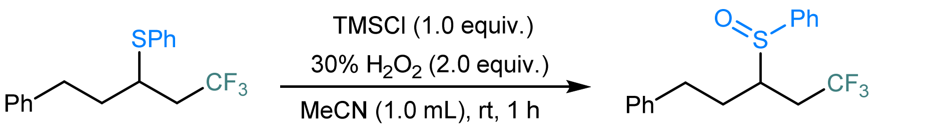

8 (1.0 equiv.) **72, 72'** (41%, 29%), d.r.= 1.4:1

According to the procedures reported in the literature,[3] an oven-dried vial (4 mL) containing a magnetic stir bar was charged with **8** (0.2 mmol, 1.0 equiv.), MeCN (1.0 mL), TMSCl (0.2 mmol, 1.0 equiv.), and 30% aqueous H_2O_2 (0.4 mmol, 2.0 equiv.). Then the resulting reaction mixture was stirred at room temperature for 1 h. After completion, the reaction was quenched with saturated aqueous $Na_2S_2O_3$ (5 mL), and the mixture was extracted with ethyl acetate (~10 mL × 3). The combined organic layers were dried over anhydrous Na_2SO_4 and concentrated in vacuo. The residue was purified by column chromatography on silica gel and eluted with petroleum ether/ethyl acetate (10:1 to 4:1) to afford compound **72** as a colorless oil (41% yield) and compound **72'** as a colorless oil (29% yield), *d.r.* = 1.4:1.

(5,5,5-trifluoro-3-(phenylsulfinyl)pentyl)benzene (72): The title compound was obtained as colorless oil by preparative TLC using petroleum ether/EtOAc (5:1) as the eluent; 26.8 mg, 41% yield; R_f=0.5 (petroleum ether/EtOAc = 5:1); ^{1}**H NMR** (500 MHz, $CDCl_3$) δ 7.55 – 7.43 (m, 5H), 7.37 – 7.30 (m, 2H), 7.27 – 7.21 (m, 3H), 3.09 – 3.00 (m, 1H), 2.98 – 2.89 (m, 1H), 2.77 – 2.66 (m, 1H), 2.42 – 2.32 (m, 1H), 2.31 – 2.25 (m, 2H), 2.25 – 2.14 (m, 1H). ^{13}**C NMR** (126 MHz, $CDCl_3$) δ 141.1, 139.8, 131.3, 129.4, 128.8, 128.5, 126.6, 126.5 (q, J = 277.1 Hz), 124.3, 57.8 (q, J = 1.6 Hz), 32.6, 30.7, 30.0 (q, J = 29.9 Hz). ^{19}**F NMR** (471 MHz, $CDCl_3$) δ –63.16 (t, J = 11.0 Hz). **HRMS** (ESI-TOF) *m/z*: $[M+H]^+$ Calcd for $C_{17}H_{18}OF_3S^+$ 327.1030; Found 327.1034.

(5,5,5-trifluoro-3-(phenylsulfinyl)pentyl)benzene (72'): The title compound was obtained as colorless oil by preparative TLC using petroleum ether/EtOAc (5:1) as the eluent; 19.0 mg, 29% yield; R_f=0.4 (petroleum ether/EtOAc = 5:1); ^{1}**H NMR** (500 MHz, CDCl3) δ 7.53 (m, 4H), 7.32 – 7.14 (m, 4H), 7.05 – 6.98 (m, 2H), 3.00 – 2.84 (m, 2H), 2.82 – 2.73 (m, 1H), 2.67 – 2.58 (m, 1H), 2.11 – 1.98 (m, 1H), 1.96 – 1.81 (m, 2H). ^{13}**C NMR** (126 MHz, $CDCl_3$) δ 139.9, 139.8, 131.6, 129.4, 128.8, 128.3, 126.6, 126.5 (q, J = 277.4 Hz), 125.1, 56.7 (q, J = 1.6 Hz), 32.6, 31.9 (q, J = 29.5 Hz), 28.3.

^{19}F NMR (471 MHz, $CDCl_3$) δ –63.16 (t, J = 10.5 Hz). **HRMS** (ESI-TOF) *m/z*: $[M+H]^+$ Calcd for $C_{17}H_{18}OF_3S^+$ 327.1030; Found 327.1034.

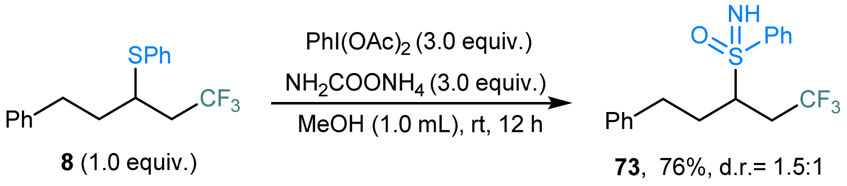

According to the procedures reported in the literature,[3,6] an oven-dried 4 mL vial containing a magnetic stir bar was charged with **8** (0.2 mmol, 1.0 equiv.), (diacetoxyiodo)benzene (0.6 mmol, 3.0 equiv.), ammonium carbamate (0.6 mmol, 3.0 equiv.), and MeOH (1.0 mL). Then the resulting reaction mixture was stirred at room temperature for 12 h. After completion, the solvent was removed *in vacuo*, and the residue was purified by silica gel column chromatography (petroleum ether/ethyl acetate = 15:1) to afford compound **73** as a colorless oil (76% yield, *d.r.* = 1.5:1).

Imino(phenyl)(1,1,1-trifluoro-5-phenylpentan-3-yl)-λ^6-sulfanone (73): The title compound was obtained as yellow oil by preparative TLC using petroleum ether/EtOAc (15:1) as the eluent; 52.0 mg, 76% yield; R_f=0.5 (petroleum ether/EtOAc = 15:1); The product was obtained as an inseparable mixture of isomers. The diastereomeric ratio was determined by ^{19}F NMR spectroscopy of the inseparable mixture of isomers (*d.r.* = 1.5:1). The following NMR data represent the combined peaks of both isomers. **^{1}H NMR** (500 MHz, $CDCl_3$) δ 7.97 – 7.87 (m, 2H), 7.69 – 7.62 (m, 1H), 7.58 – 7.54 (m, 2H), 7.27 – 7.21 (m, 2H), 7.19 – 7.14 (m, 1H), 7.09 – 6.99 (m, 2H), 3.31 – 3.23 (m, 1H), 3.02 – 2.92 (m, 1H), 2.86 – 2.76 (m, 2H), 2.67 – 2.57 (m, 1H), 2.48 – 2.37 (m, 1H), 2.37 – 2.22 (m, 1H), 2.12 – 1.93 (m, 1H). **^{13}C NMR** (126 MHz, $CDCl_3$) δ 140.25, 140.23, 139.8, 139.6, 133.7, 133.6, 129.4, 129.18, 129.13, 129.0, 128.6, 128.4, 128.3, 127.7, 126.37, 126.36, 125.9 (q, J = 277.1 Hz), 59.5 (q, J = 2.2 Hz), 59.2 (q, J = 2.1 Hz), 32.7, 32.5, 31.0, 30.7. **^{19}F NMR** (471 MHz, comp. pulse decoupling, $CDCl_3$) δ –63.32, –63.35. **HRMS** (ESI-TOF) *m/z*: $[M+H]^+$ Calcd for $C_{17}H_{19}NOF_3S^+$ 342.1139; Found 342.1143.

X-ray Crystallographic Analysis

A vacuum-dried pure sample of compound **71** (around 50–80 mg) was transferred into a vial and dissolved in 2 mL of CH_2Cl_2 followed by the layering of 2.5 mL hexane. The vial was kept at room temperature to allow for slow evaporation. White, blocky crystals were formed after 1–2 weeks. The crystals were subjected to the single crystal X-ray crystallographic analysis. Crystallographic data for compound **71** (CCDC 2537092) has been deposited at the Cambridge Crystallographic Data Centre. These data can be obtained free of charge via www.ccdc.cam.ac.uk/data_request/cif. The thermal ellipsoid plot of compound **71**, drawn at the 50% probability level, is provided in the Supporting Information (Figure S10).

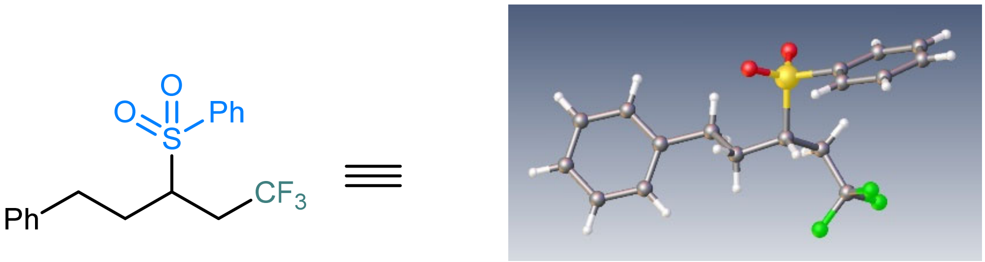

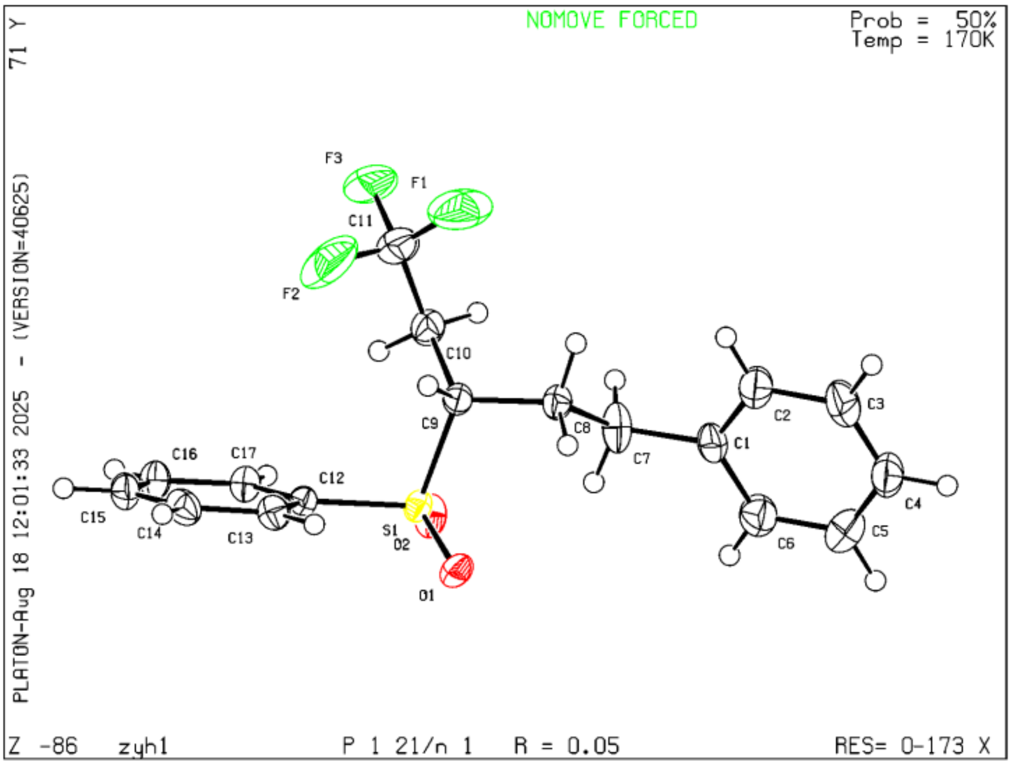

Figure S10. Thermal ellipsoid plot of compound **71** at the 50% probability level.

Empirical formula	$C_{17}H_{17}F_3O_2S$
Formula weight	342.36
Temperature/K	170
Crystal system	monoclinic
Space group	$P2_1/n$
a/Å	5.4433(2)
b/Å	14.2877(4)
c/Å	20.7876(7)
α/°	90
β/°	94.1270(10)
γ/°	90
Volume/Å^3	1612.51(9)
Z	4
ρcalcg/cm^3	1.410
μ/mm^{-1}	0.238
F(000)	712.0
Crystal size/mm^3	0.15 × 0.06 × 0.05
Radiation	MoKα (λ = 0.71073)
2Θ range for data collection/°	3.928 to 52.754
Index ranges	$-6 \leq h \leq 6, -17 \leq k \leq 17, -25 \leq l \leq 25$
Reflections collected	18311
Independent reflections	3289 [R_{int} = 0.0733, R_{sigma} = 0.0509]
Data/restraints/parameters	3289/0/208
Goodness-of-fit on F^2	1.041
Final R indexes [I>=2σ (I)]	R_1 = 0.0476, wR_2 = 0.0960
Final R indexes [all data]	R_1 = 0.0697, wR_2 = 0.1071
Largest diff. peak/hole / e Å^{-3}	0.48/-0.40

NMR Spectra

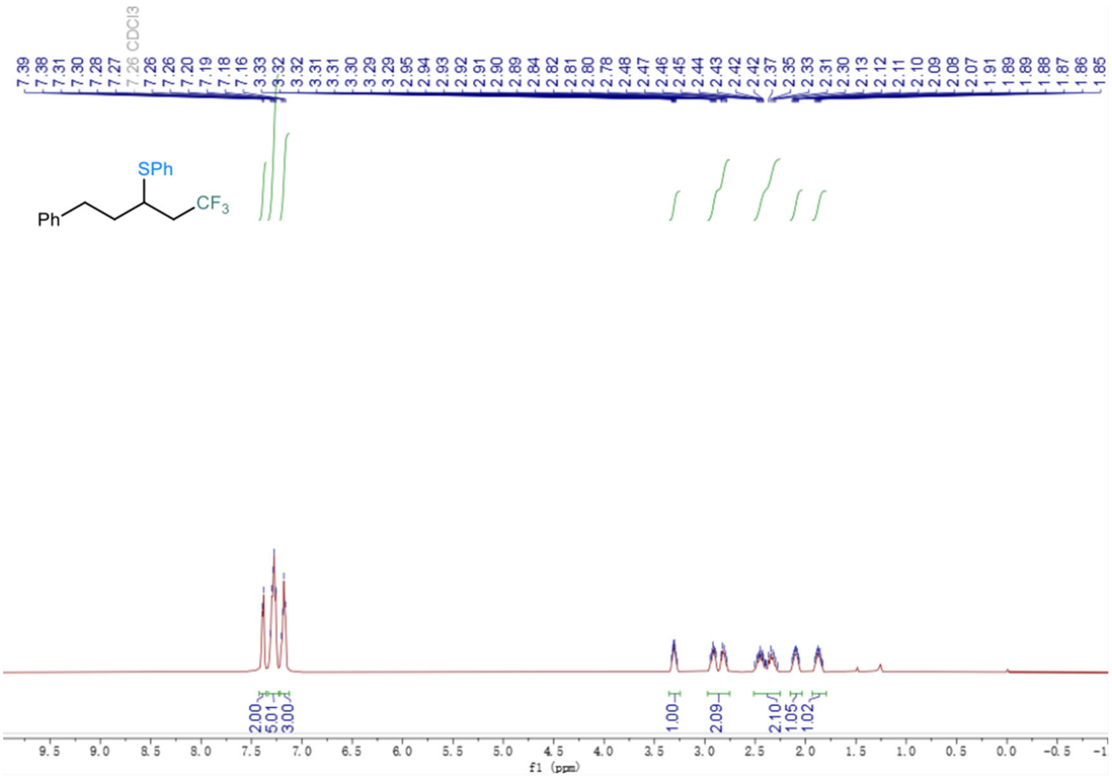

^{1}H NMR spectrum (500 MHz, Chloroform-*d*) of **8**

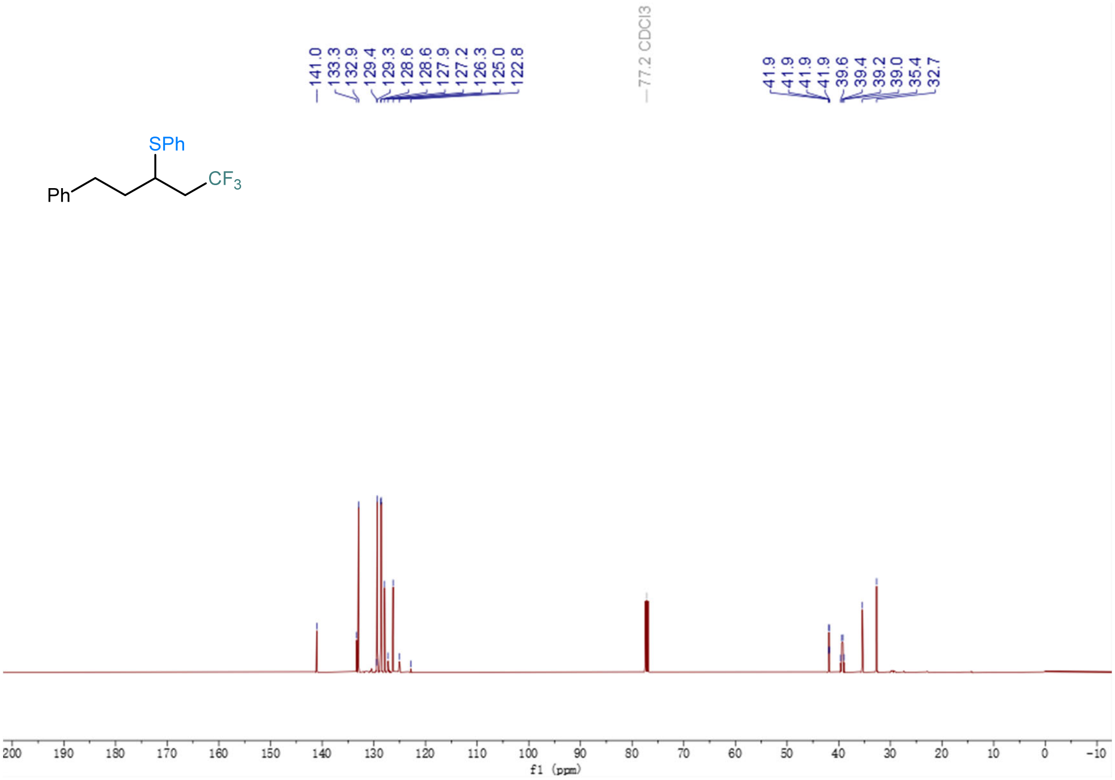

^{13}C NMR spectrum (126 MHz, Chloroform-*d*) of **8**

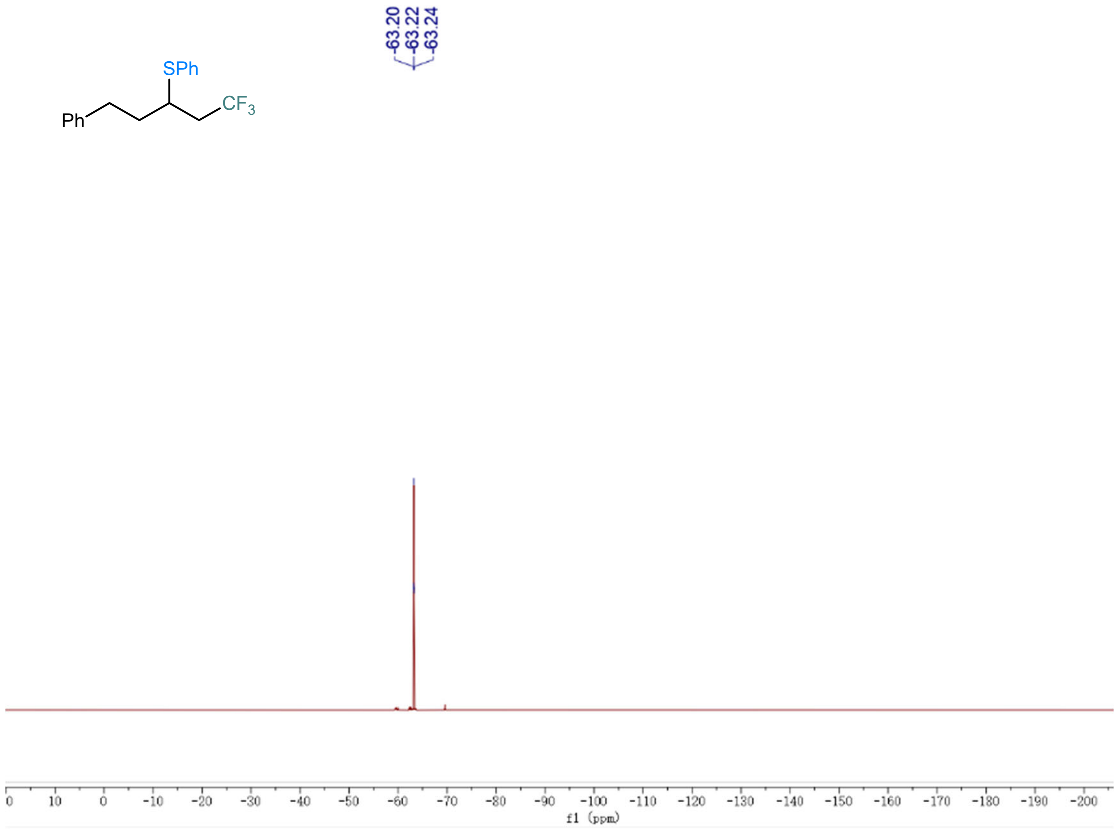

^{19}F NMR spectrum (471 MHz, Chloroform-*d*) of **8**

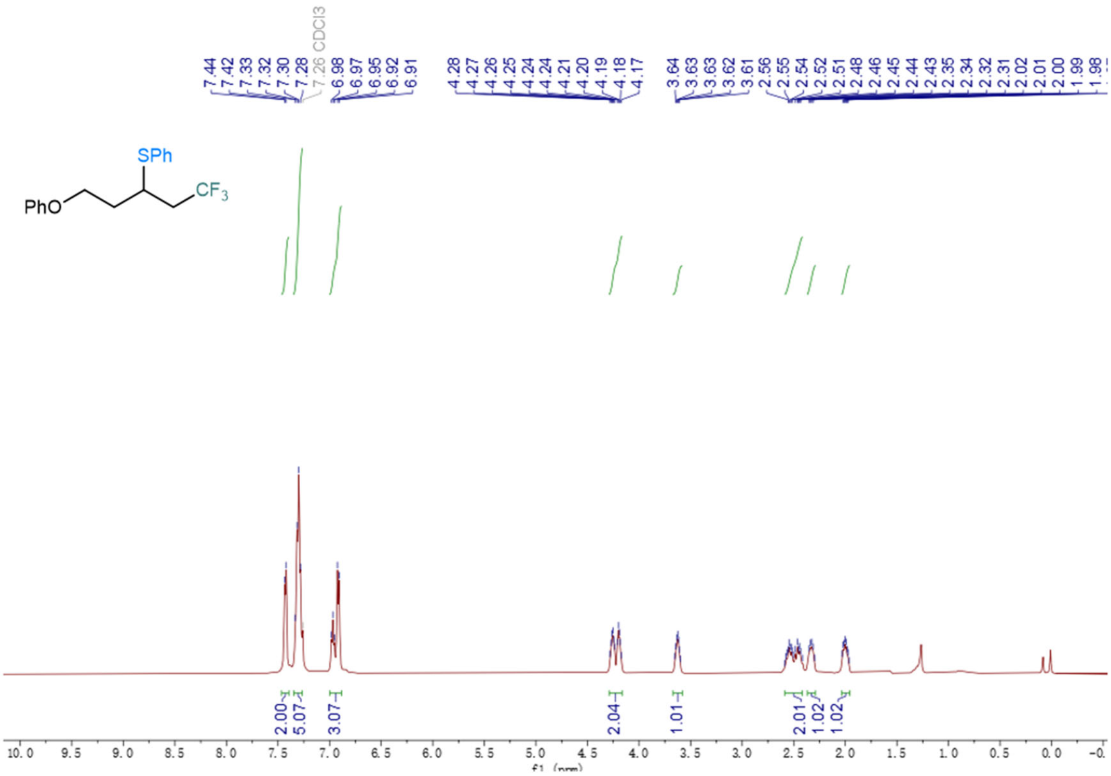

^{1}H NMR spectrum (500 MHz, Chloroform-*d*) of **9**

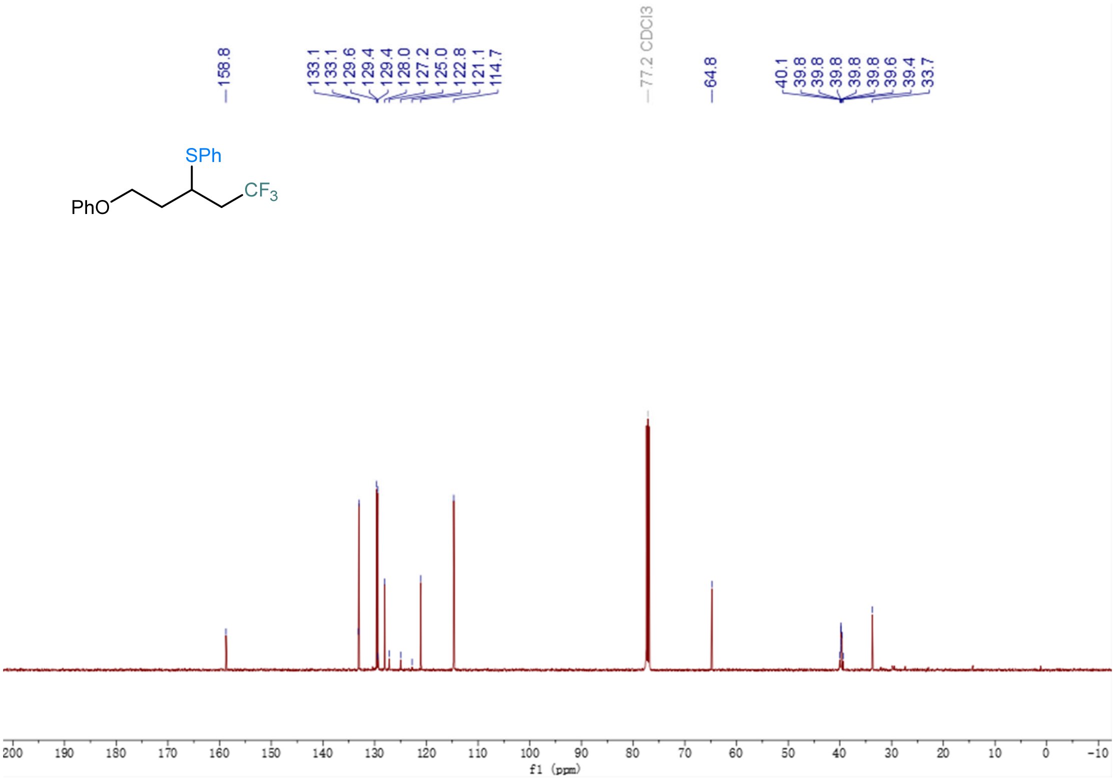

^{13}C NMR spectrum (126 MHz, Chloroform-*d*) of **9**

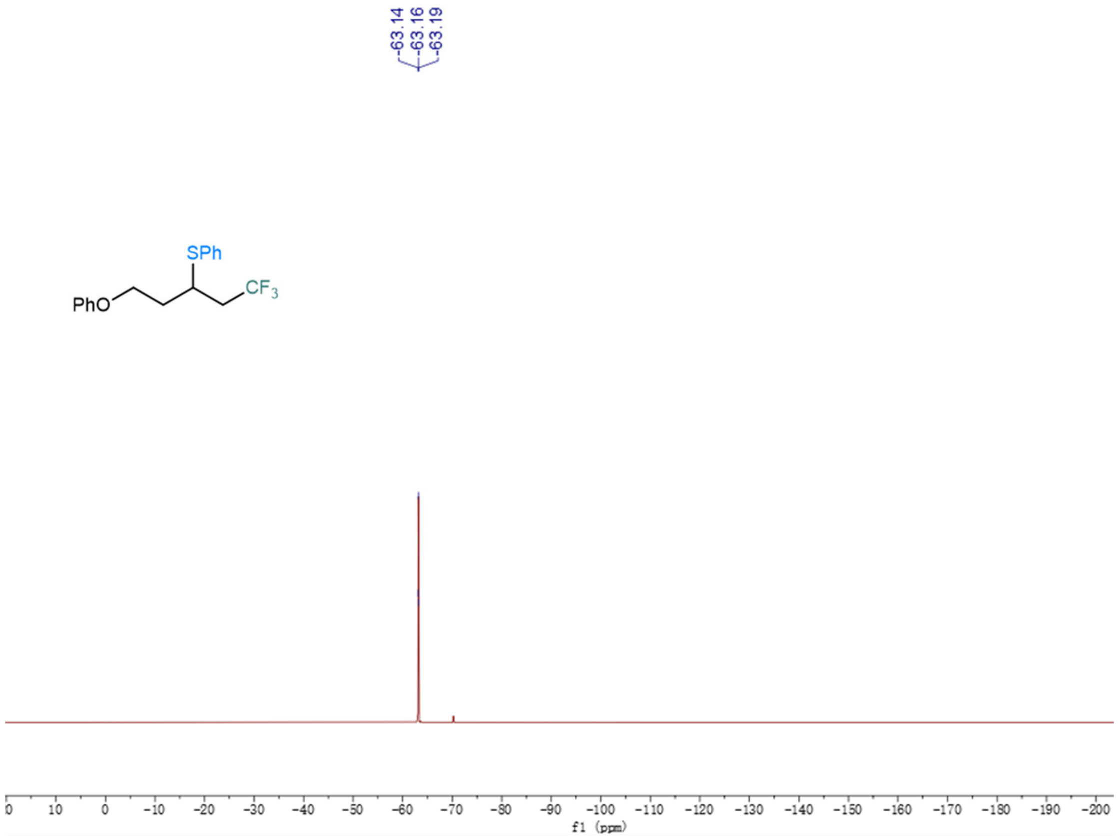

^{19}F NMR spectrum (471 MHz, Chloroform-*d*) of **9**

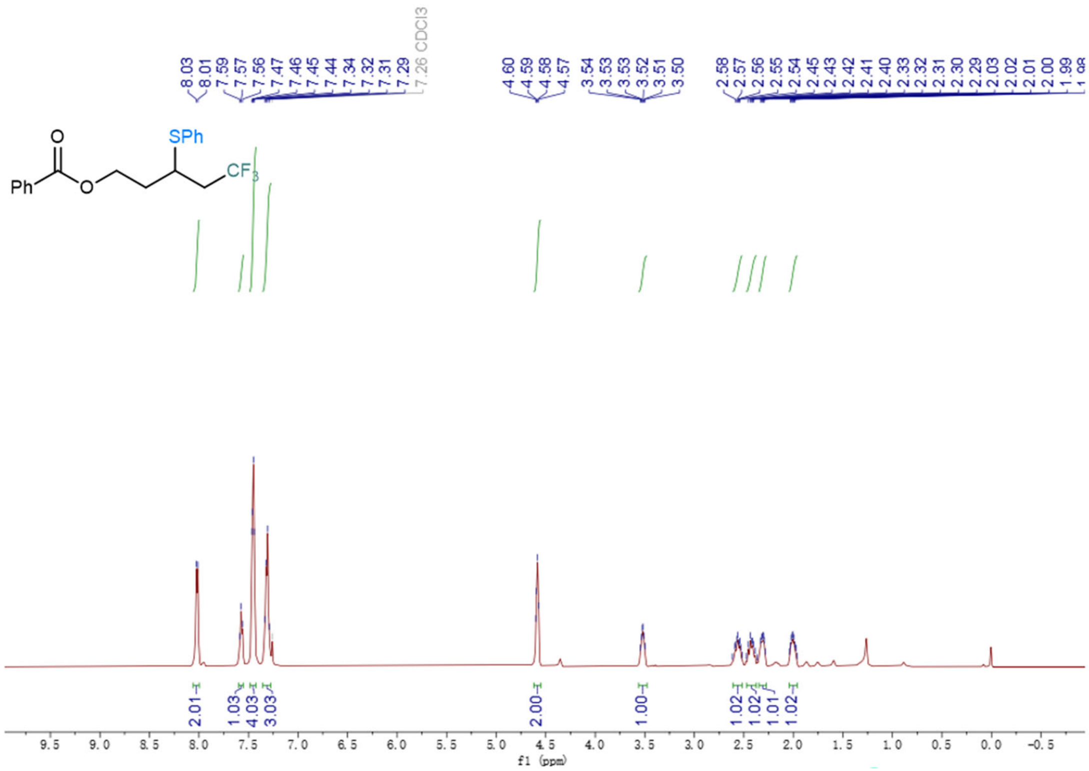

^{1}H NMR spectrum (500 MHz, Chloroform-*d*) of **10**

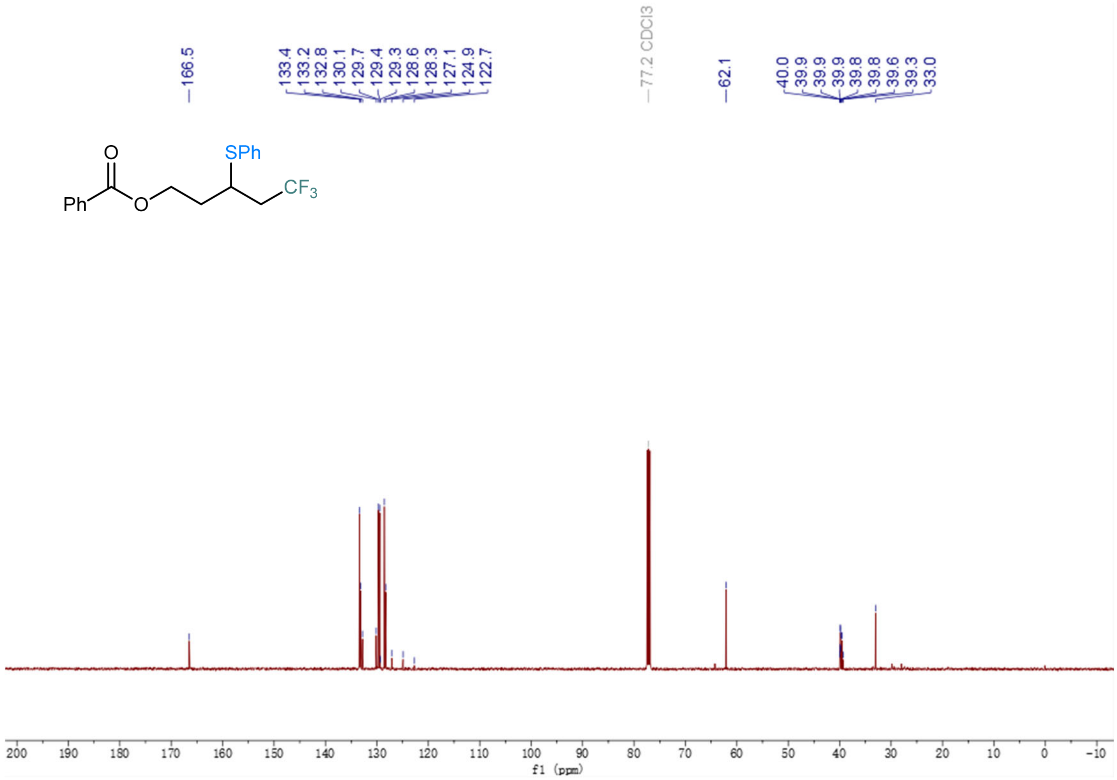

^{13}C NMR spectrum (126 MHz, Chloroform-*d*) of **10**

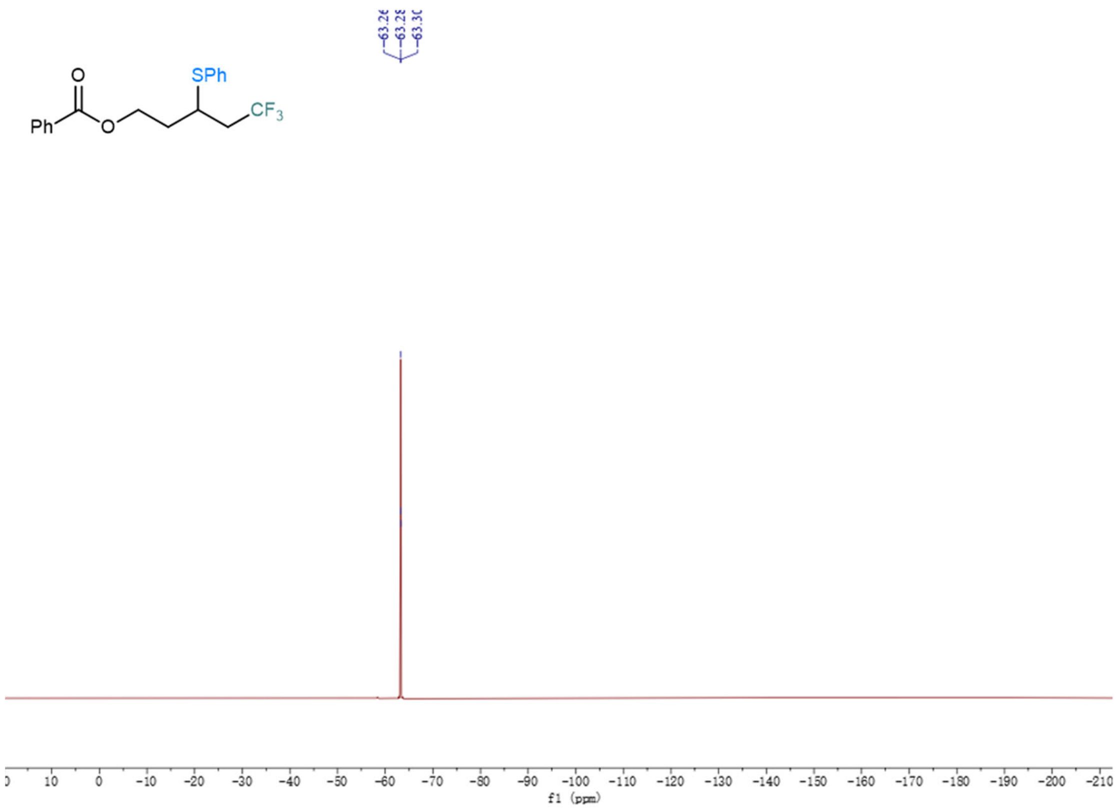

^{19}F NMR spectrum (471 MHz, Chloroform-*d*) of **10**

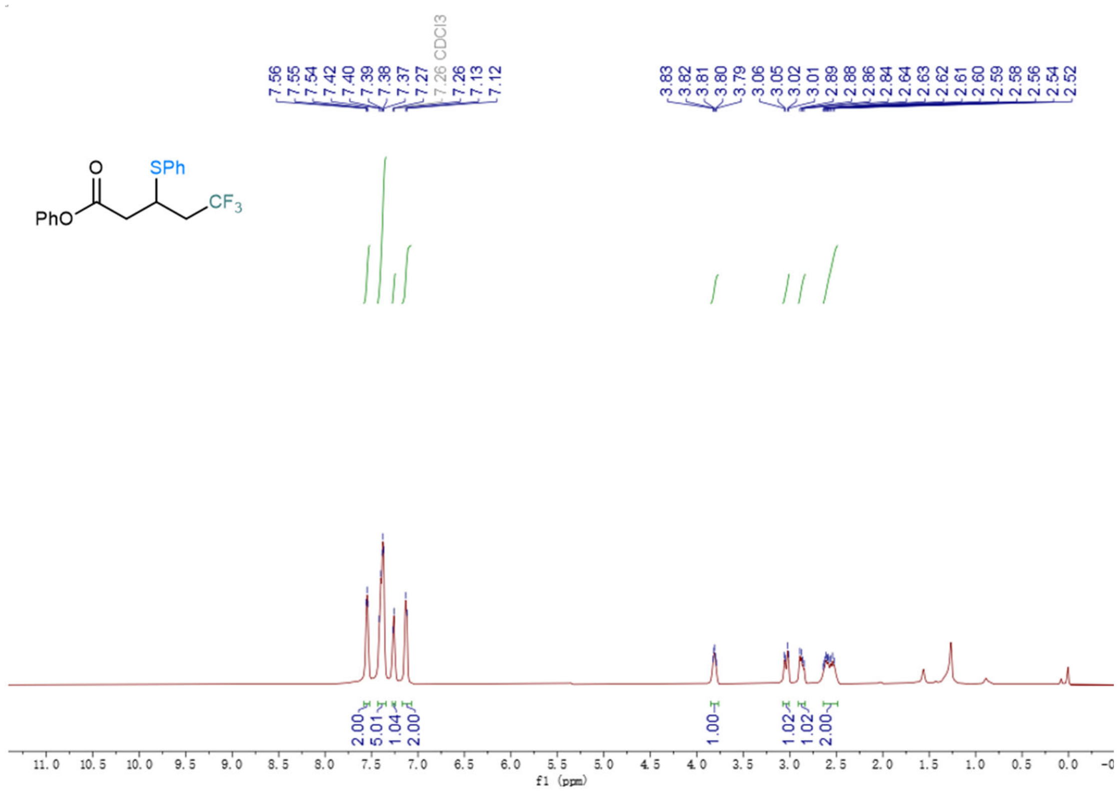

^{1}H NMR spectrum (500 MHz, Chloroform-*d*) of **11**

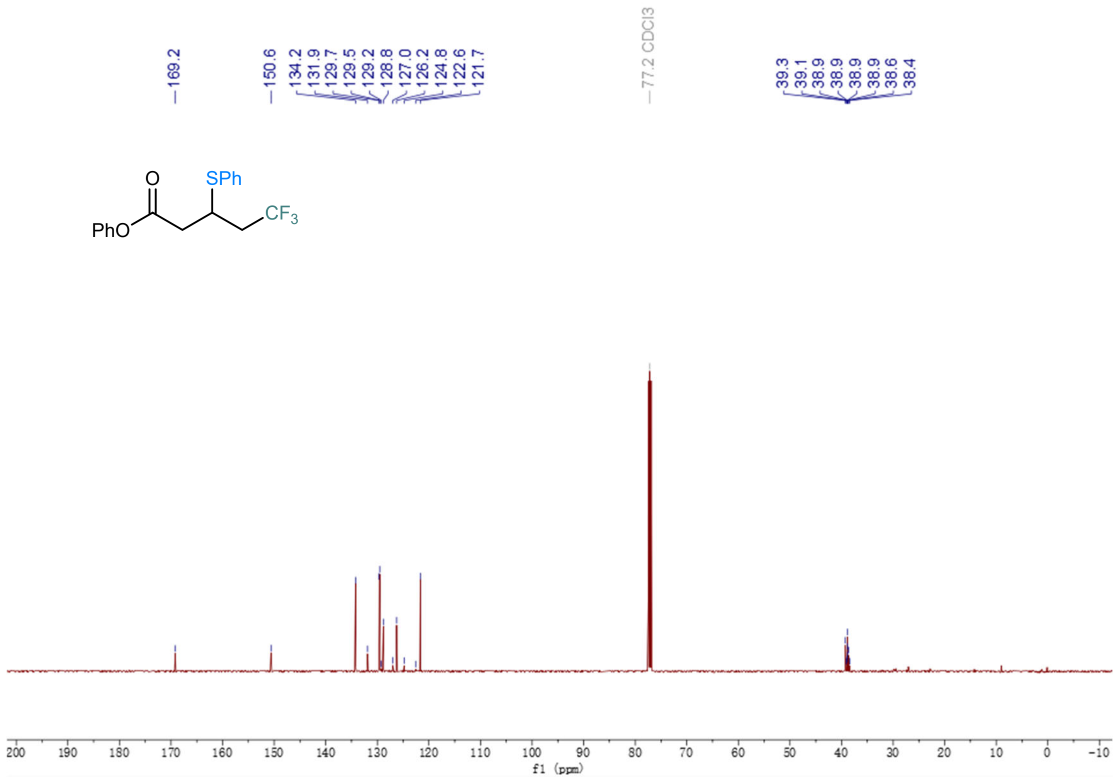

^{13}C NMR spectrum (126 MHz, Chloroform-*d*) of **11**

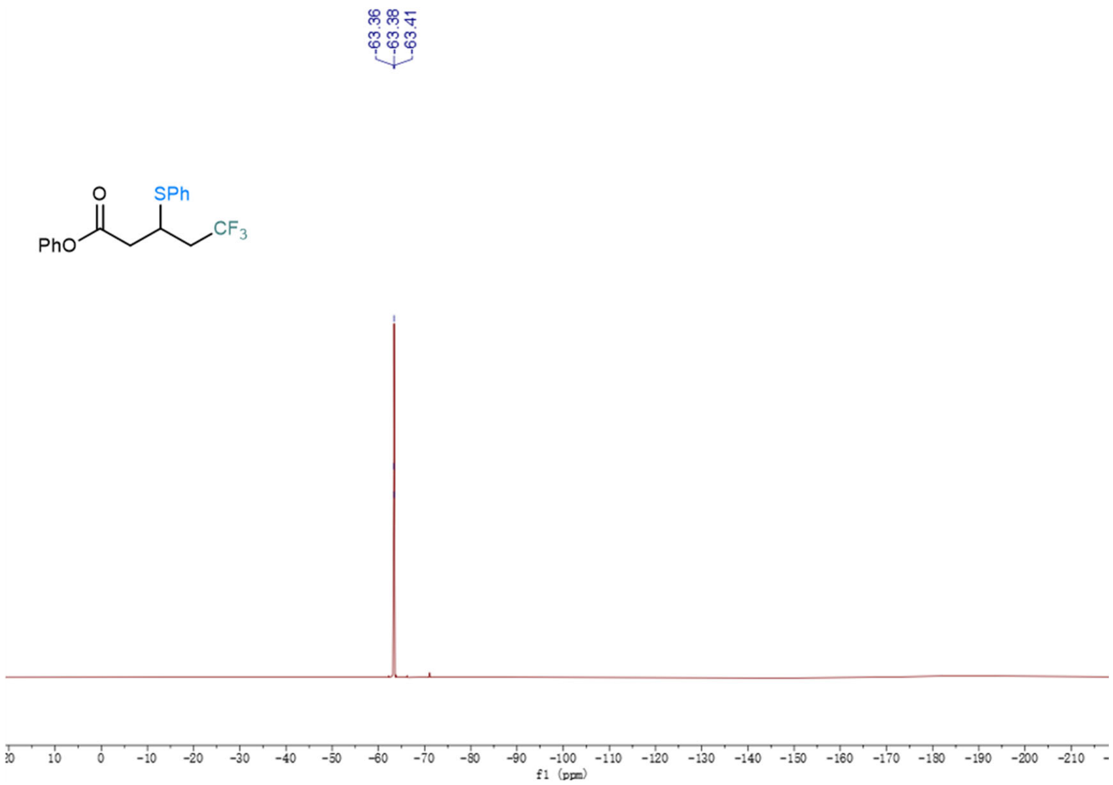

^{19}F NMR spectrum (471 MHz, Chloroform-*d*) of **11**

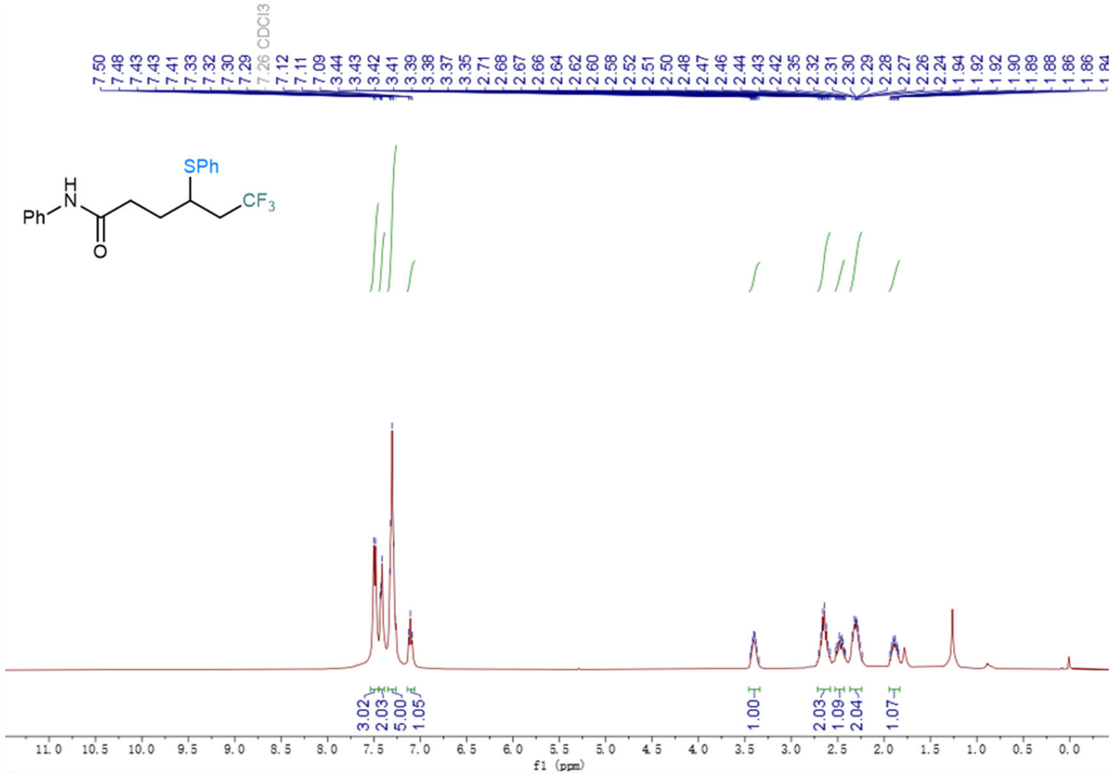

^{1}H NMR spectrum (500 MHz, Chloroform-*d*) of **12**

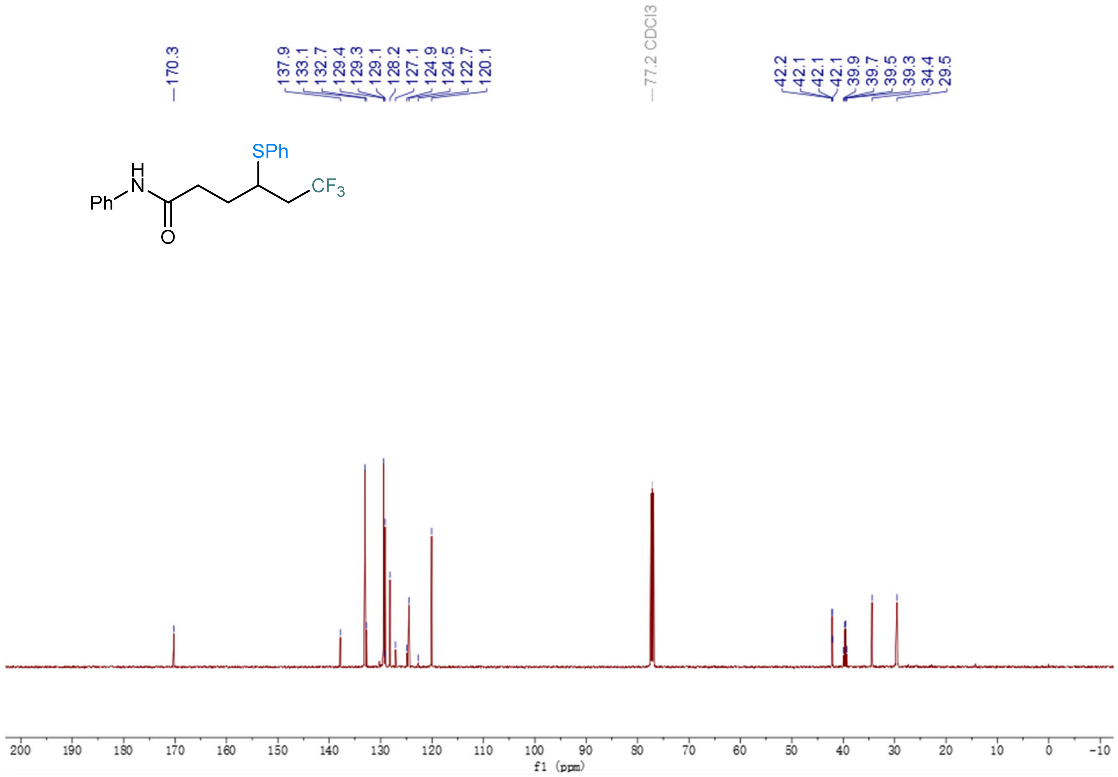

^{13}C NMR spectrum (126 MHz, Chloroform-*d*) of **12**

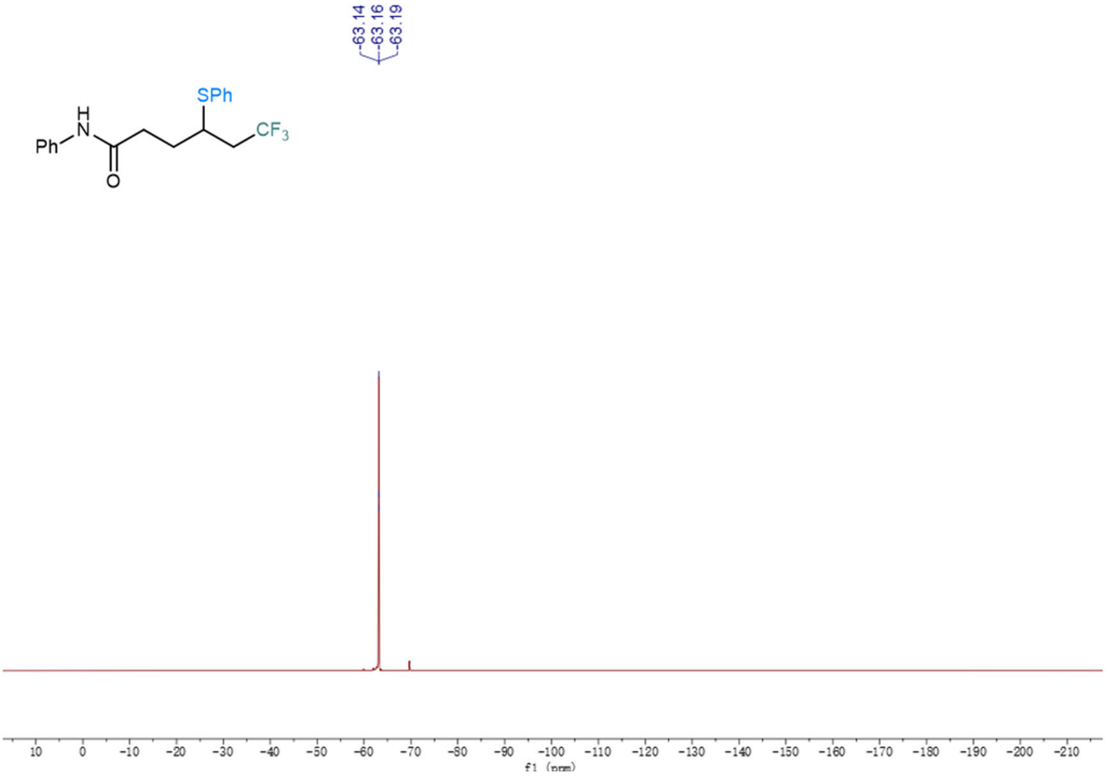

^{19}F NMR spectrum (471 MHz, Chloroform-*d*) of **12**

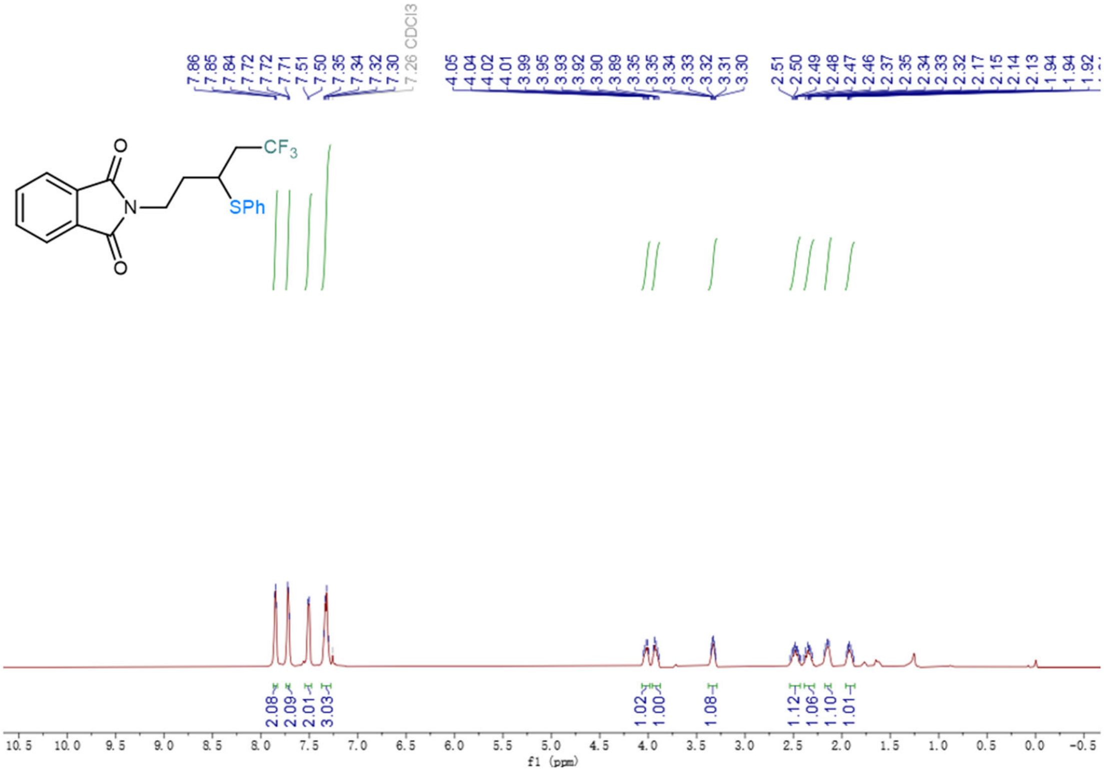

^{1}H NMR spectrum (500 MHz, Chloroform-*d*) of **13**

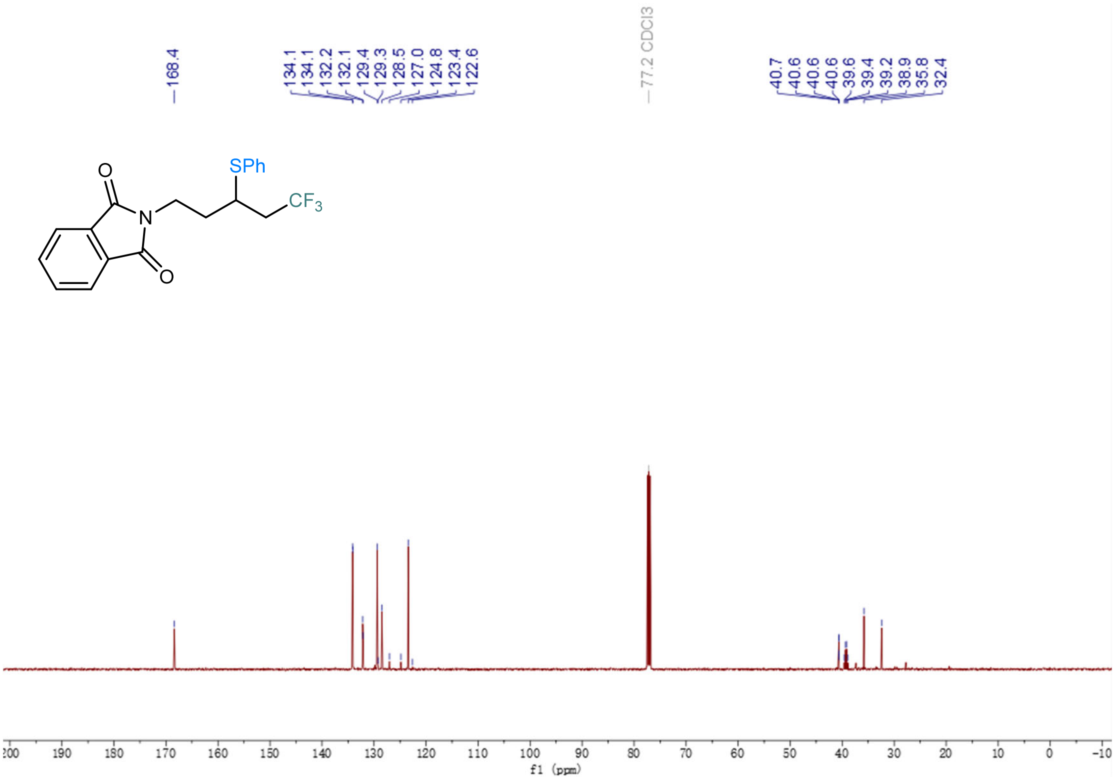

^{13}C NMR spectrum (126 MHz, Chloroform-*d*) of **13**

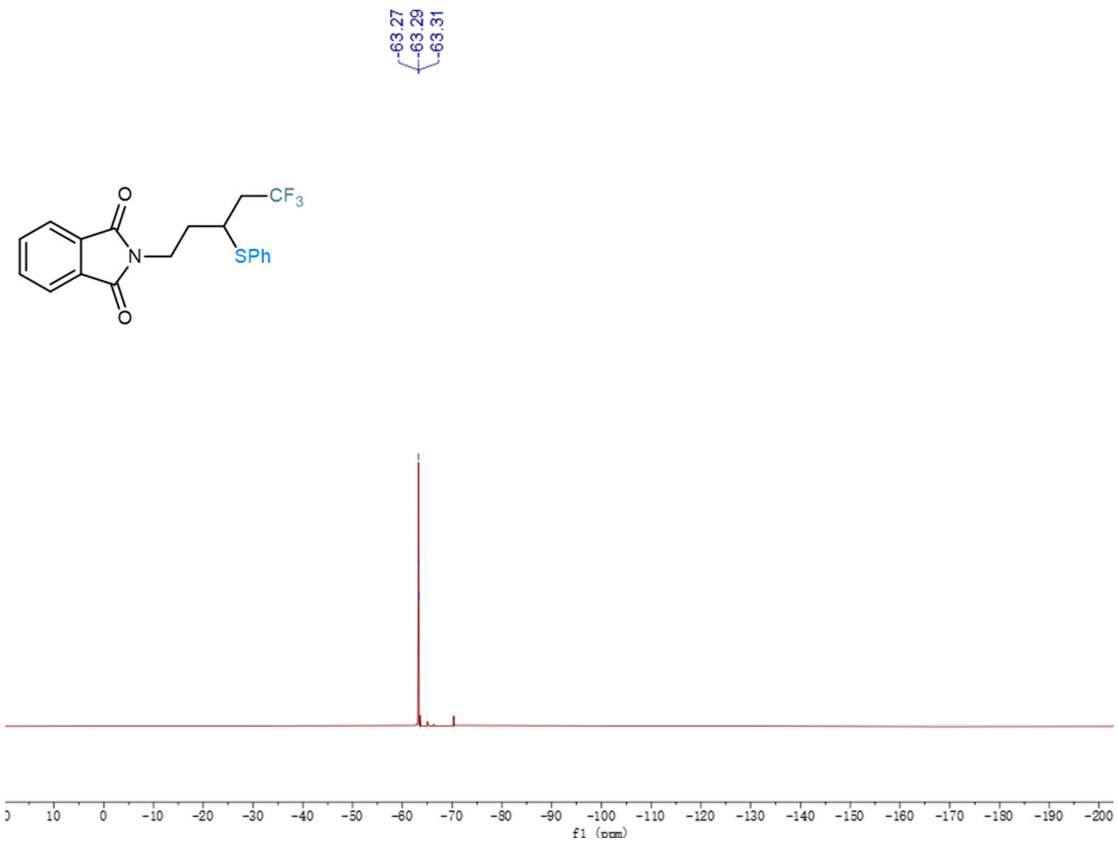

^{19}F NMR spectrum (471 MHz, Chloroform-*d*) of **13**

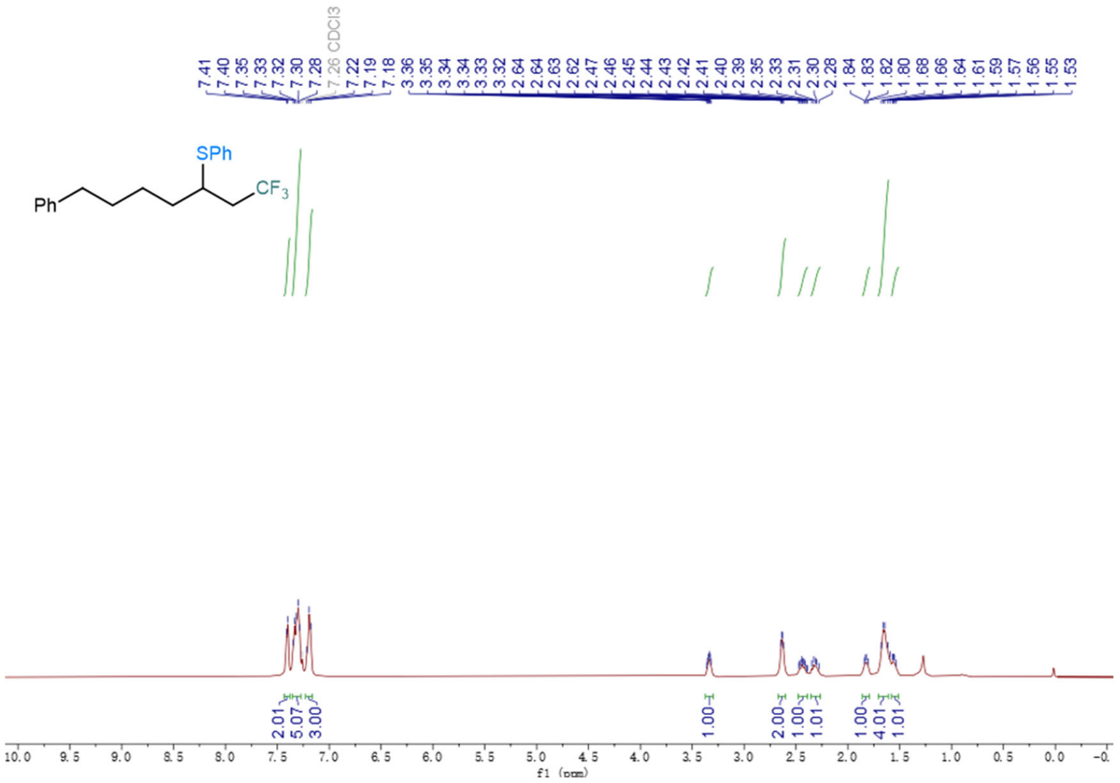

^{1}H NMR spectrum (500 MHz, Chloroform-*d*) of **14**

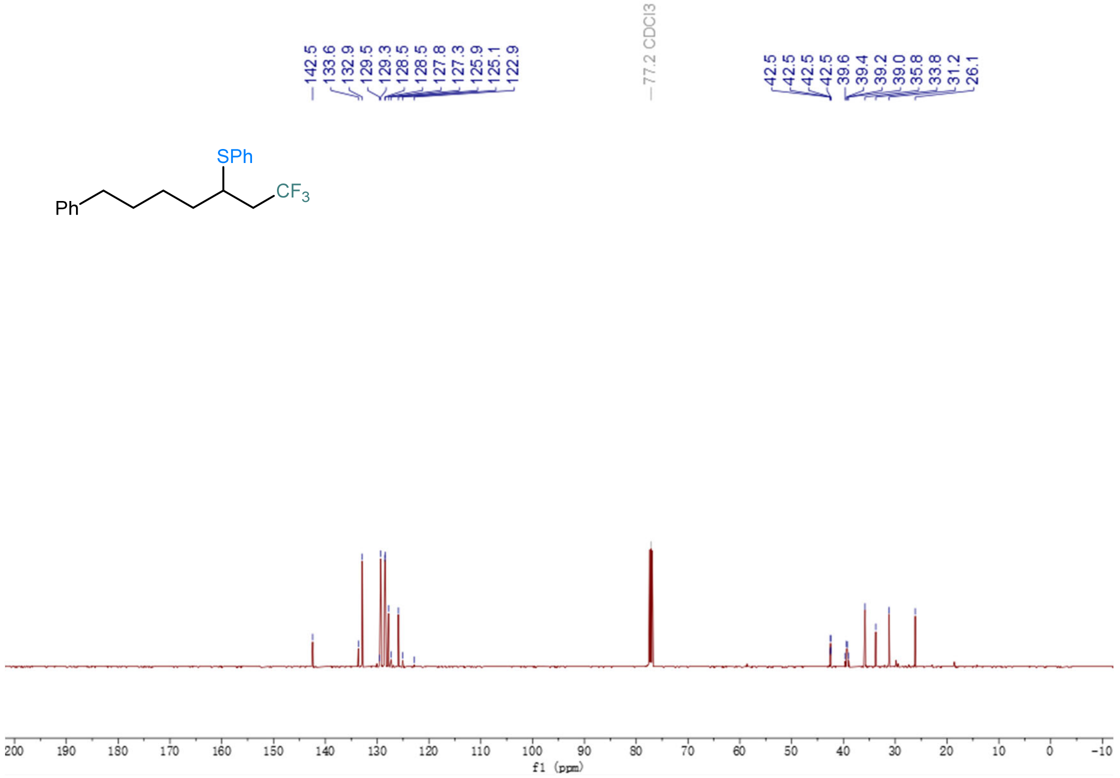

^{13}C NMR spectrum (126 MHz, Chloroform-*d*) of **14**

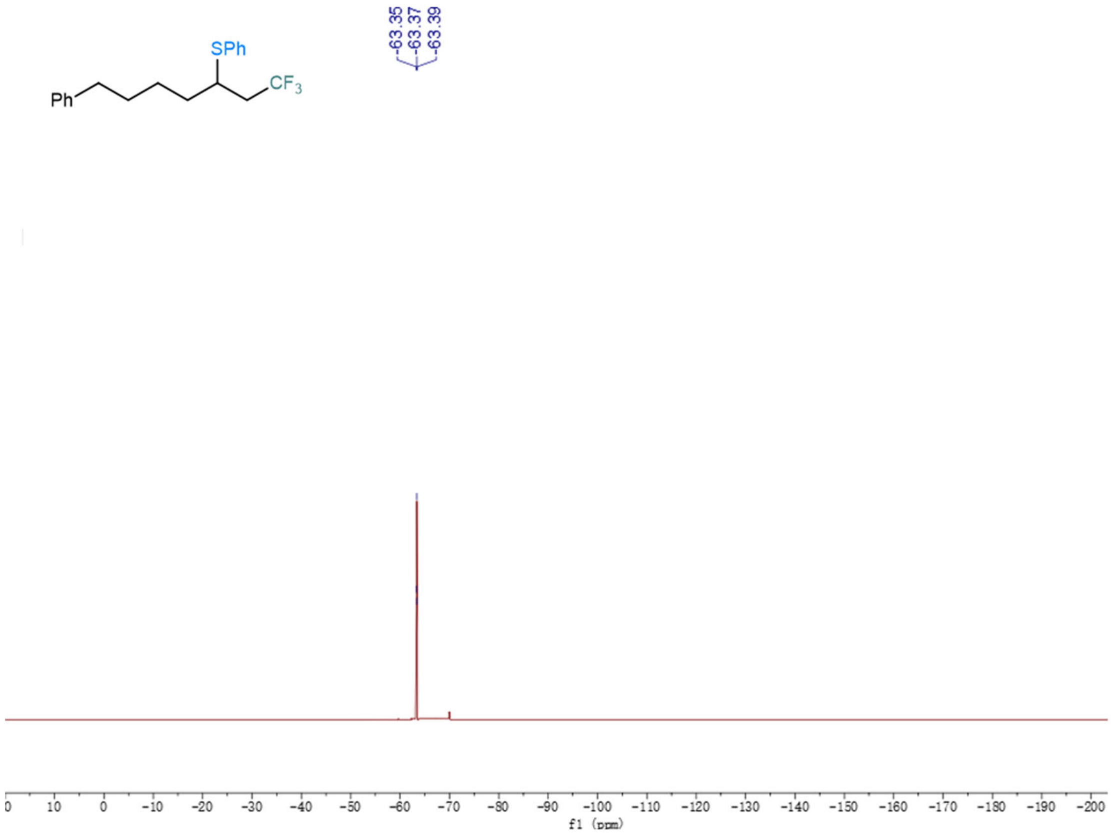

^{19}F NMR spectrum (471 MHz, Chloroform-*d*) of **14**

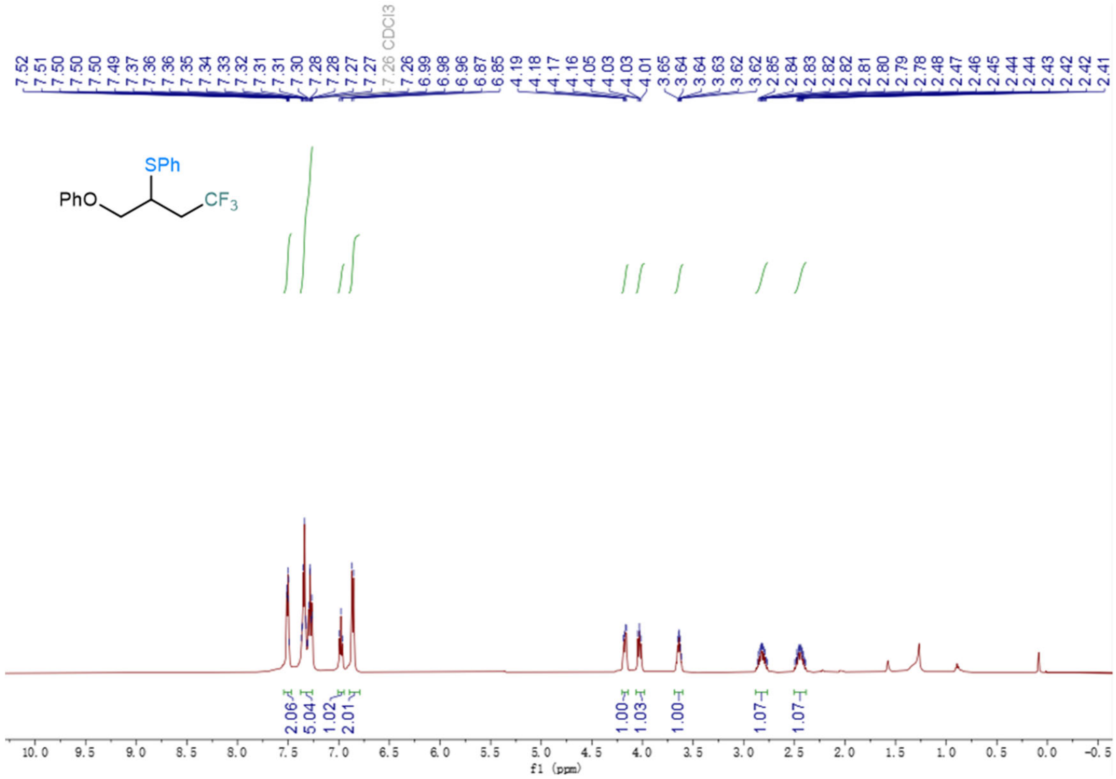

^{1}H NMR spectrum (500 MHz, Chloroform-*d*) of **15**

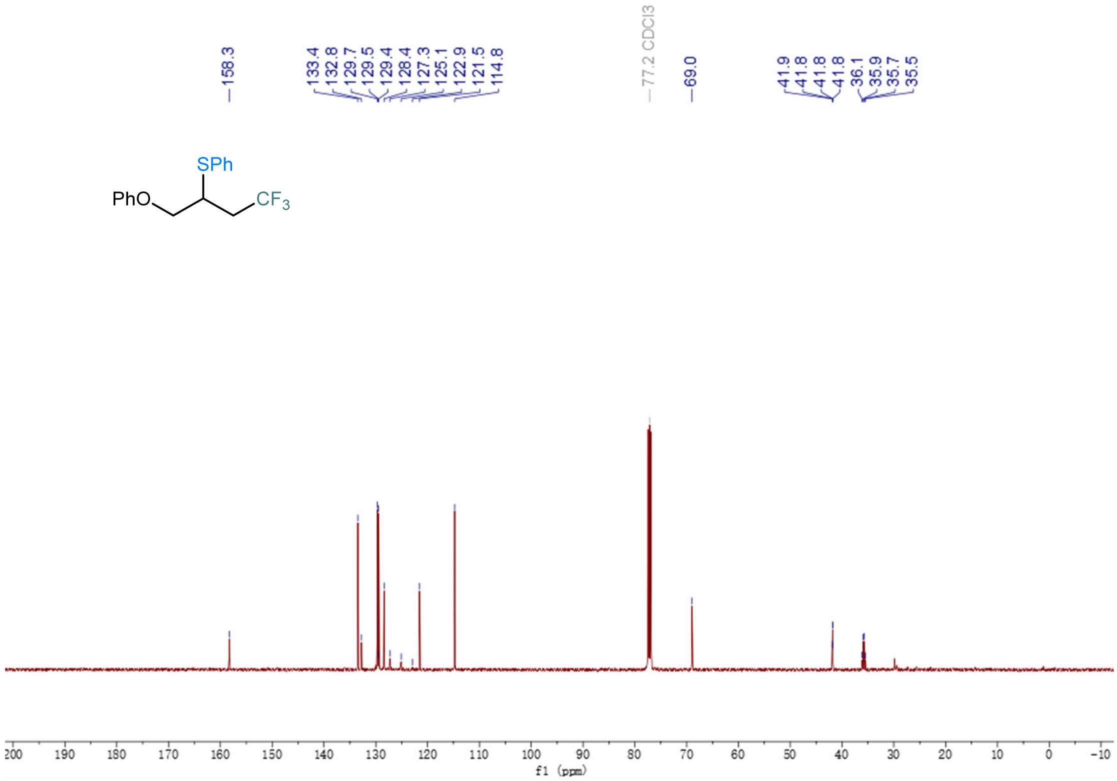

^{13}C NMR spectrum (126 MHz, Chloroform-*d*) of **15**

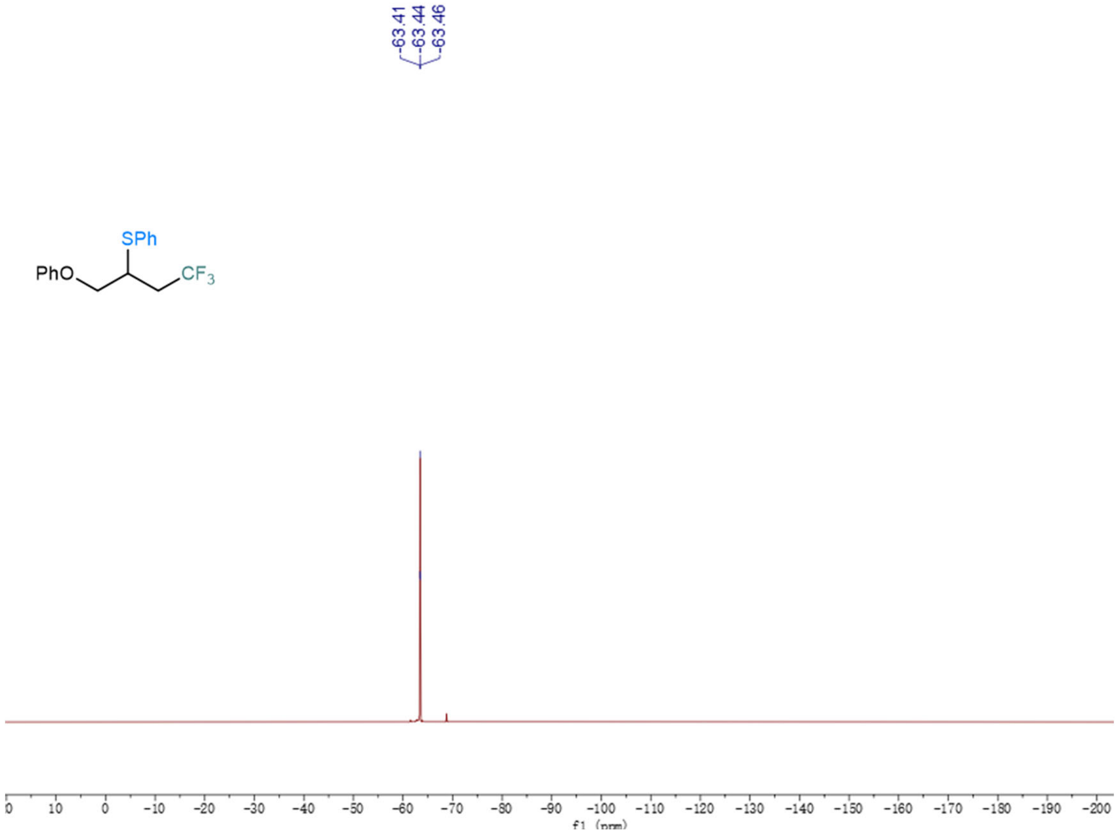

^{19}F NMR spectrum (471 MHz, Chloroform-*d*) of **15**

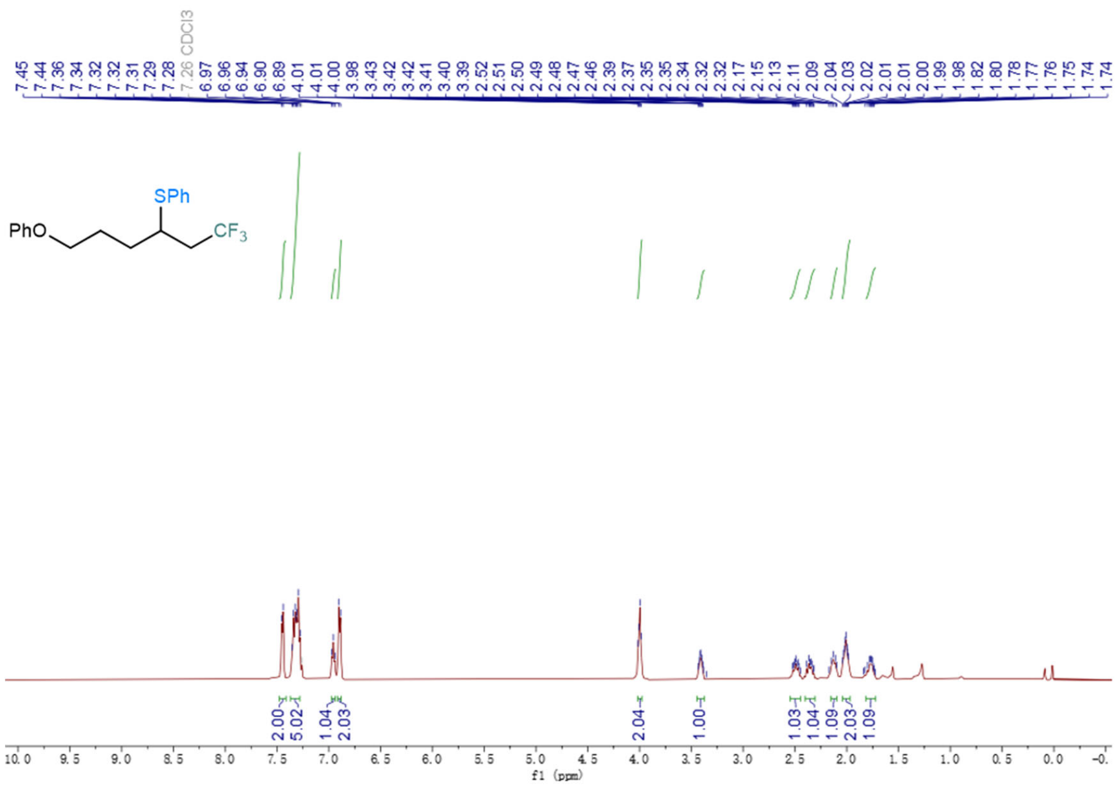

^{1}H NMR spectrum (500 MHz, Chloroform-*d*) of **16**

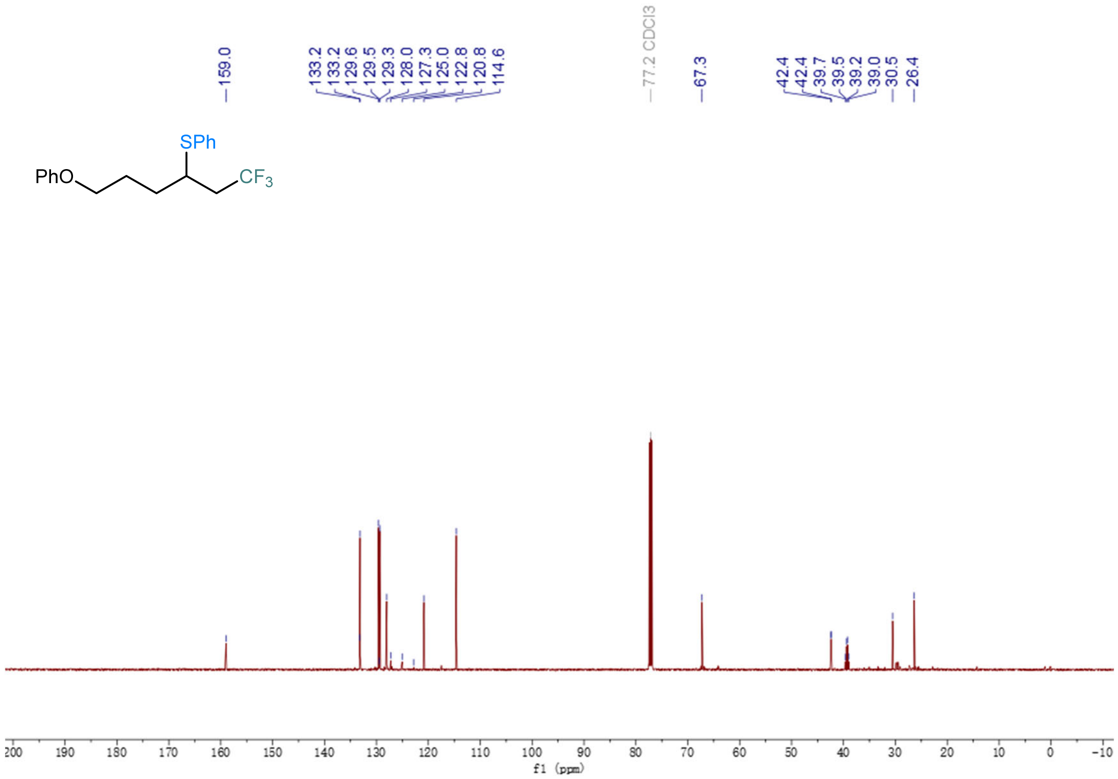

^{13}C NMR spectrum (126 MHz, Chloroform-*d*) of **16**

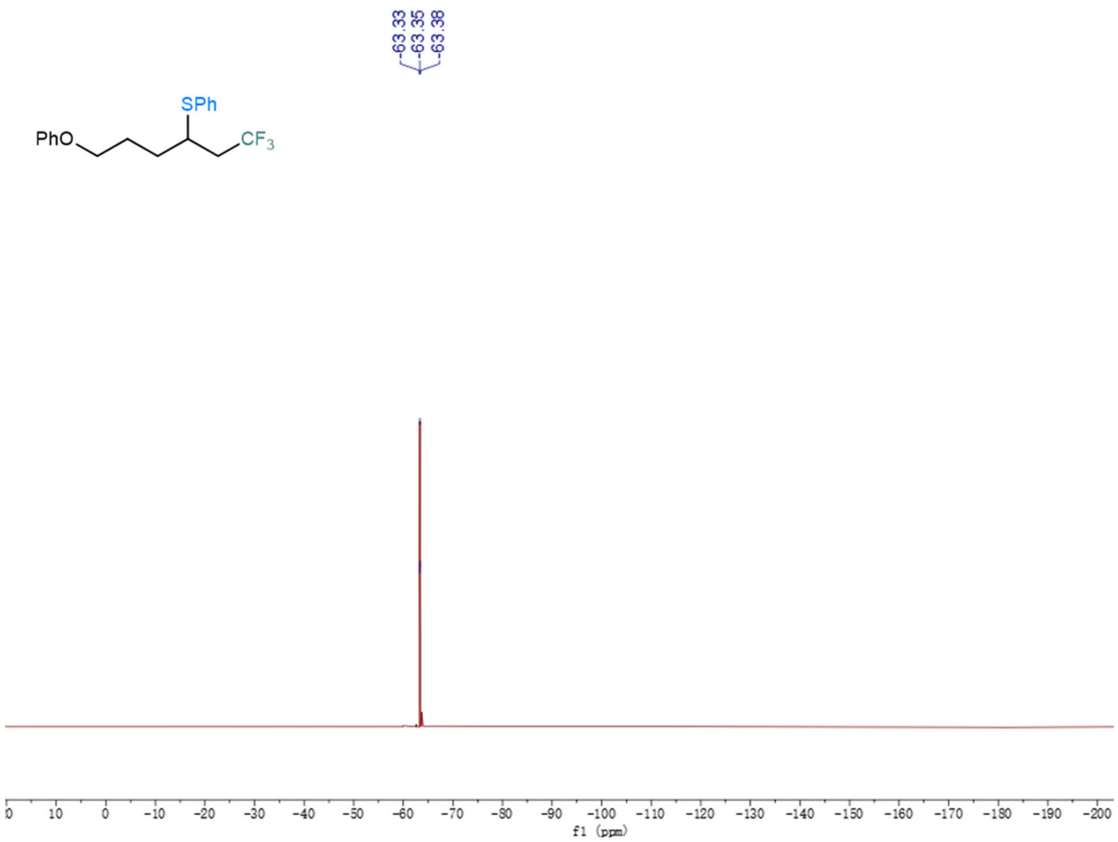

^{19}F NMR spectrum (471 MHz, Chloroform-*d*) of **16**

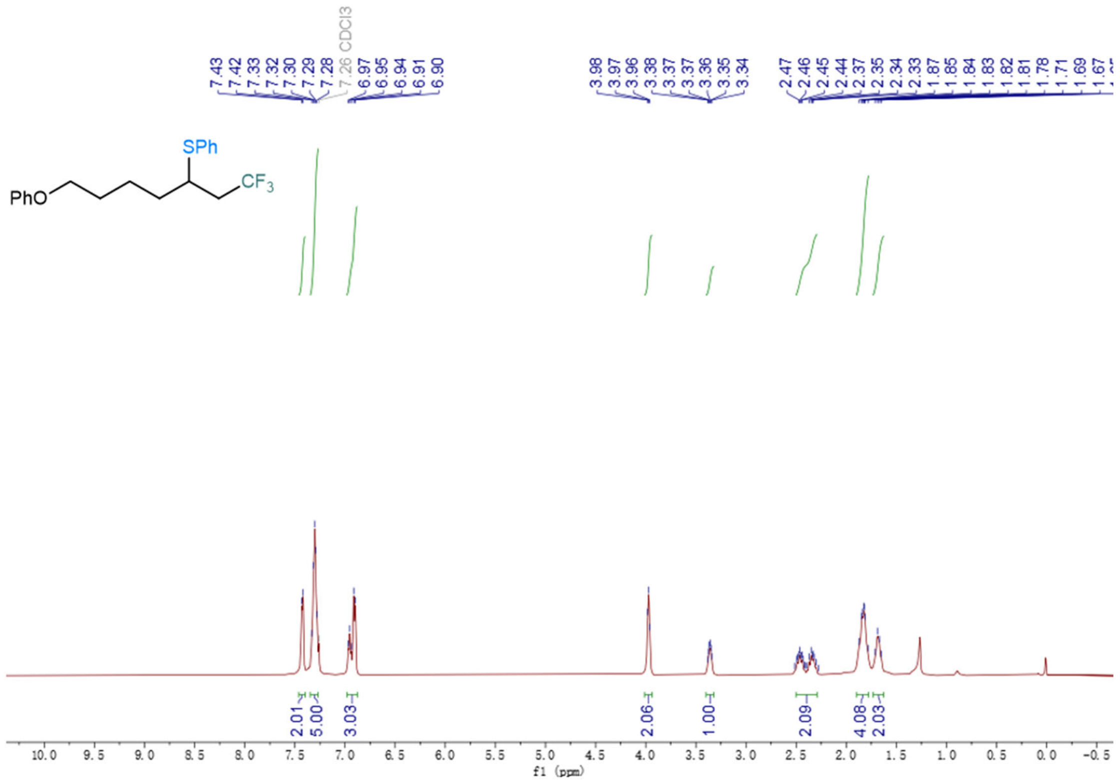

^{1}H NMR spectrum (500 MHz, Chloroform-*d*) of **17**

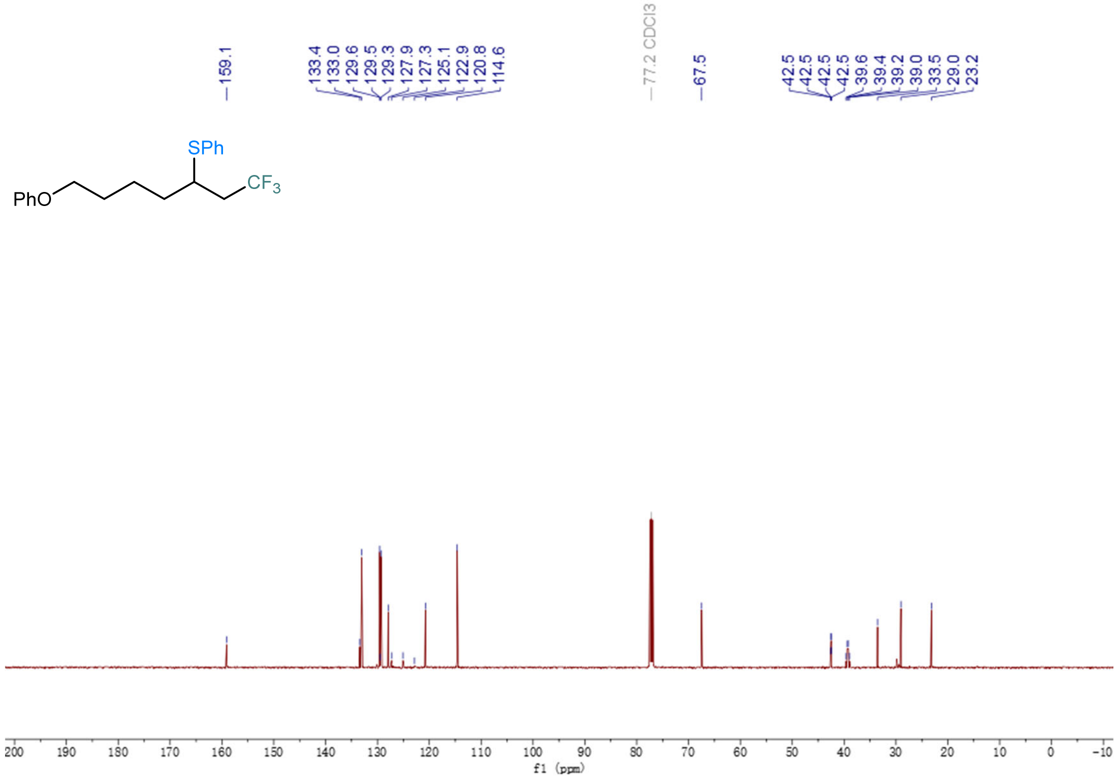

^{13}C NMR spectrum (126 MHz, Chloroform-*d*) of **17**

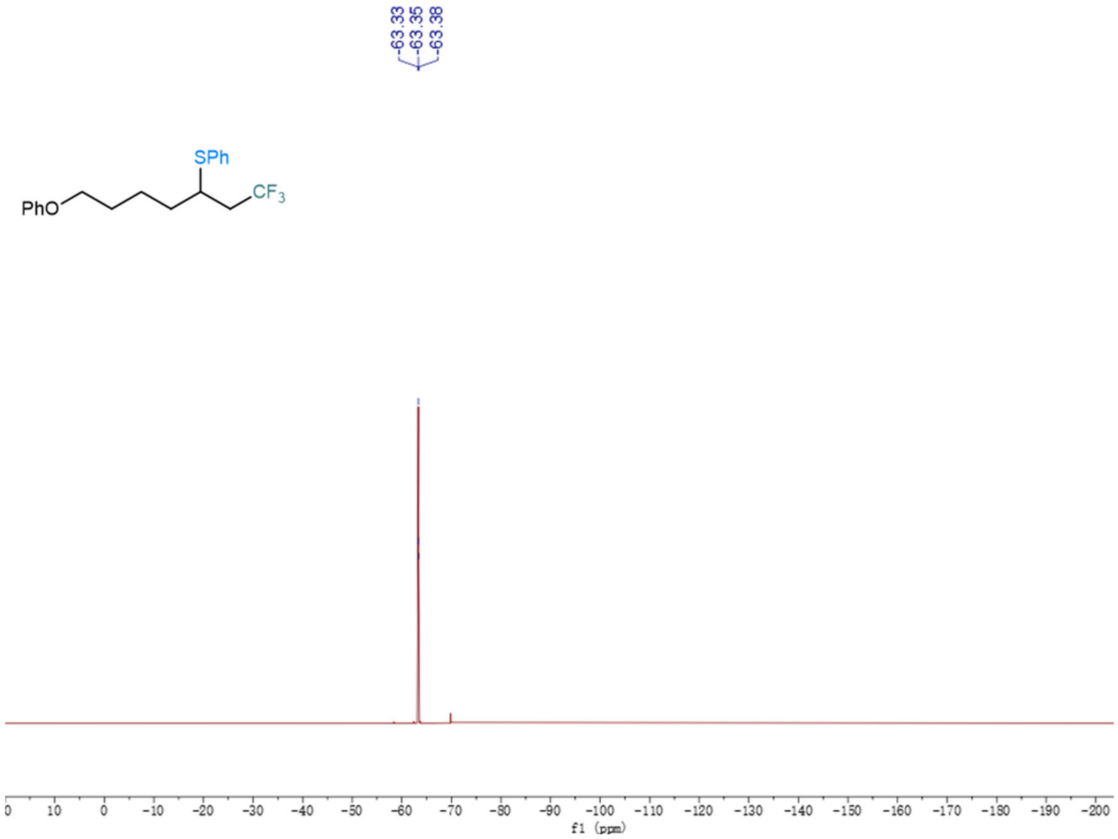

^{19}F NMR spectrum (471 MHz, Chloroform-*d*) of **17**

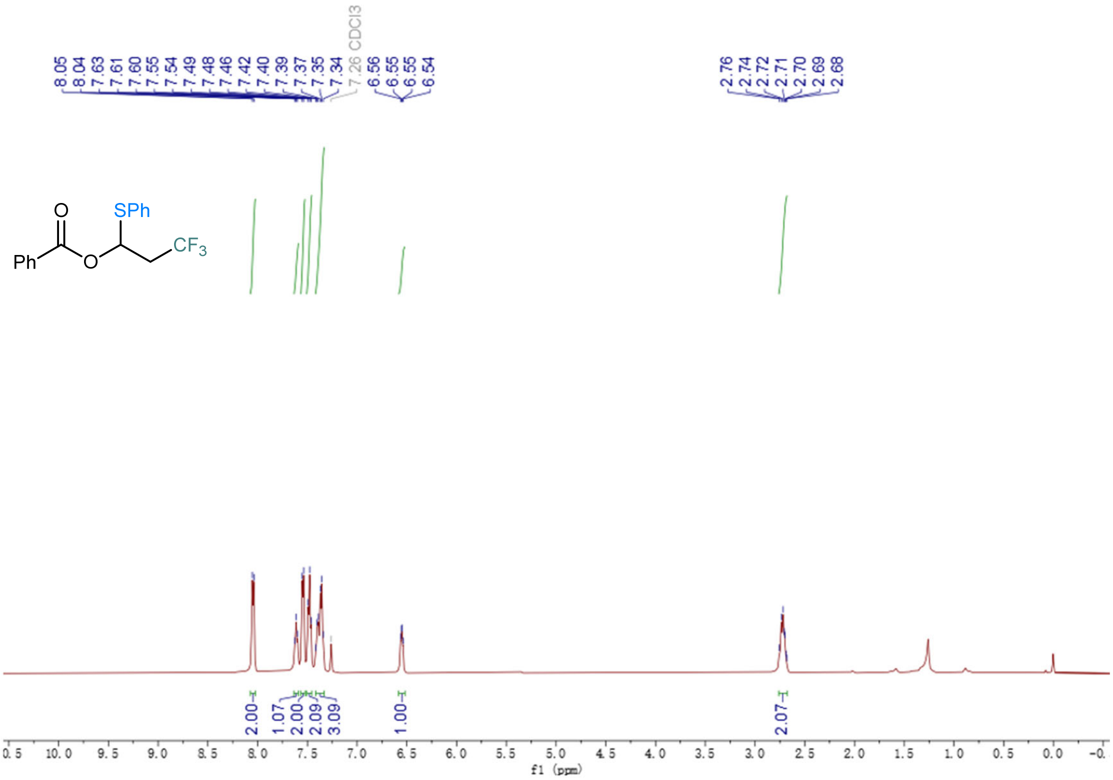

^{1}H NMR spectrum (500 MHz, Chloroform-*d*) of **18**

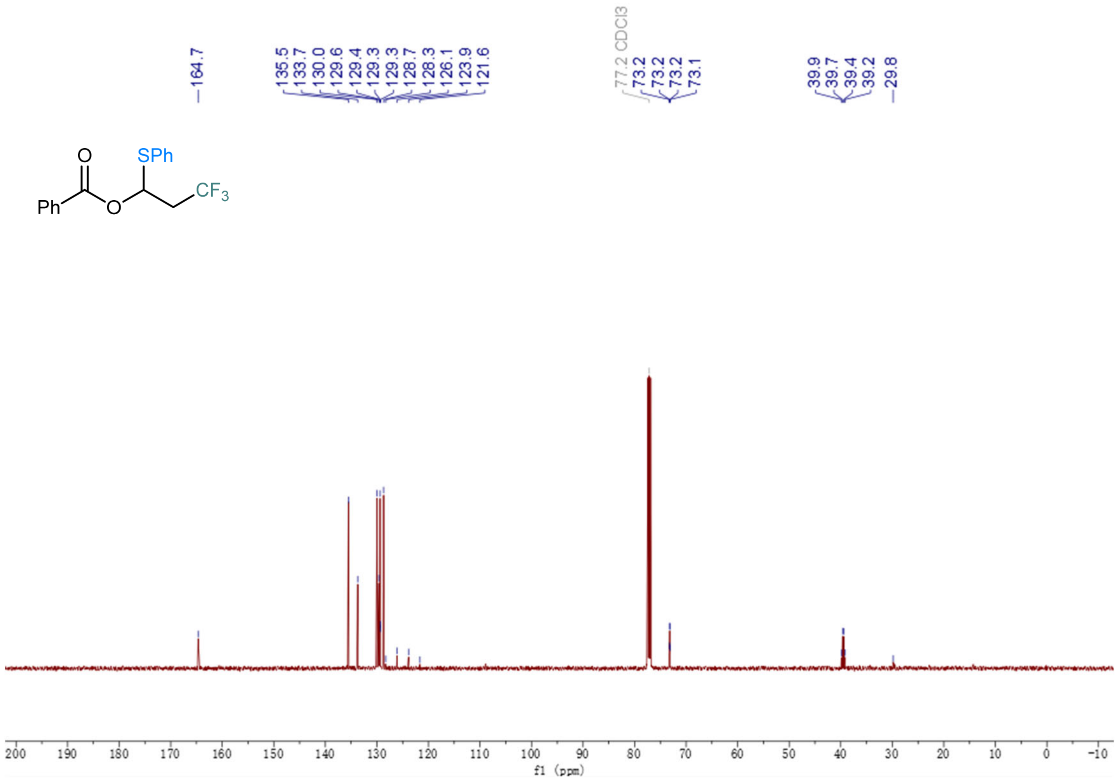

^{13}C NMR spectrum (126 MHz, Chloroform-*d*) of **18**

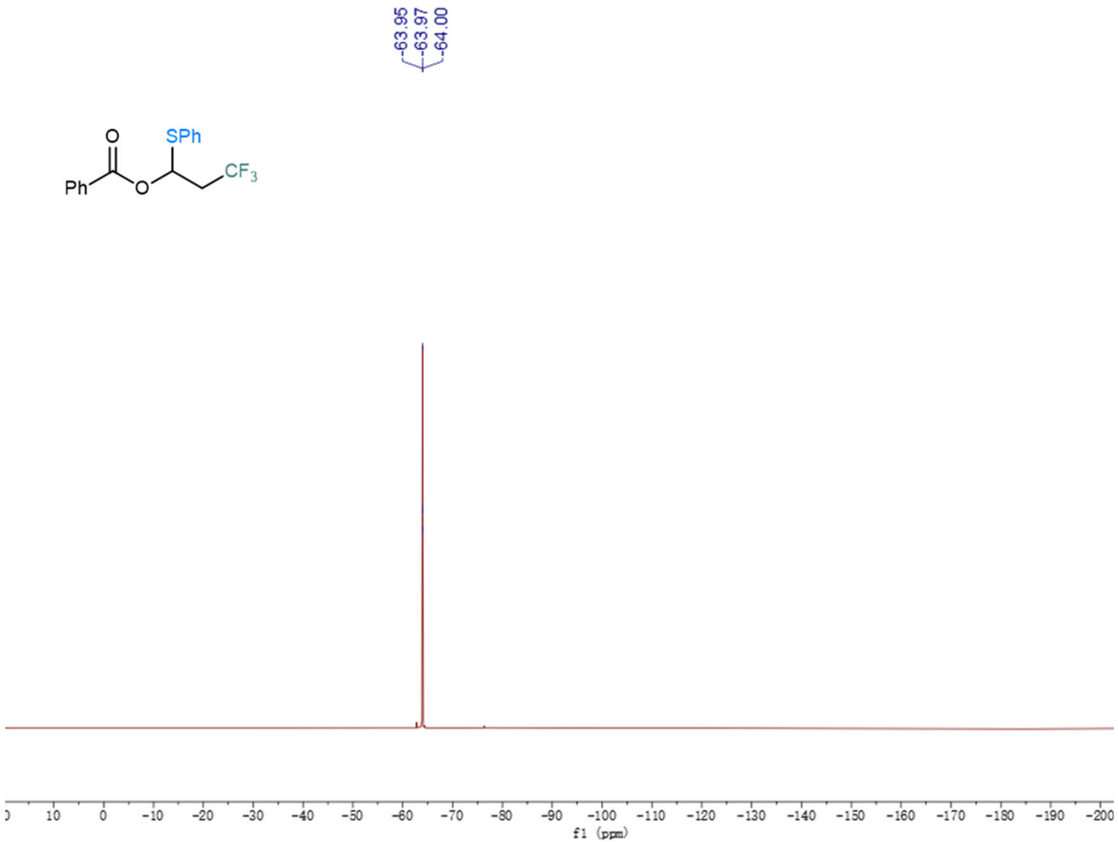

^{19}F NMR spectrum (471 MHz, Chloroform-*d*) of **18**

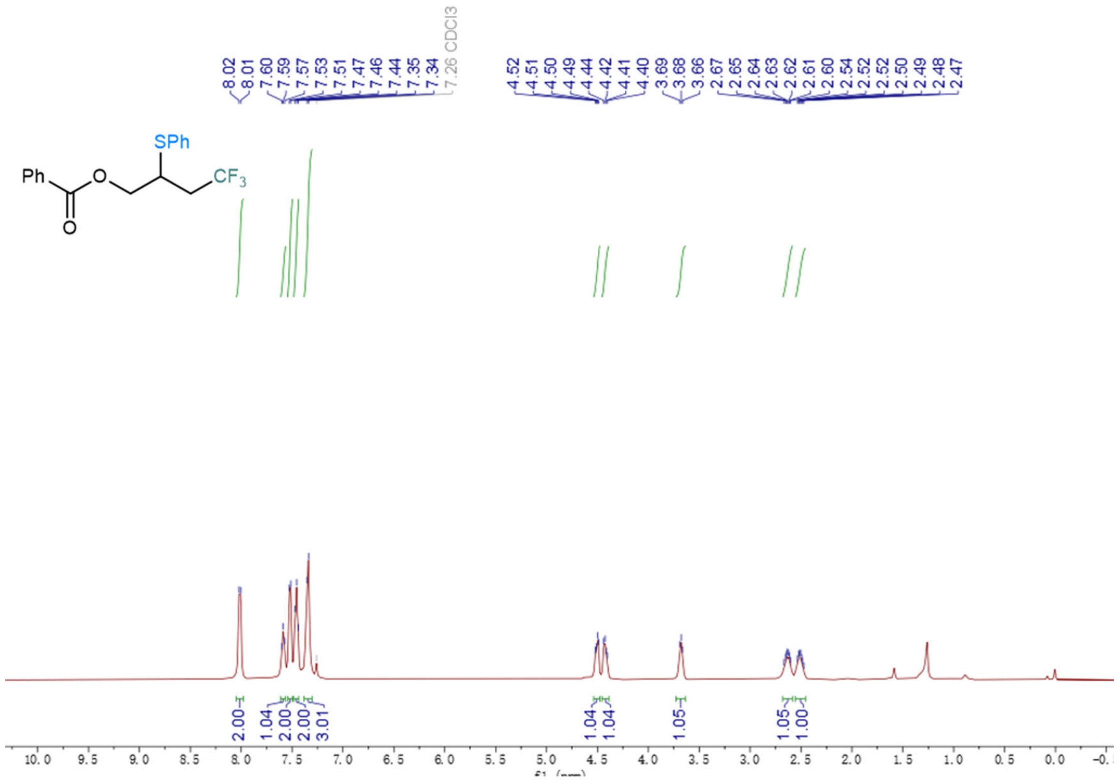

^{1}H NMR spectrum (500 MHz, Chloroform-*d*) of **19**

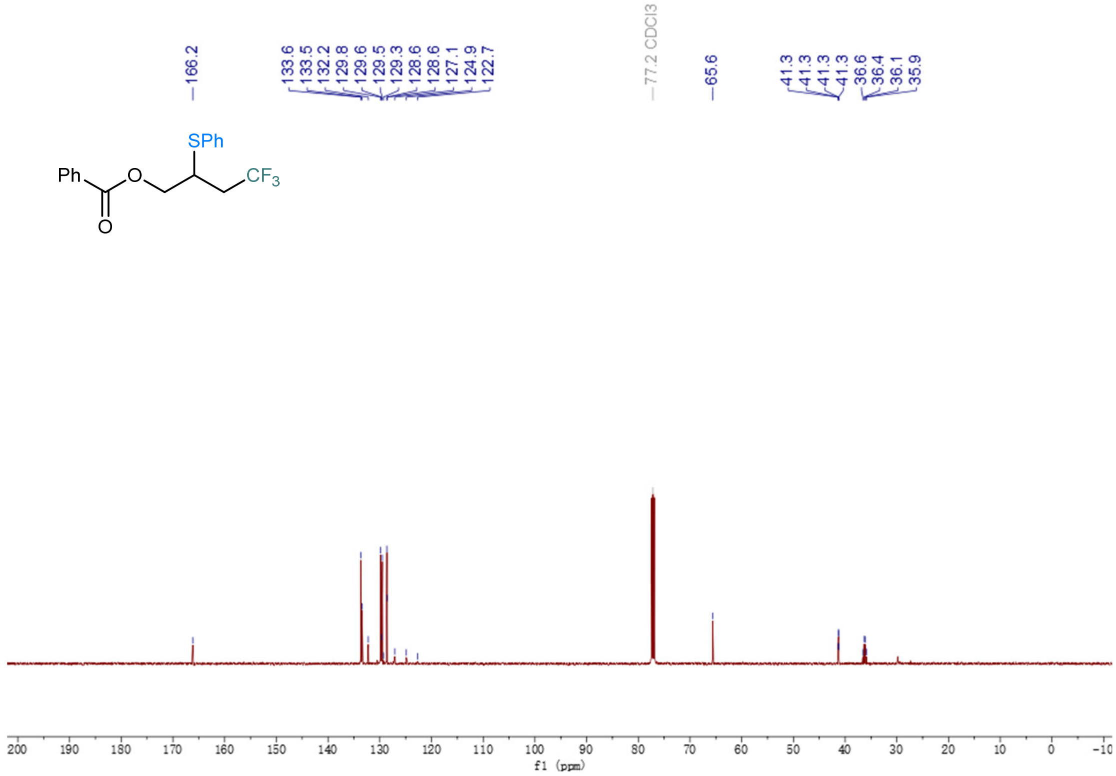

^{13}C NMR spectrum (126 MHz, Chloroform-*d*) of **19**

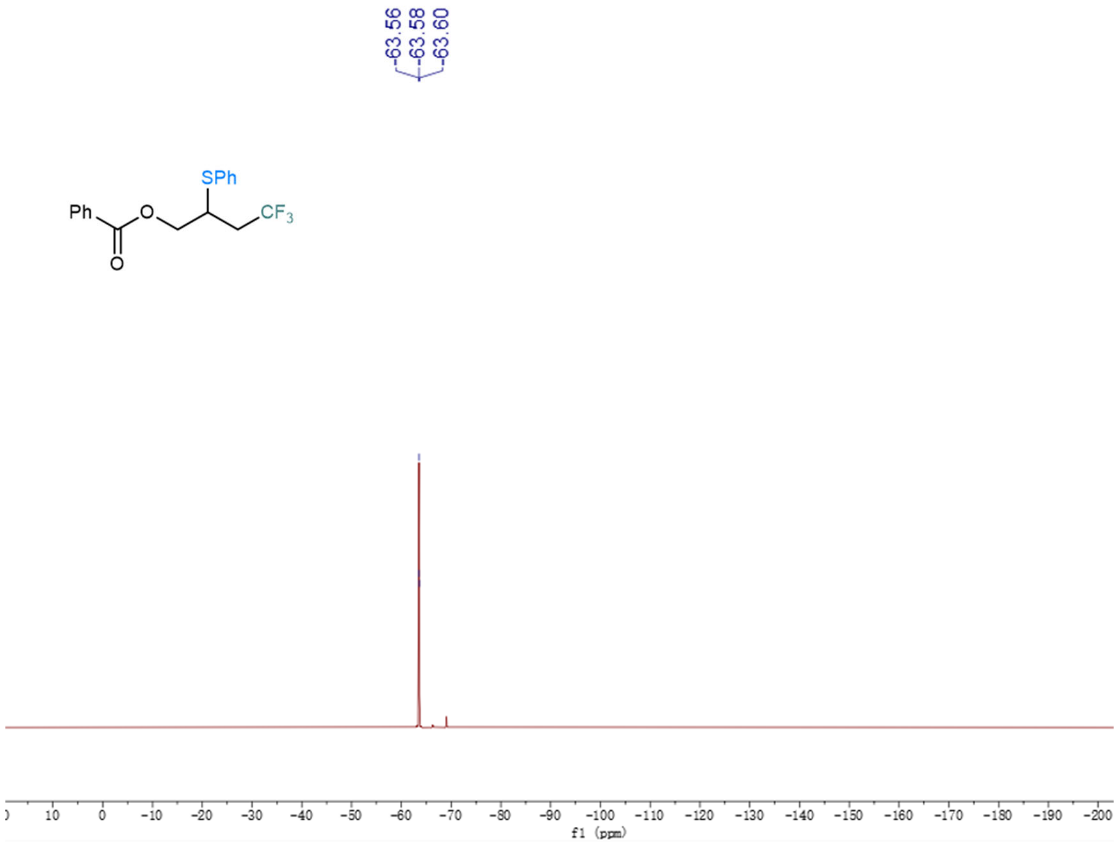

^{19}F NMR spectrum (471 MHz, Chloroform-*d*) of **19**

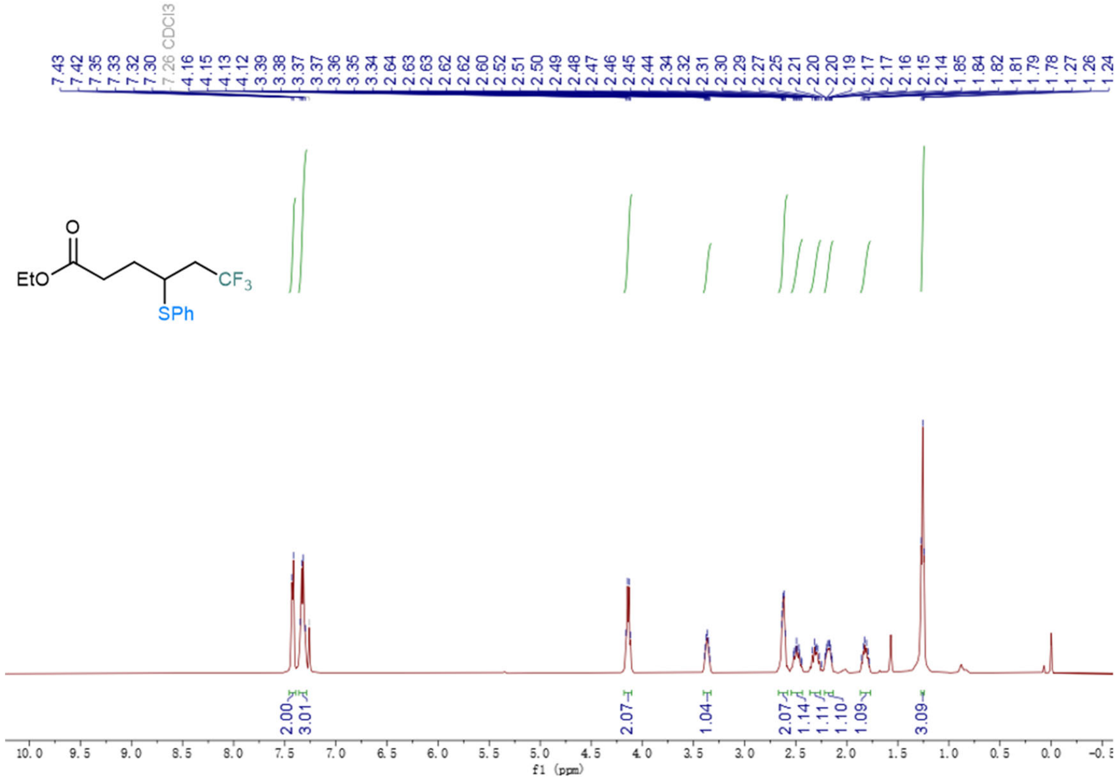

^{1}H NMR spectrum (500 MHz, Chloroform-*d*) of **20**

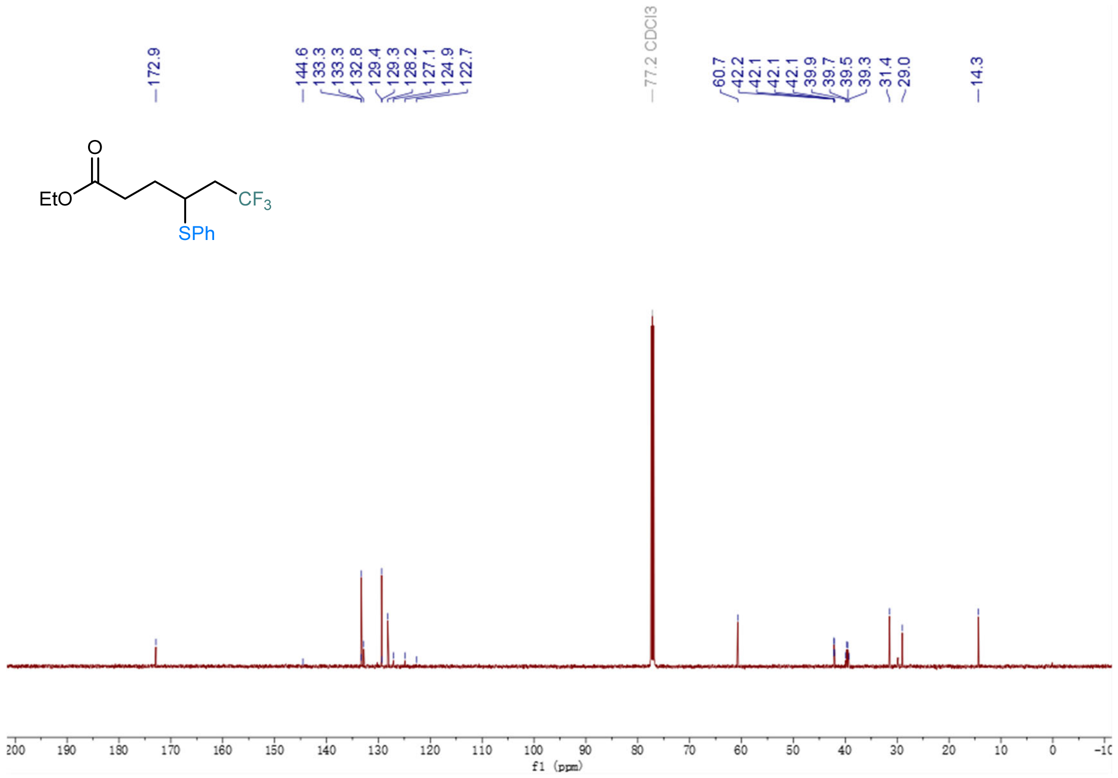

^{13}C NMR spectrum (126 MHz, Chloroform-*d*) of **20**

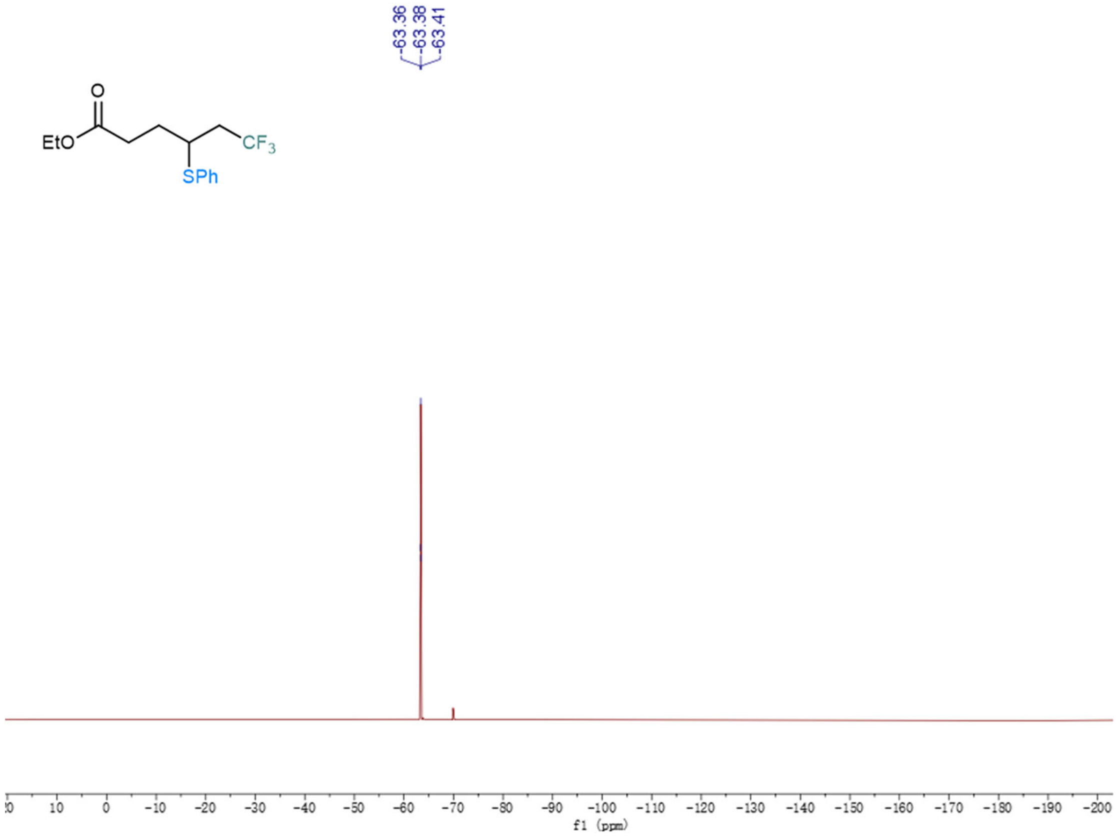

^{19}F NMR spectrum (471 MHz, Chloroform-*d*) of **20**

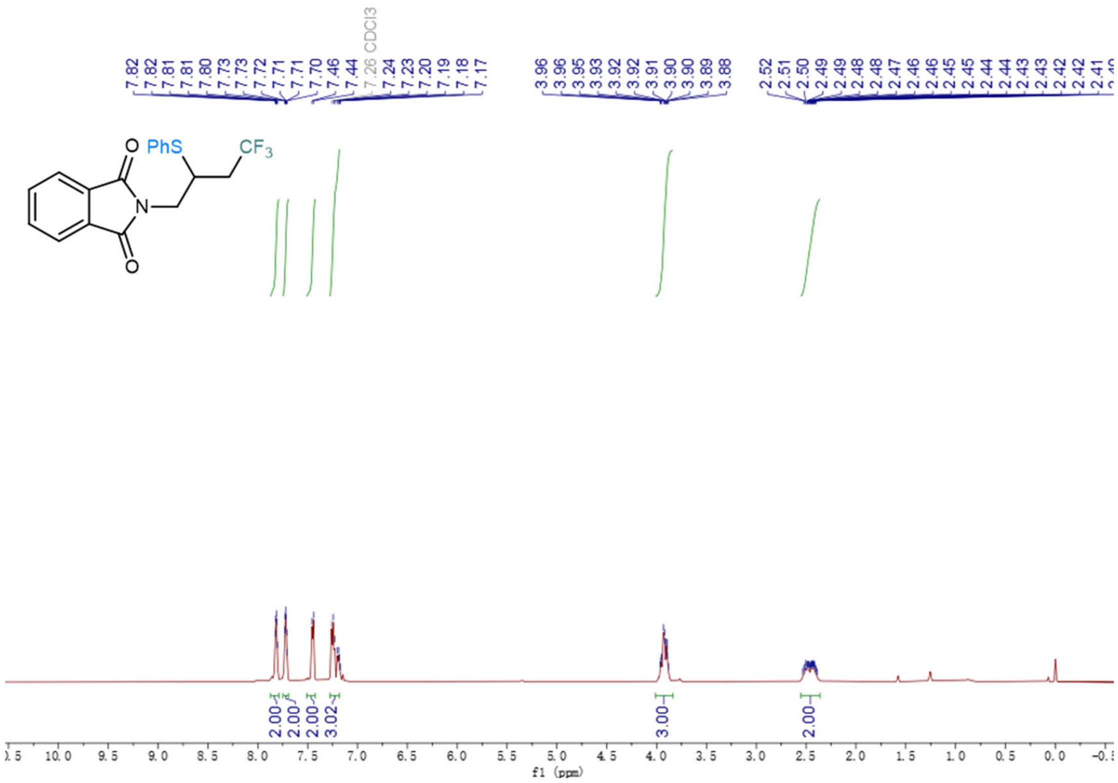

^{1}H NMR spectrum (500 MHz, Chloroform-*d*) of **21**

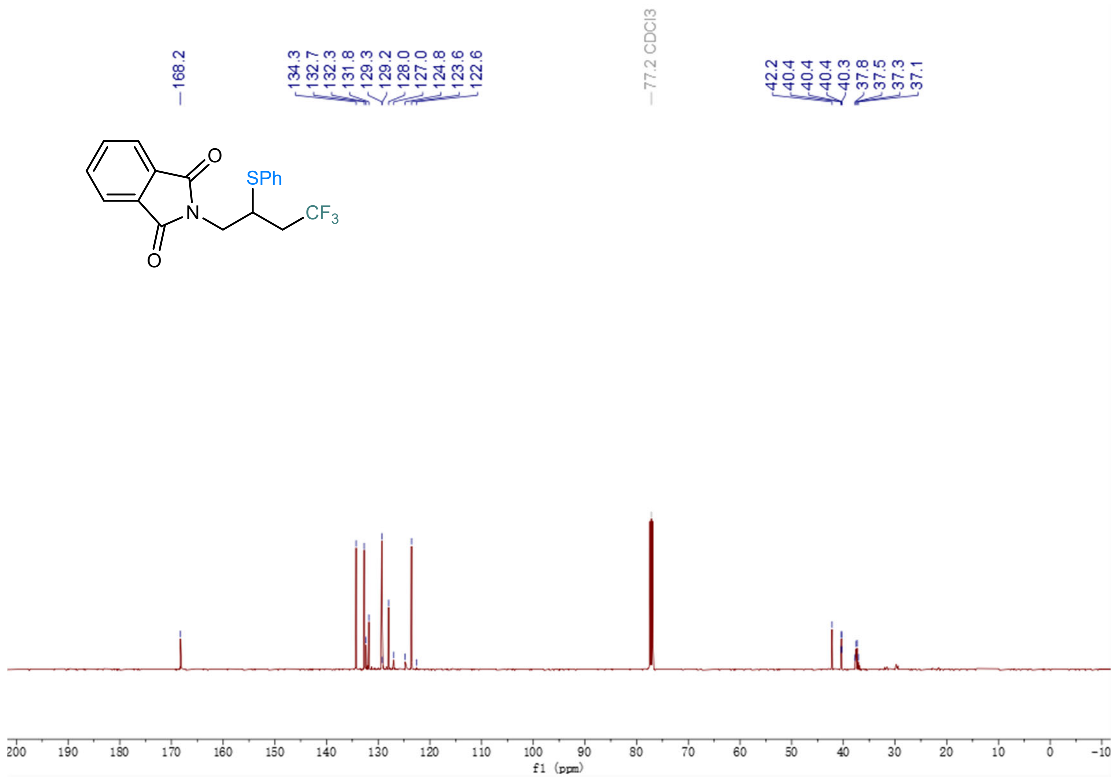

^{13}C NMR spectrum (126 MHz, Chloroform-*d*) of **21**

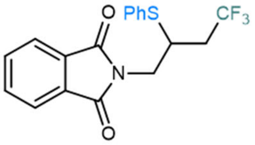

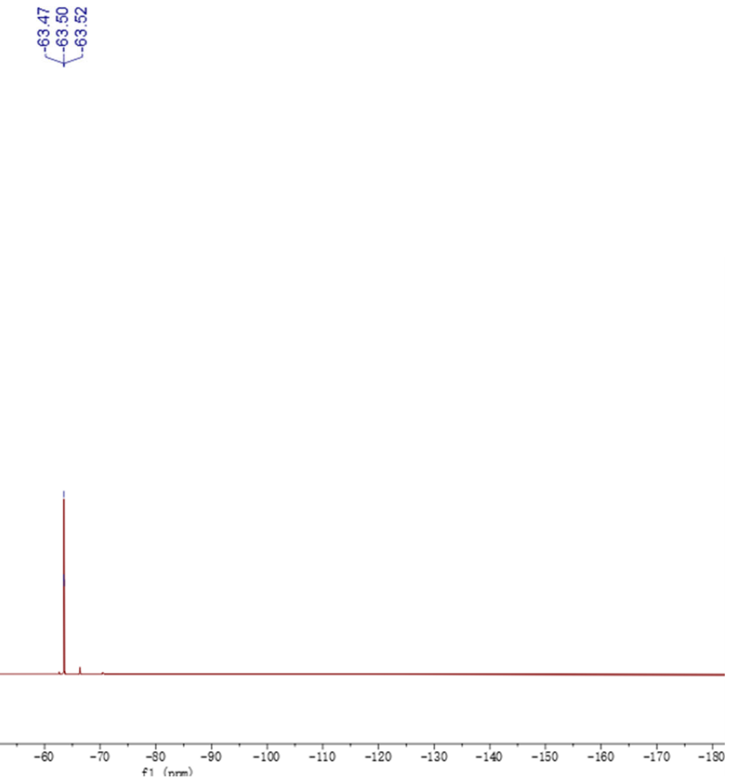

^{19}F NMR spectrum (471 MHz, Chloroform-*d*) of **21**

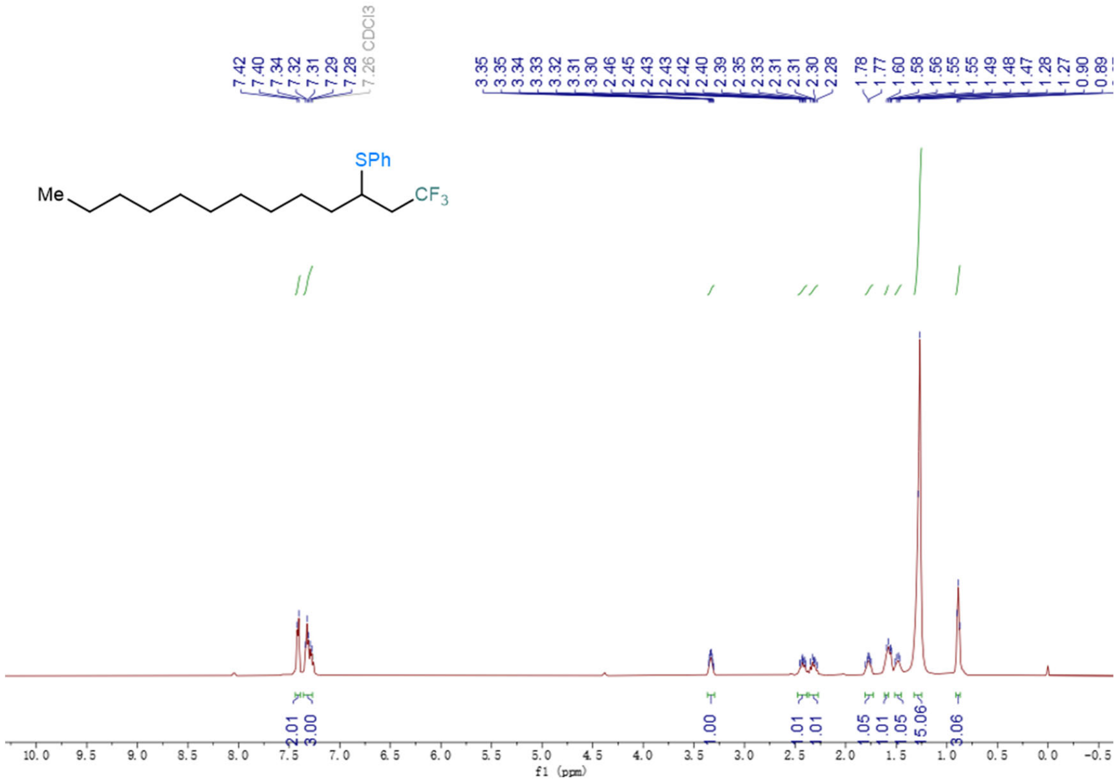

^{1}H NMR spectrum (500 MHz, Chloroform-*d*) of **22**

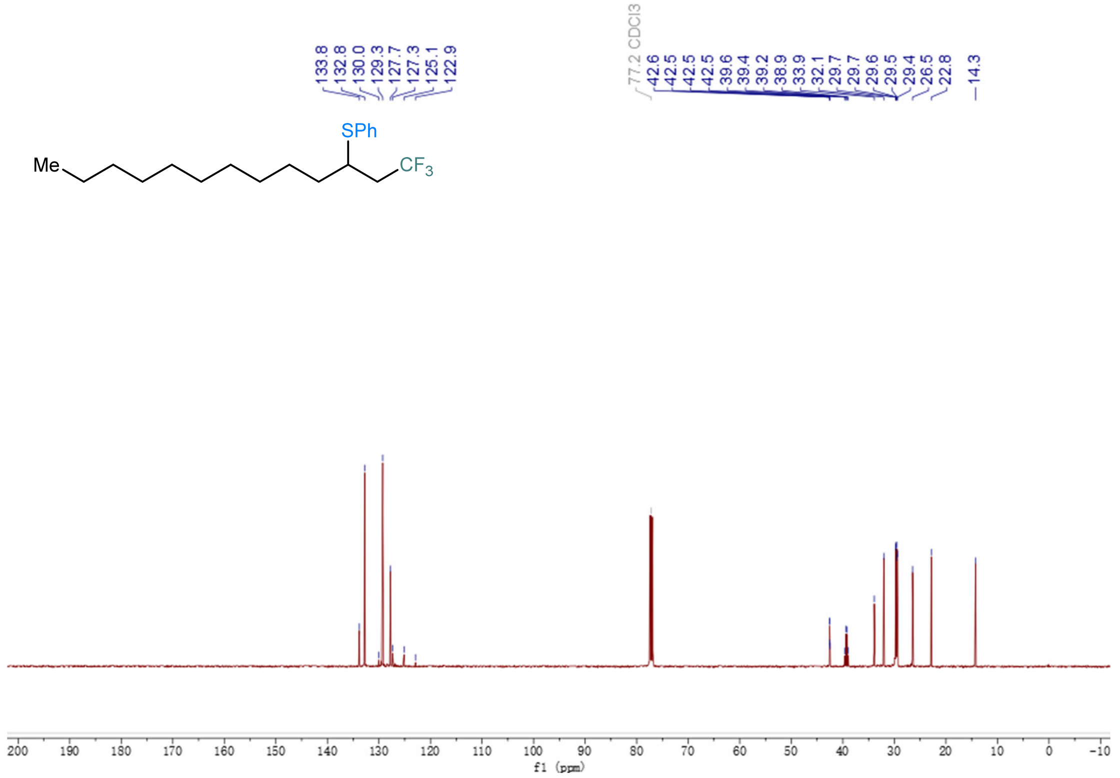

^{13}C NMR spectrum (126 MHz, Chloroform-*d*) of **22**

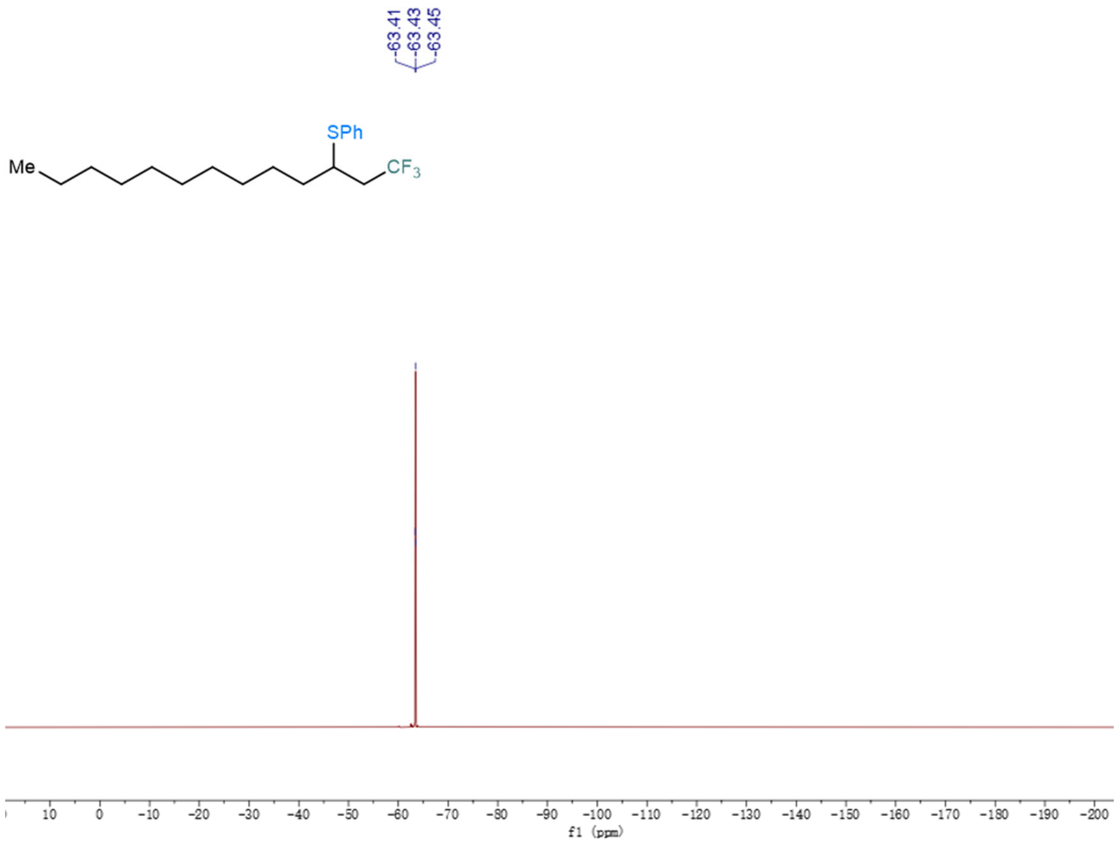

^{19}F NMR spectrum (471 MHz, Chloroform-*d*) of **22**

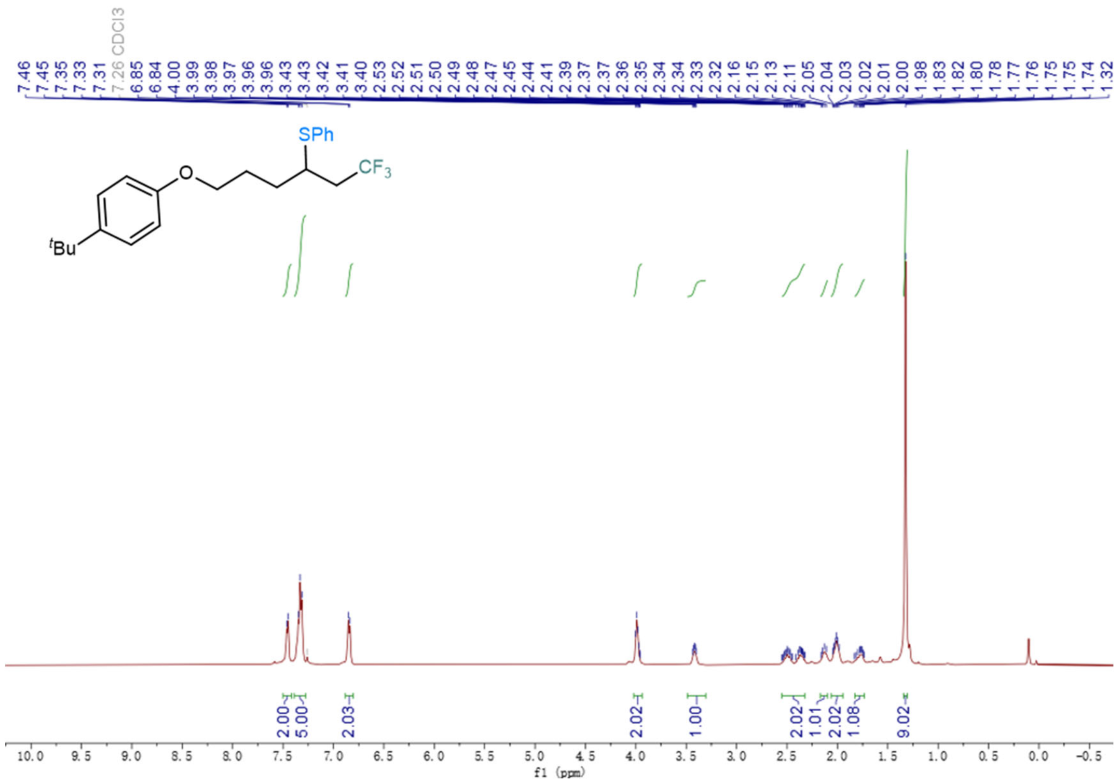

^{1}H NMR spectrum (500 MHz, Chloroform-*d*) of **23**

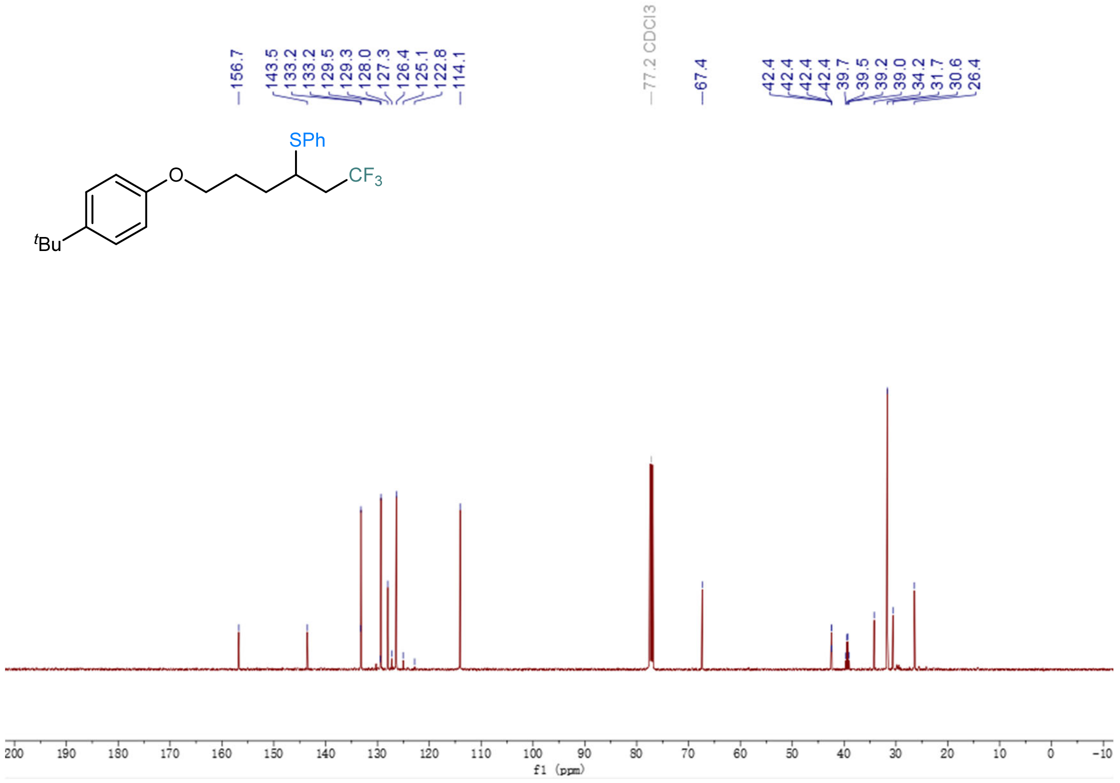

^{13}C NMR spectrum (126 MHz, Chloroform-*d*) of **23**

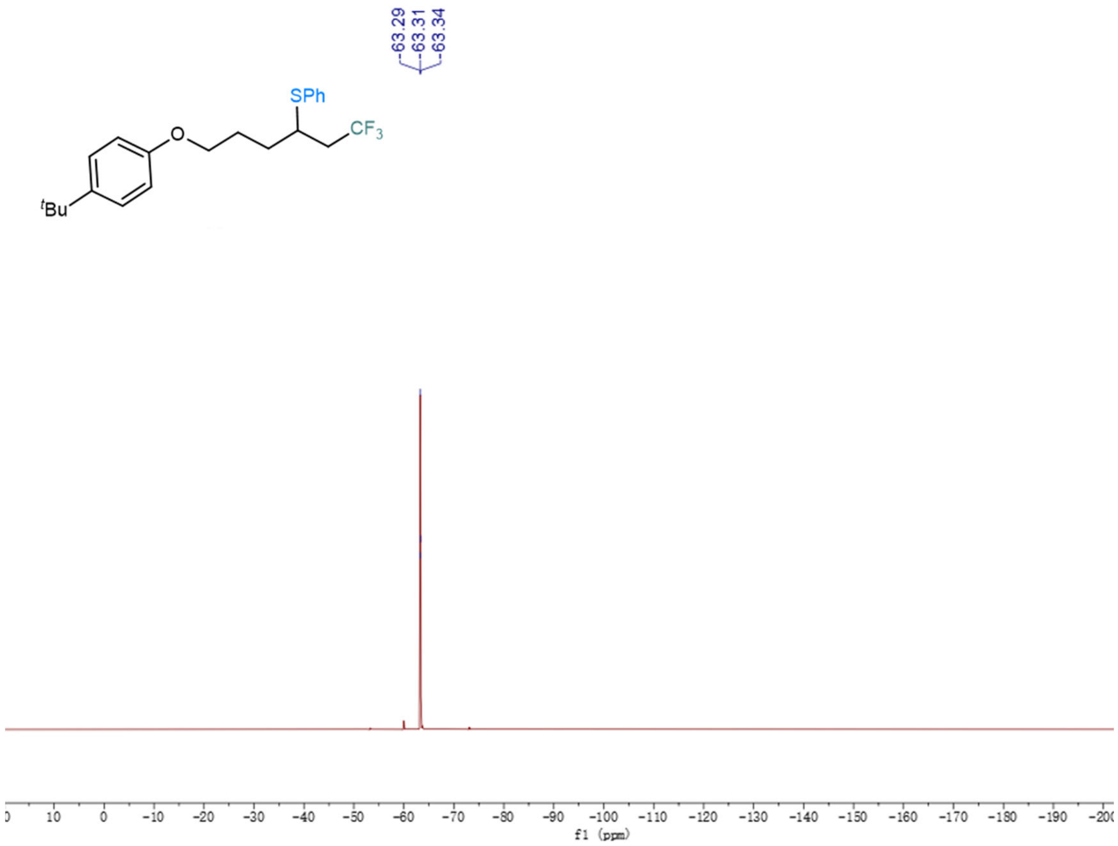

^{19}F NMR spectrum (471 MHz, Chloroform-*d*) of **23**

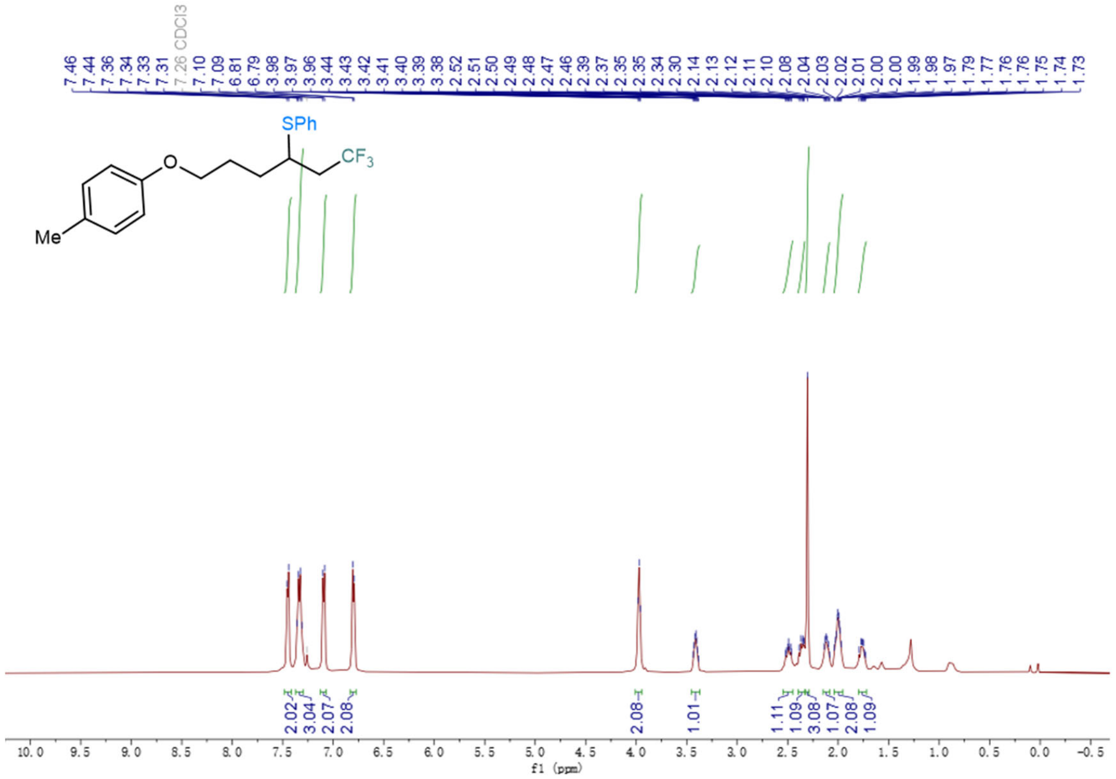

^{1}H NMR spectrum (500 MHz, Chloroform-*d*) of **24**

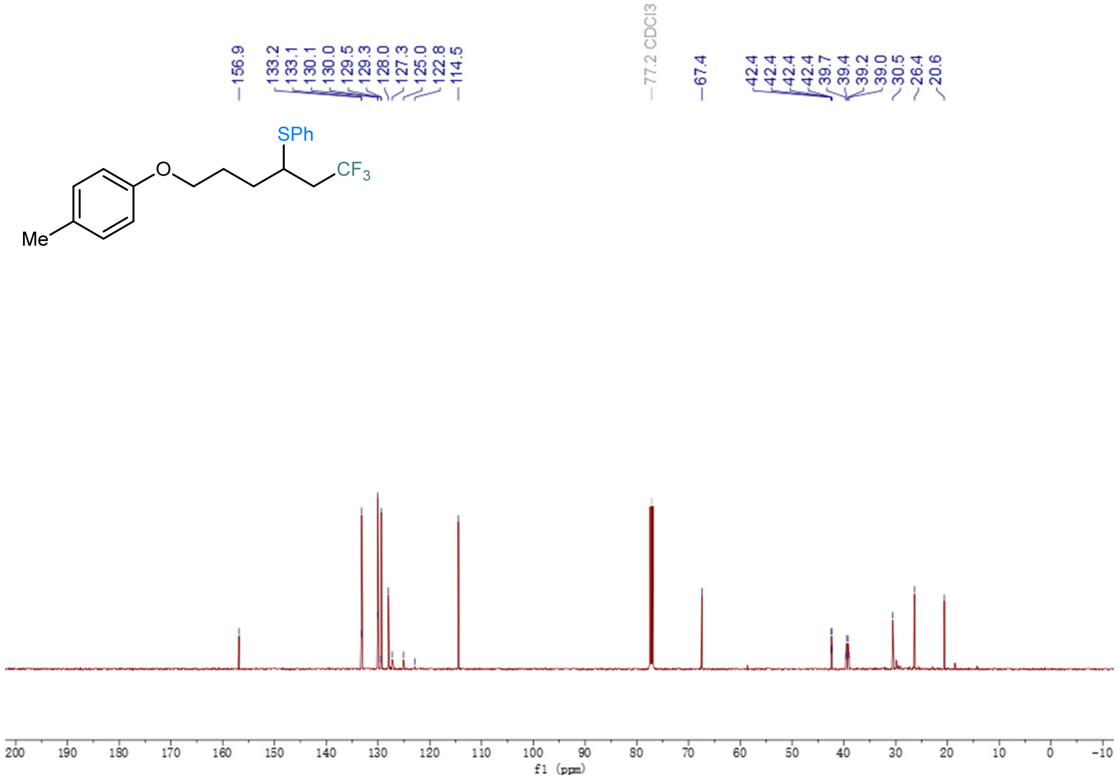

^{13}C NMR spectrum (126 MHz, Chloroform-*d*) of **24**

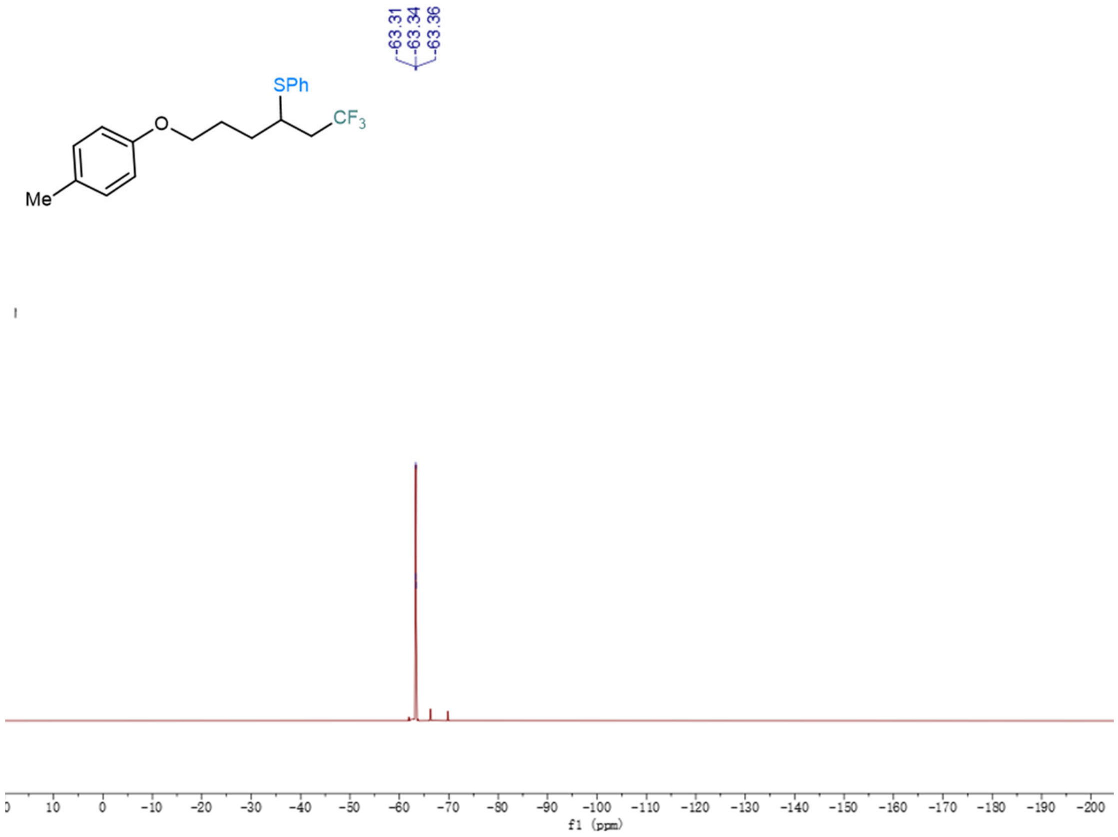

^{19}F NMR spectrum (471 MHz, Chloroform-*d*) of **24**

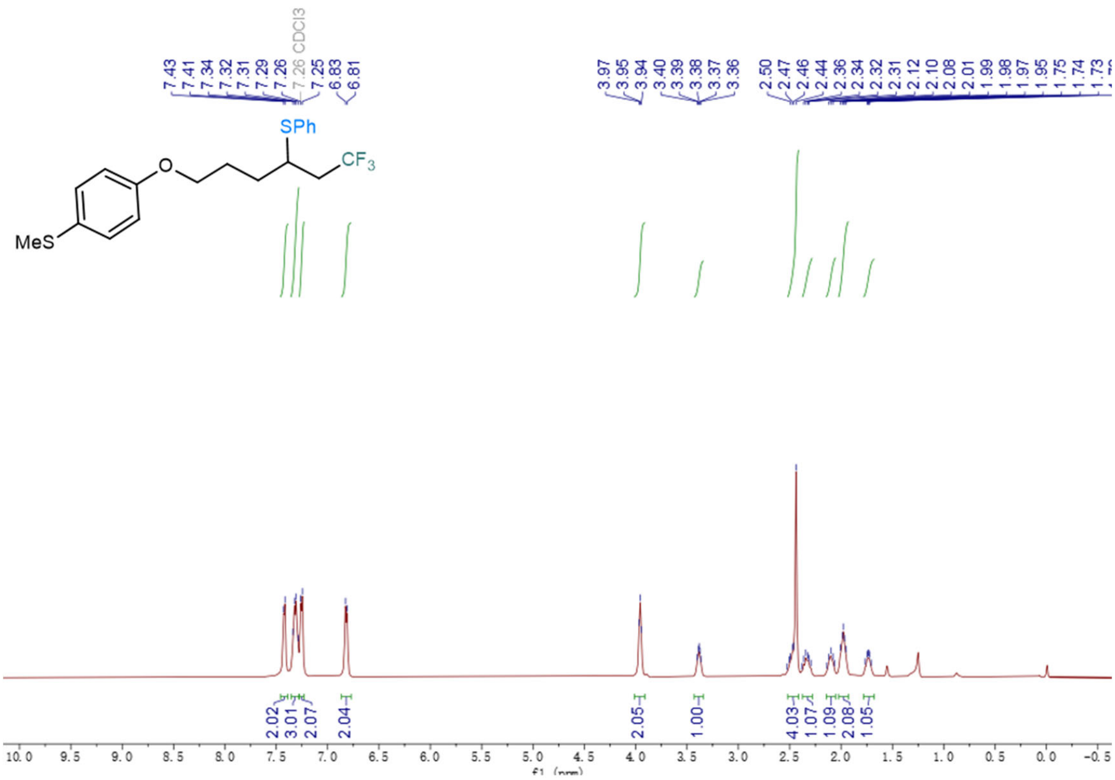

^{1}H NMR spectrum (500 MHz, Chloroform-*d*) of **25**

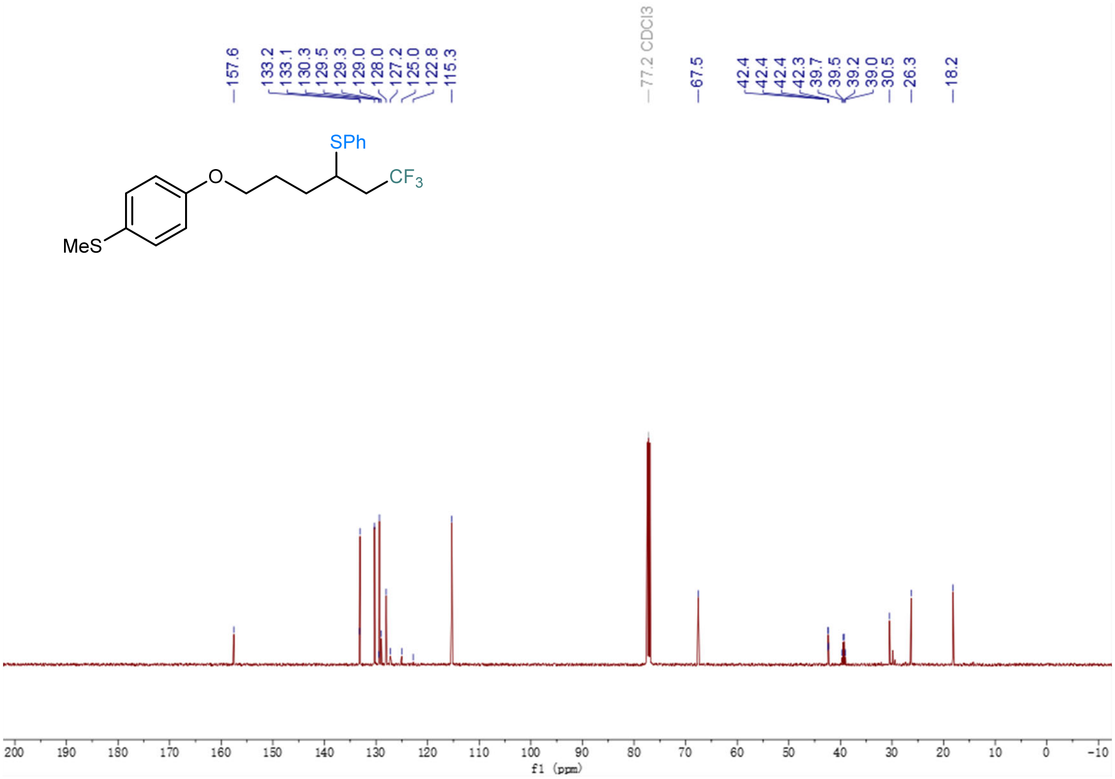

^{13}C NMR spectrum (126 MHz, Chloroform-*d*) of **25**

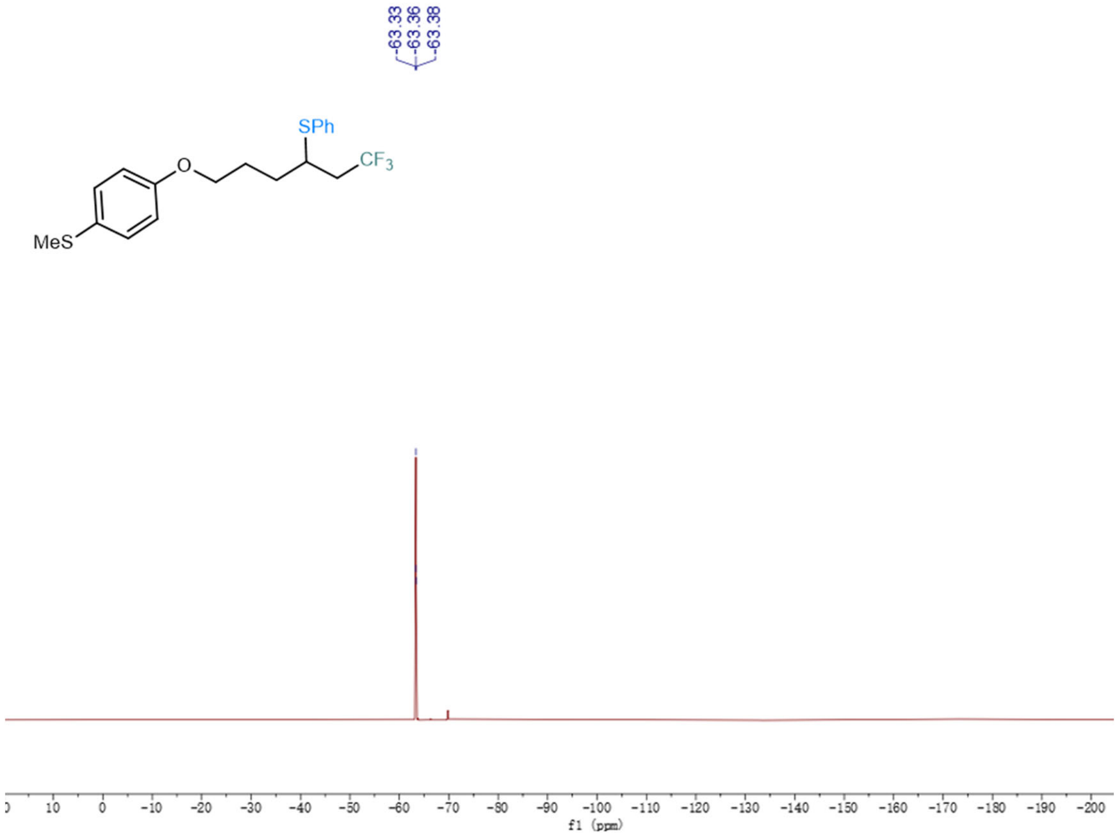

^{19}F NMR spectrum (471 MHz, Chloroform-*d*) of **25**

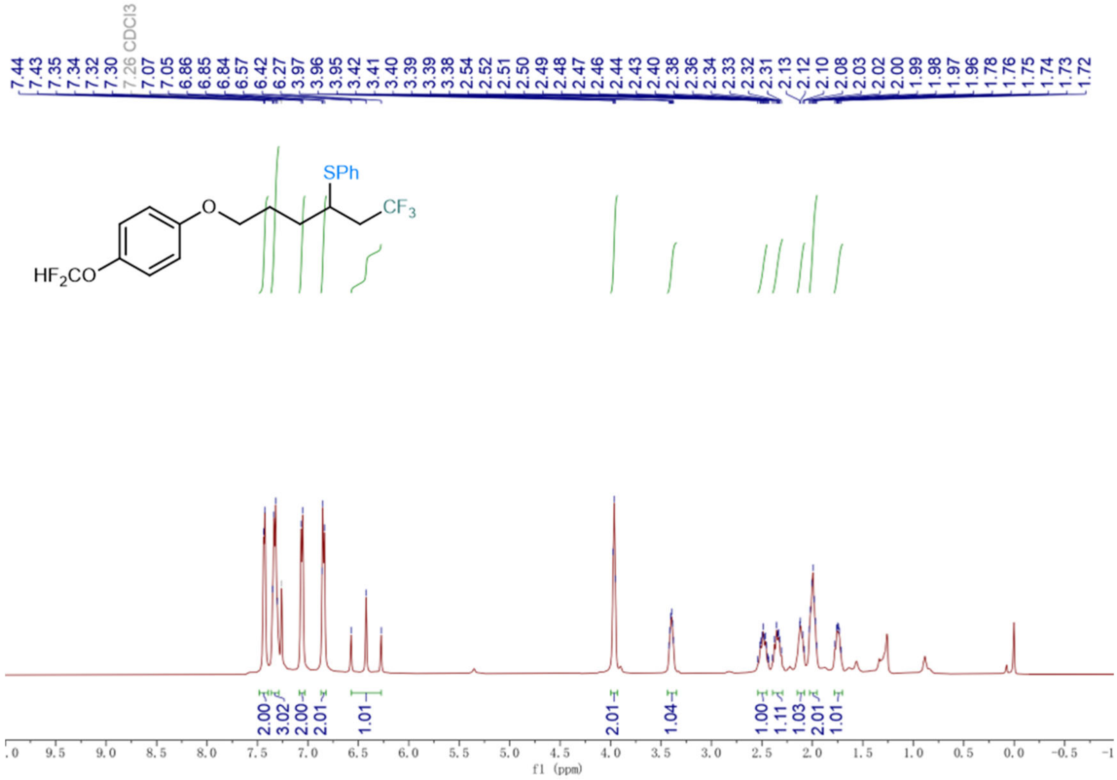

[1] H NMR spectrum (500 MHz, Chloroform-*d*) of **26**

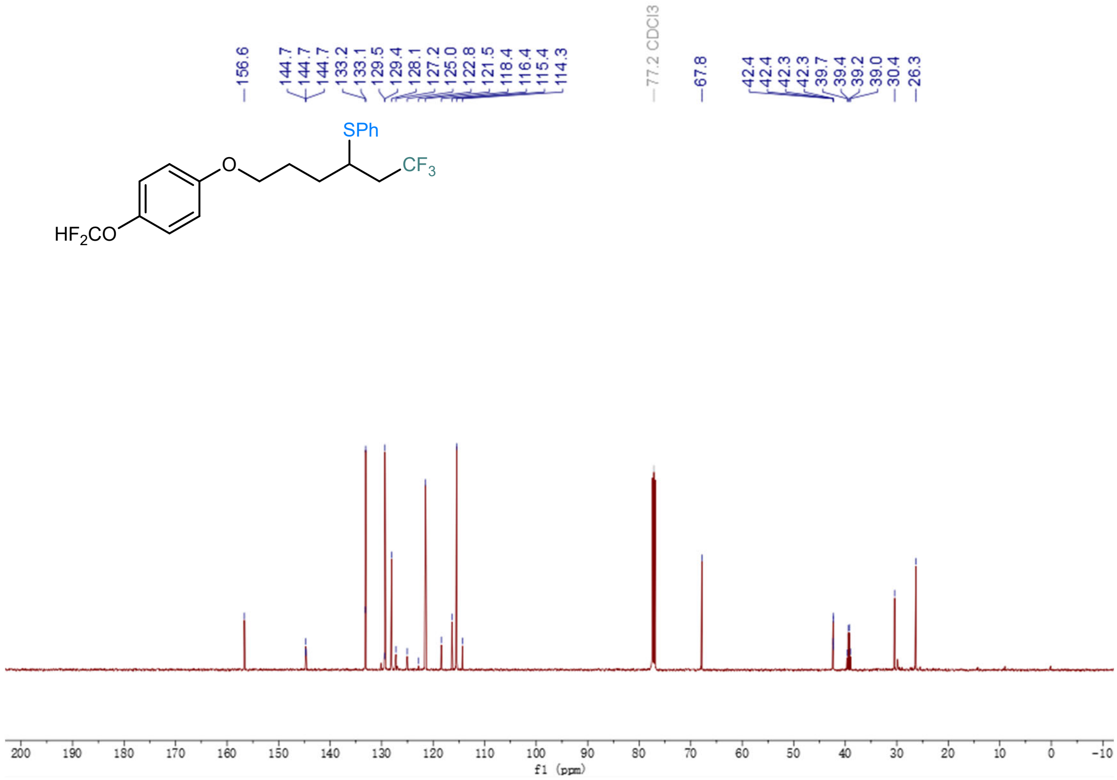

[13] C NMR spectrum (126 MHz, Chloroform-*d*) of **26**

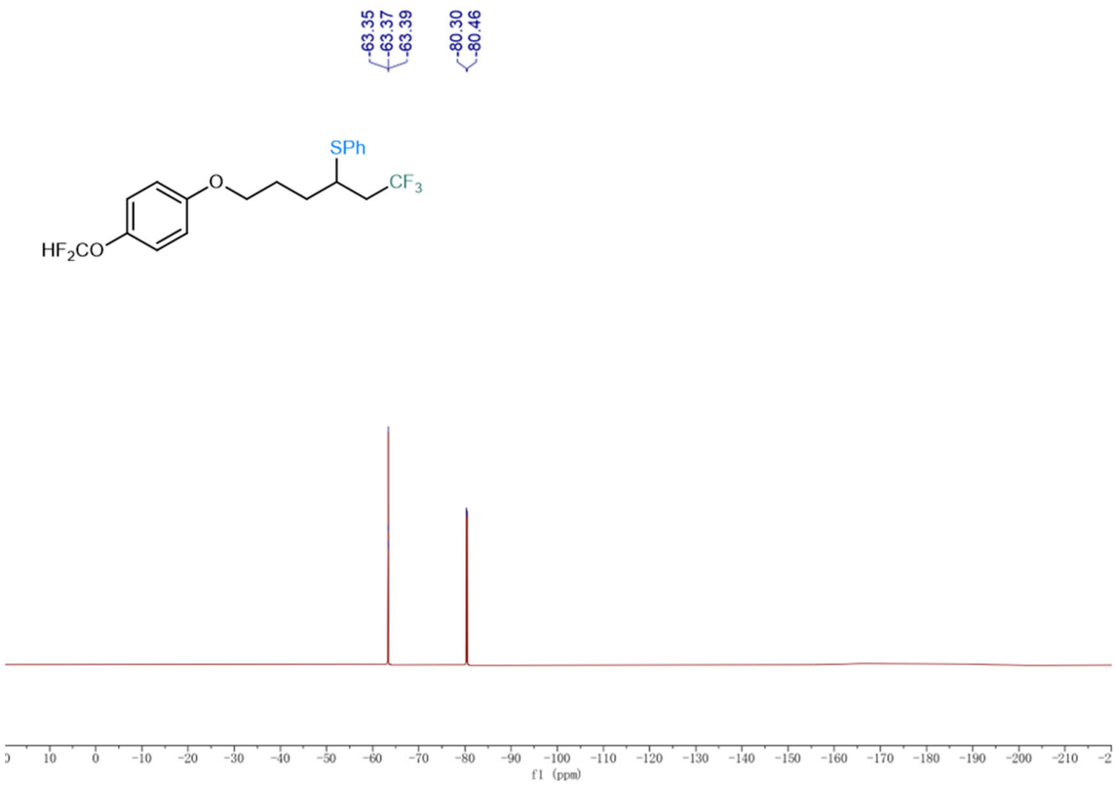

^{19}F NMR spectrum (471 MHz, Chloroform-*d*) of **26**

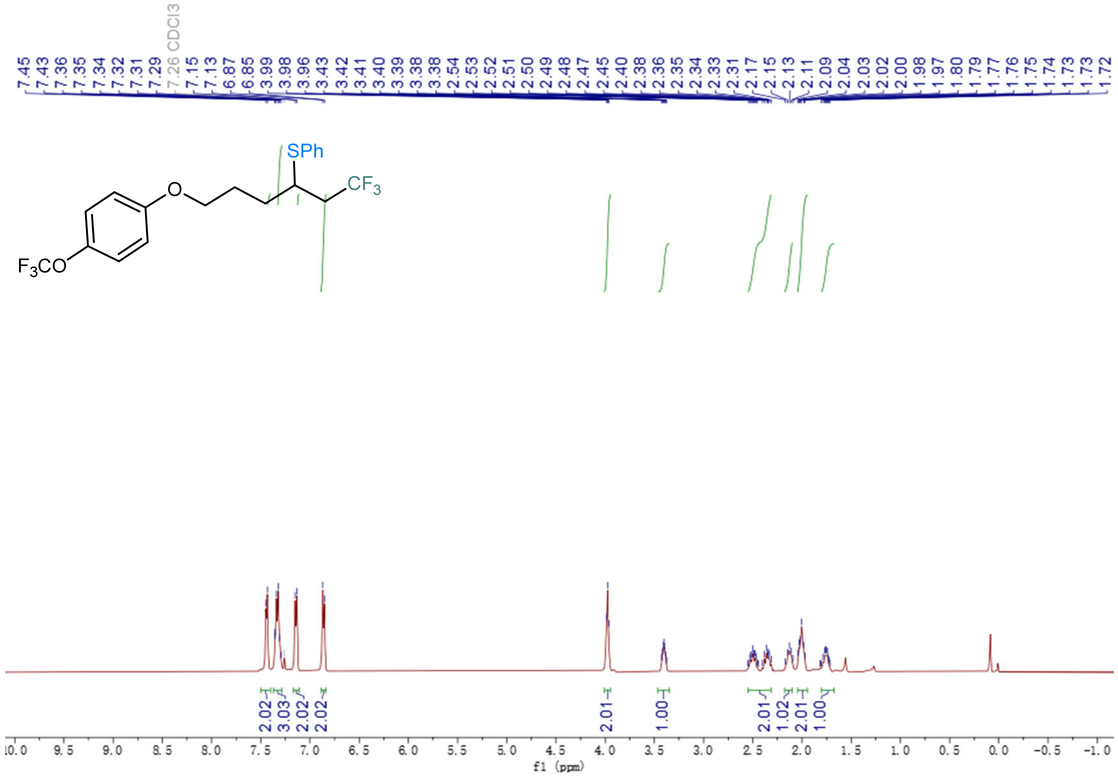

^{1}H NMR spectrum (500 MHz, Chloroform-*d*) of **27**

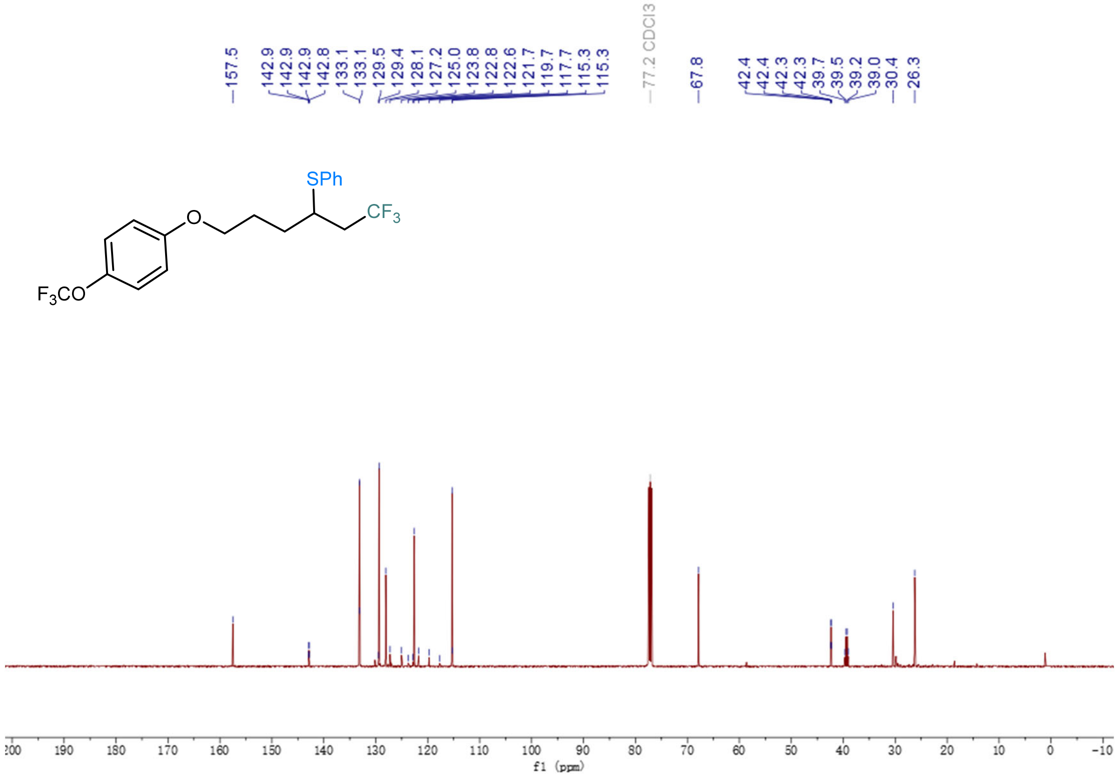

^{13}C NMR spectrum (126 MHz, Chloroform-*d*) of **27**

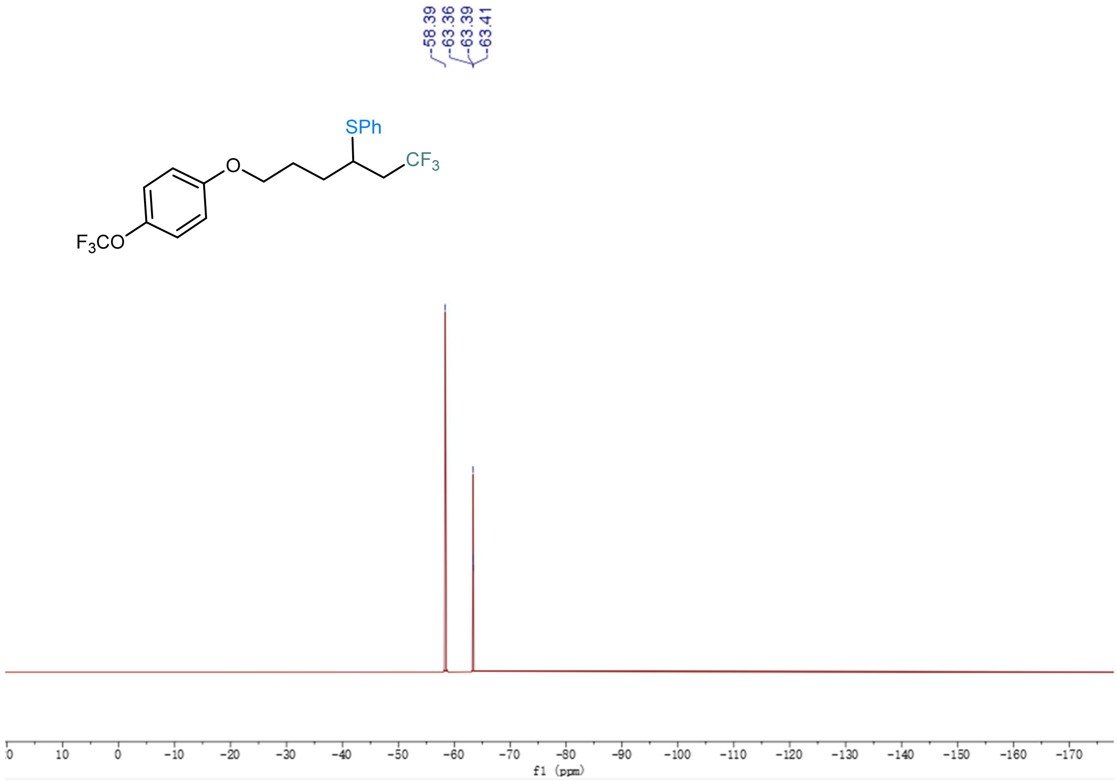

^{19}F NMR spectrum (471 MHz, Chloroform-*d*) of **27**

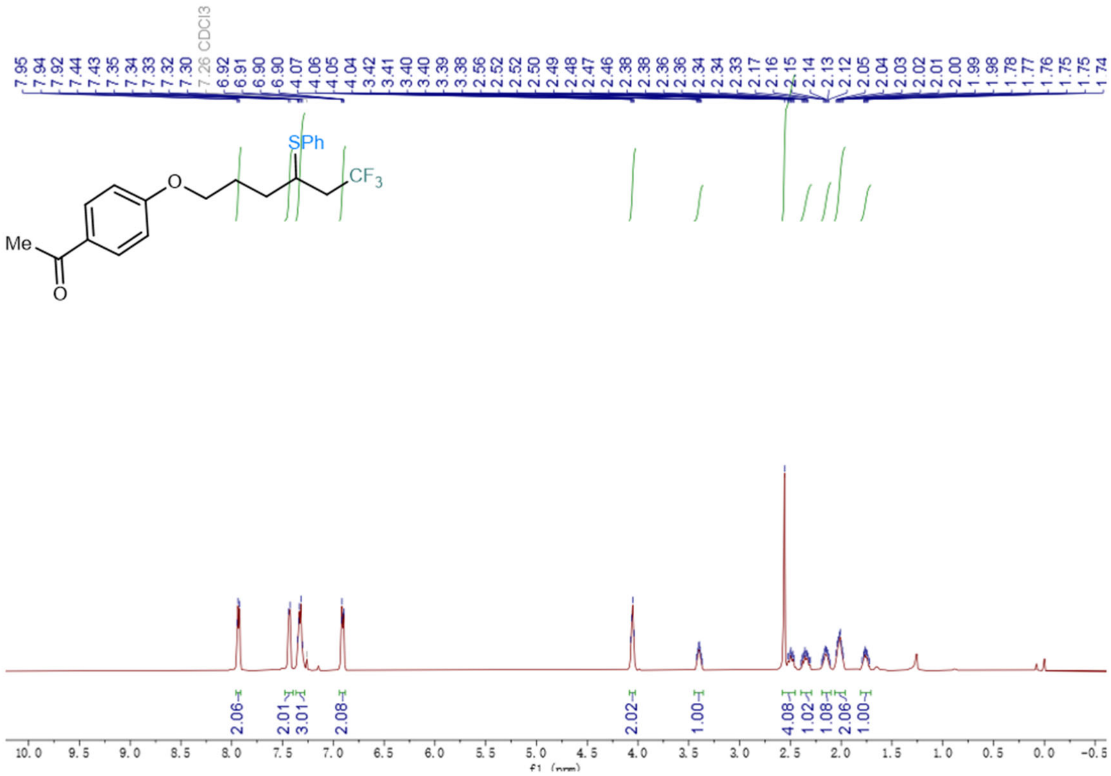

^{1}H NMR spectrum (500 MHz, Chloroform-*d*) of **28**

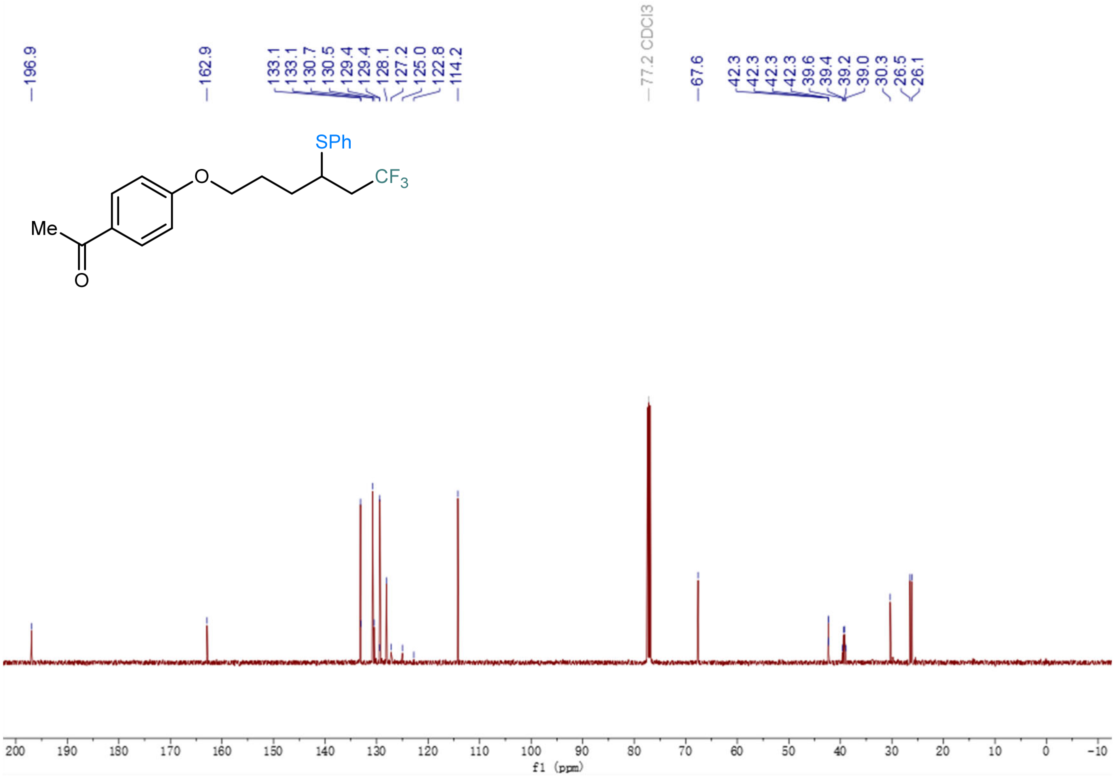

^{13}C NMR spectrum (126 MHz, Chloroform-*d*) of **28**

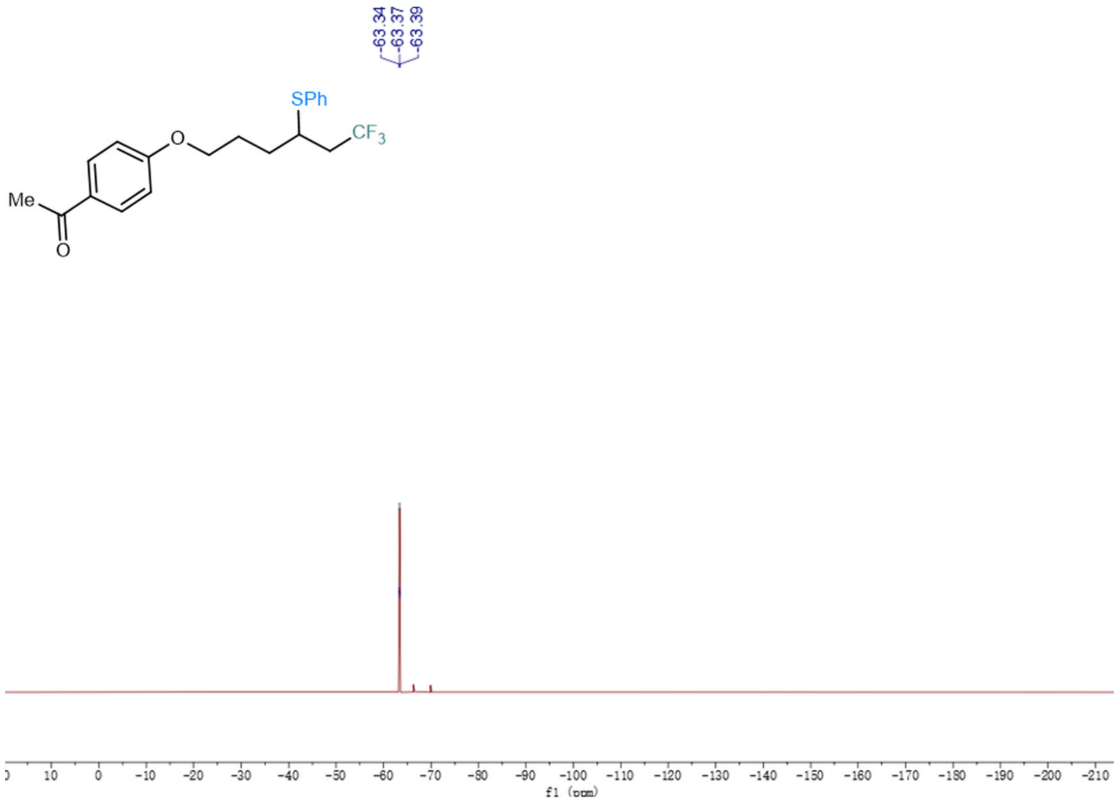

^{19}F NMR spectrum (471 MHz, Chloroform-*d*) of **28**

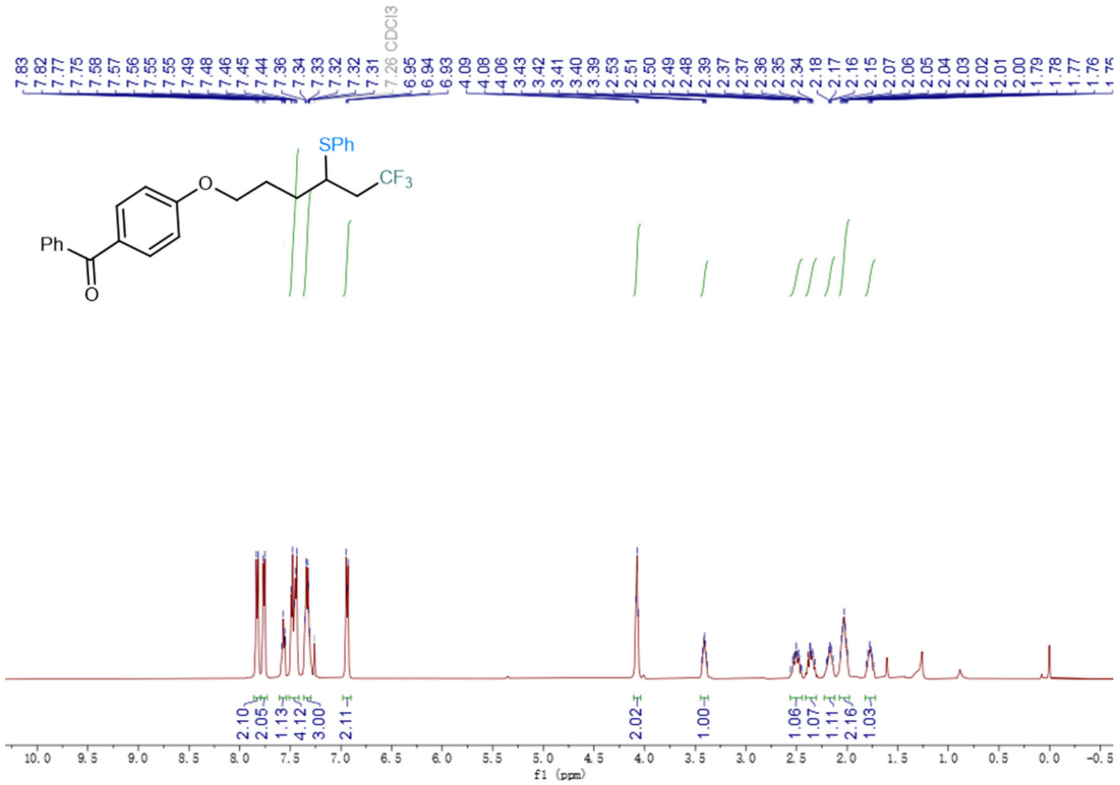

^{1}H NMR spectrum (500 MHz, Chloroform-*d*) of **29**

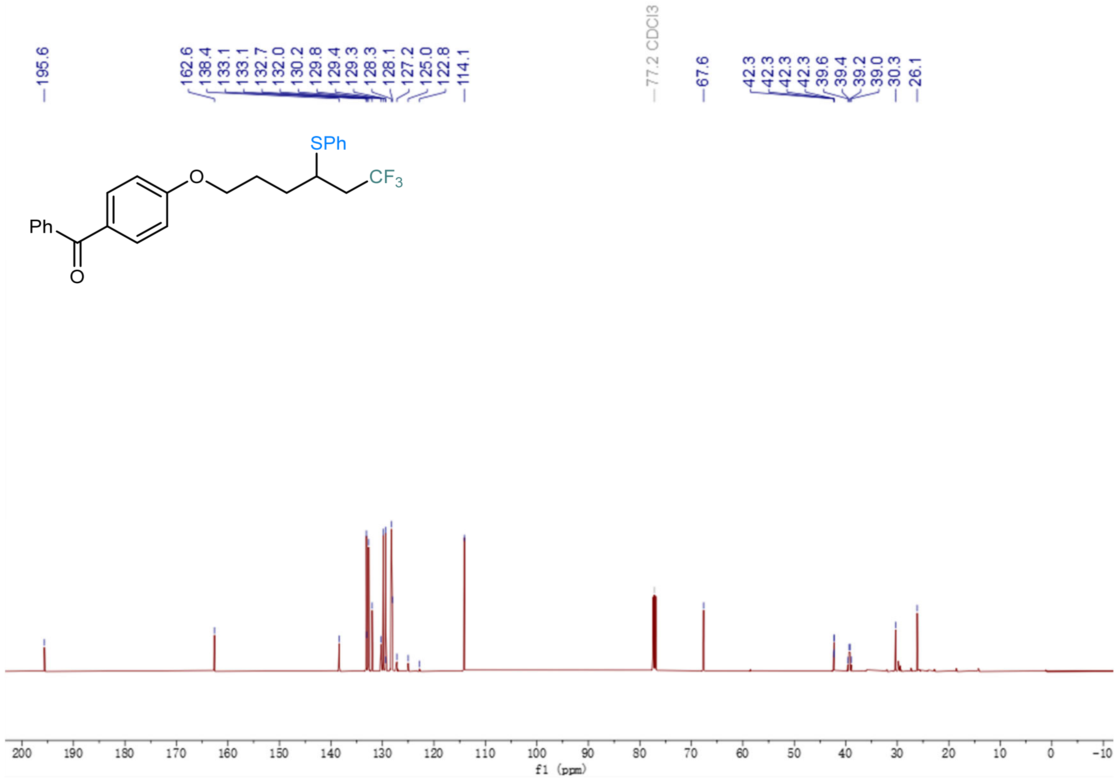

^{13}C NMR spectrum (126 MHz, Chloroform-*d*) of **29**

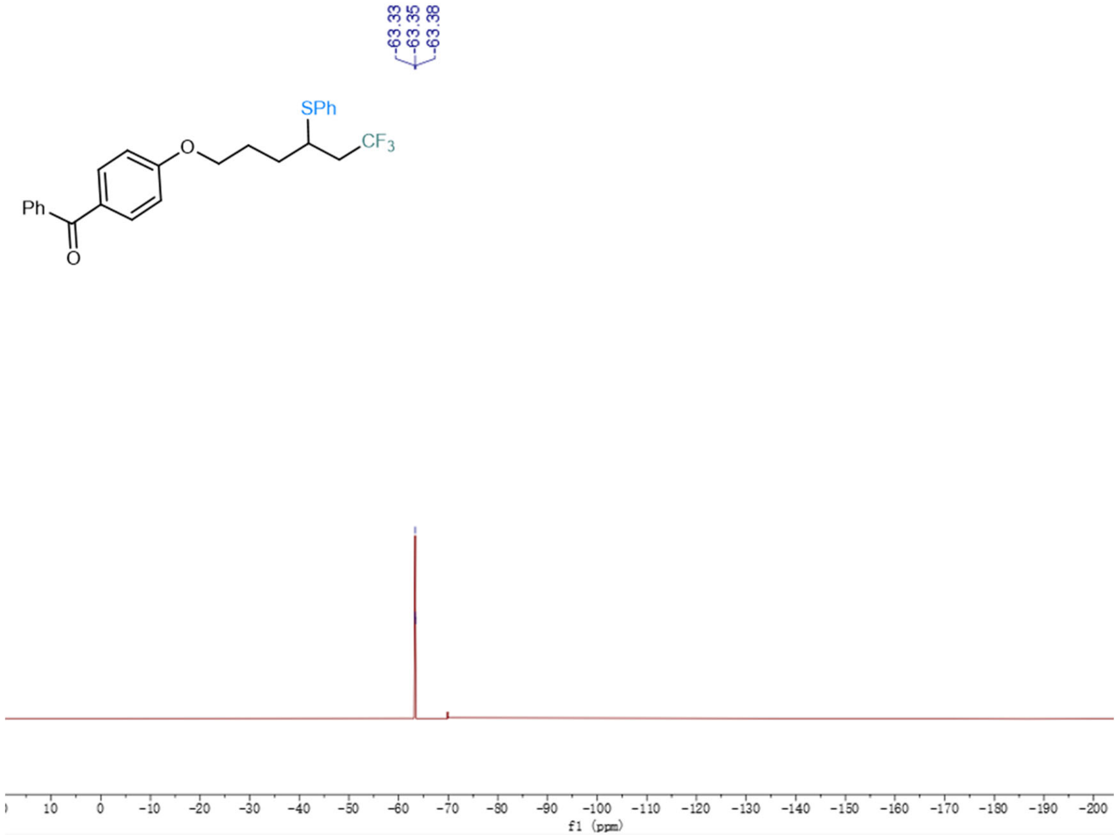

^{19}F NMR spectrum (471 MHz, Chloroform-*d*) of **29**

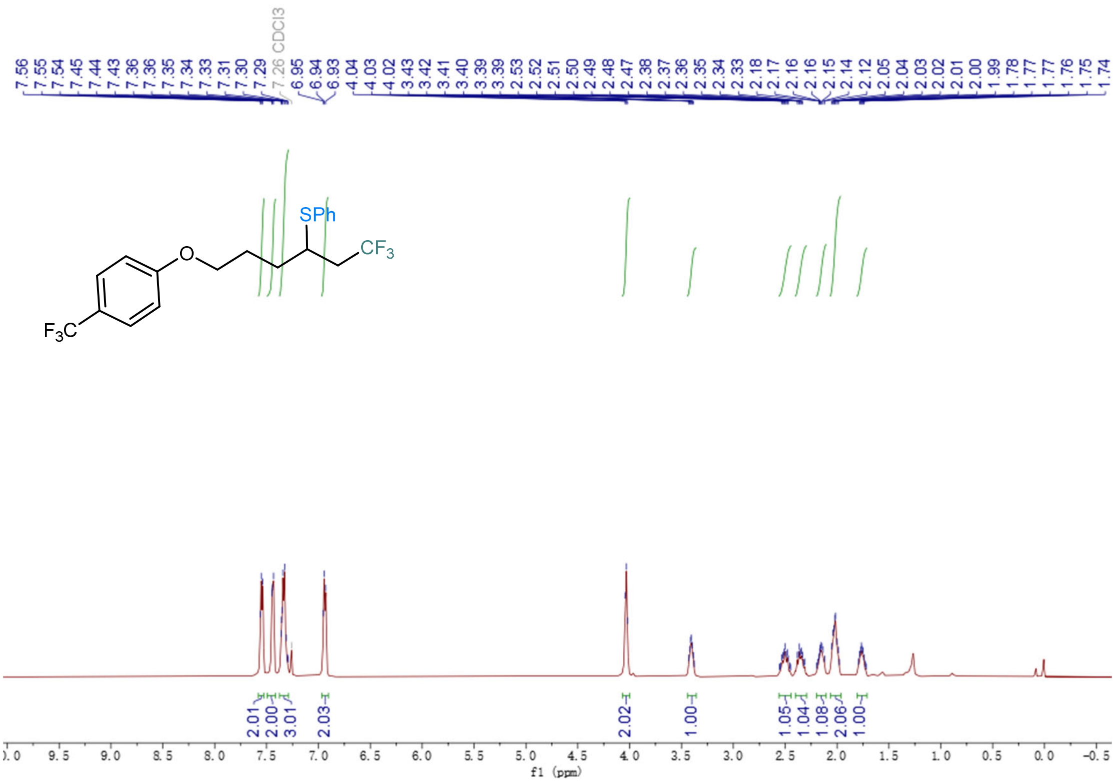

^{1}H NMR spectrum (500 MHz, Chloroform-*d*) of **30**

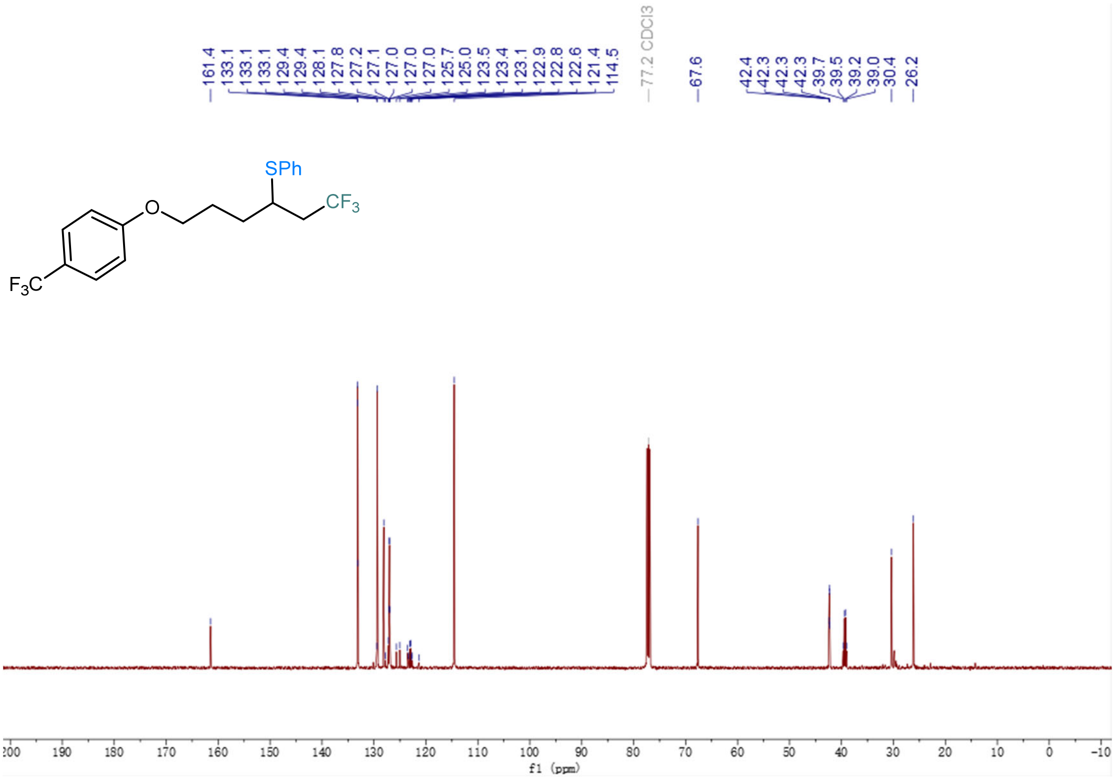

^{13}C NMR spectrum (126 MHz, Chloroform-*d*) of **30**

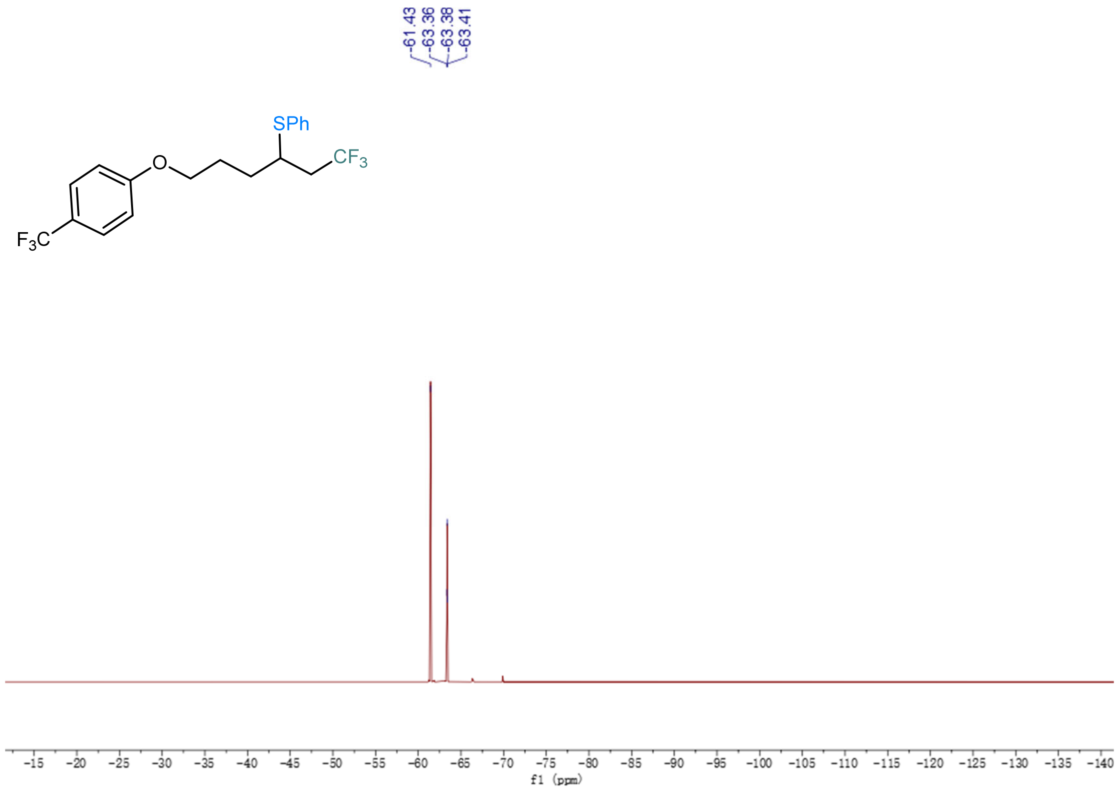

^{19}F NMR spectrum (471 MHz, Chloroform-*d*) of **30**

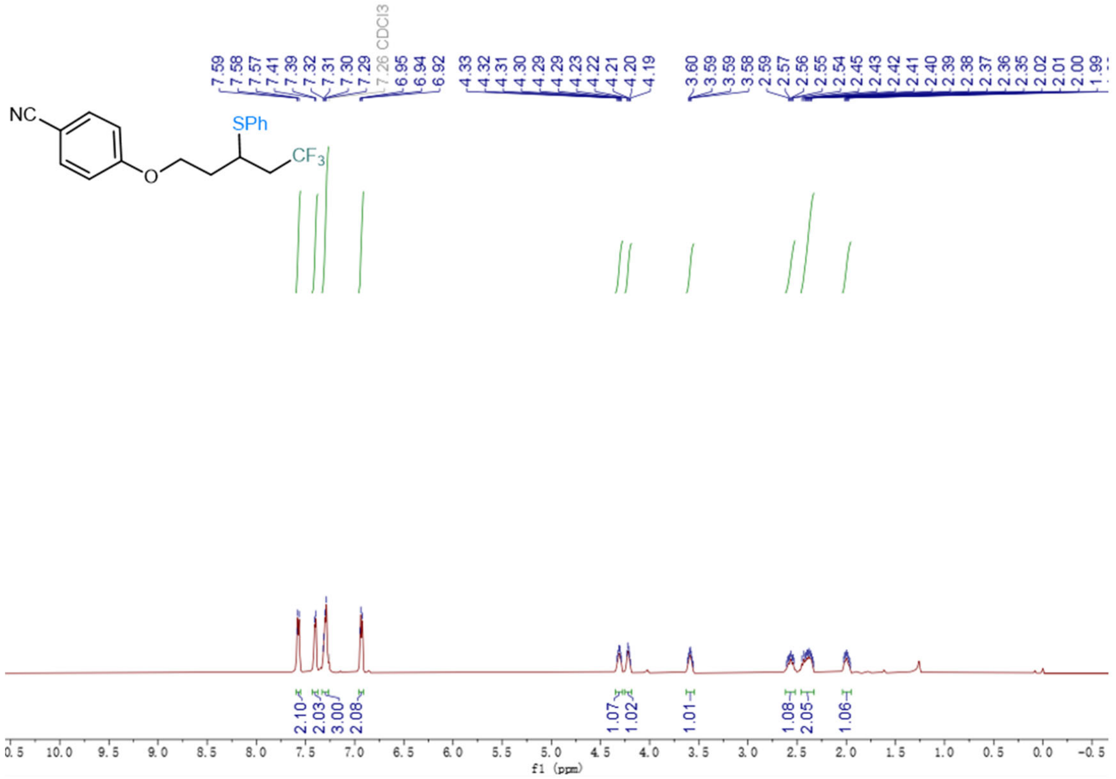

^{1}H NMR spectrum (500 MHz, Chloroform-*d*) of **31**

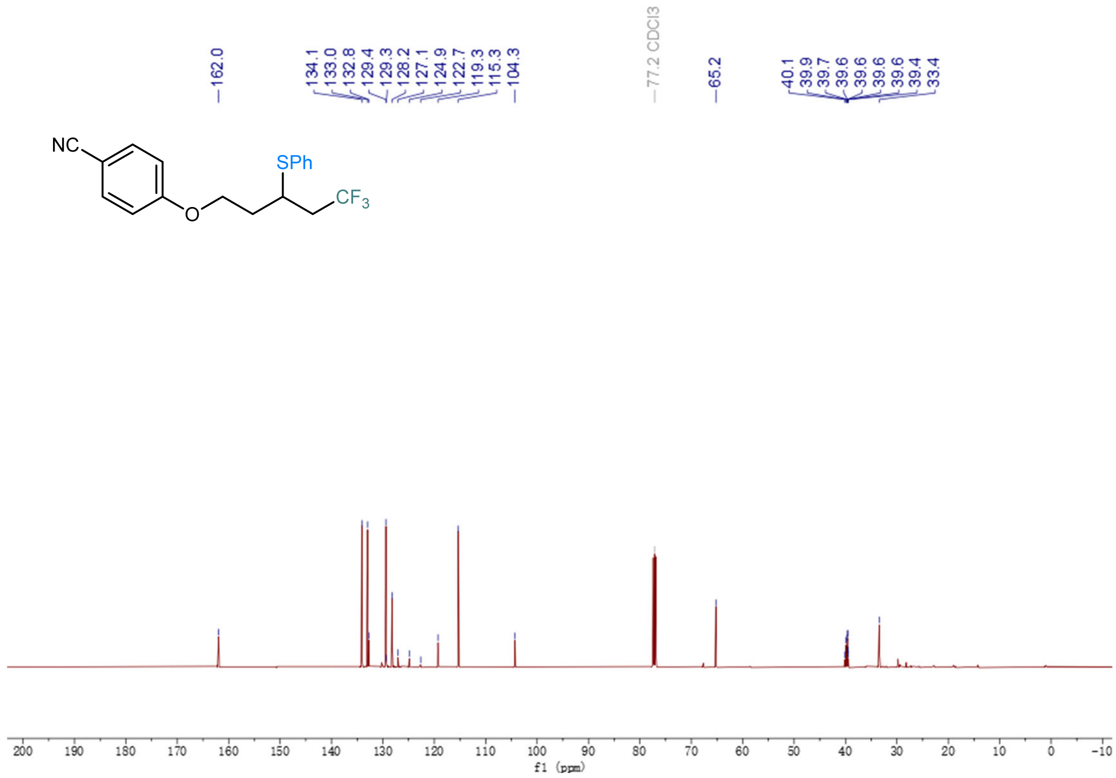

^{13}C NMR spectrum (126 MHz, Chloroform-*d*) of **31**

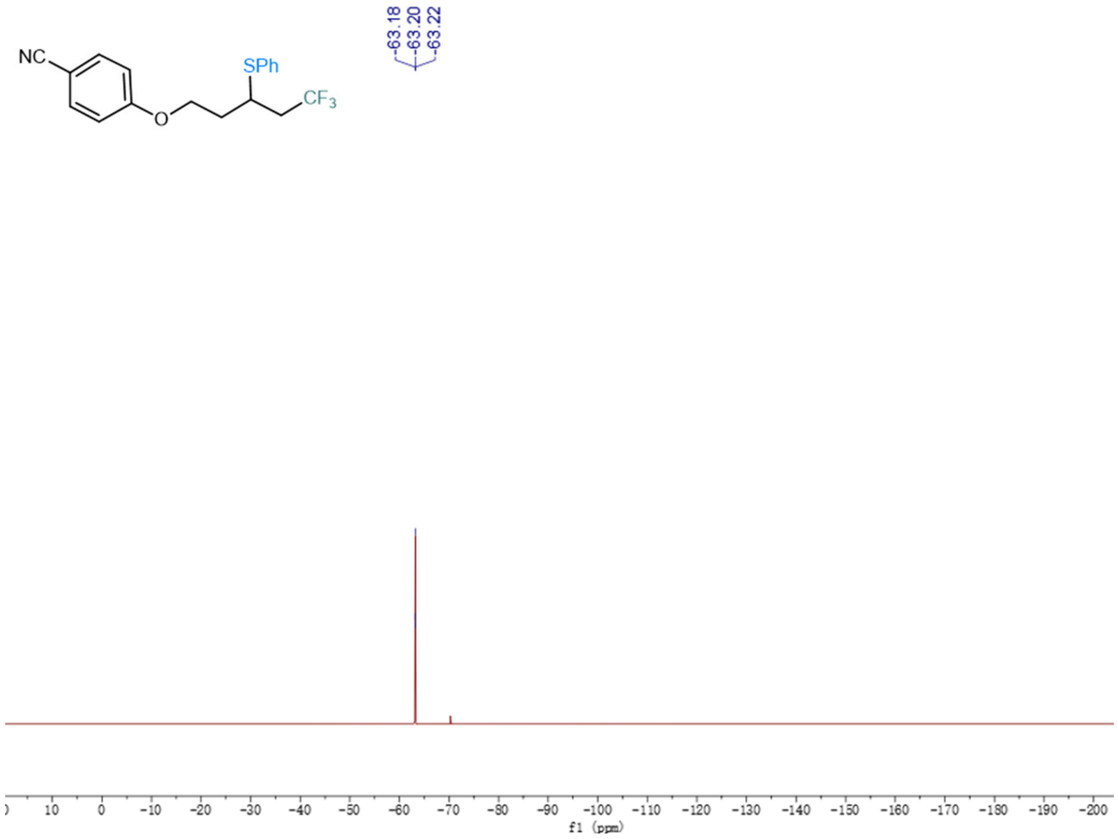

^{19}F NMR spectrum (471 MHz, Chloroform-*d*) of **31**

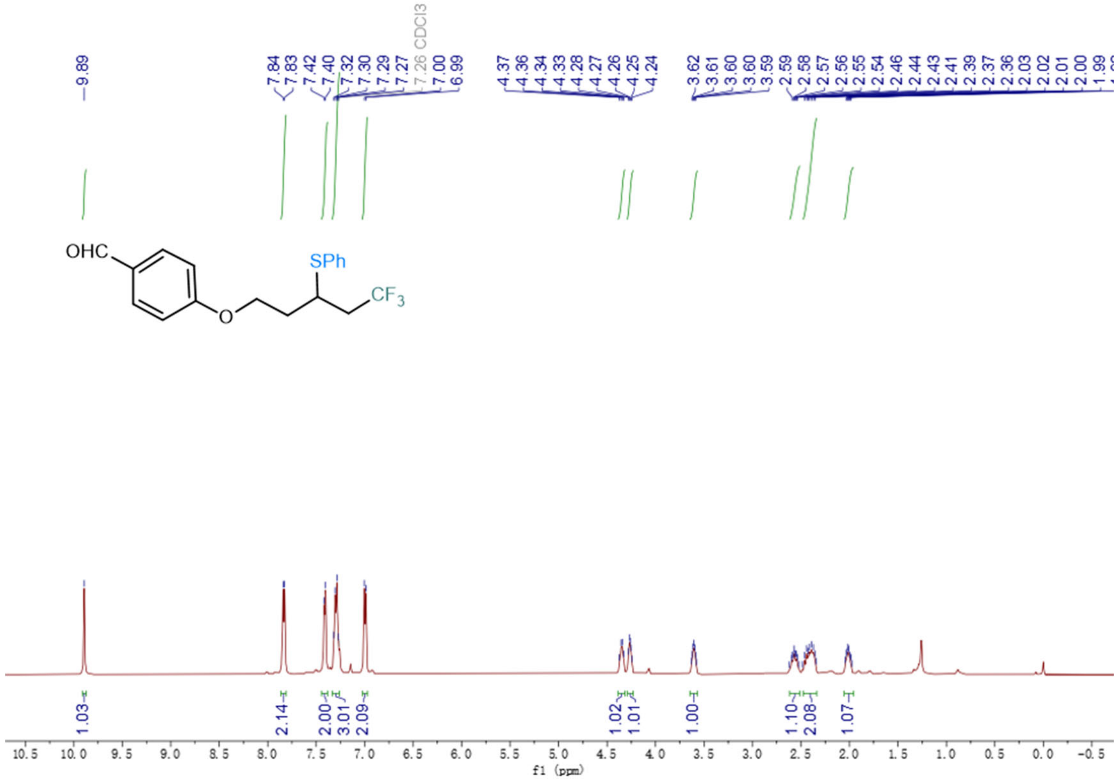

^{1}H NMR spectrum (500 MHz, Chloroform-*d*) of **32**

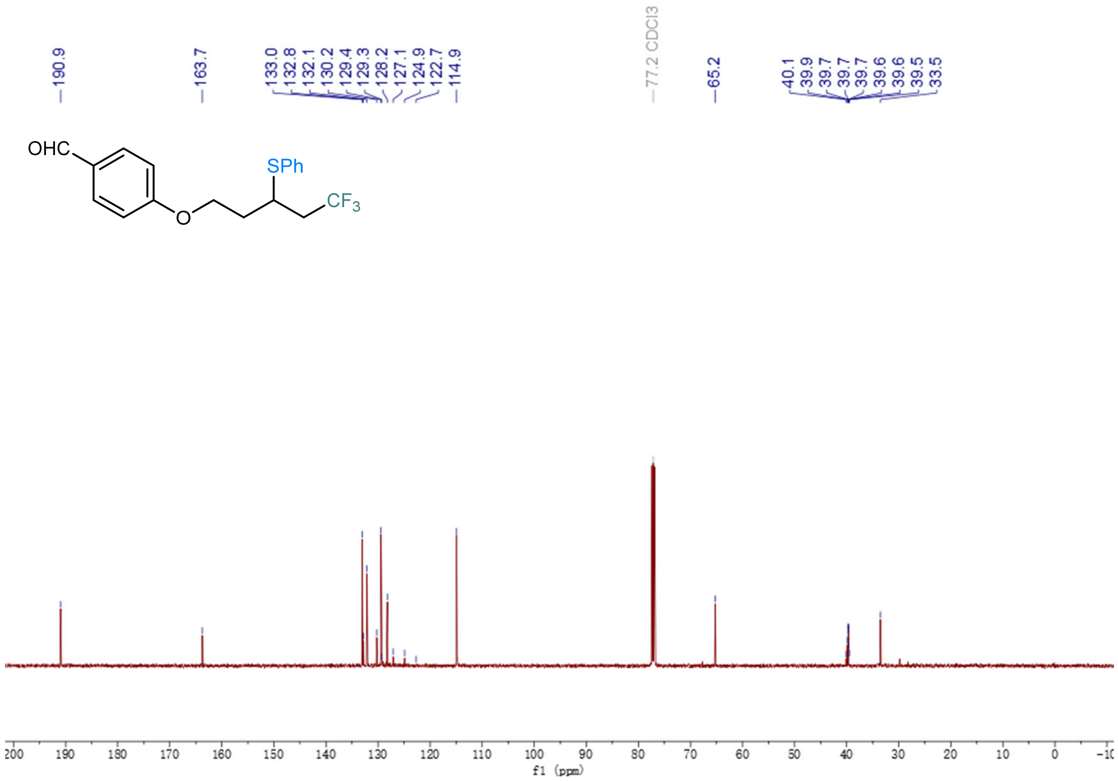

^{13}C NMR spectrum (126 MHz, Chloroform-*d*) of **32**

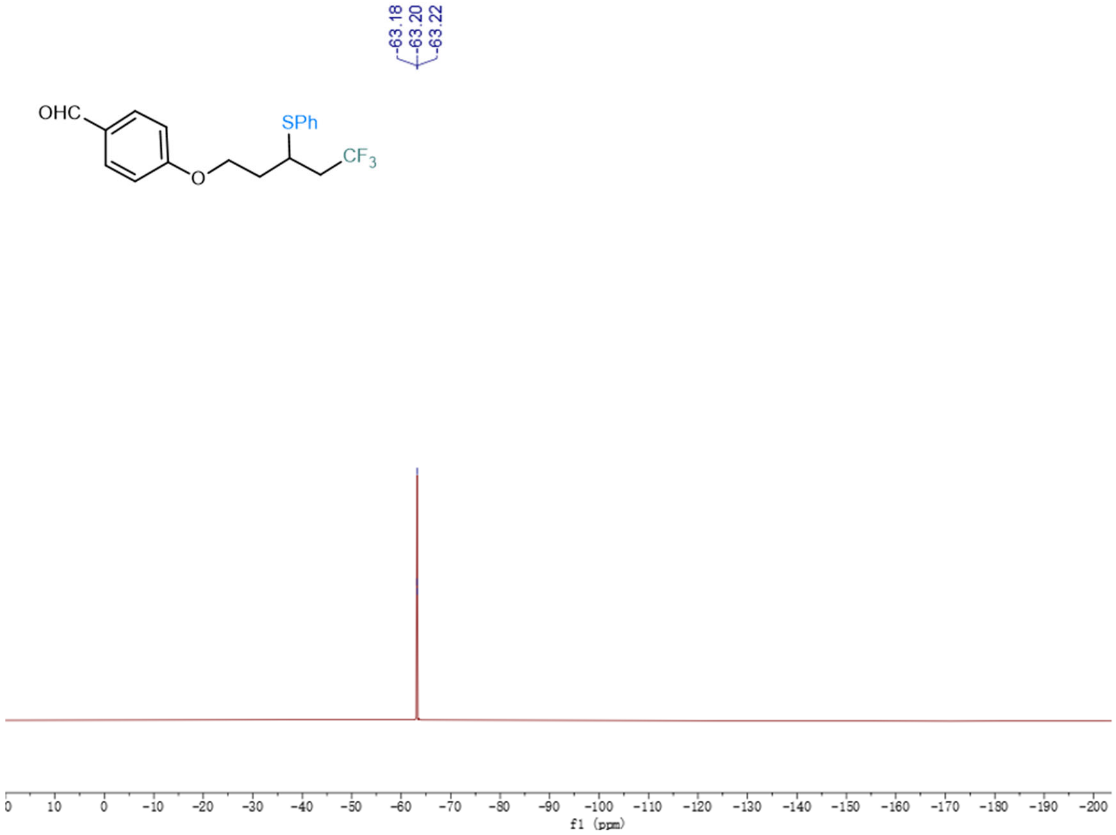

^{19}F NMR spectrum (471 MHz, Chloroform-*d*) of **32**

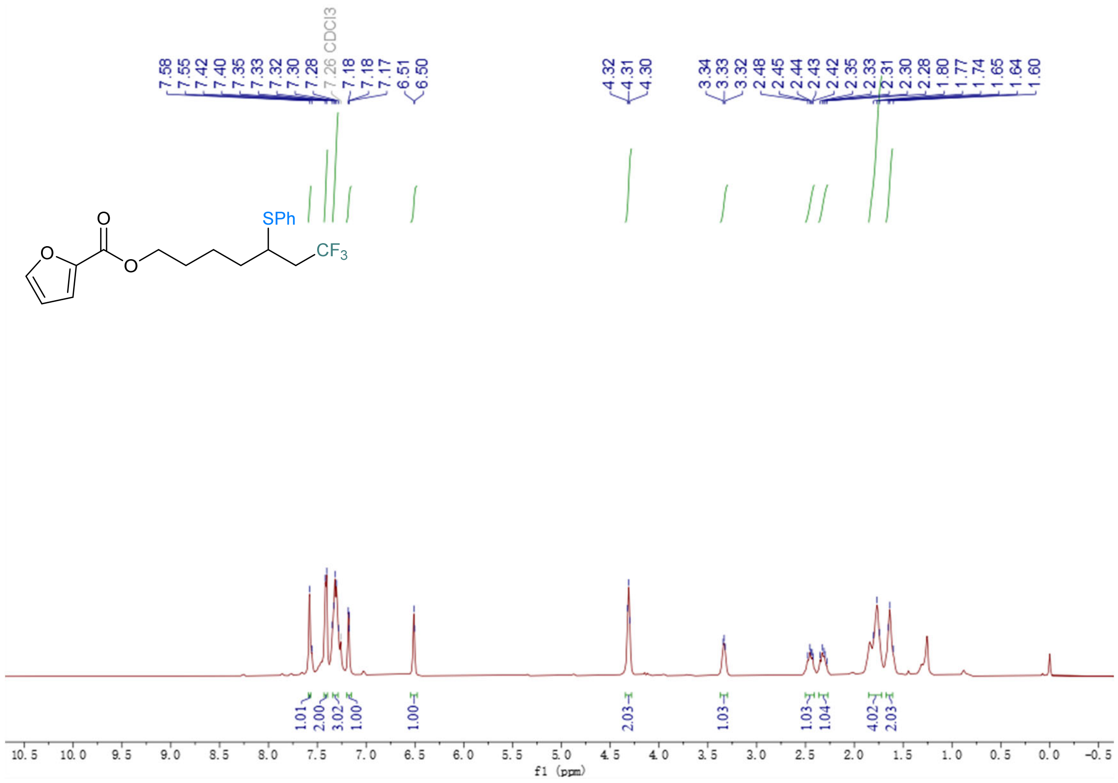

^{1}H NMR spectrum (500 MHz, Chloroform-*d*) of **33**

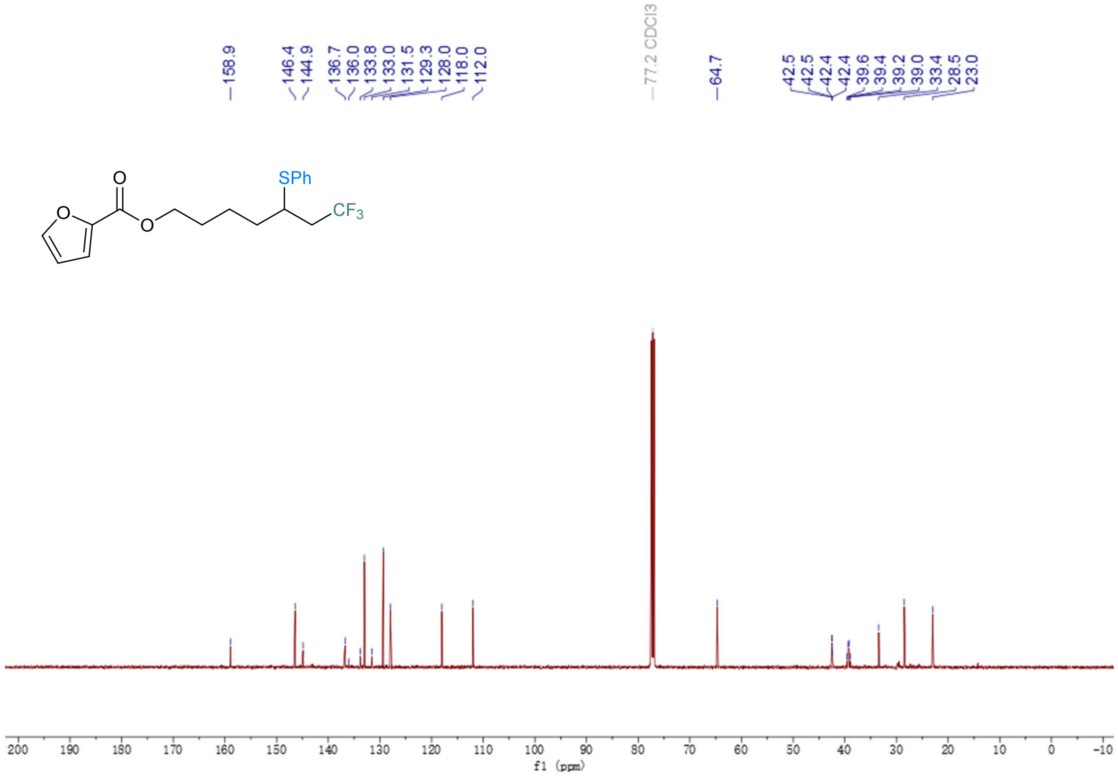

^{13}C NMR spectrum (126 MHz, Chloroform-*d*) of **33**

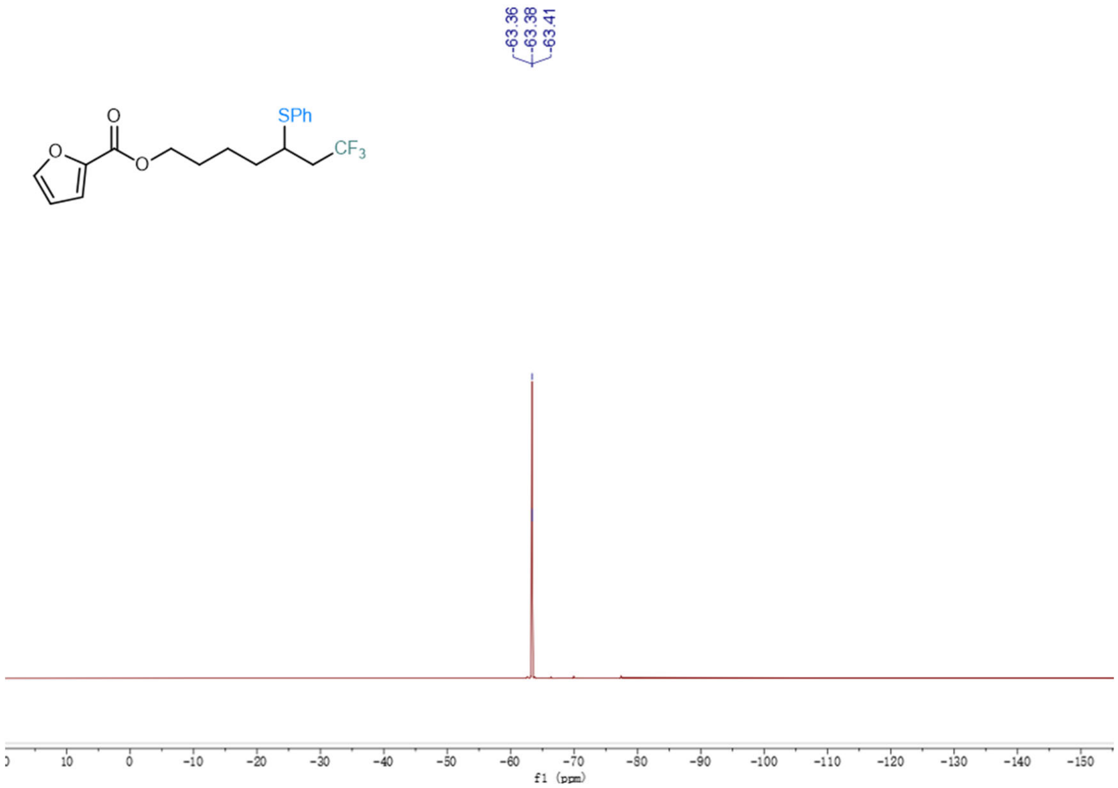

^{19}F NMR spectrum (471 MHz, Chloroform-*d*) of **33**

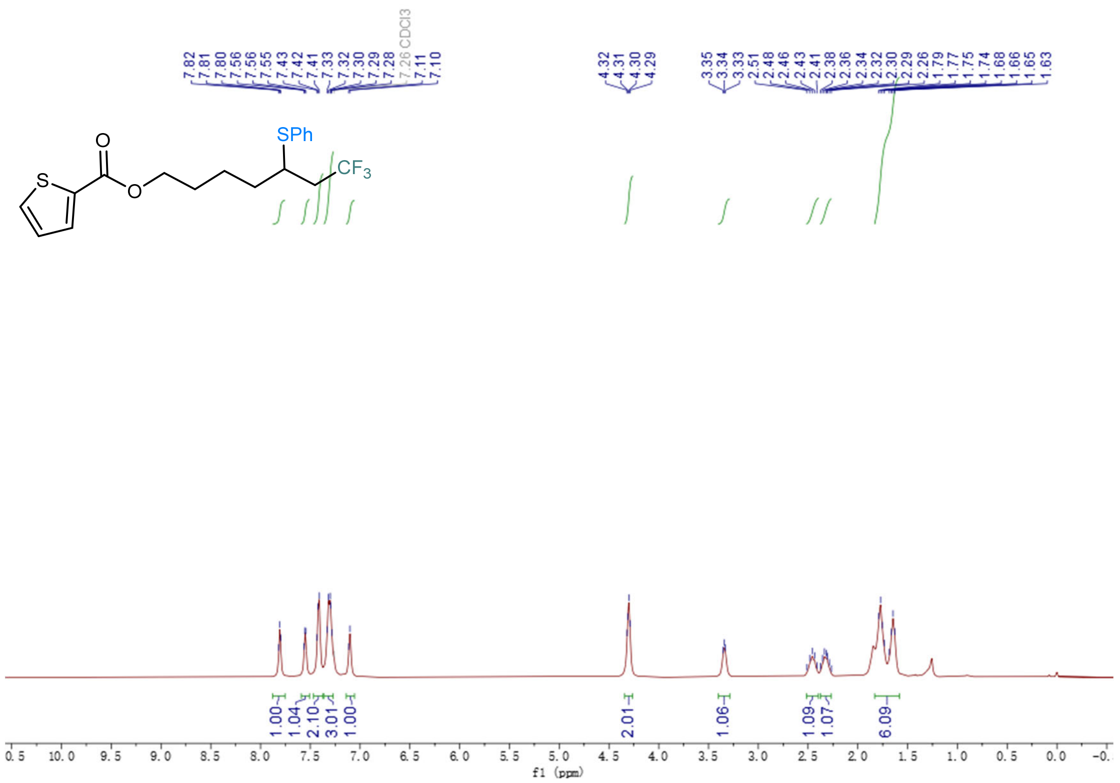

^{1}H NMR spectrum (500 MHz, Chloroform-*d*) of **34**

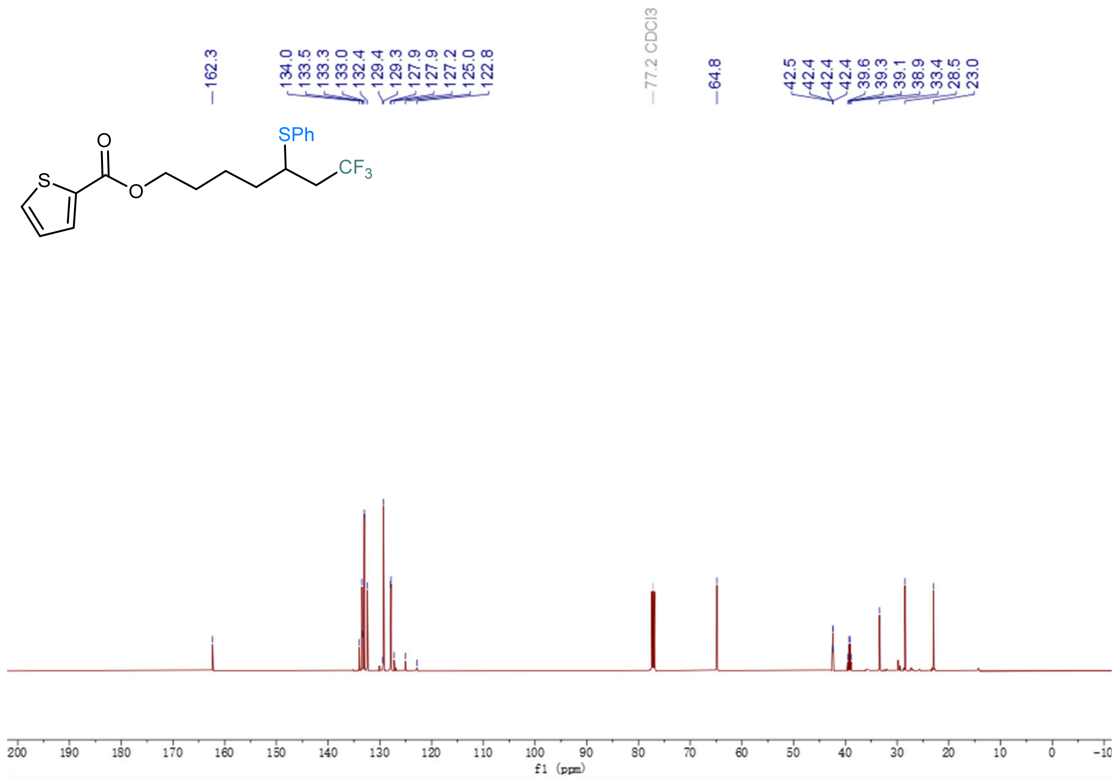

^{13}C NMR spectrum (126 MHz, Chloroform-*d*) of **34**

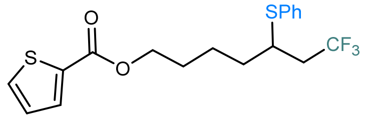

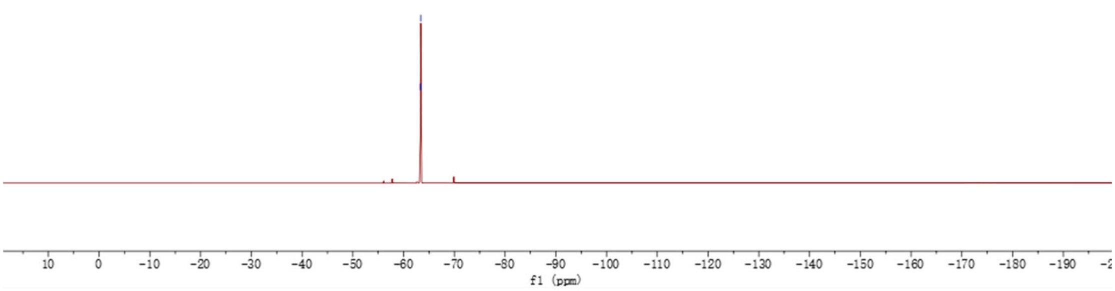

^{19}F NMR spectrum (471 MHz, Chloroform-*d*) of **34**

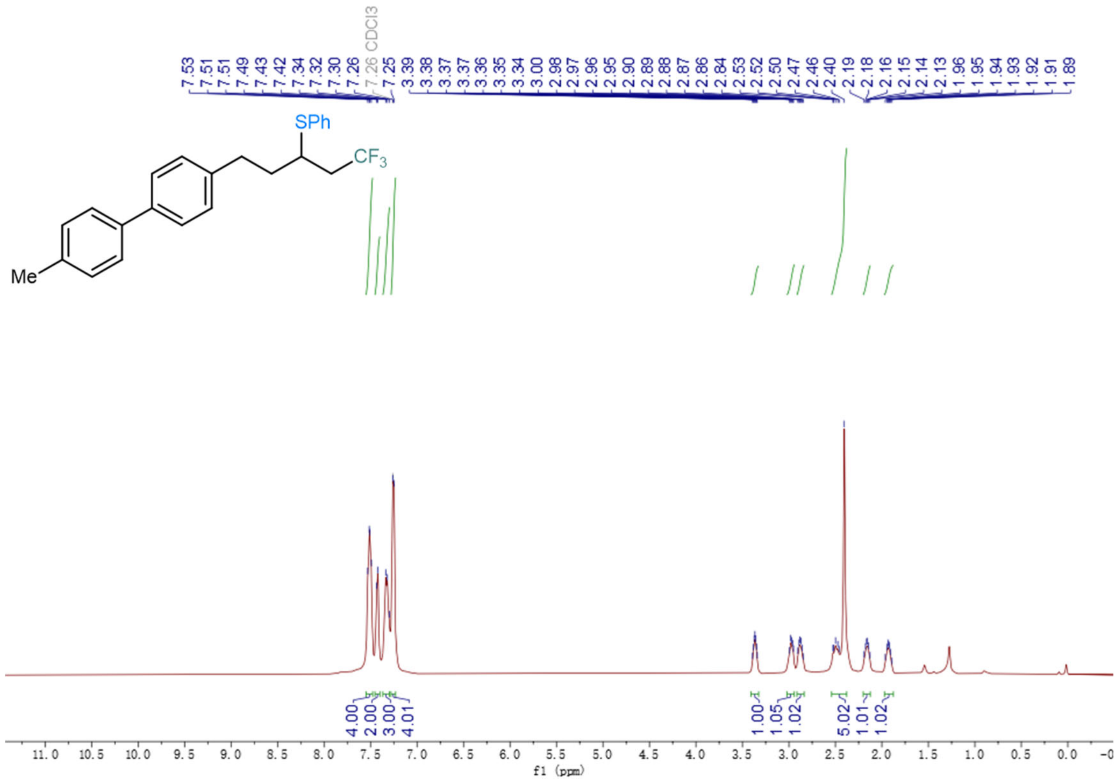

^{1}H NMR spectrum (500 MHz, Chloroform-*d*) of **35**

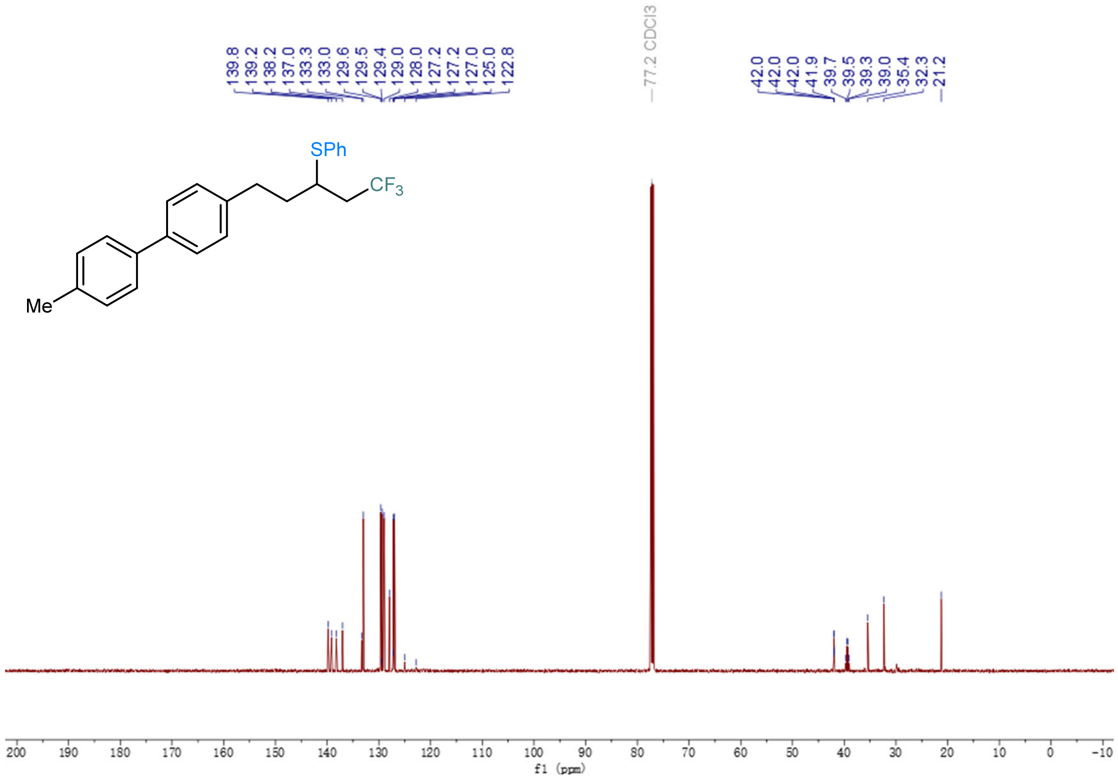

^{13}C NMR spectrum (126 MHz, Chloroform-*d*) of **35**

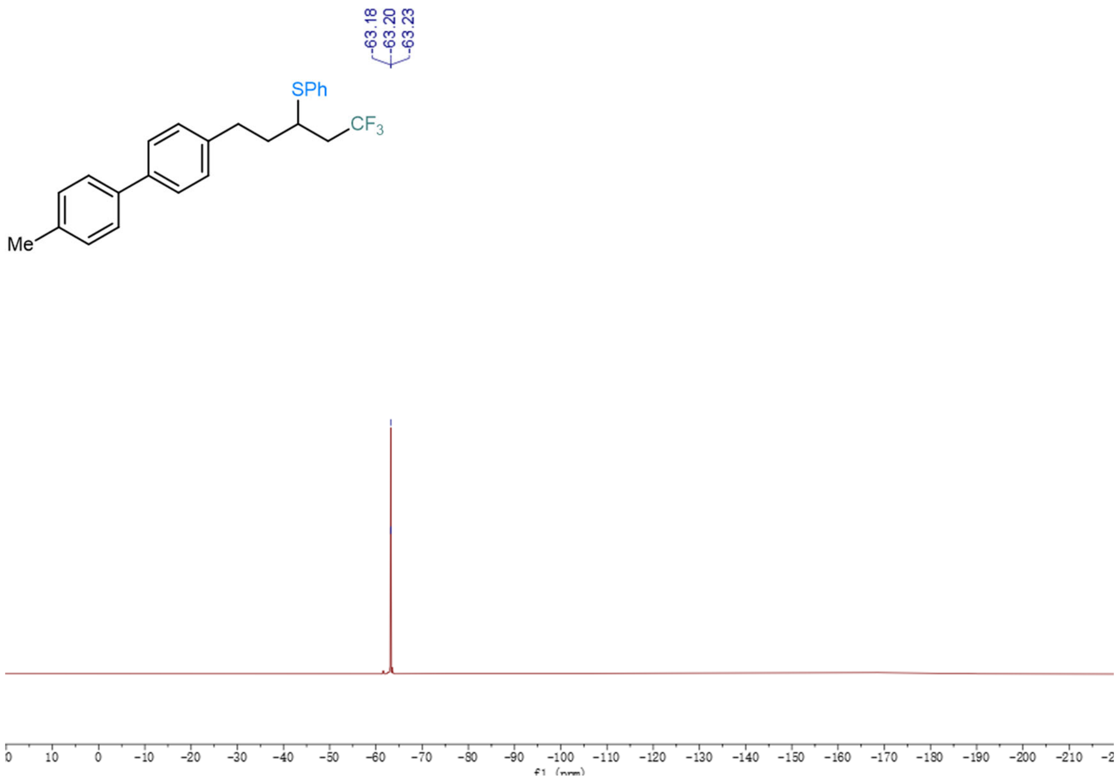

^{19}F NMR spectrum (471 MHz, Chloroform-*d*) **35**

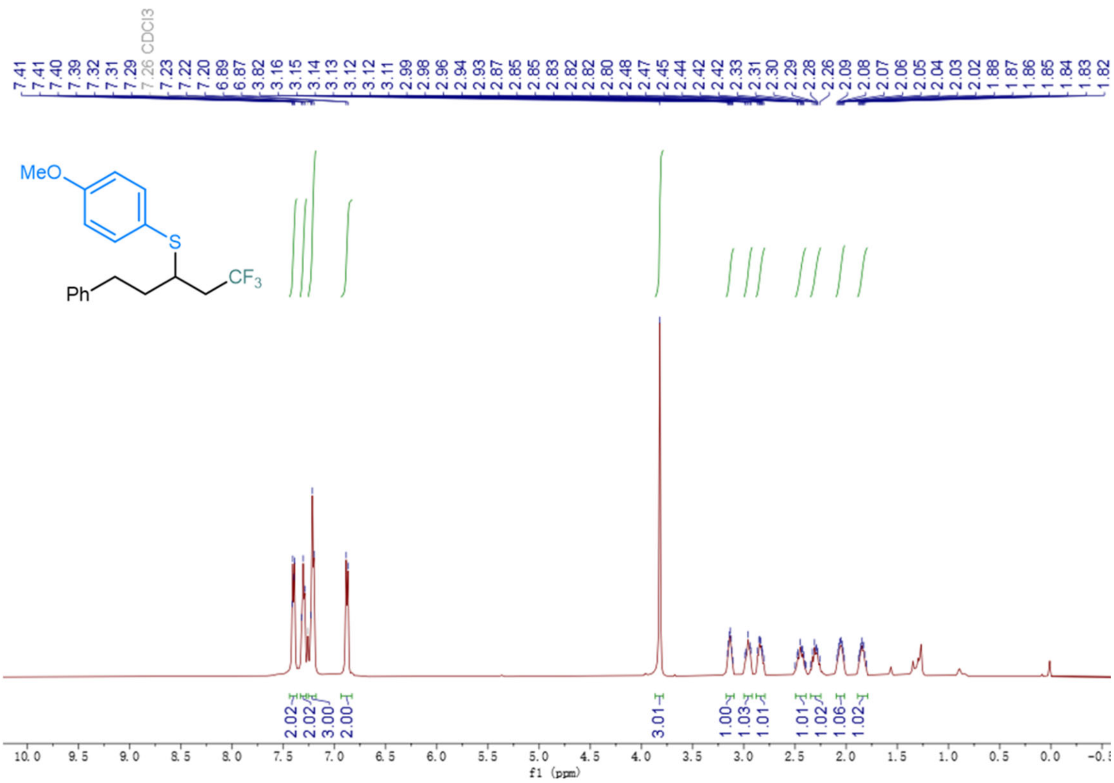

^{1}H NMR spectrum (500 MHz, Chloroform-*d*) of **36**

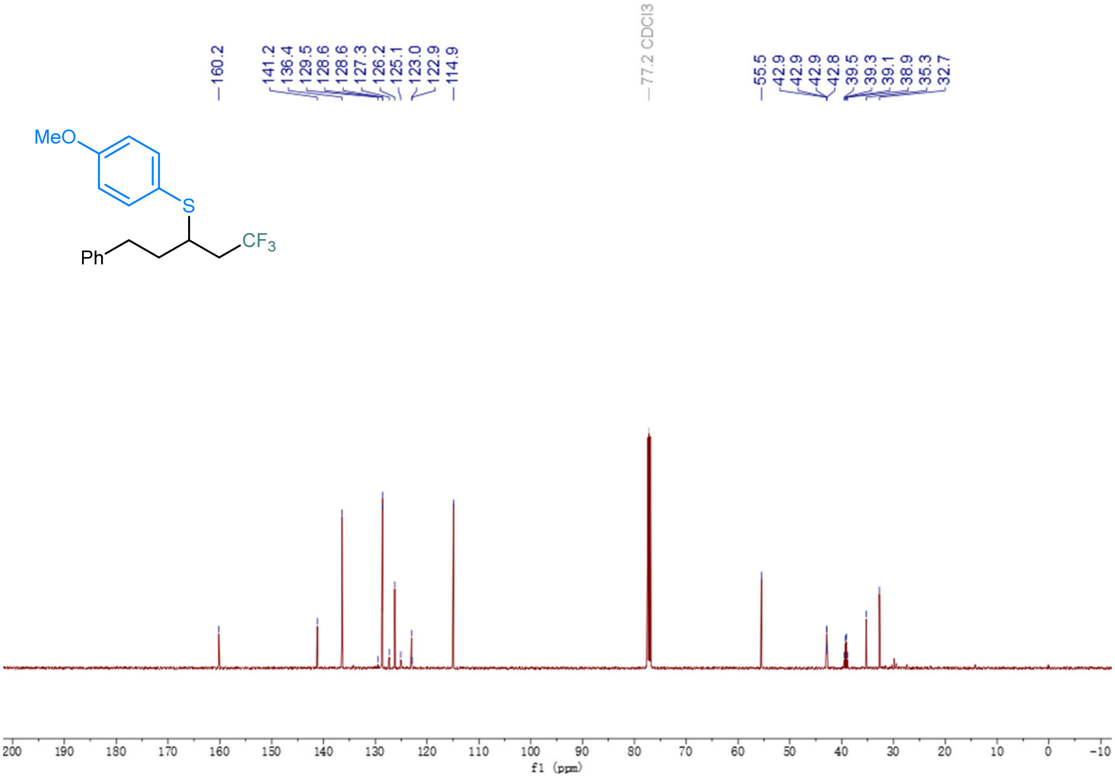

^{13}C NMR spectrum (126 MHz, Chloroform-*d*) of **36**

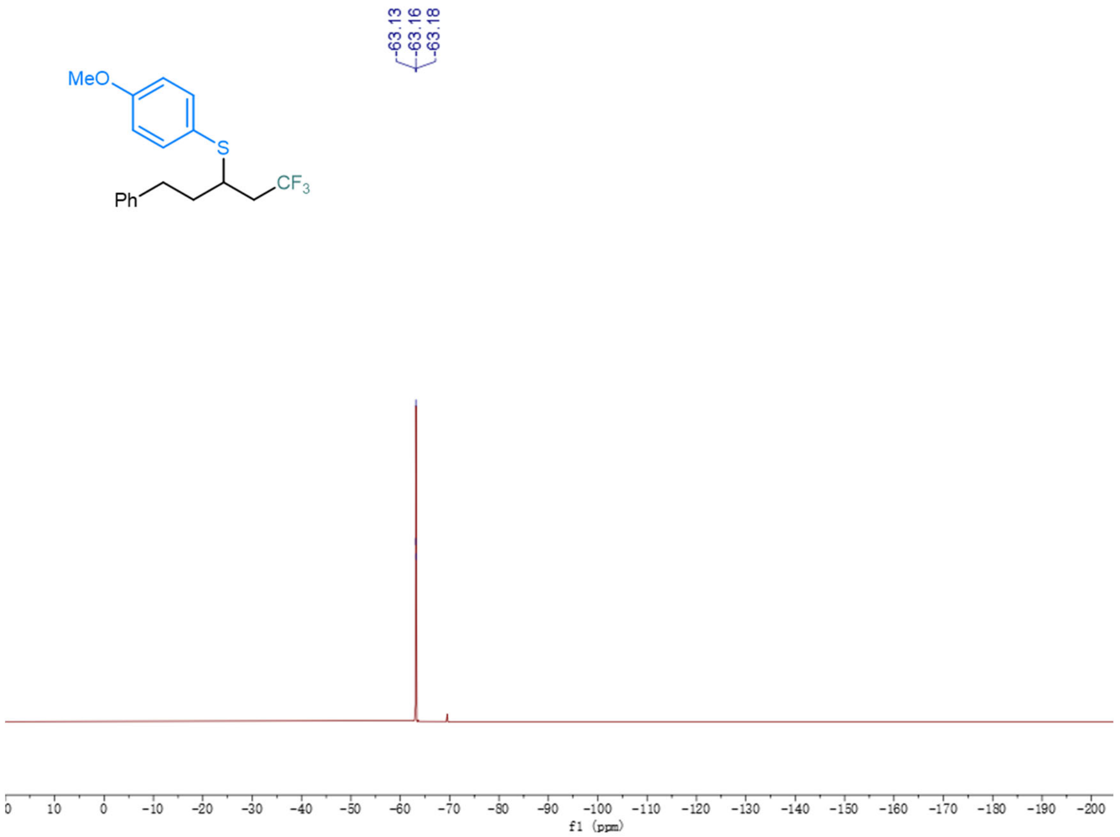

^{19}F NMR spectrum (471 MHz, Chloroform-*d*) of **36**

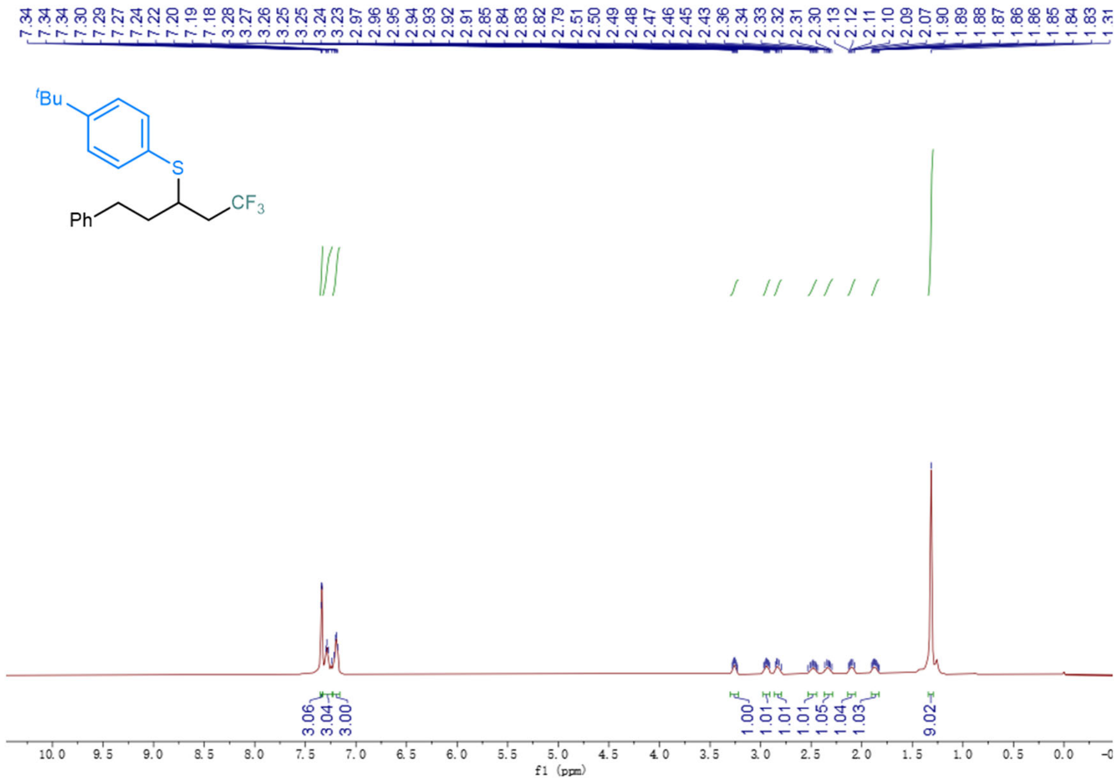

^{1}H NMR spectrum (500 MHz, Chloroform-*d*) of **37**

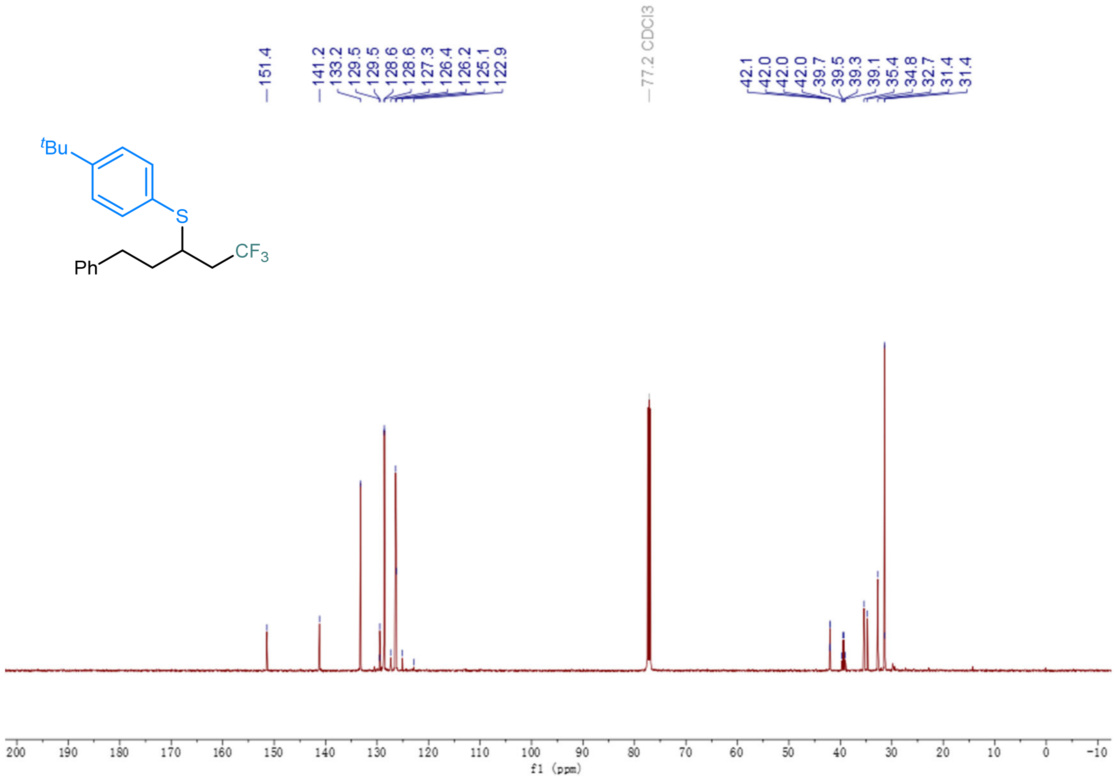

^{13}C NMR spectrum (126 MHz, Chloroform-*d*) of **37**

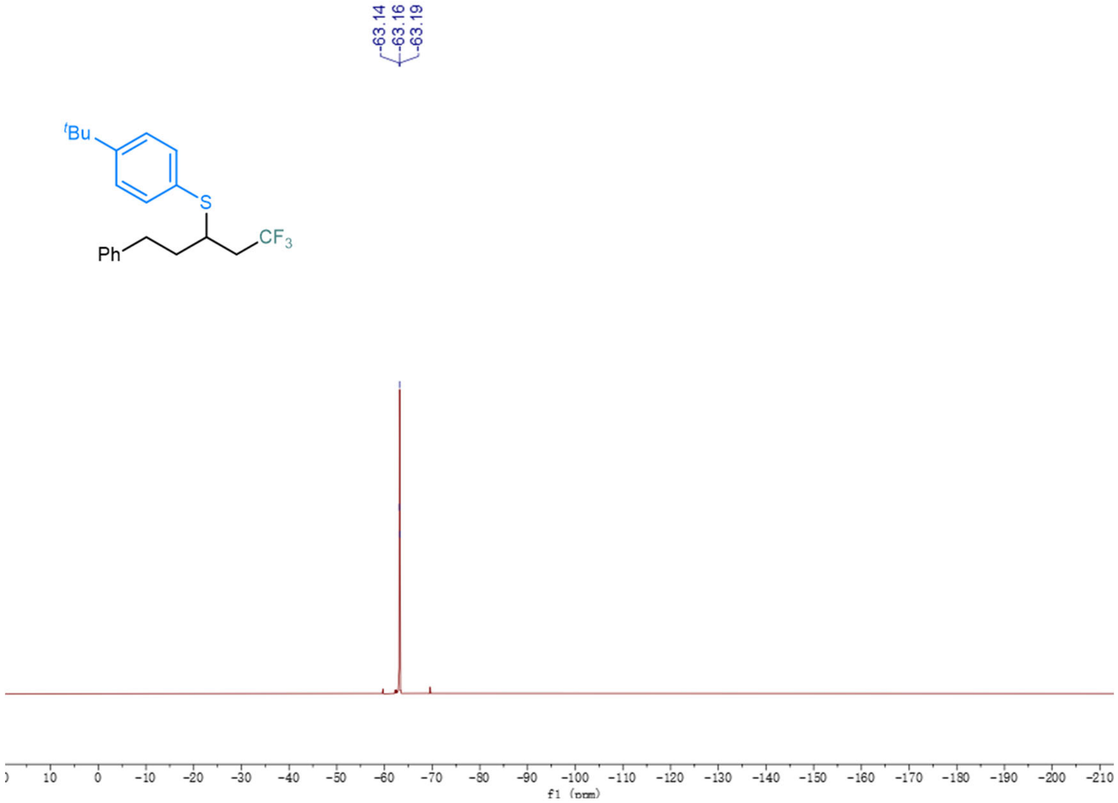

^{19}F NMR spectrum (471 MHz, Chloroform-*d*) of **37**

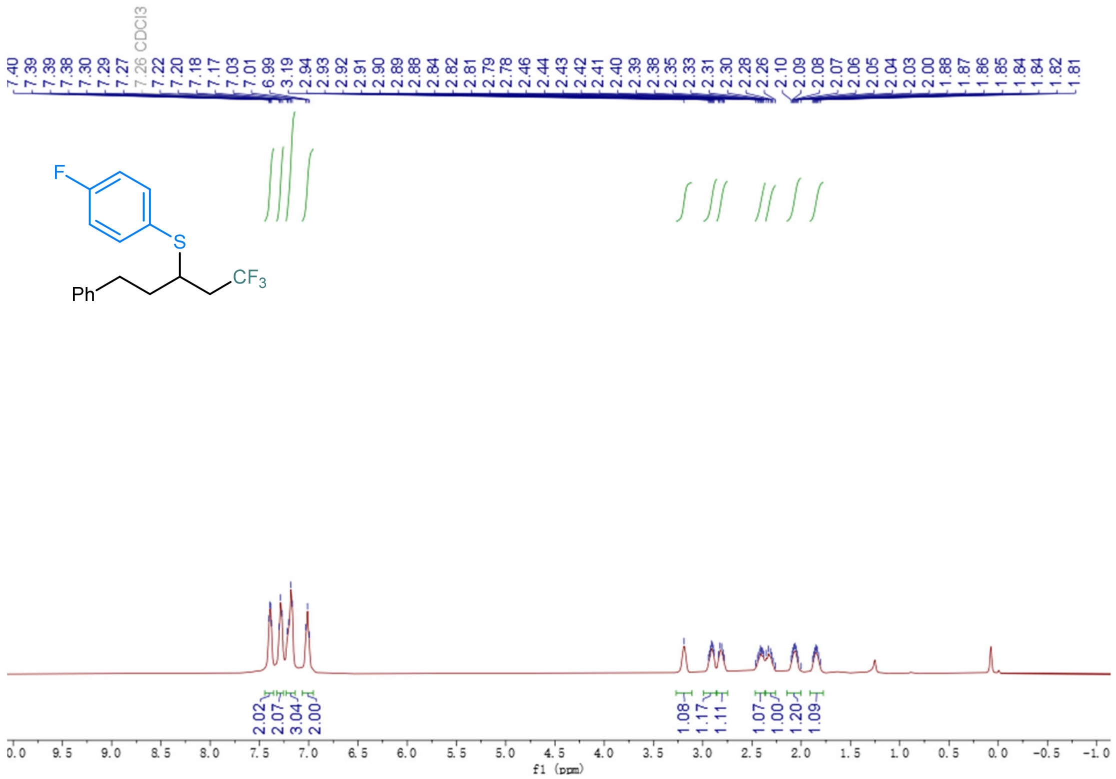

^{1}H NMR spectrum (500 MHz, Chloroform-*d*) of **38**

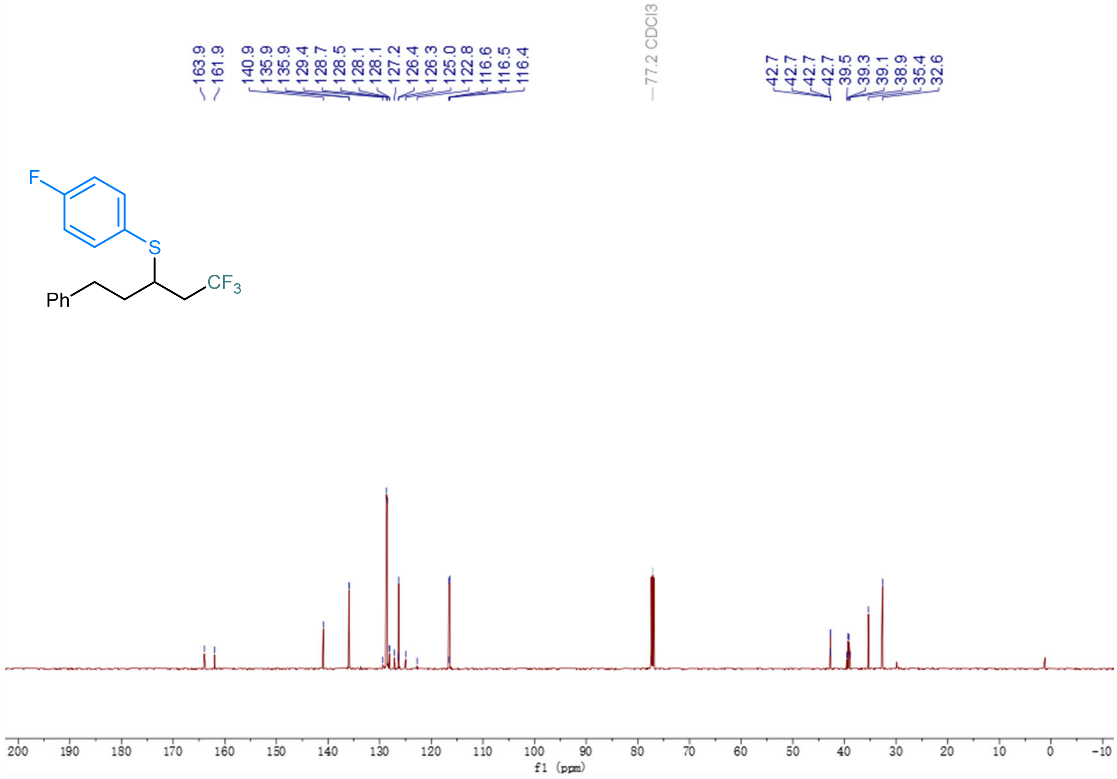

^{13}C NMR spectrum (126 MHz, Chloroform-*d*) of **38**

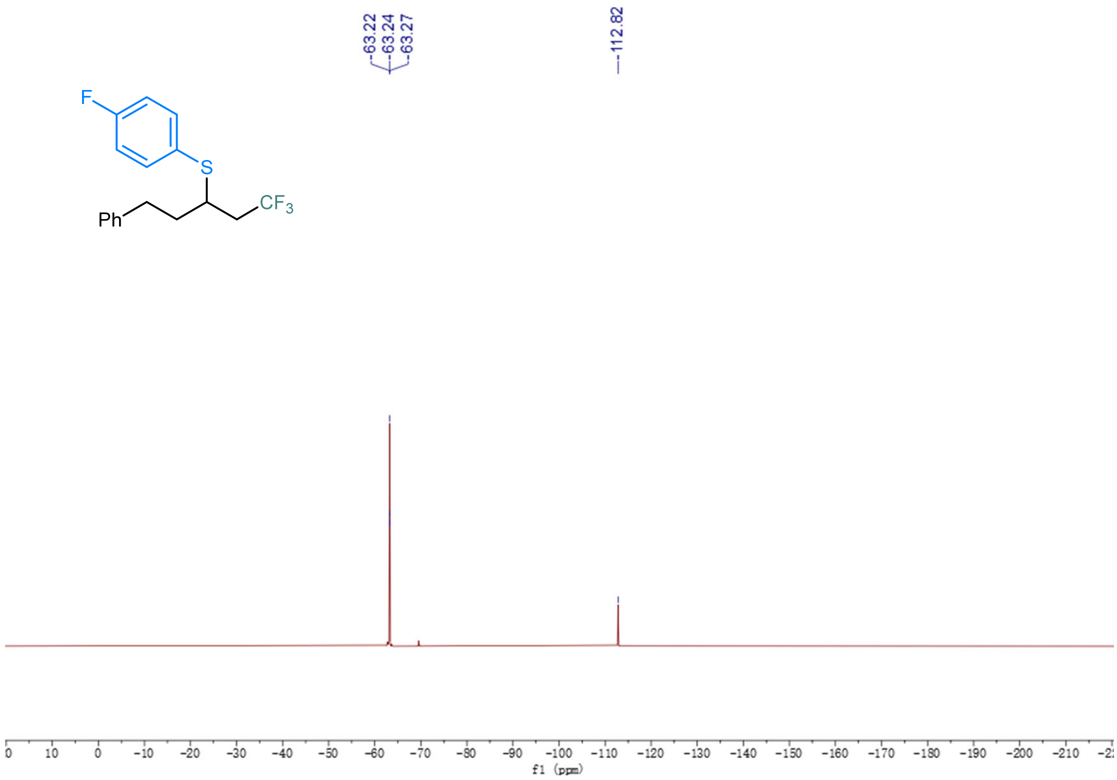

^{19}F NMR spectrum (471 MHz, Chloroform-*d*) of **38**

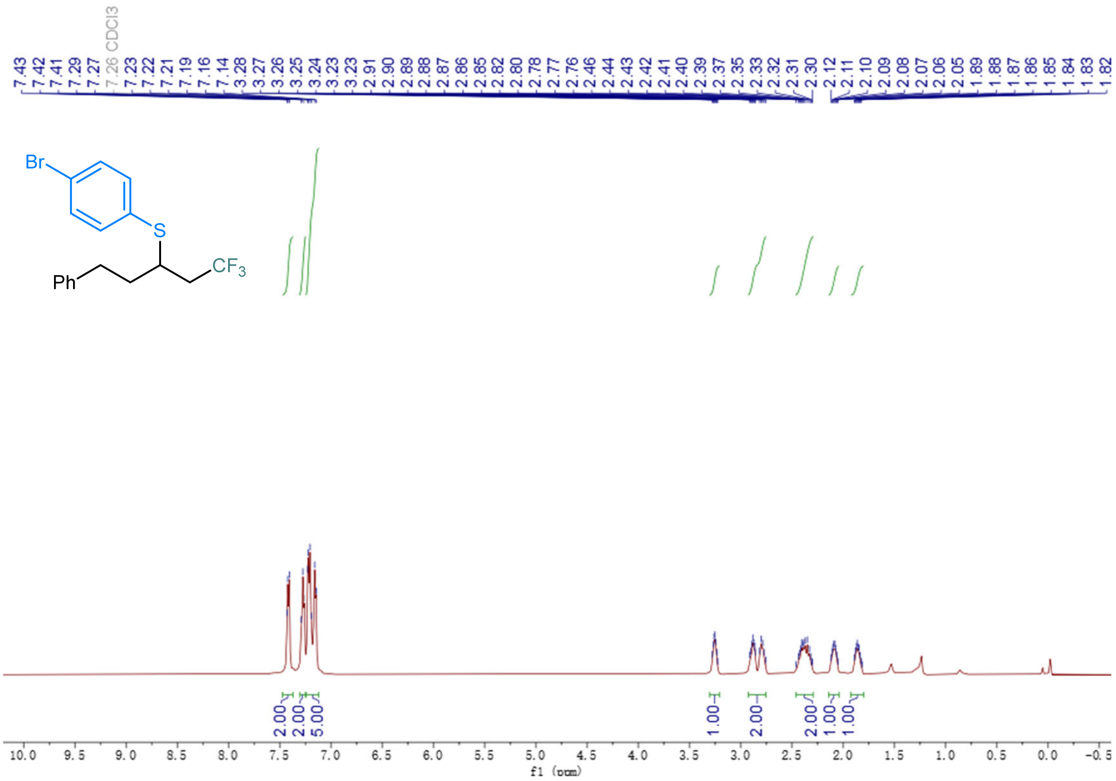

^{1}H NMR spectrum (500 MHz, Chloroform-*d*) of **39**

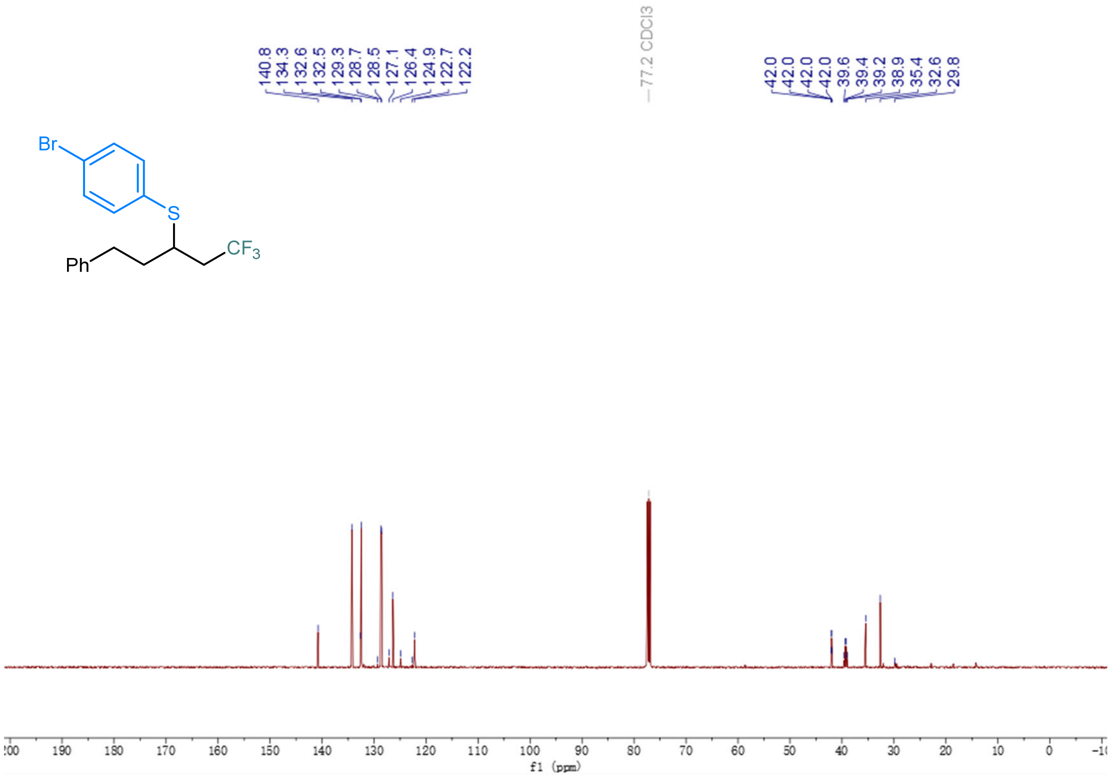

^{13}C NMR spectrum (126 MHz, Chloroform-*d*) of **39**

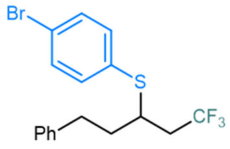

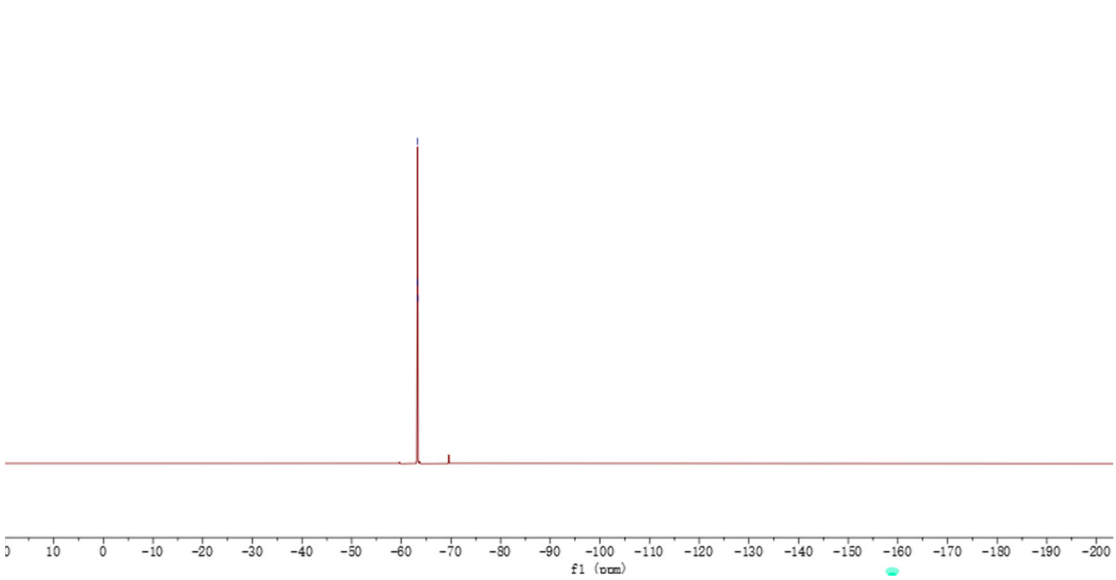

^{19}F NMR spectrum (471 MHz, Chloroform-*d*) of **39**

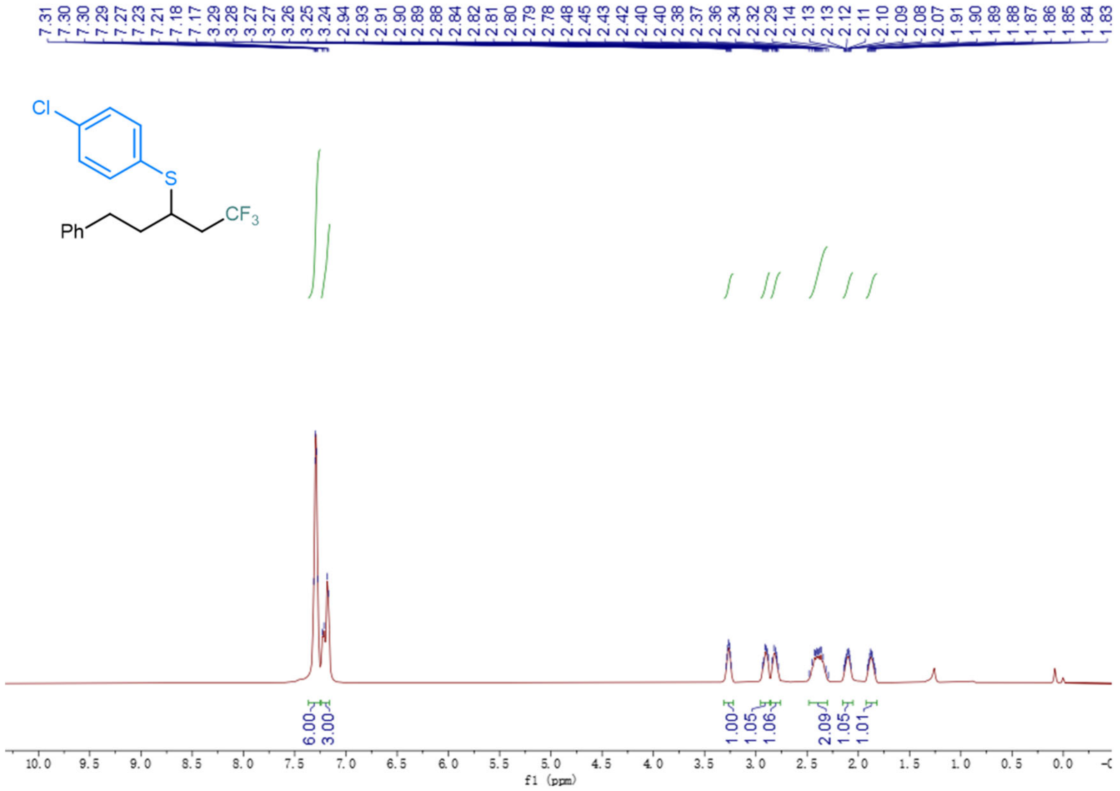

^{1}H NMR spectrum (500 MHz, Chloroform-*d*) of **40**

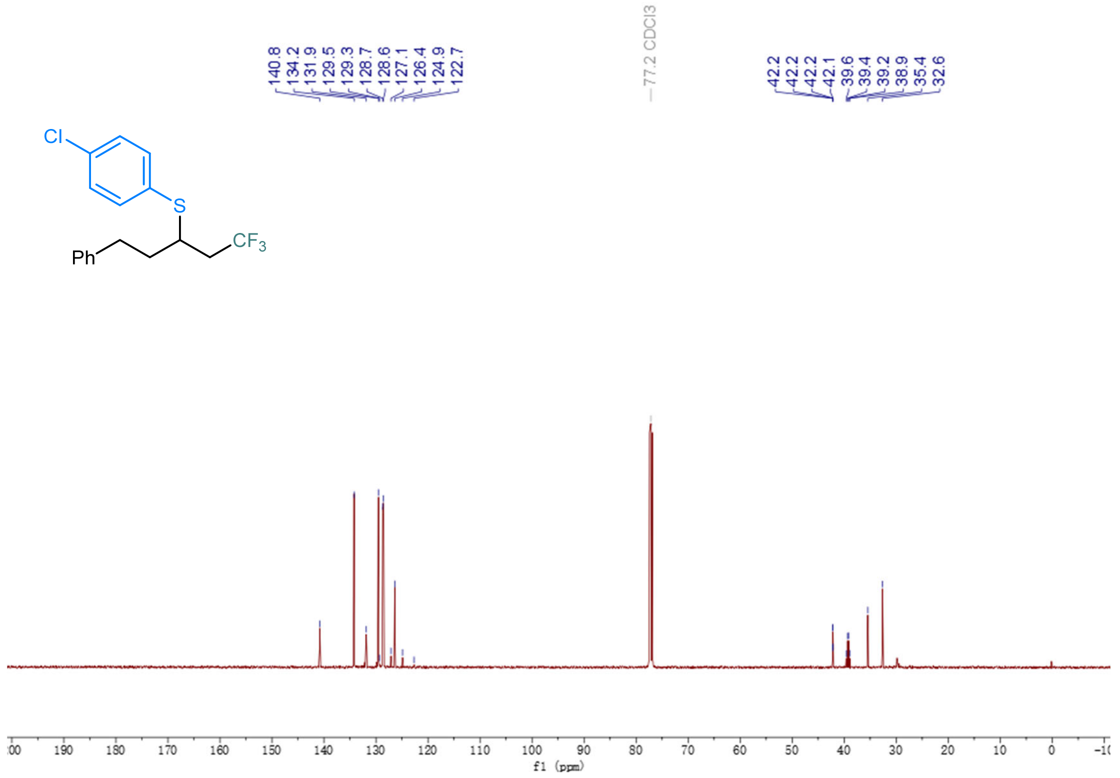

^{13}C NMR spectrum (126 MHz, Chloroform-*d*) of **40**

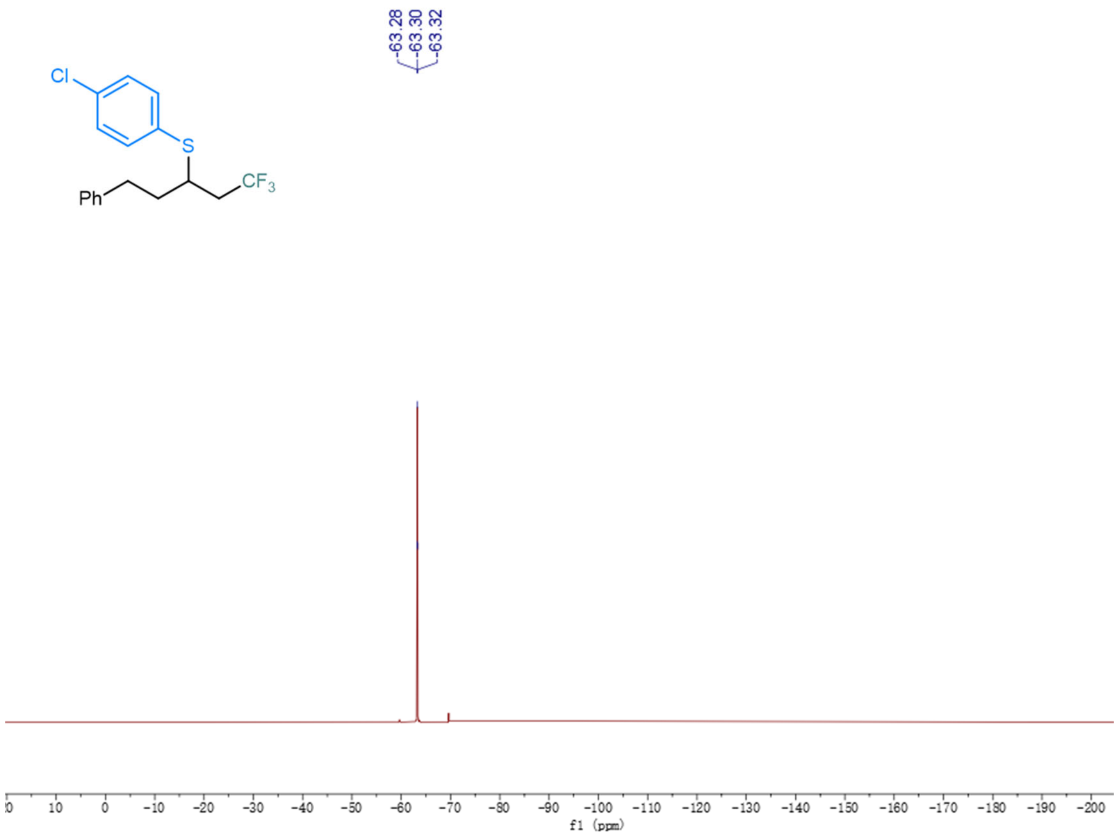

^{19}F NMR spectrum (471 MHz, Chloroform-*d*) of **40**

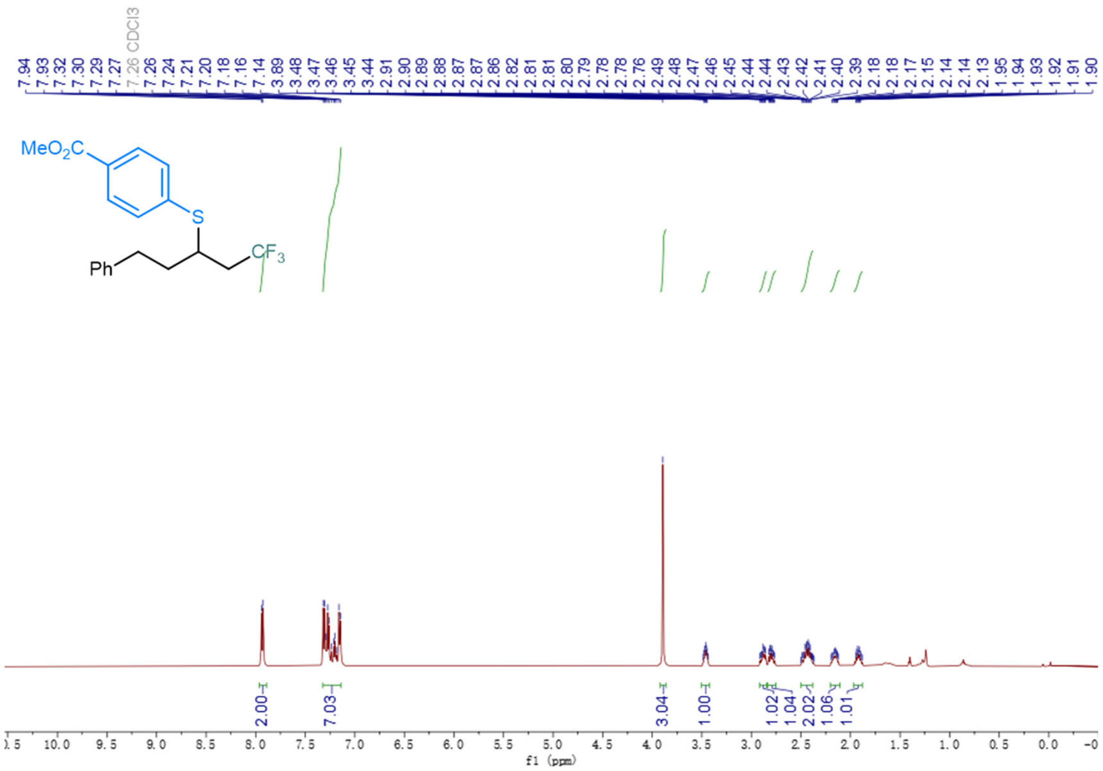

^{1}H NMR spectrum (500 MHz, Chloroform-*d*) of **41**

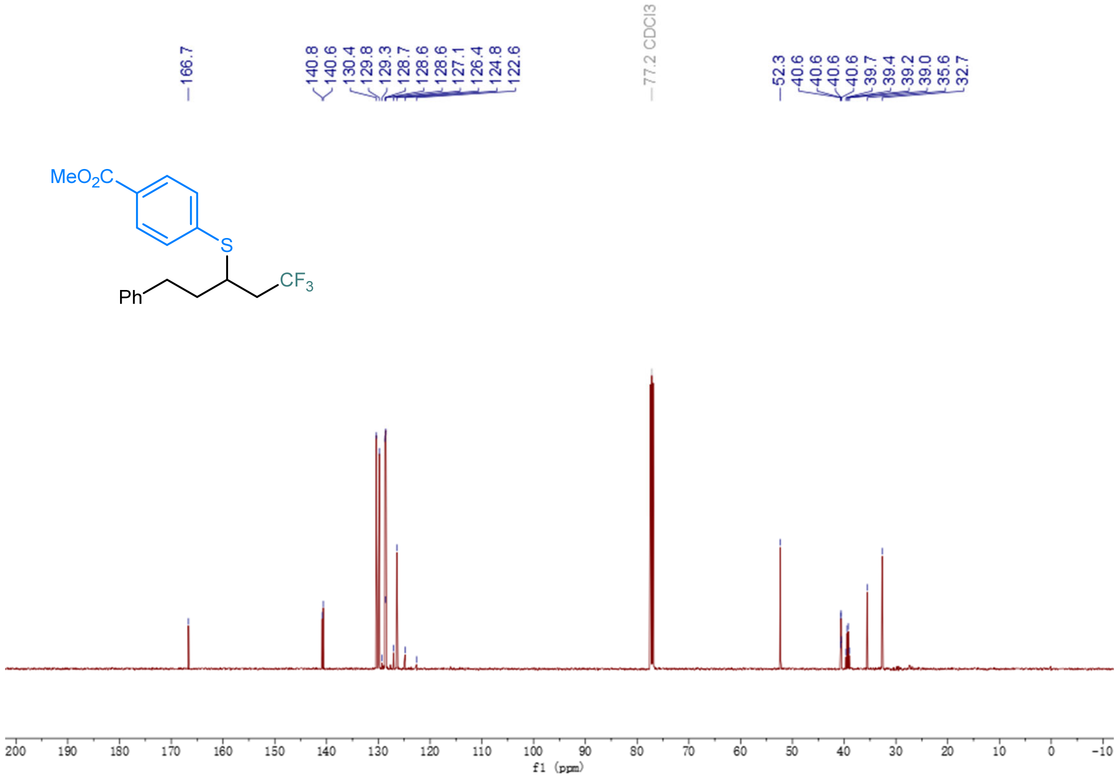

^{13}C NMR spectrum (126 MHz, Chloroform-*d*) of **41**

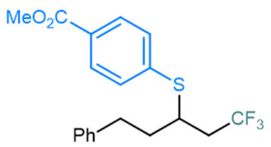

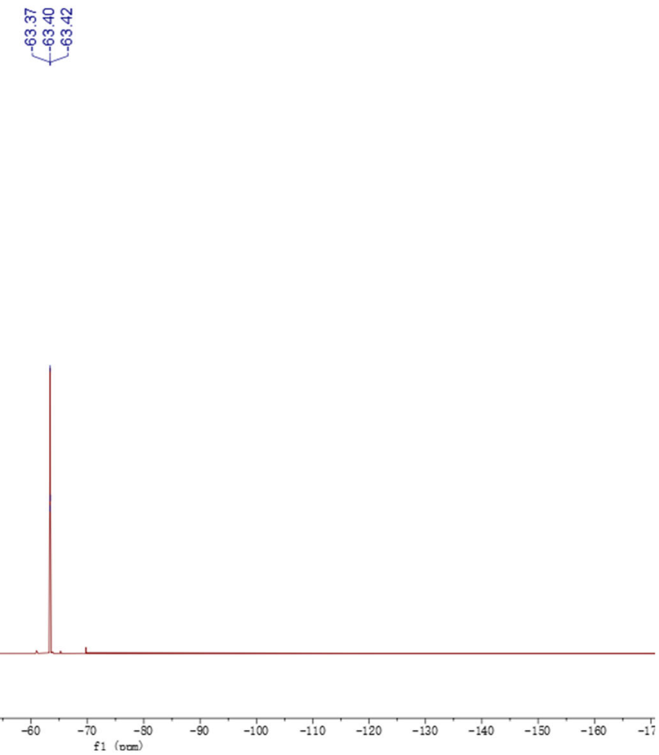

^{19}F NMR spectrum (471 MHz, Chloroform-*d*) of **41**

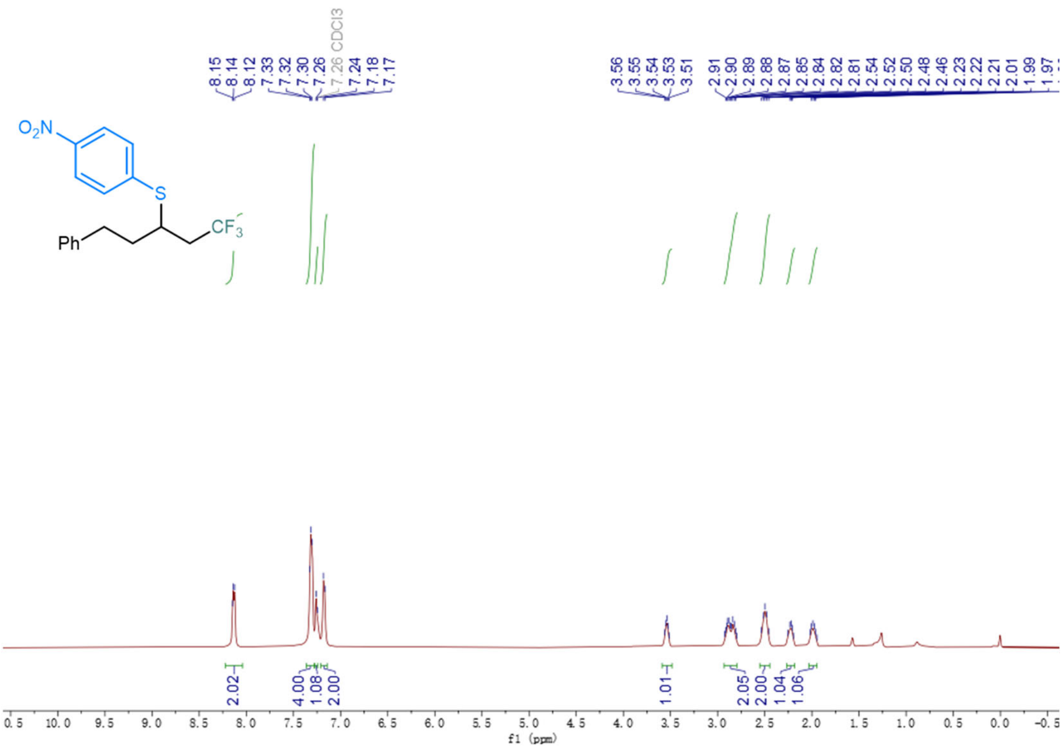

^{1}H NMR spectrum (500 MHz, Chloroform-*d*) of **42**

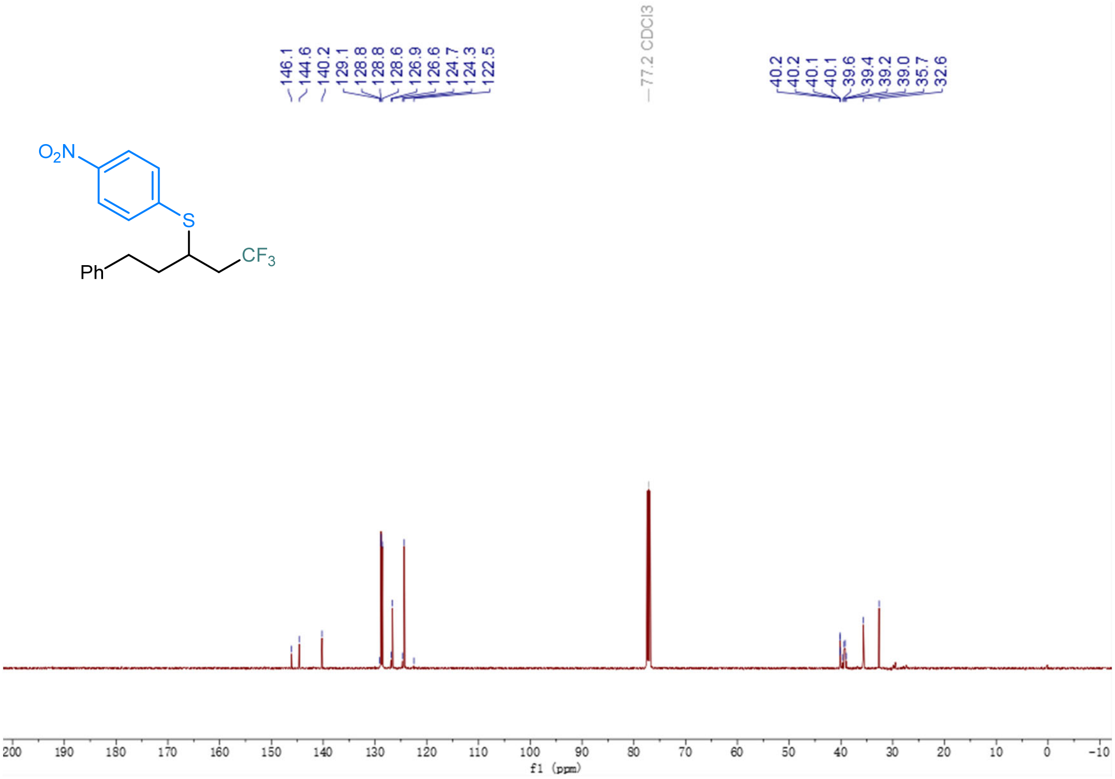

^{13}C NMR spectrum (126 MHz, Chloroform-*d*) of **42**

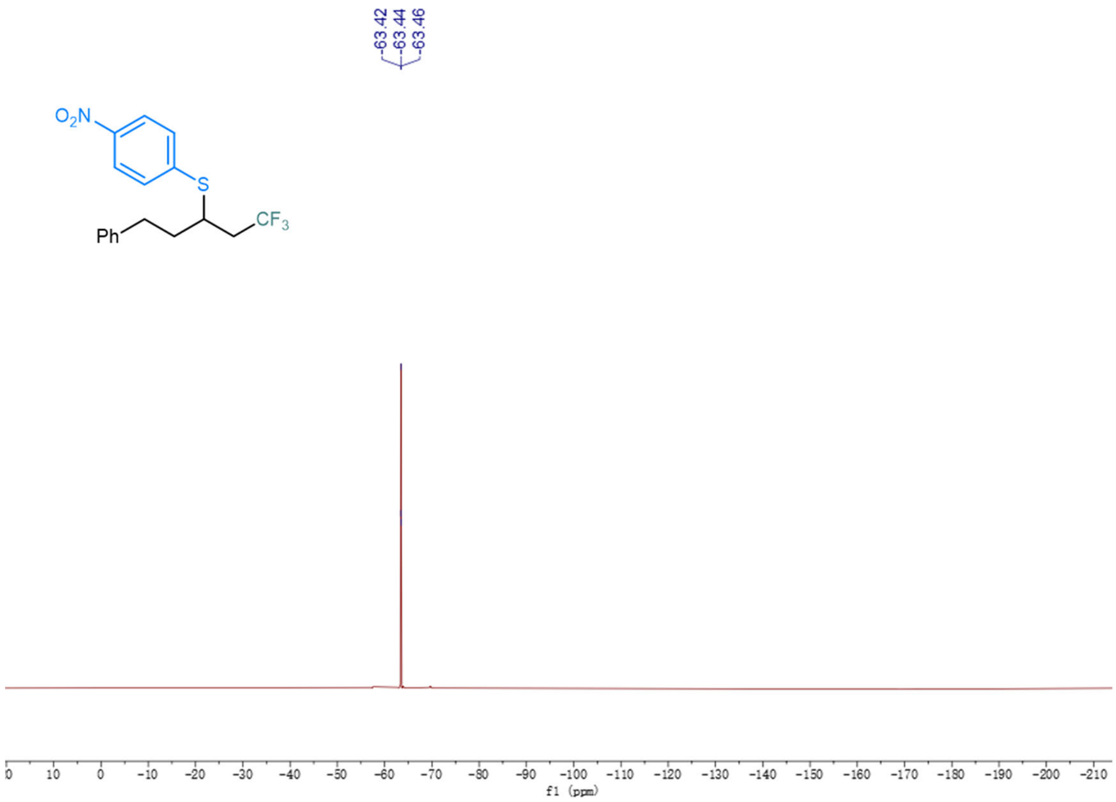

^{19}F NMR spectrum (471 MHz, Chloroform-*d*) of **42**

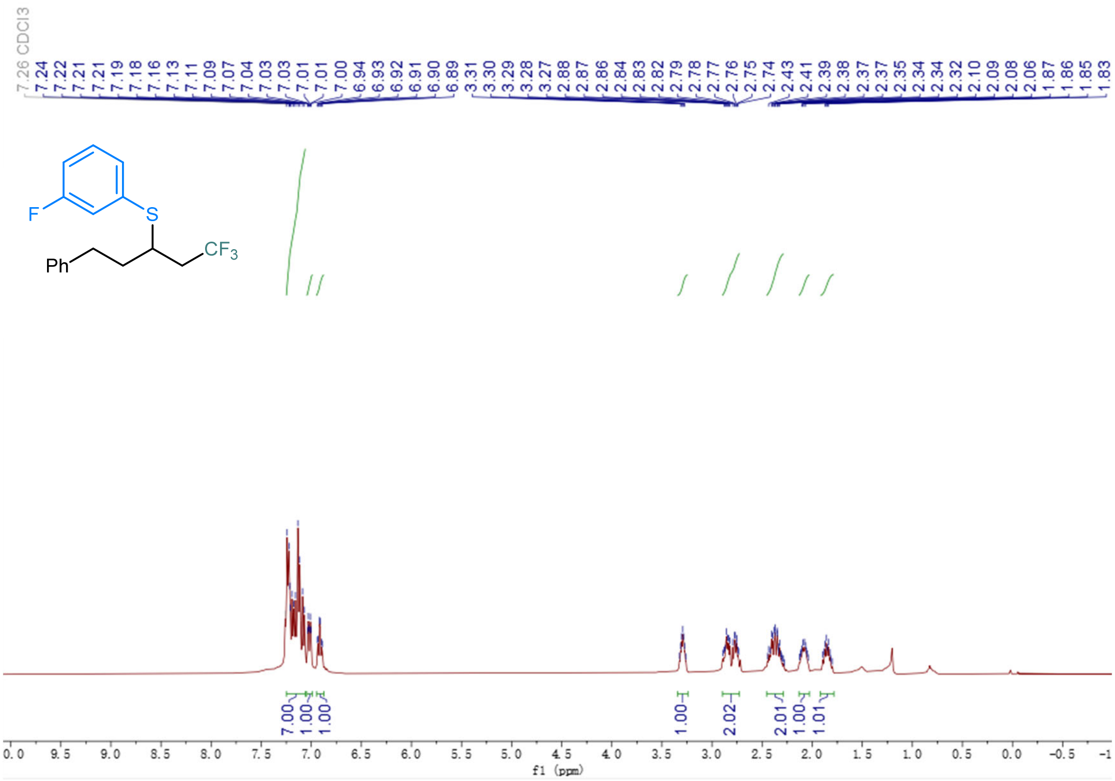

^{1}H NMR spectrum (500 MHz, Chloroform-*d*) of **43**

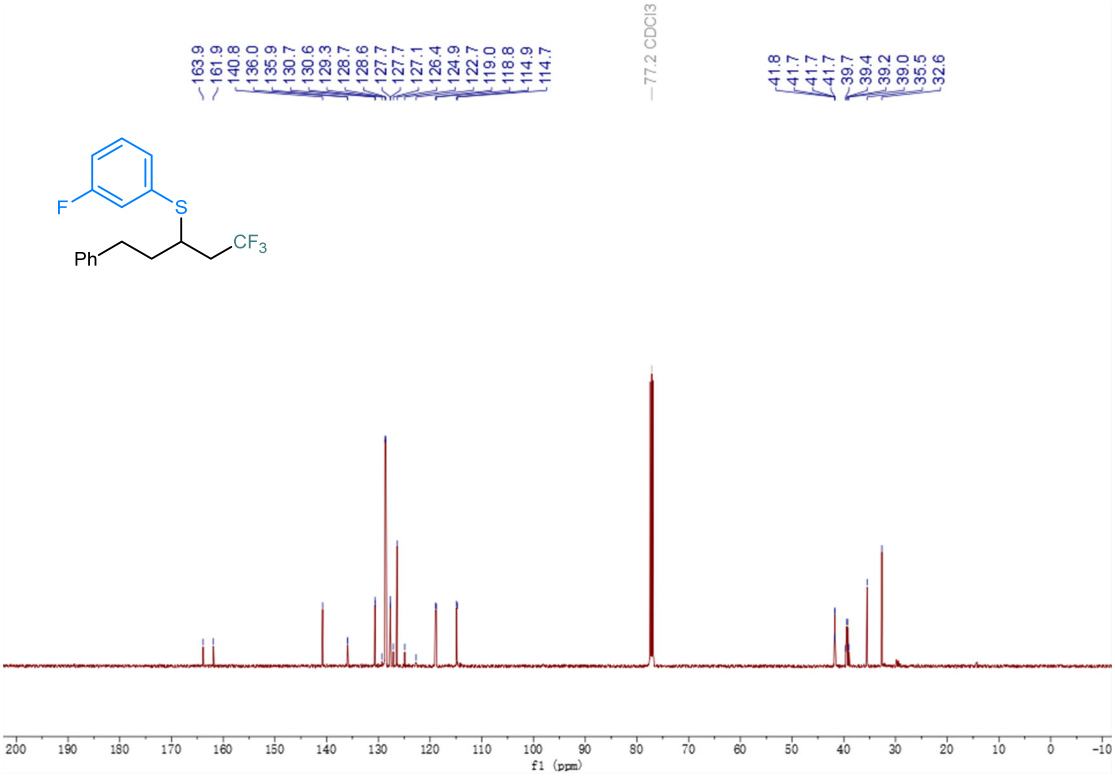

^{13}C NMR spectrum (126 MHz, Chloroform-*d*) of **43**

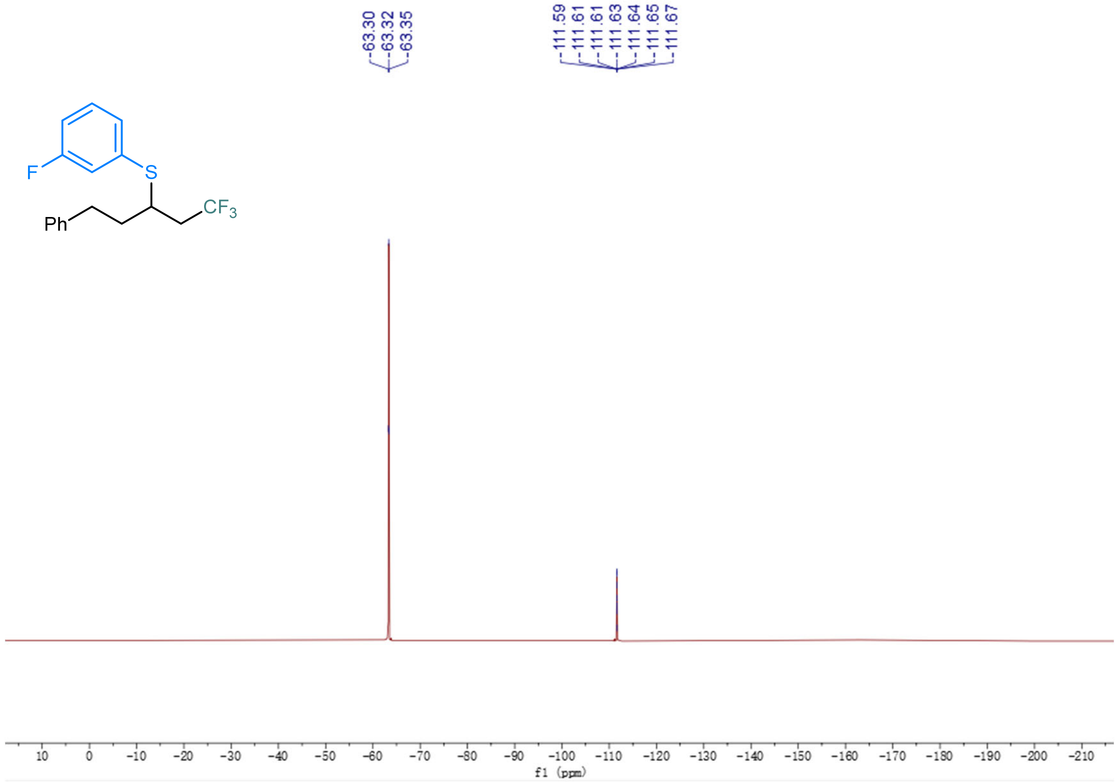

^{19}F NMR spectrum (471 MHz, Chloroform-*d*) of **43**

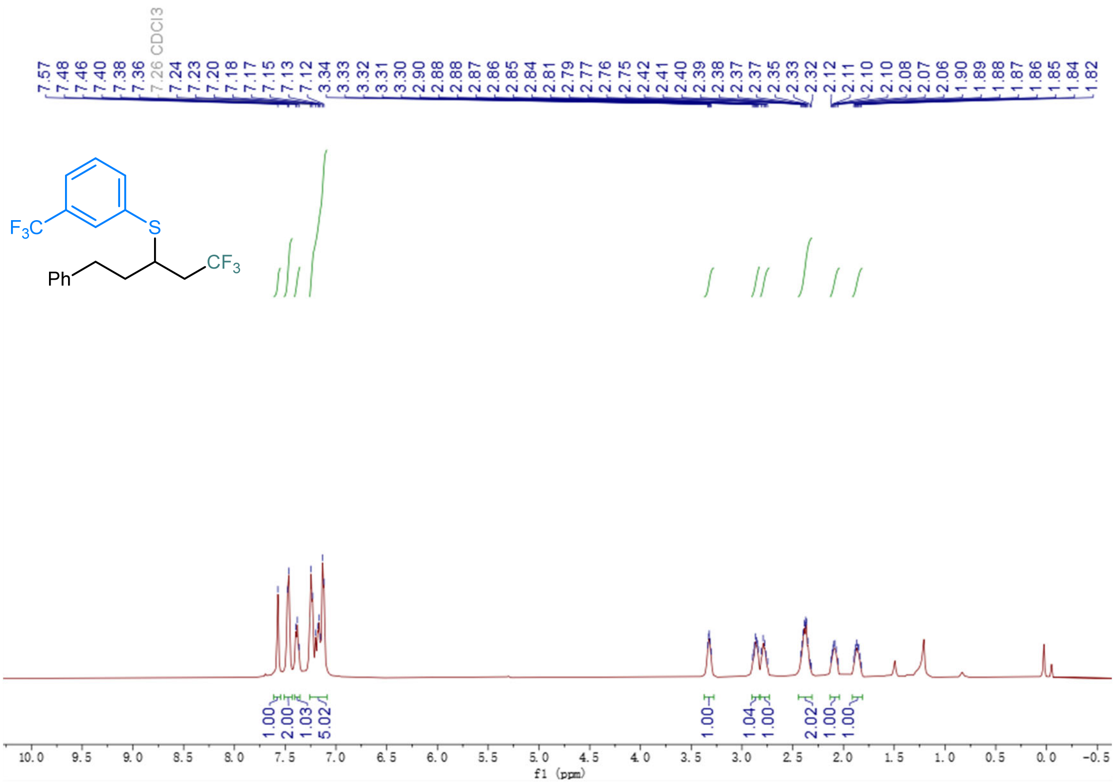

^{1}H NMR spectrum (500 MHz, Chloroform-*d*) of **44**

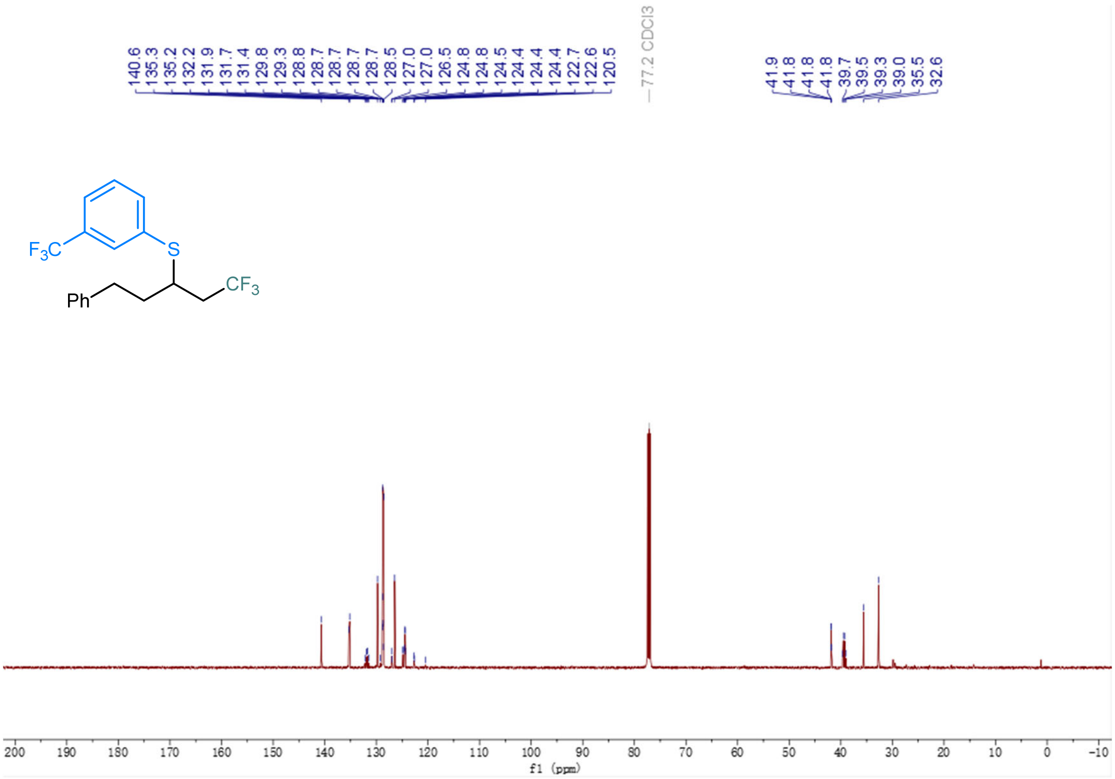

^{13}C NMR spectrum (126 MHz, Chloroform-*d*) of **44**

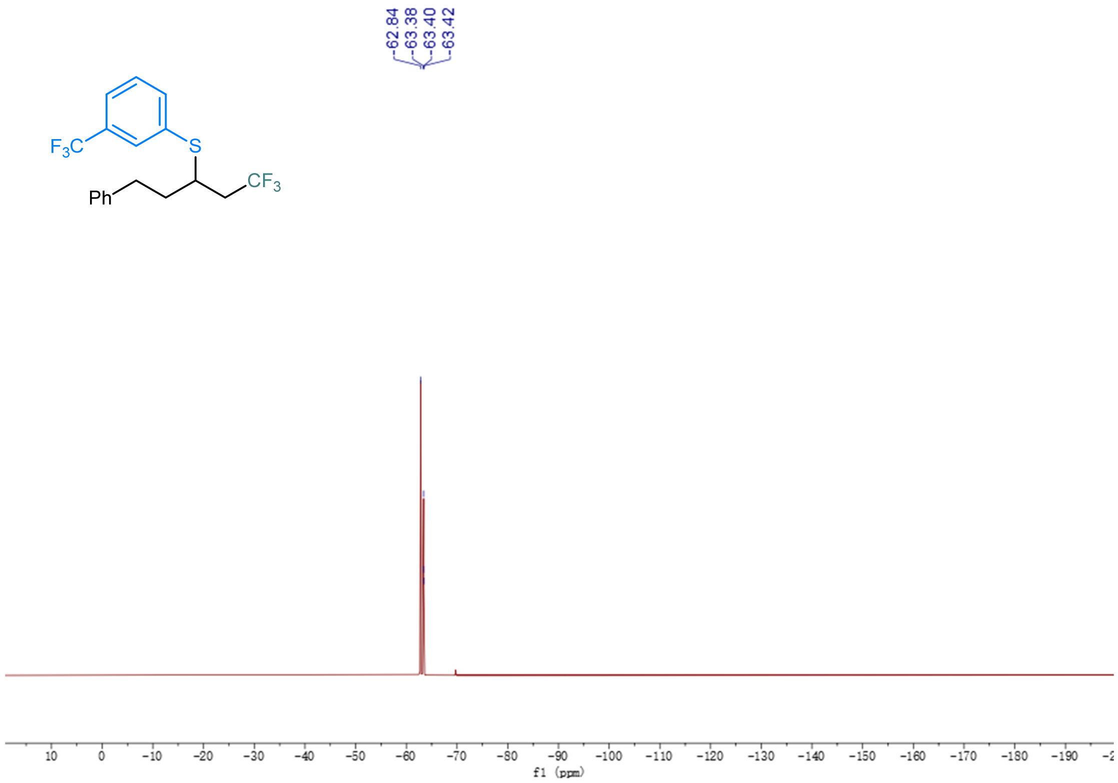

^{19}F NMR spectrum (471 MHz, Chloroform-*d*) of **44**

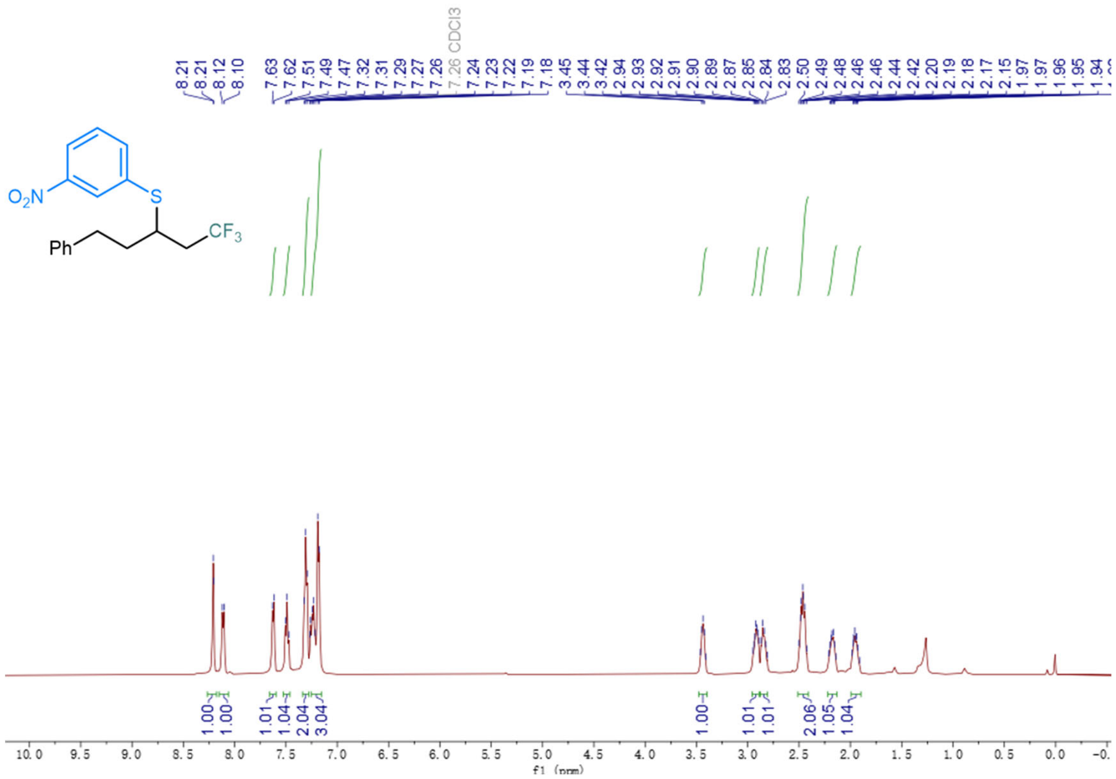

^{1}H NMR spectrum (500 MHz, Chloroform-*d*) of **45**

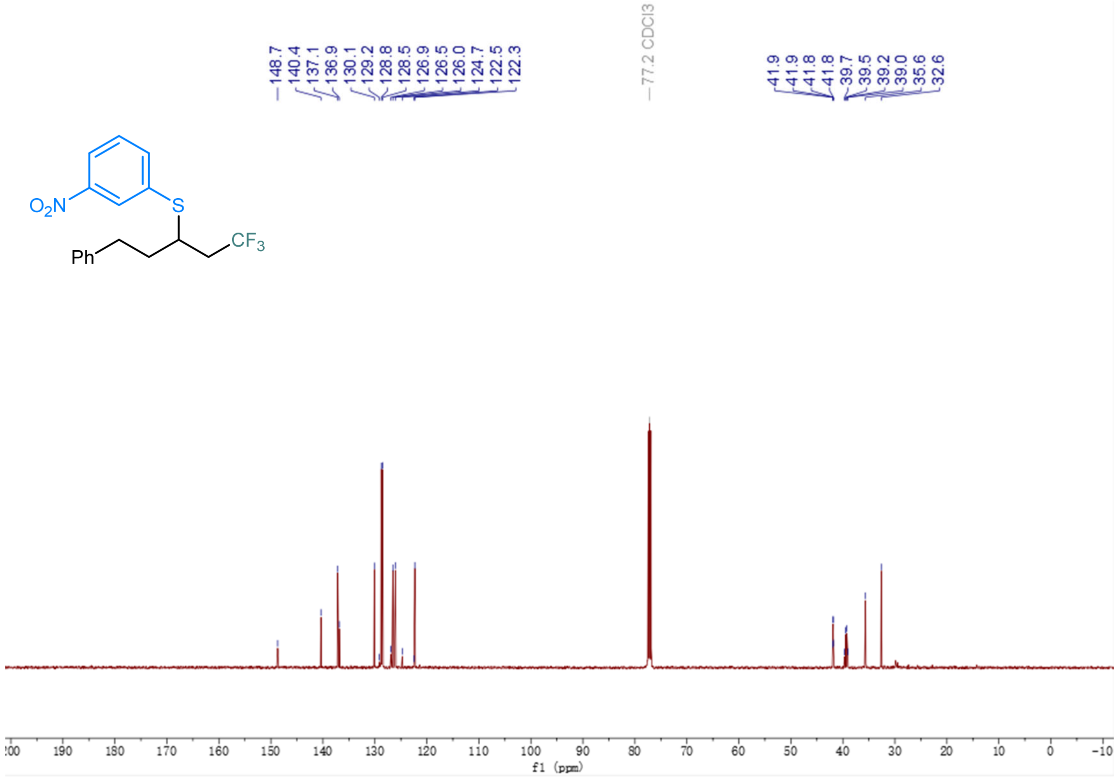

^{13}C NMR spectrum (126 MHz, Chloroform-*d*) of **45**

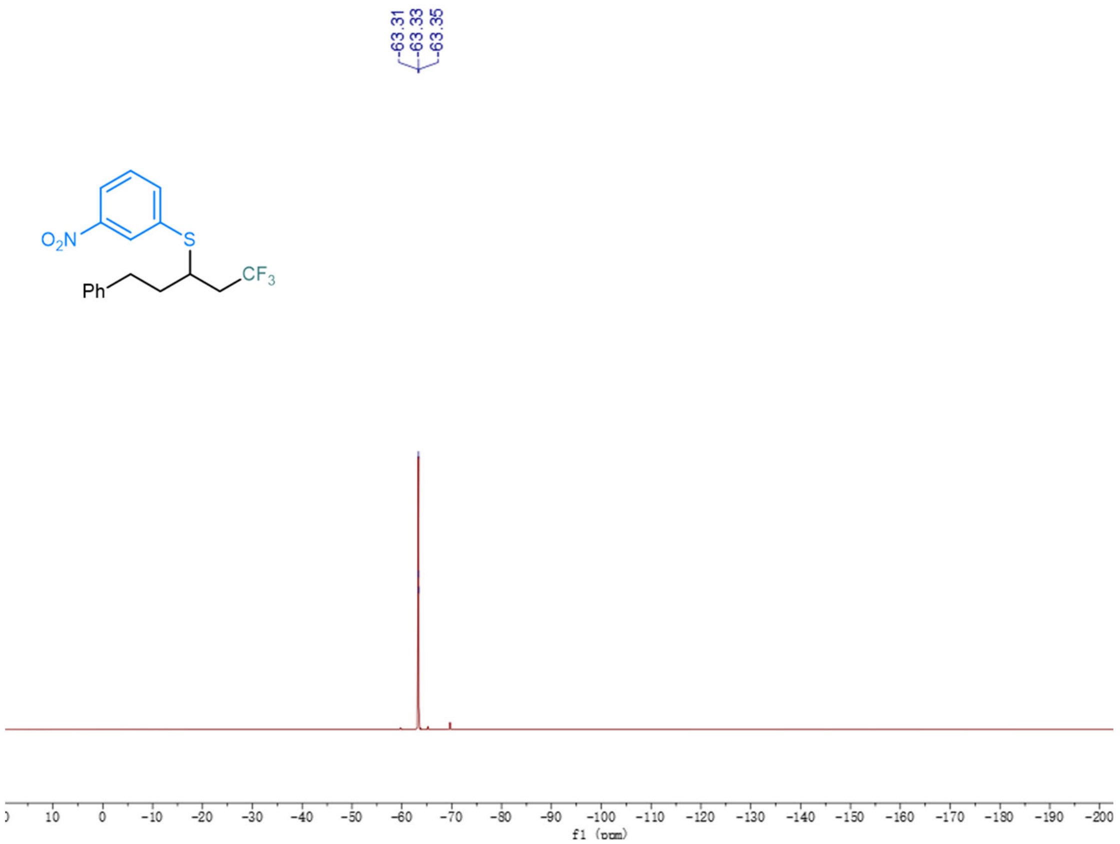

^{19}F NMR spectrum (471 MHz, Chloroform-*d*) of **45**

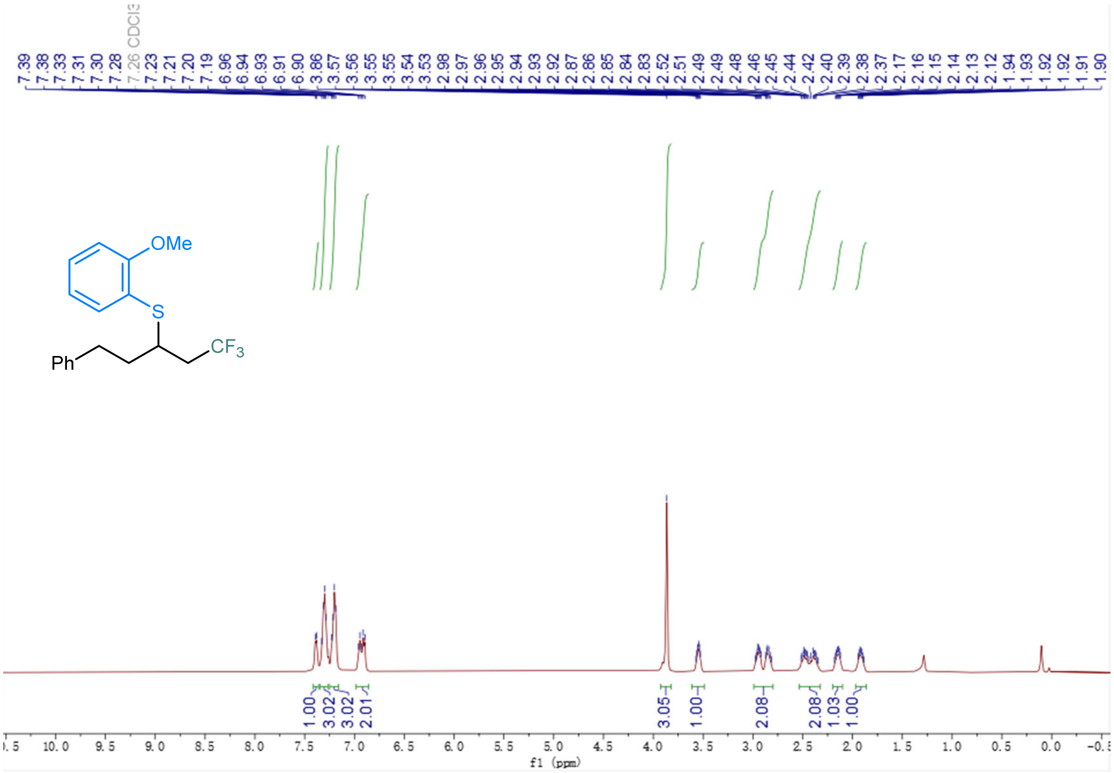

^{1}H NMR spectrum (500 MHz, Chloroform-*d*) of **46**

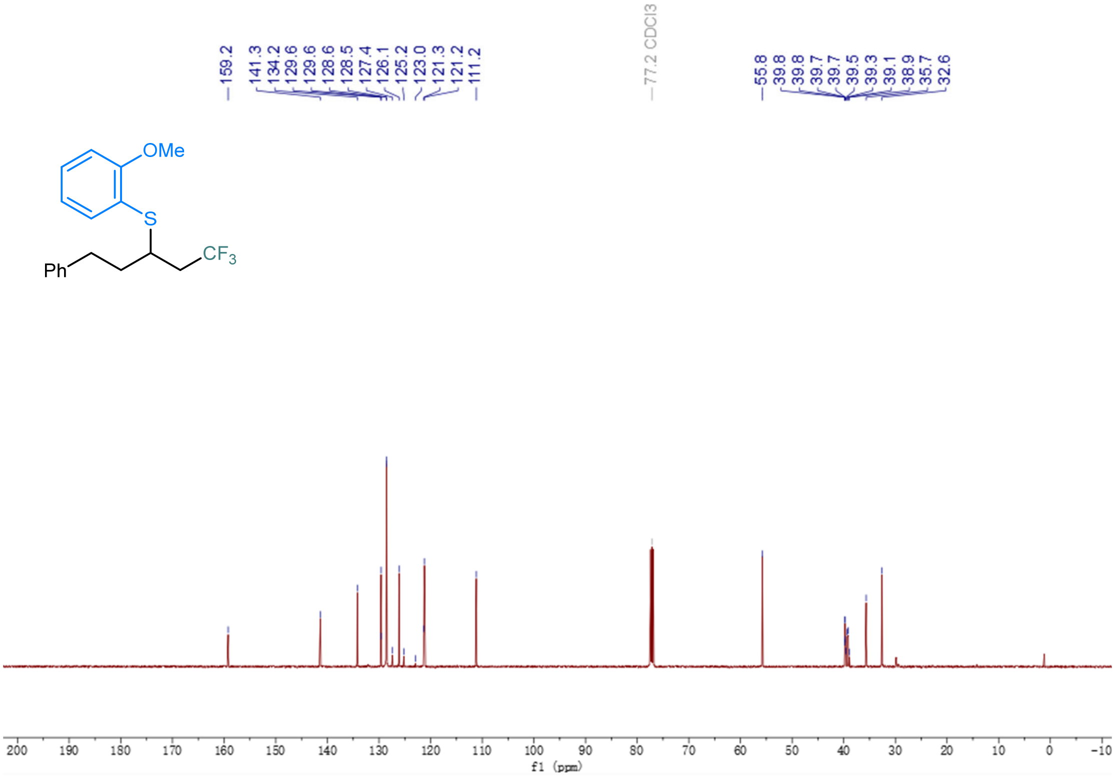

^{13}C NMR spectrum (126 MHz, Chloroform-*d*) of **46**

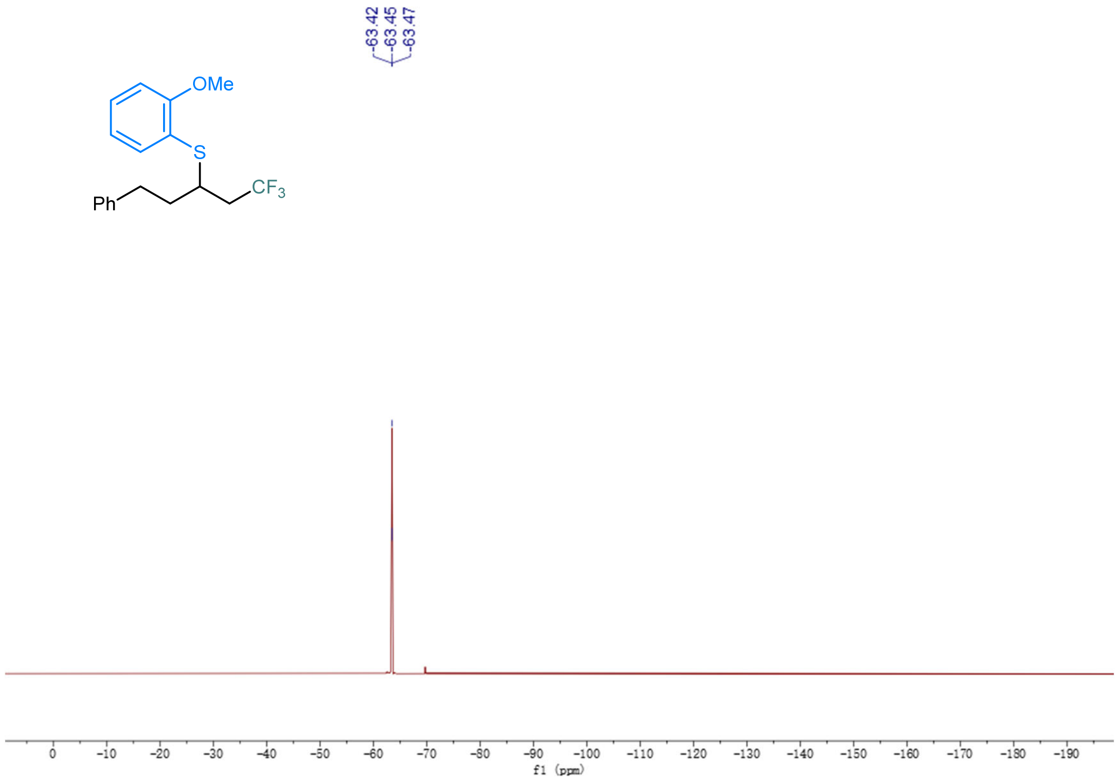

^{19}F NMR spectrum (471 MHz, Chloroform-*d*) of **46**

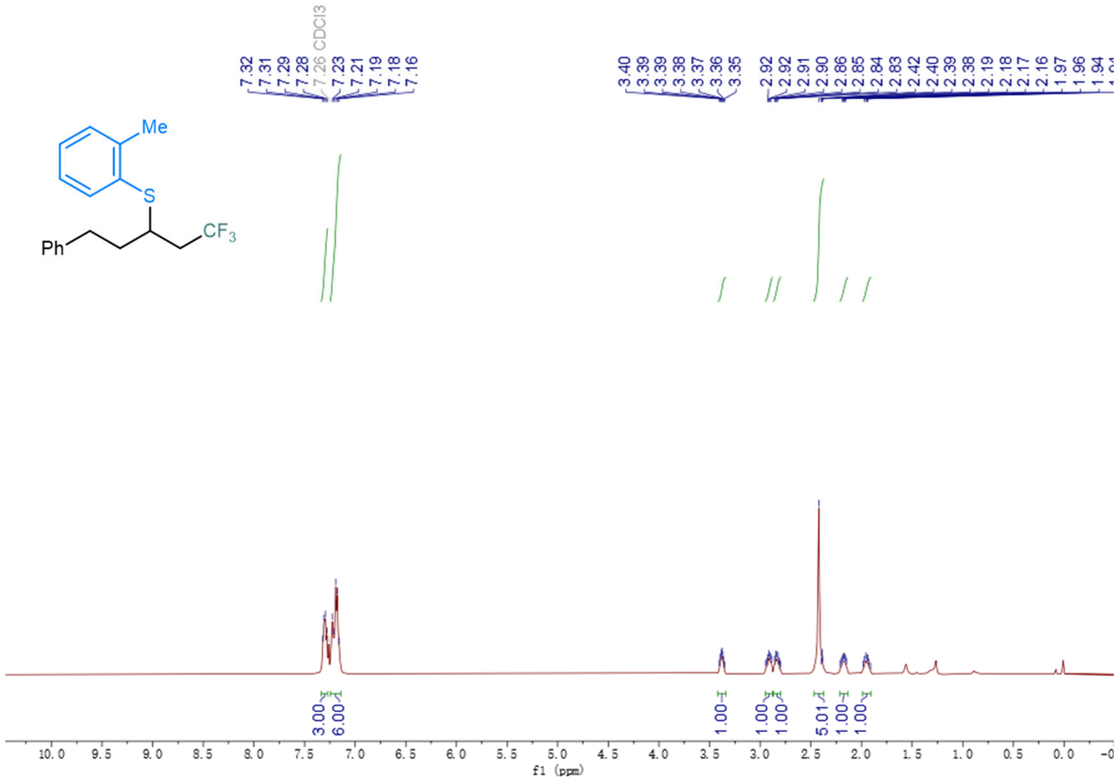

^{1}H NMR spectrum (500 MHz, Chloroform-*d*) of **47**

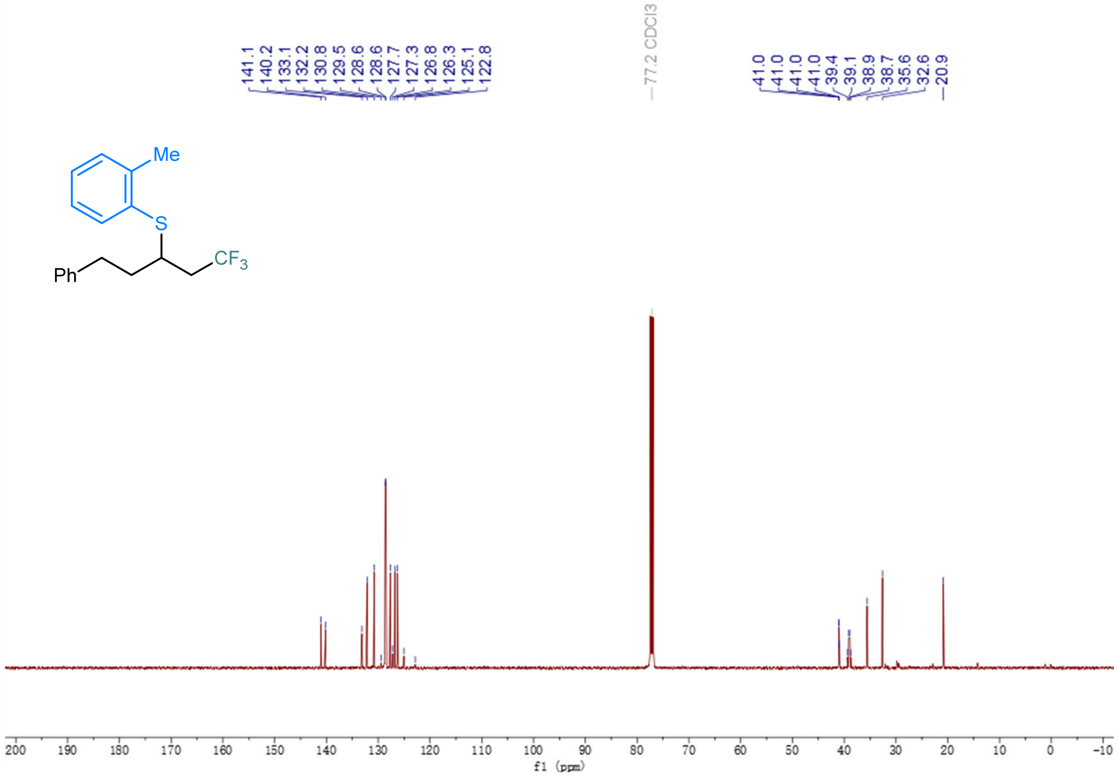

^{13}C NMR spectrum (126 MHz, Chloroform-*d*) of **47**

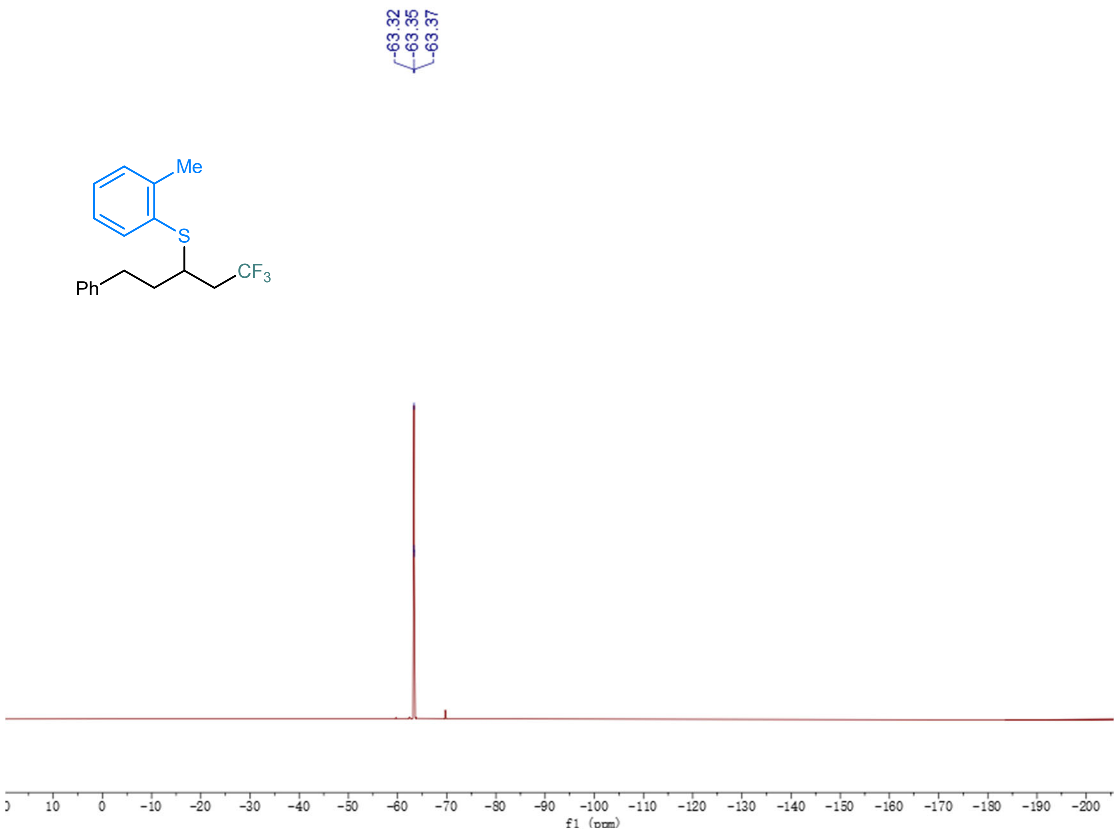

^{19}F NMR spectrum (471 MHz, Chloroform-*d*) of **47**

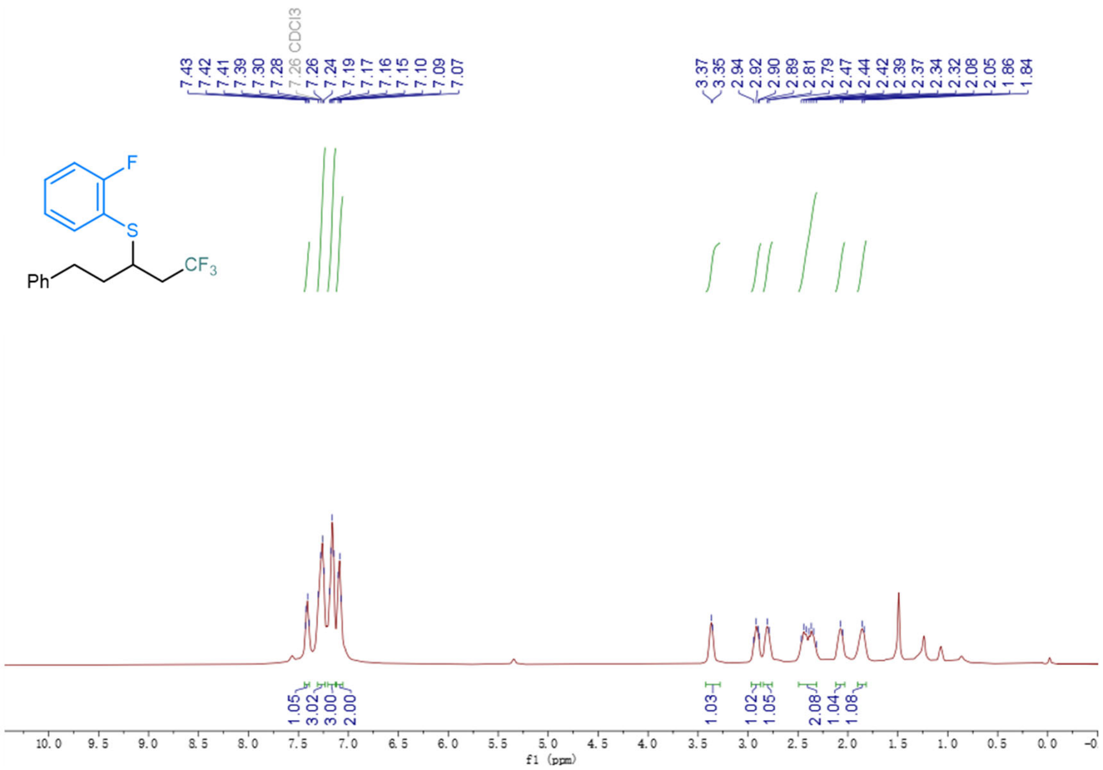

^{1}H NMR spectrum (500 MHz, Chloroform-*d*) of **48**

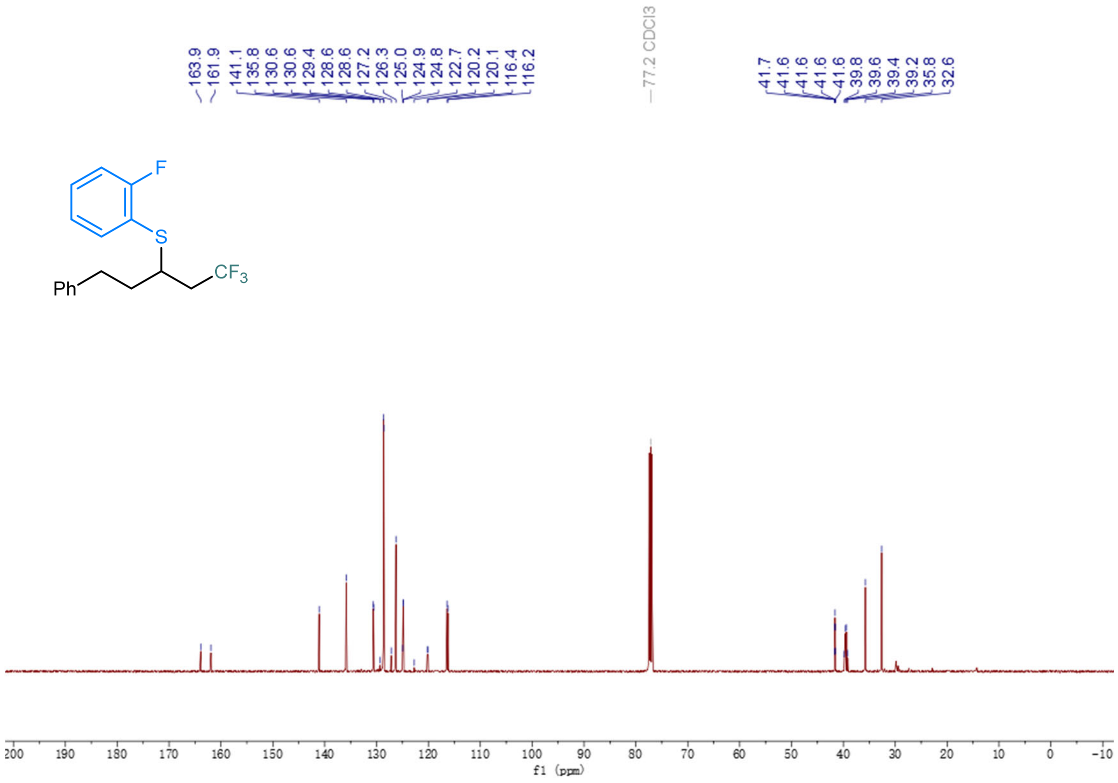

^{13}C NMR spectrum (126 MHz, Chloroform-*d*) of **48**

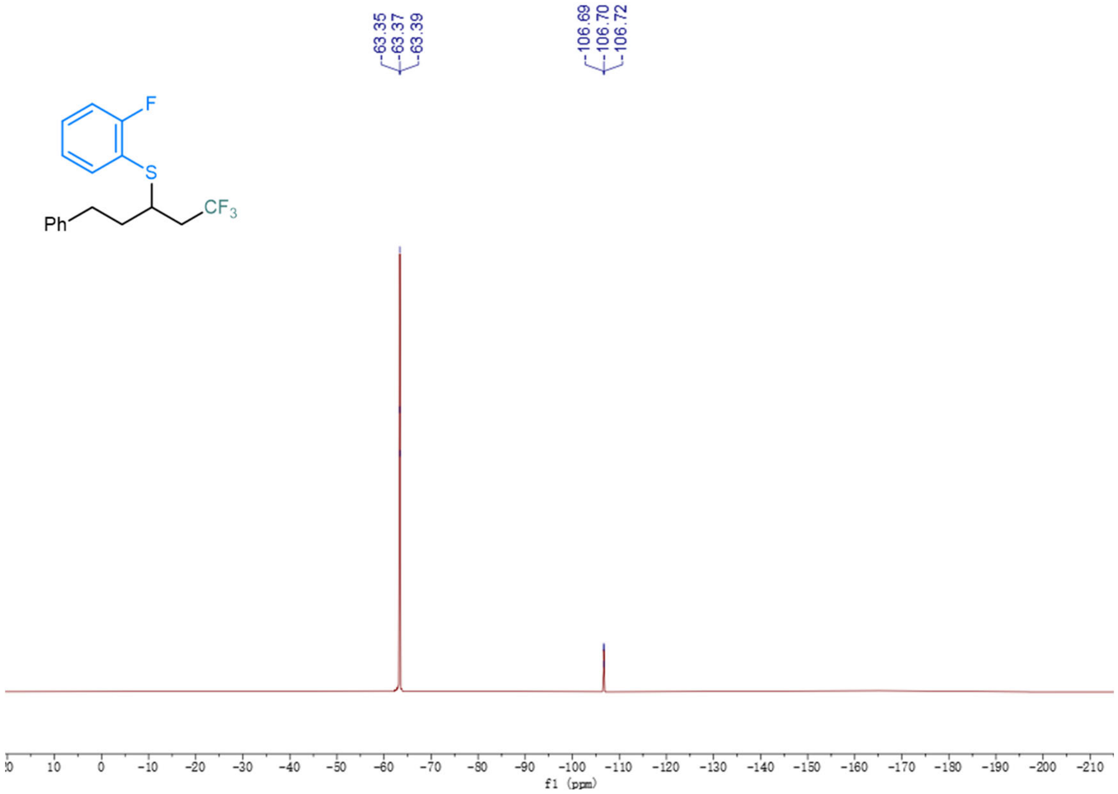

^{19}F NMR spectrum (471 MHz, Chloroform-*d*) of **48**

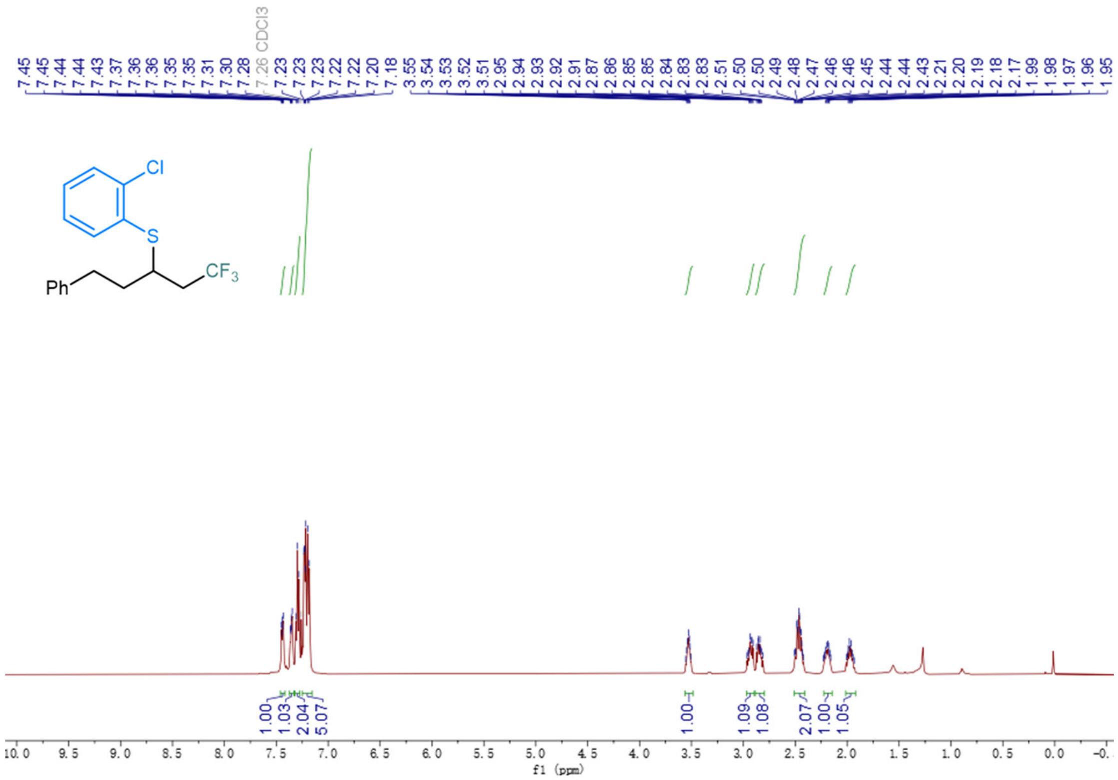

^{1}H NMR spectrum (500 MHz, Chloroform-*d*) of **49**

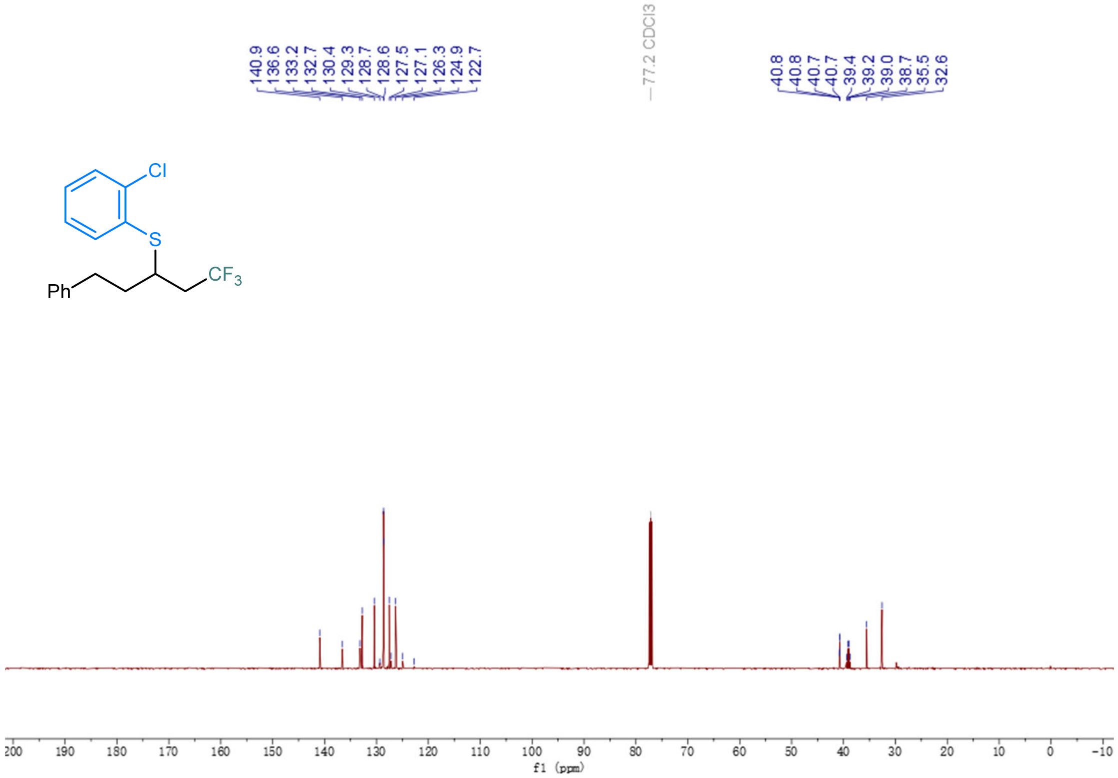

^{13}C NMR spectrum (126 MHz, Chloroform-*d*) of **49**

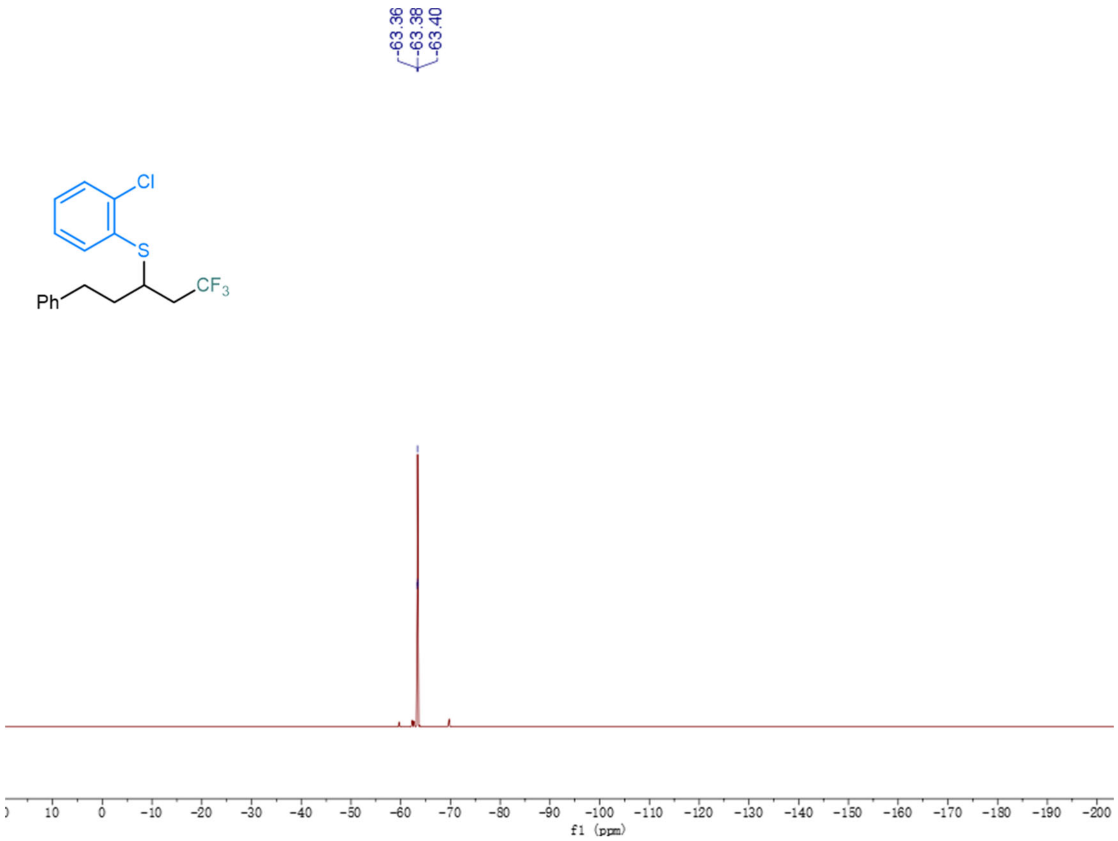

^{19}F NMR spectrum (471 MHz, Chloroform-*d*) of **49**

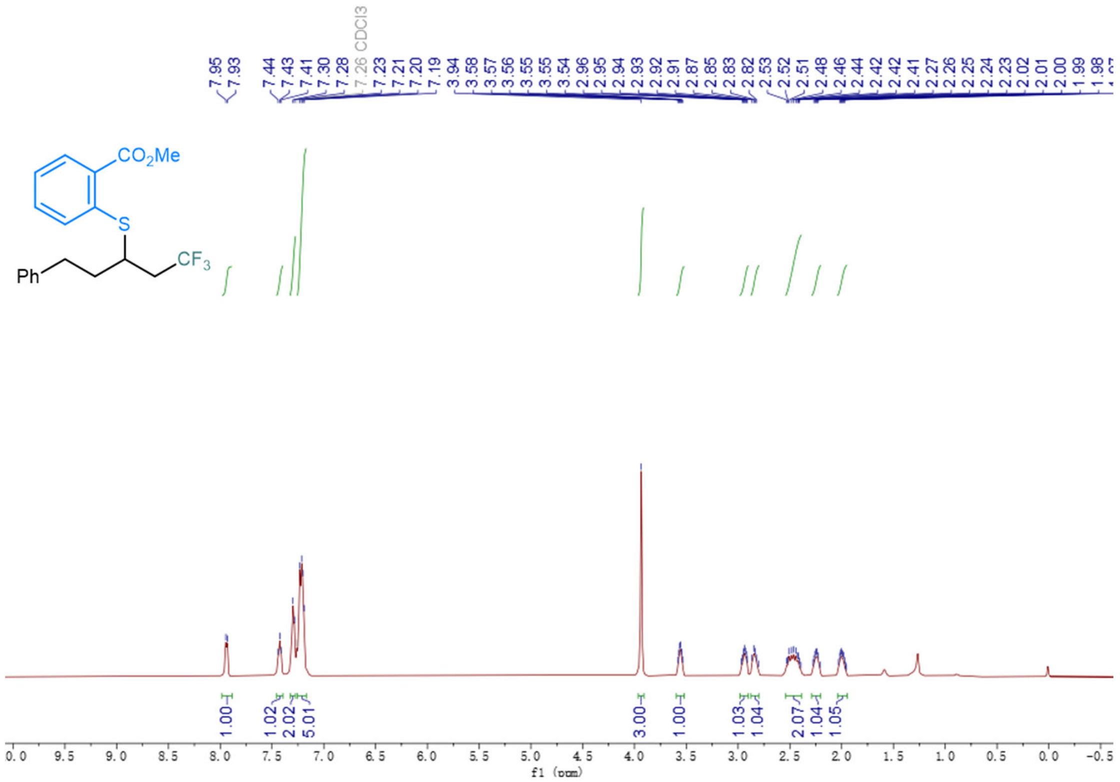

^{1}H NMR spectrum (500 MHz, Chloroform-*d*) of **50**

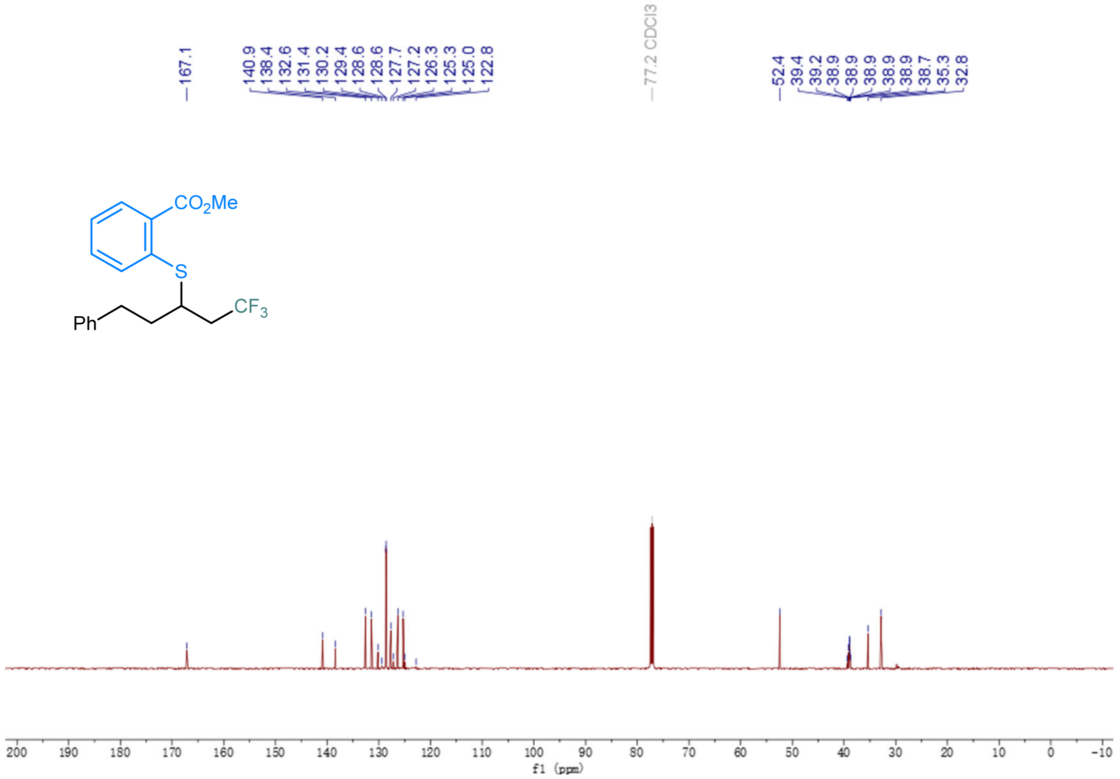

^{13}C NMR spectrum (126 MHz, Chloroform-*d*) of **50**

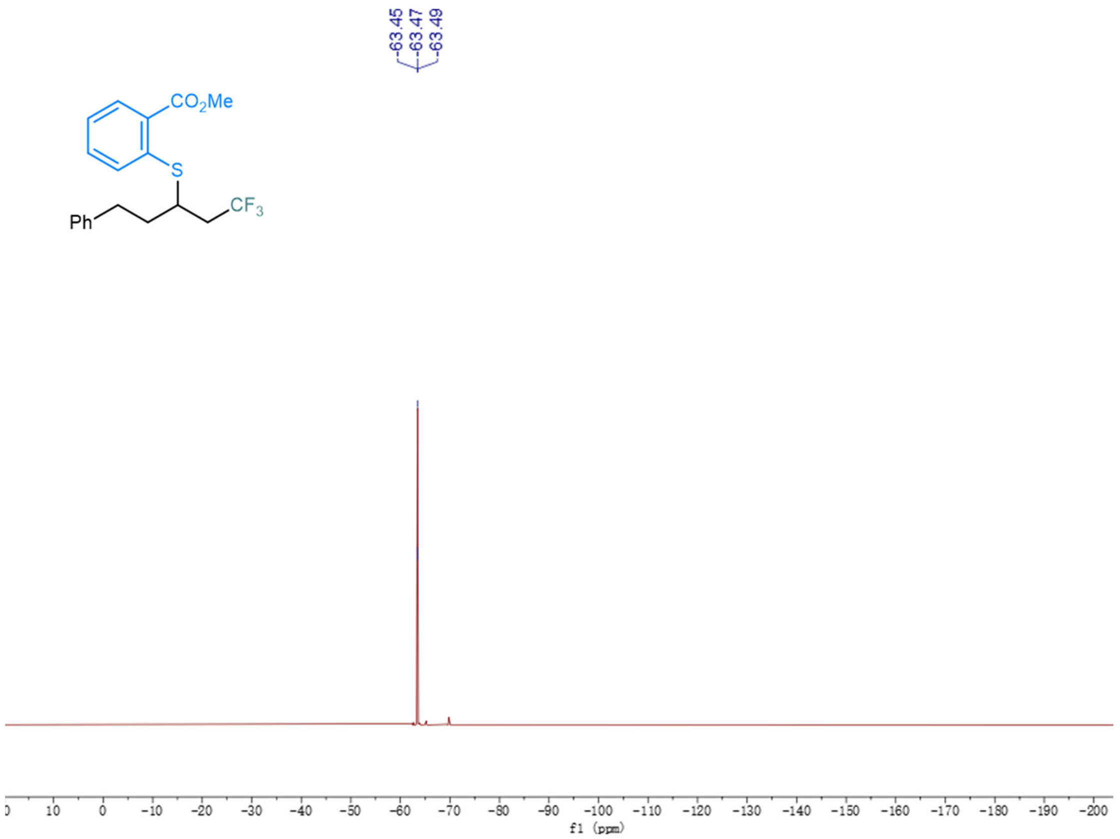

^{19}F NMR spectrum (471 MHz, Chloroform-*d*) **50**

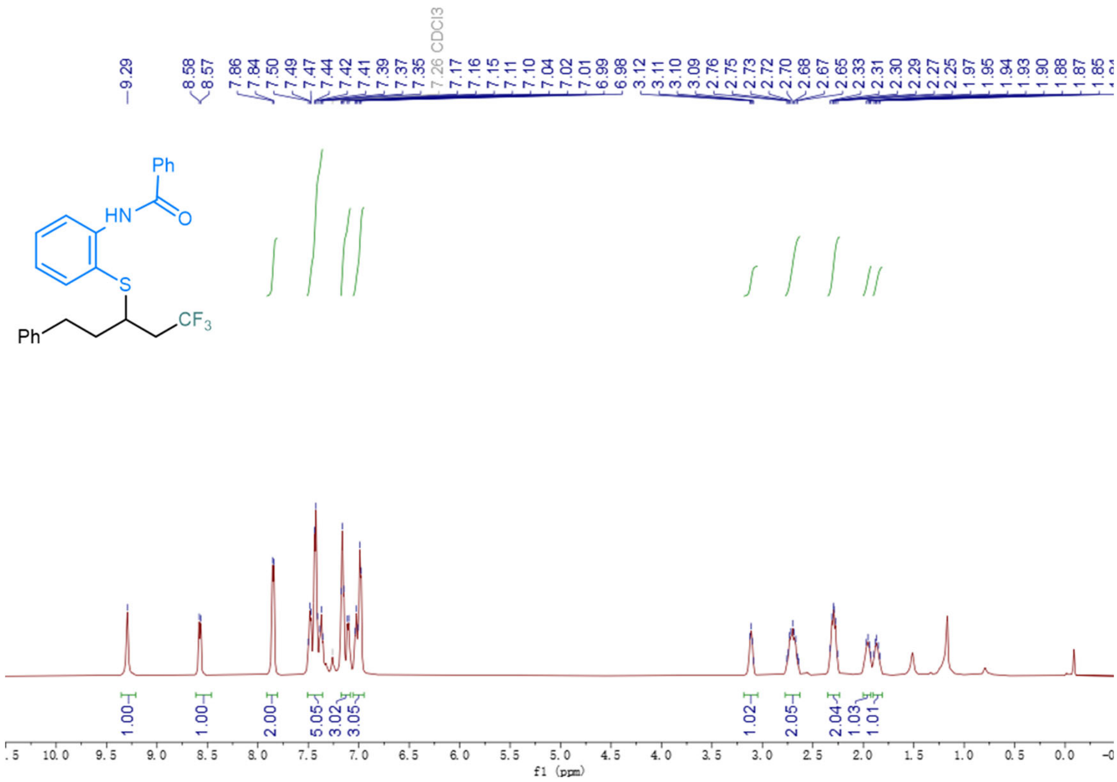

^{1}H NMR spectrum (500 MHz, Chloroform-*d*) of **51**

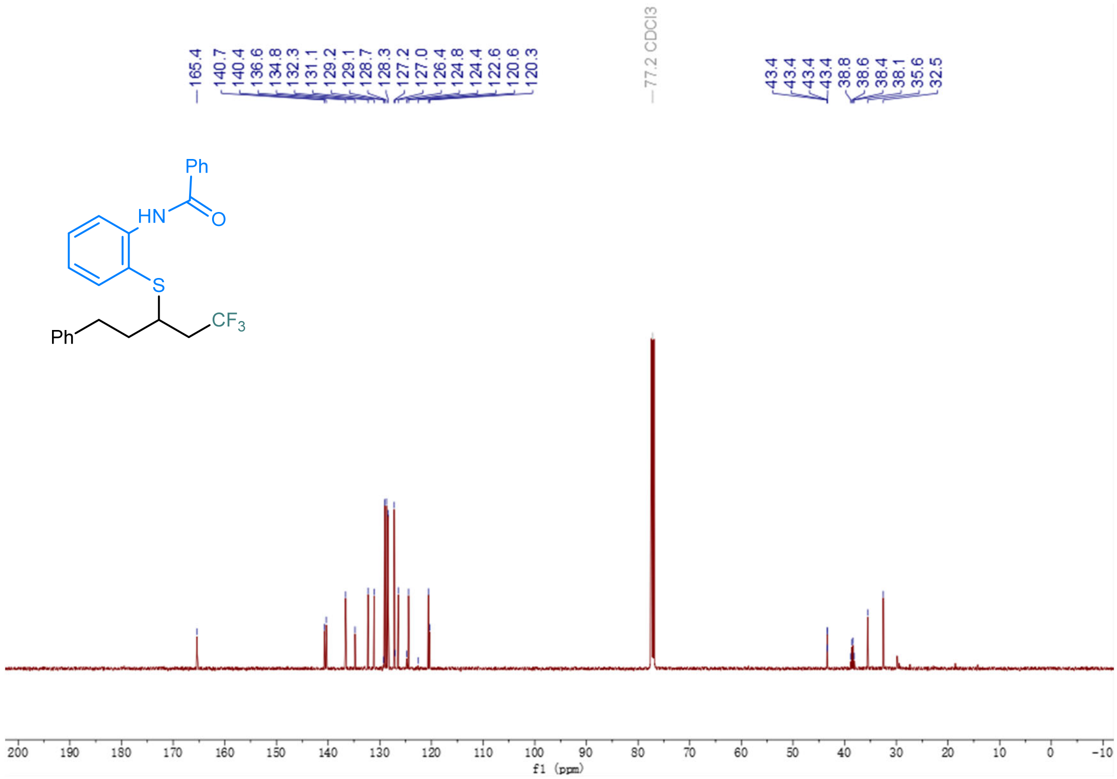

^{13}C NMR spectrum (126 MHz, Chloroform-*d*) of **51**

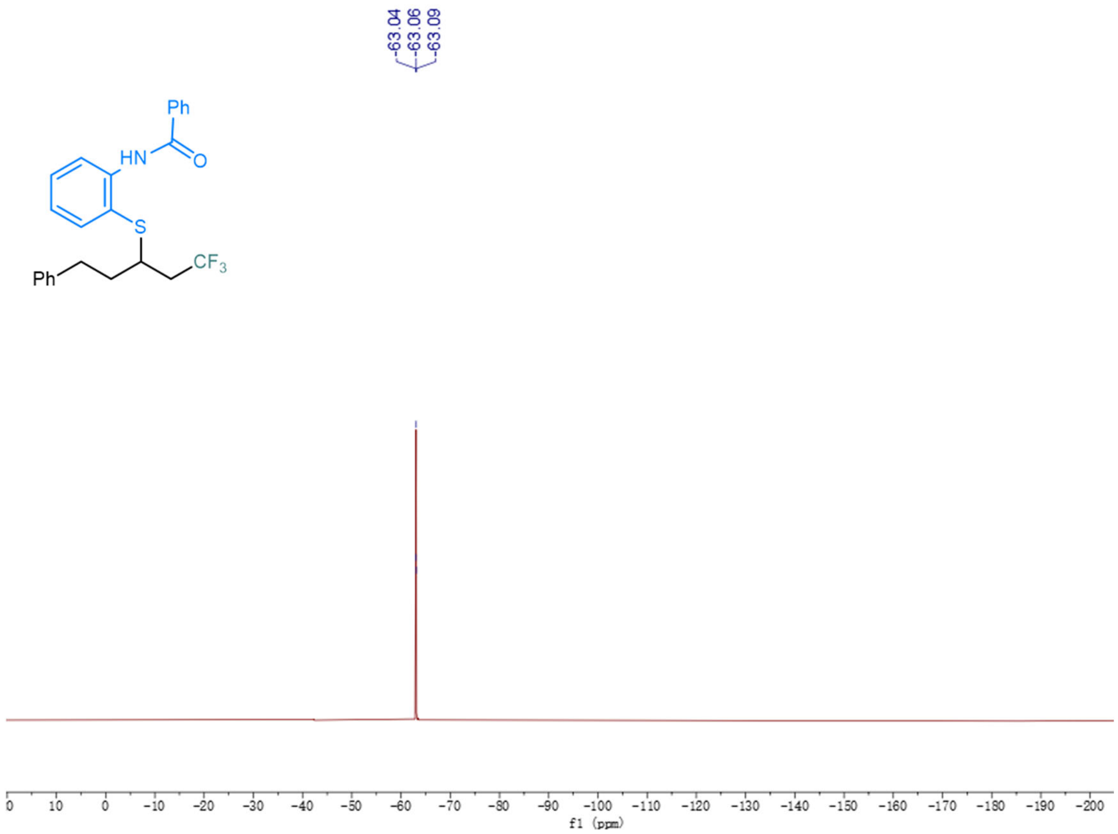

^{19}F NMR spectrum (471 MHz, Chloroform-*d*) of **51**

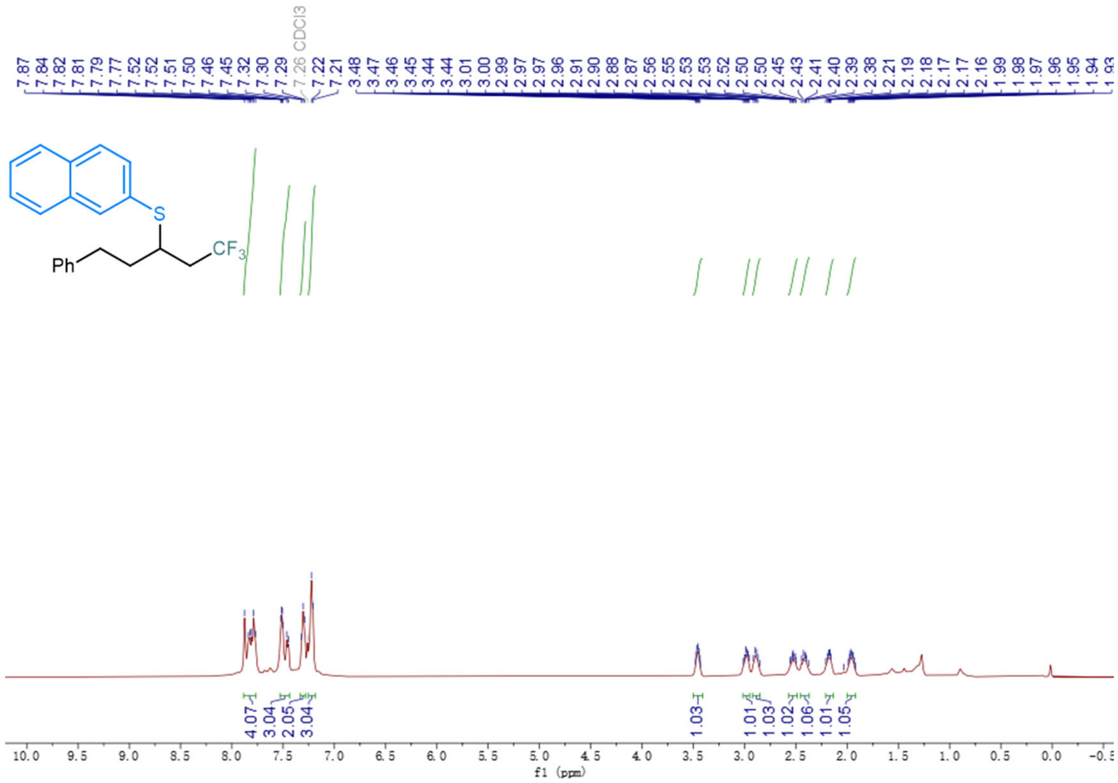

^{1}H NMR spectrum (500 MHz, Chloroform-*d*) of **52**

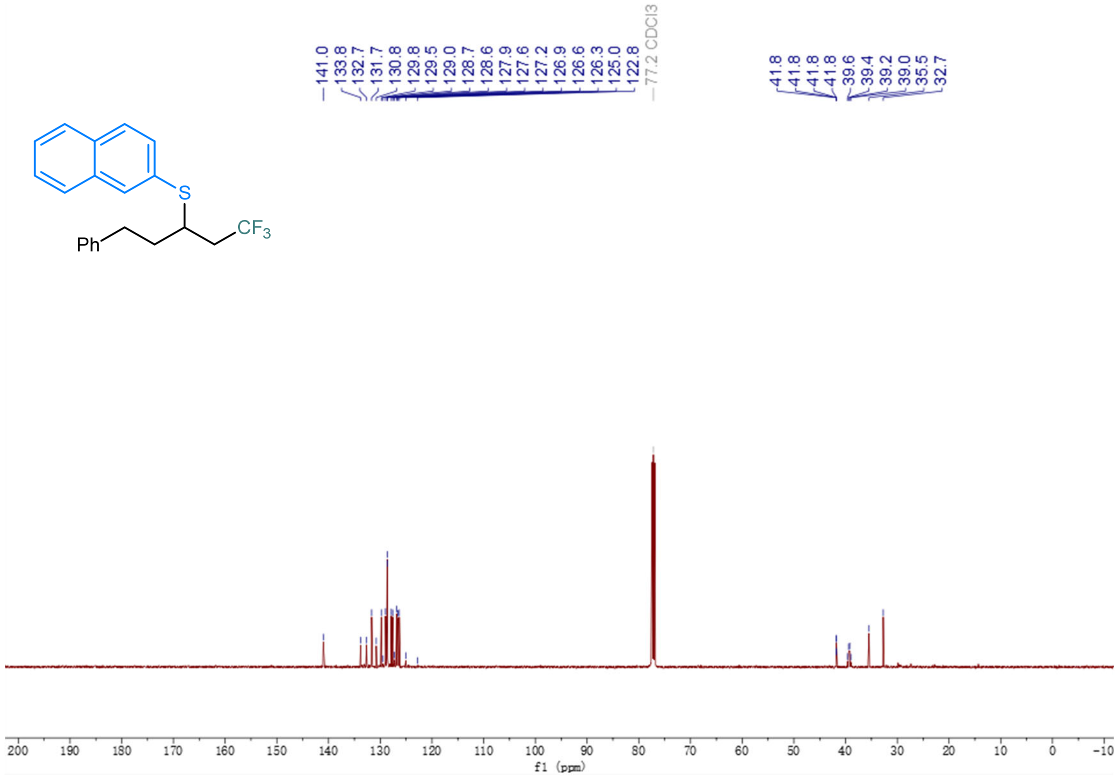

^{13}C NMR spectrum (126 MHz, Chloroform-*d*) of **52**

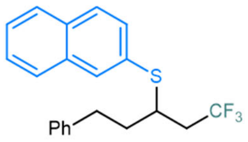

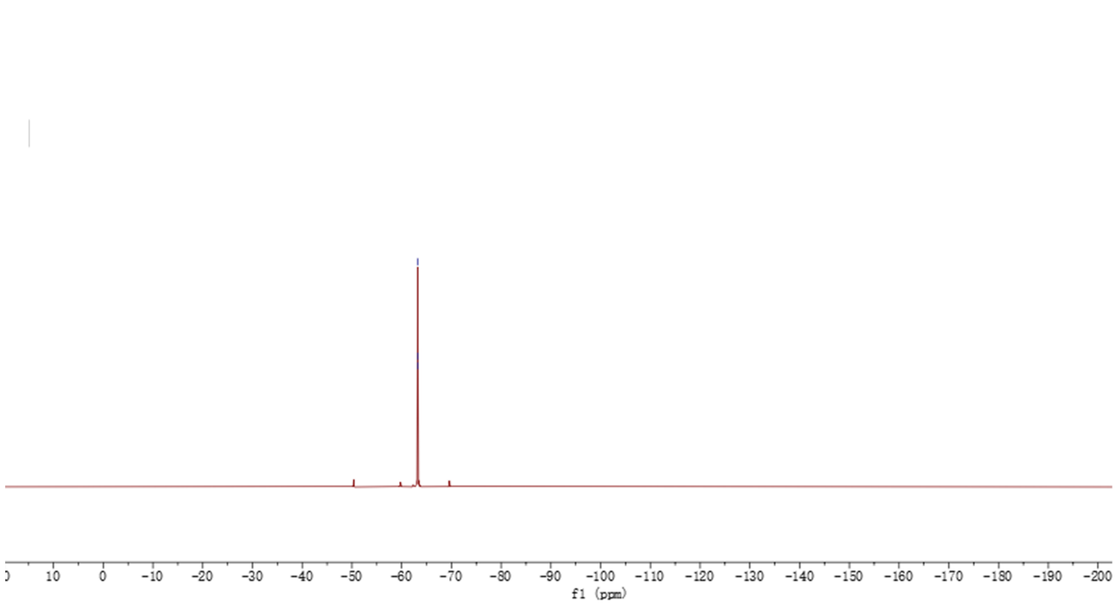

^{19}F NMR spectrum (471 MHz, Chloroform-*d*) of **52**

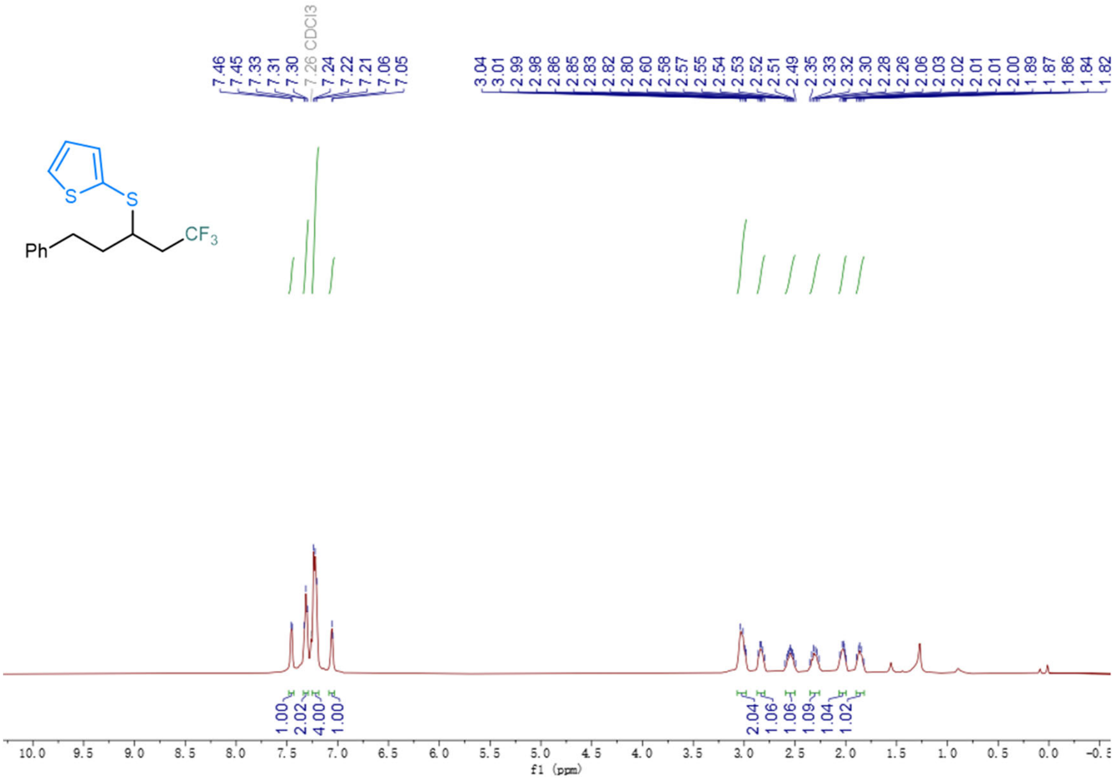

^{1}H NMR spectrum (500 MHz, Chloroform-*d*) of **53**

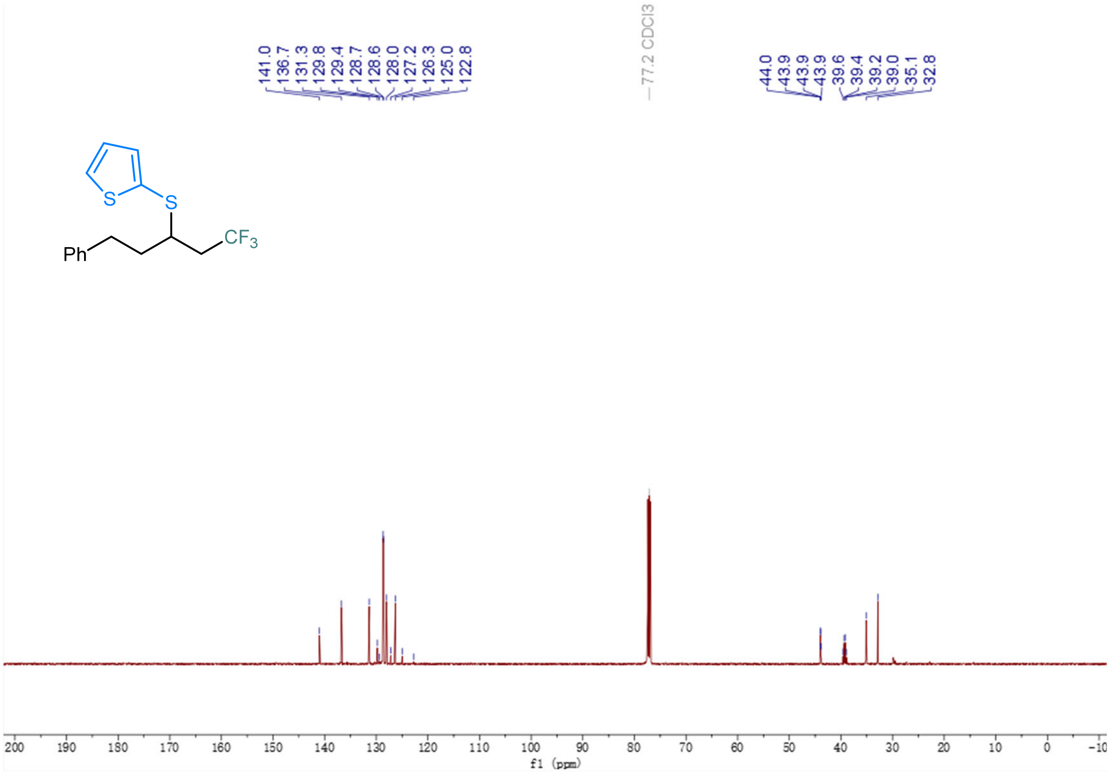

^{13}C NMR spectrum (126 MHz, Chloroform-*d*) of **53**

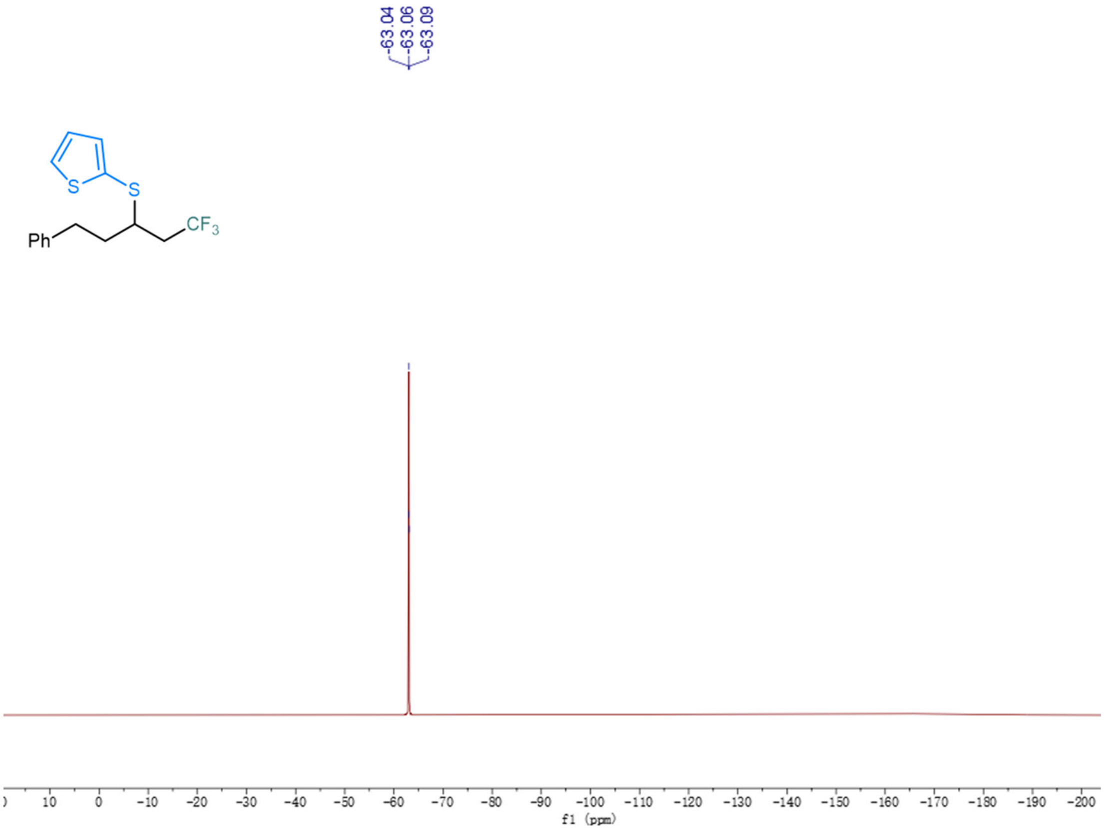

^{19}F NMR spectrum (471 MHz, Chloroform-*d*) of **53**

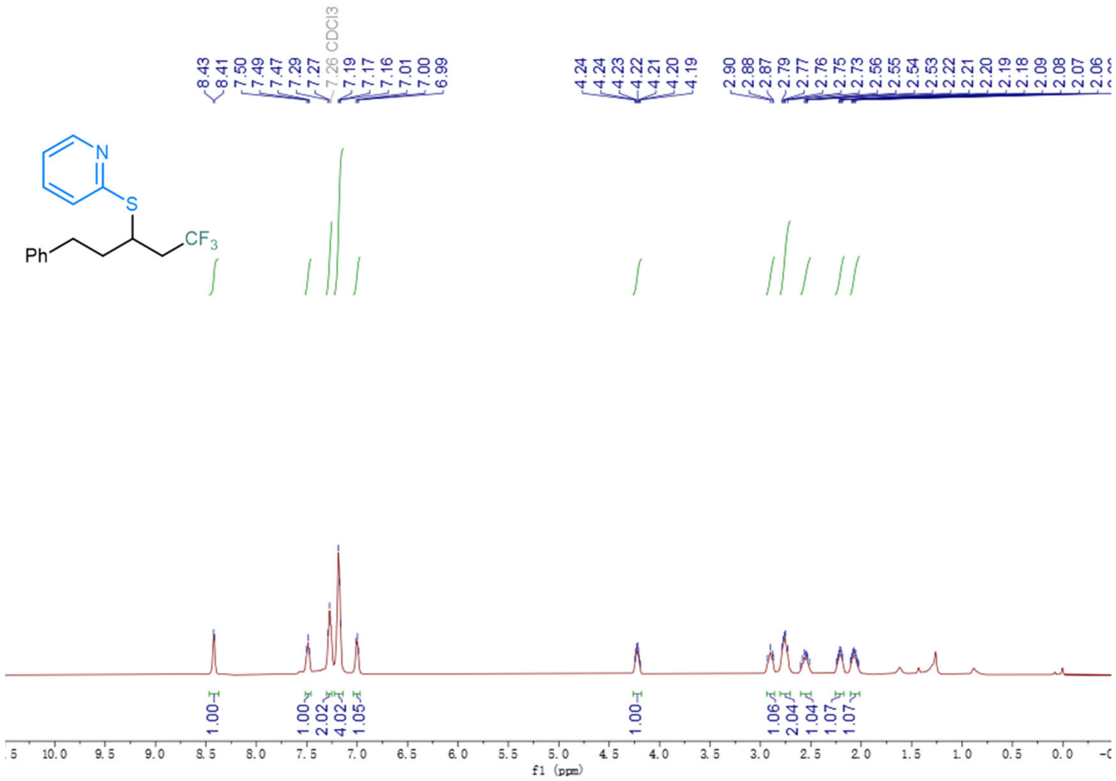

^{1}H NMR spectrum (500 MHz, Chloroform-*d*) of **54**

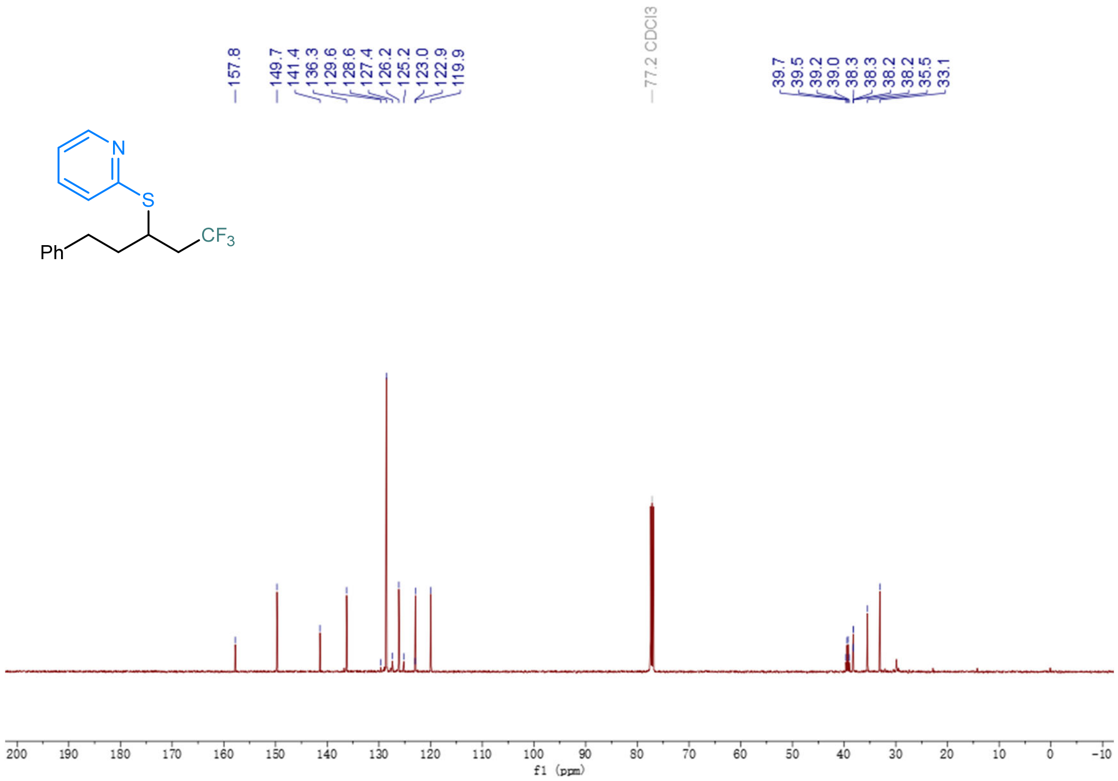

^{13}C NMR spectrum (126 MHz, Chloroform-*d*) of **54**

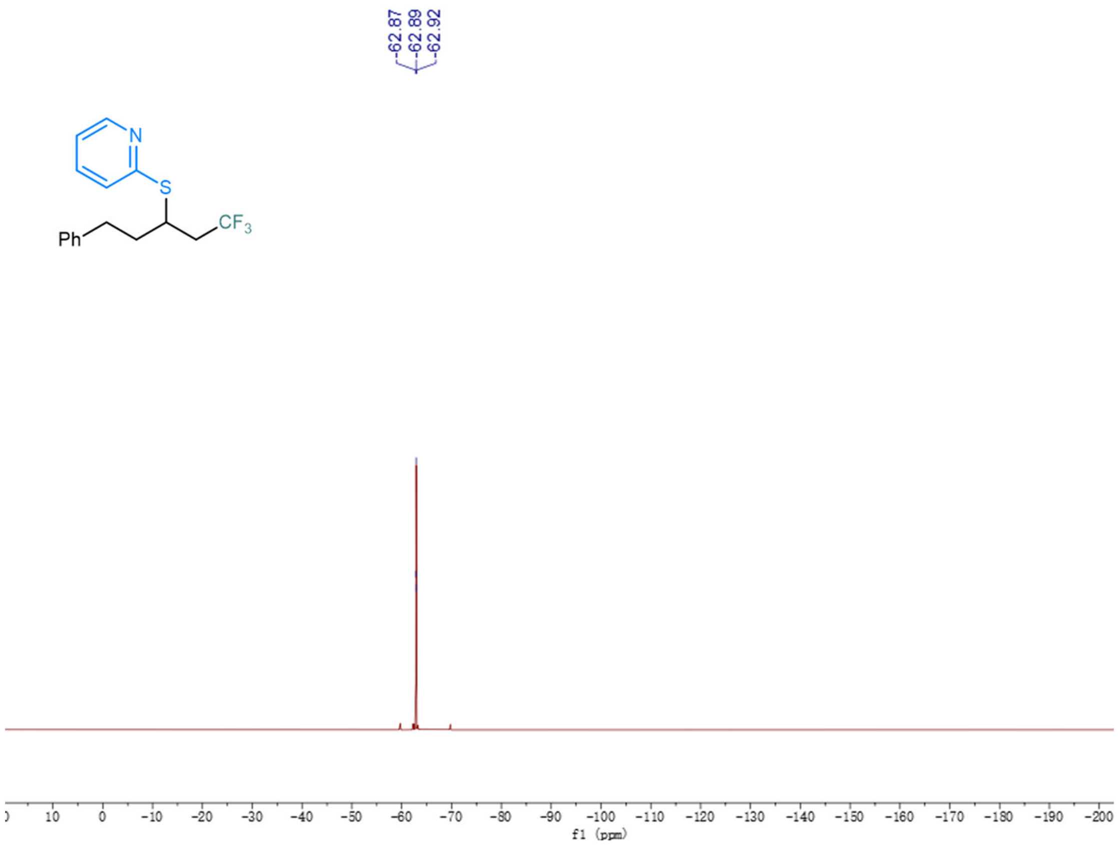

^{19}F NMR spectrum (471 MHz, Chloroform-*d*) of **54**

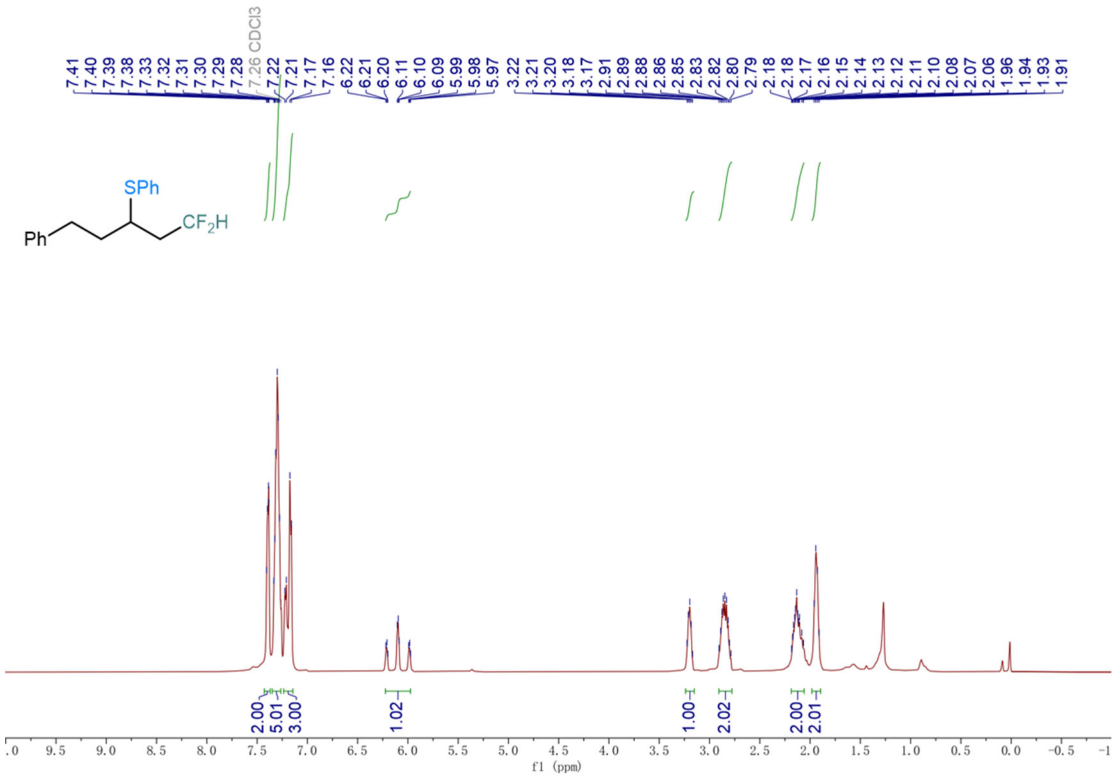

^{1}H NMR spectrum (500 MHz, Chloroform-*d*) of **55**

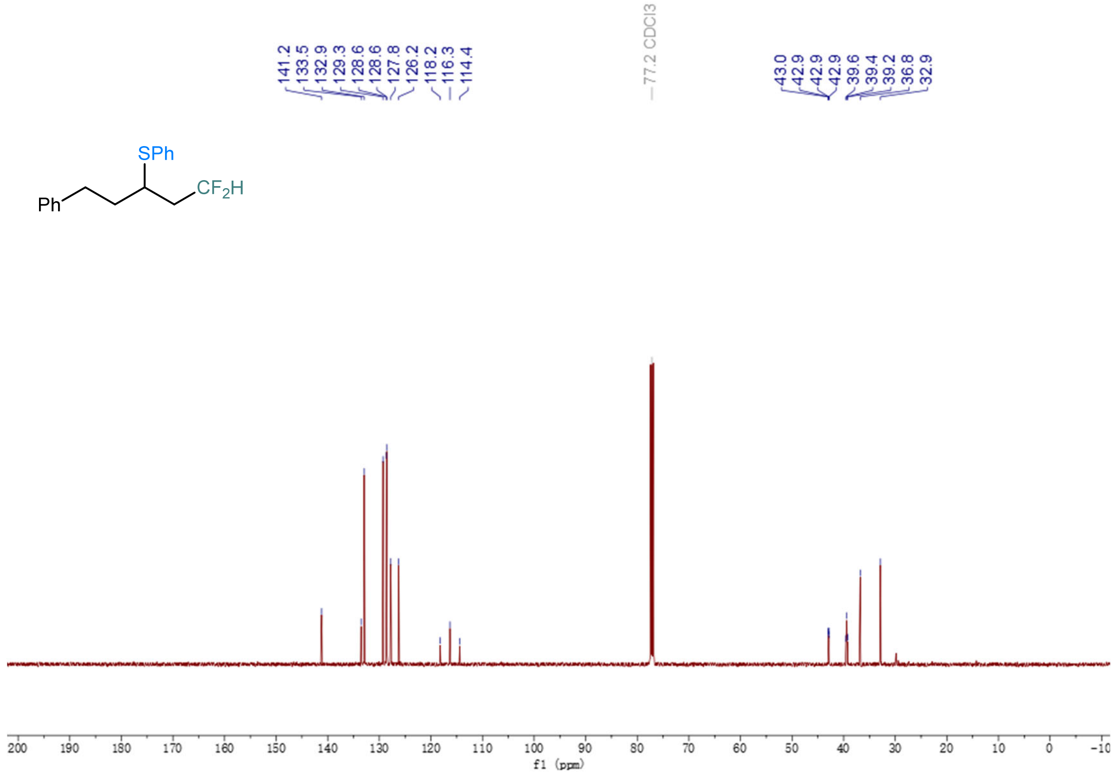

^{13}C NMR spectrum (126 MHz, Chloroform-*d*) of **55**

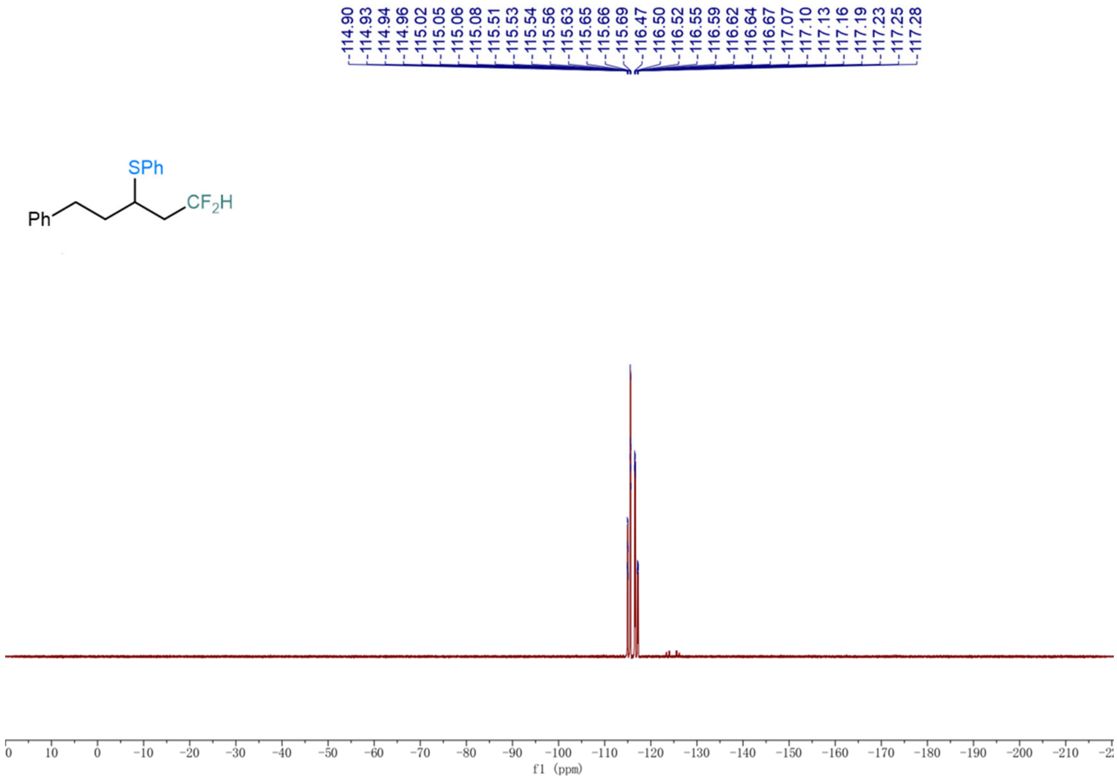

^{19}F NMR spectrum (471 MHz, Chloroform-*d*) of **55**

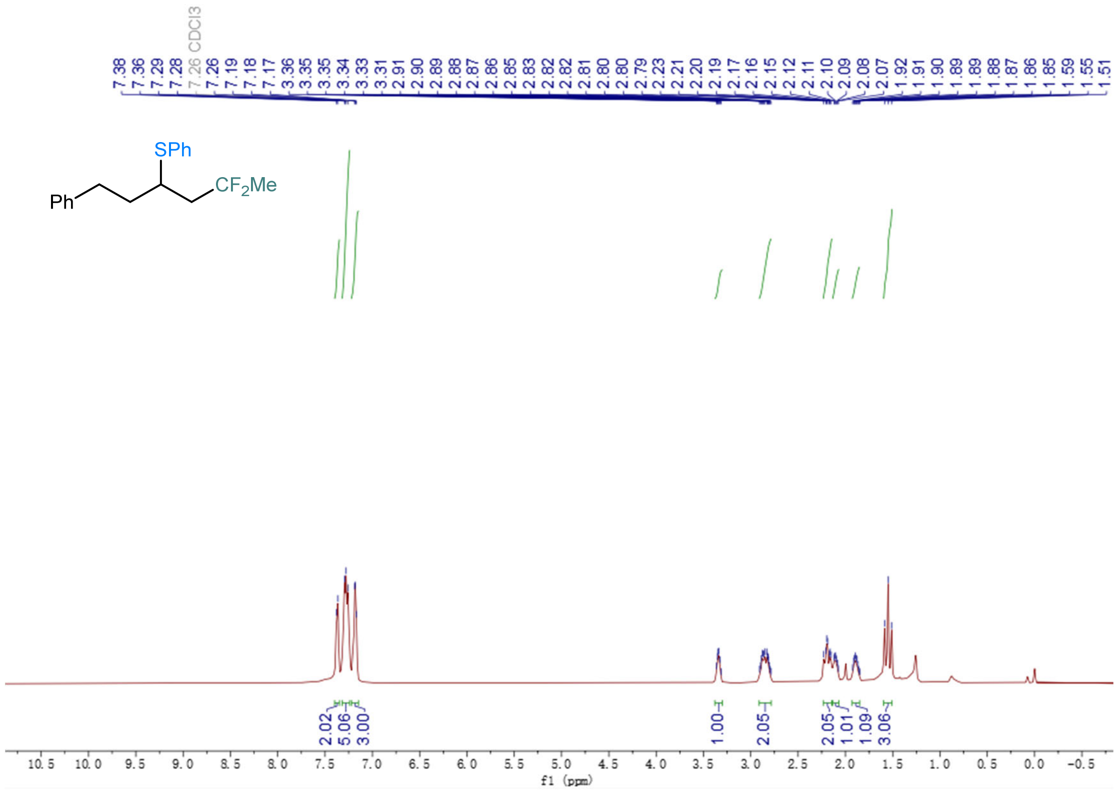

^{1}H NMR spectrum (500 MHz, Chloroform-*d*) of **56**

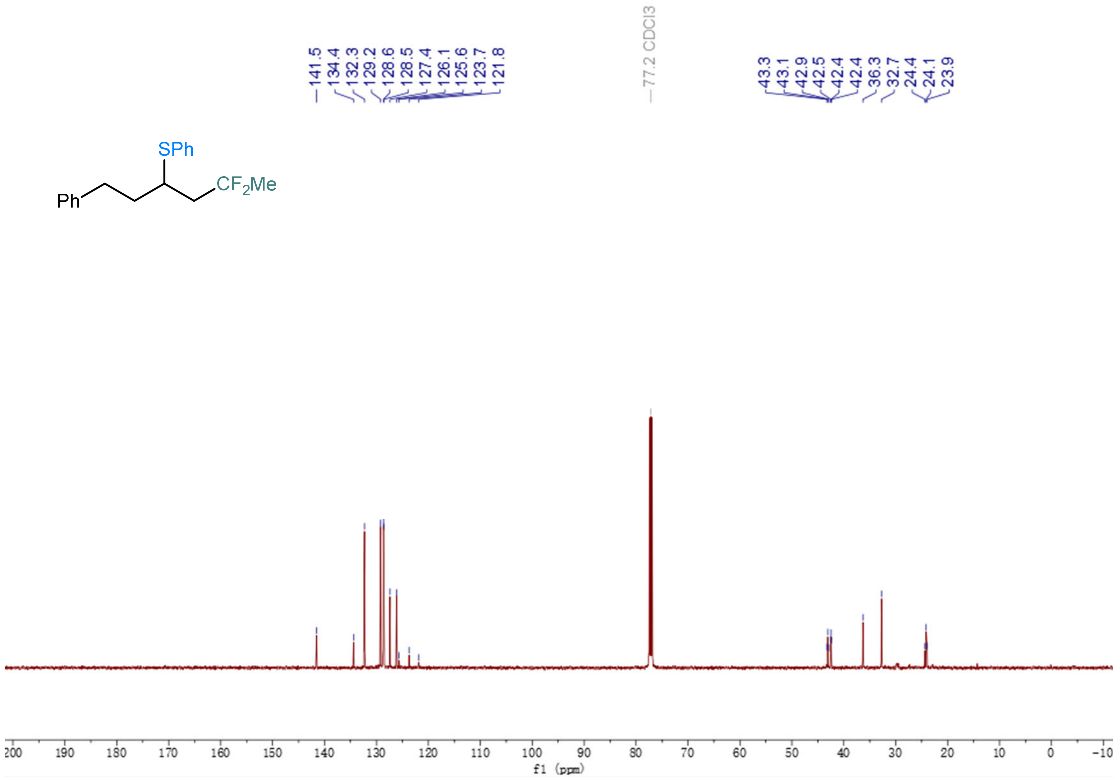

^{13}C NMR spectrum (126 MHz, Chloroform-*d*) of **56**

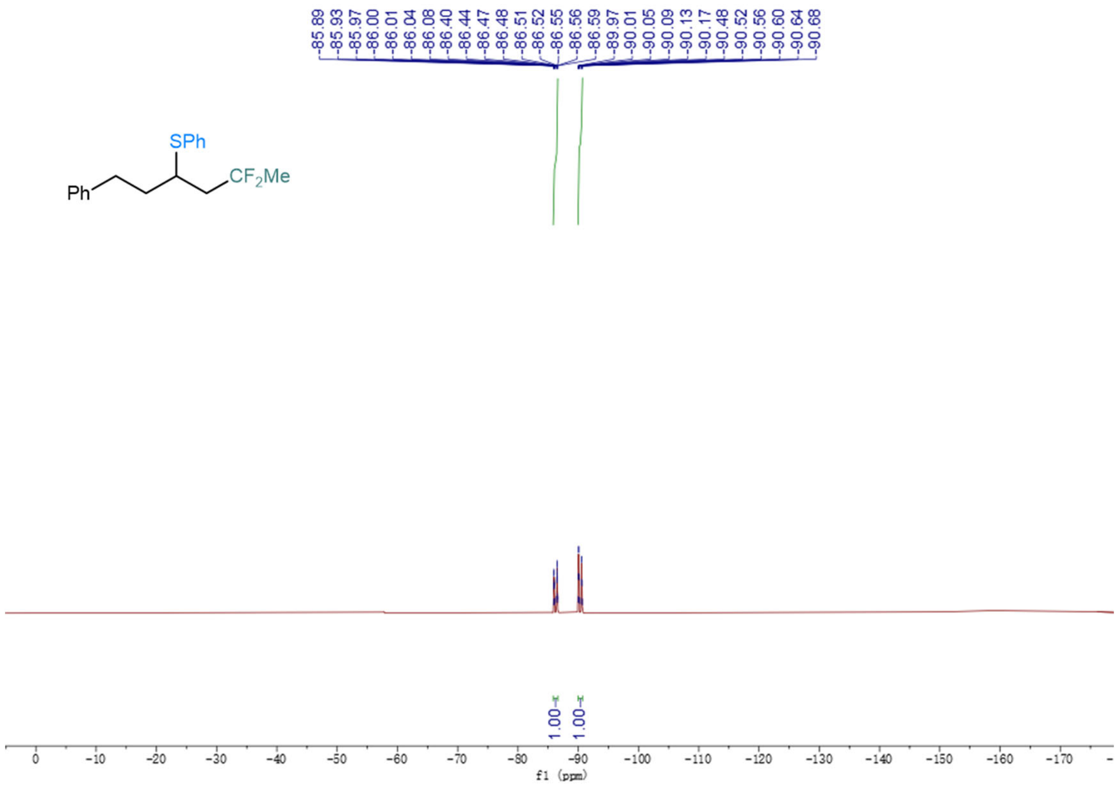

^{19}F NMR spectrum (471 MHz, Chloroform-*d*) of **56**

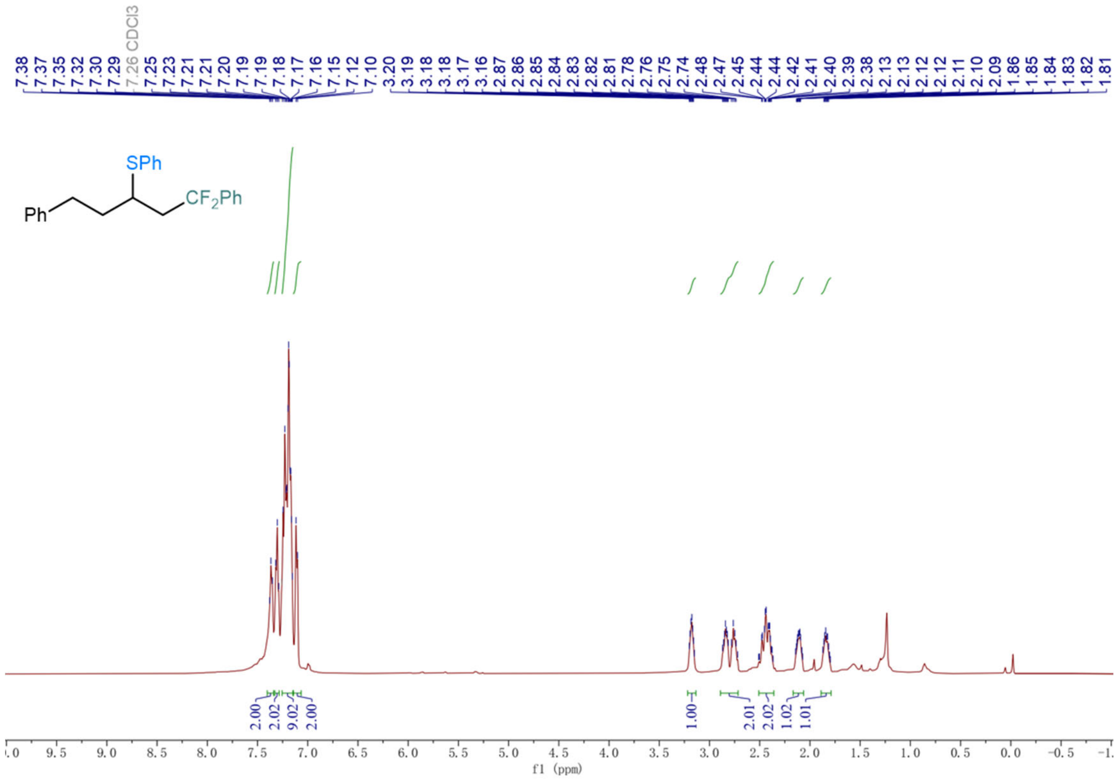

^{1}H NMR spectrum (500 MHz, Chloroform-*d*) of **57**

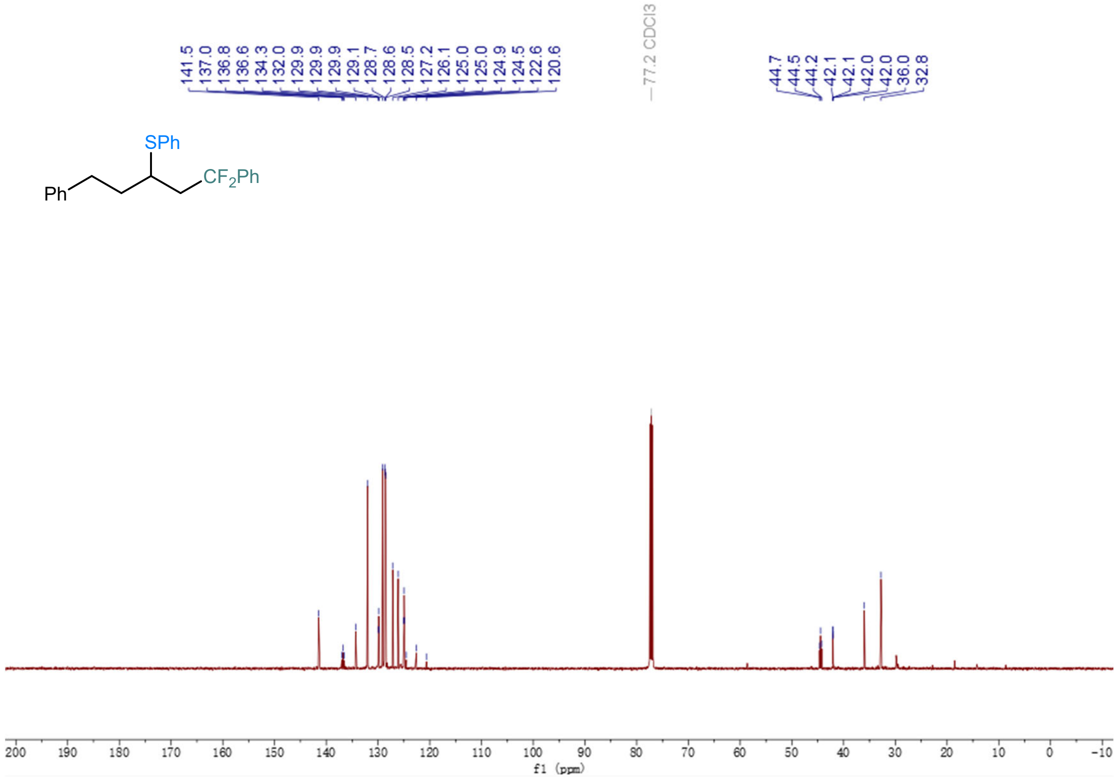

^{13}C NMR spectrum (126 MHz, Chloroform-*d*) of **57**

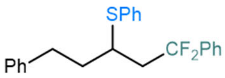

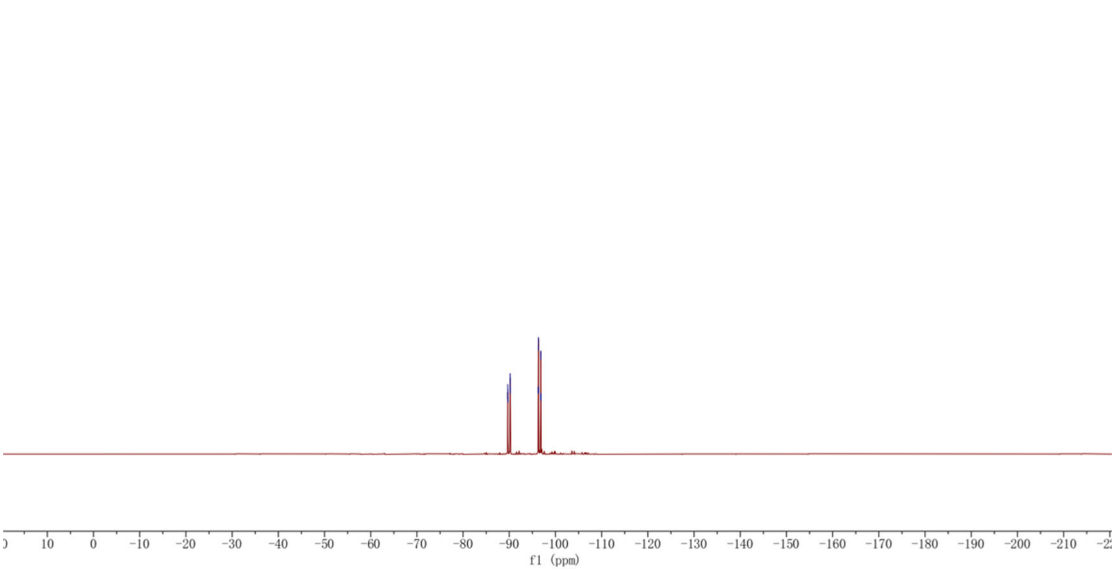

^{19}F NMR spectrum (471 MHz, Chloroform-*d*) of **57**

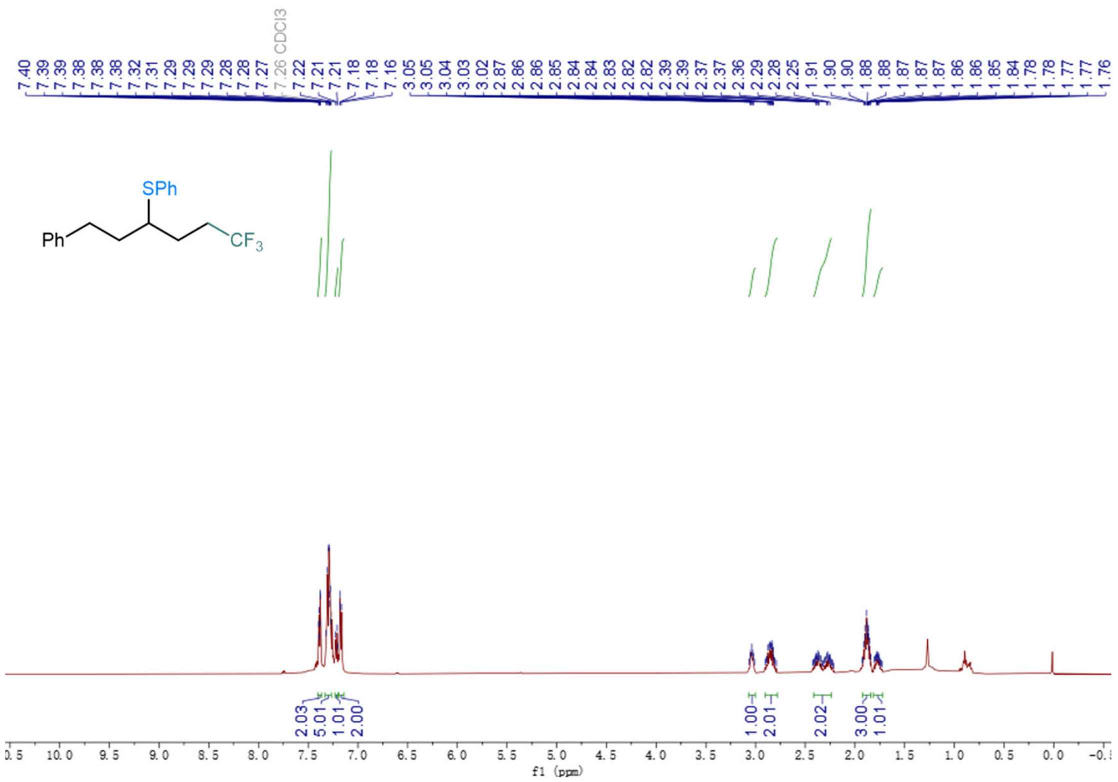

^{1}H NMR spectrum (500 MHz, Chloroform-*d*) of **58**

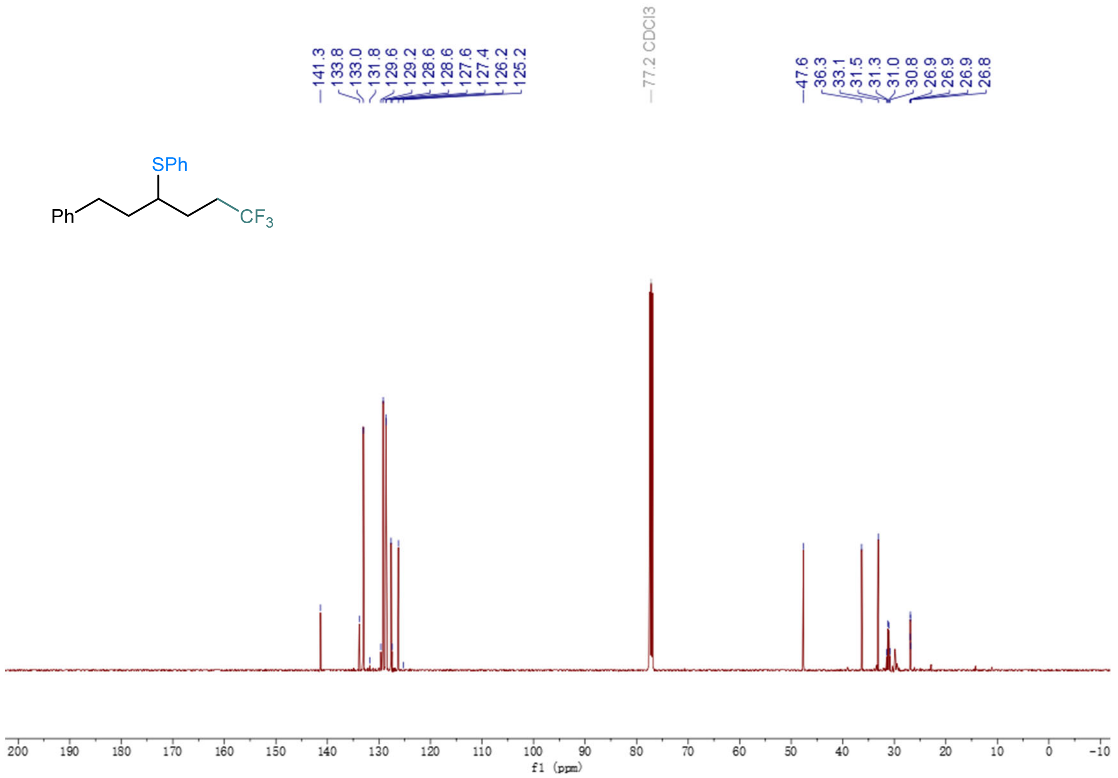

^{13}C NMR spectrum (126 MHz, Chloroform-*d*) of **58**

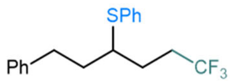

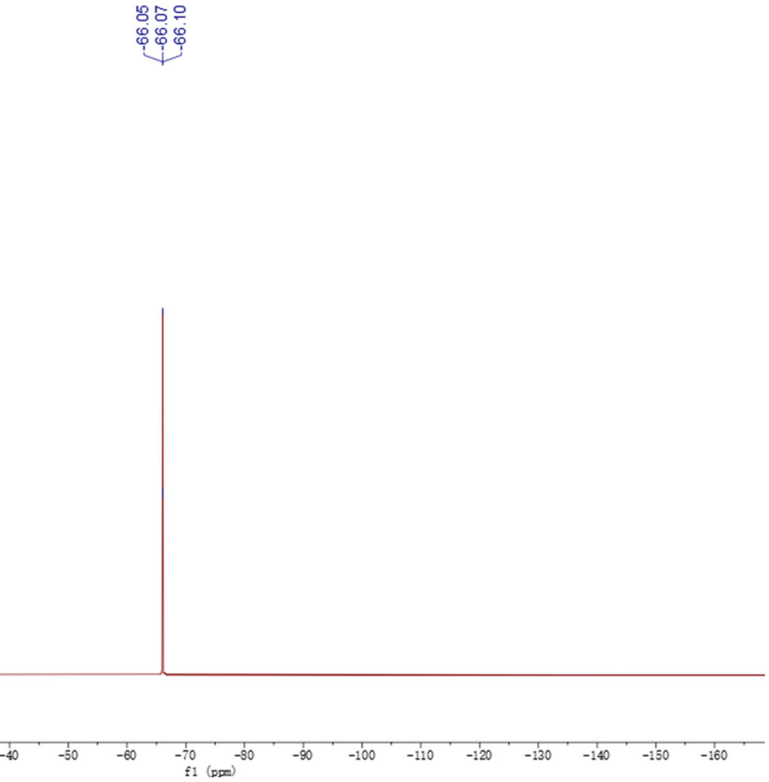

^{19}F NMR spectrum (471 MHz, Chloroform-*d*) of **58**

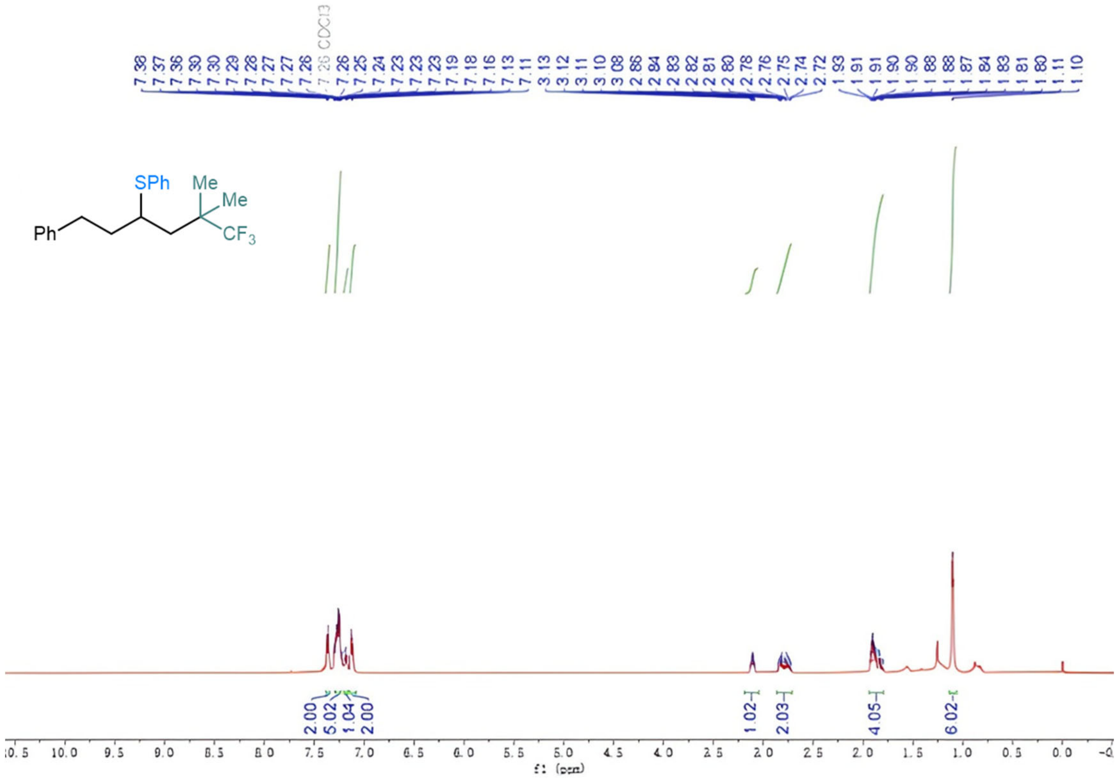

^{1}H NMR spectrum (500 MHz, Chloroform-*d*) of **59**

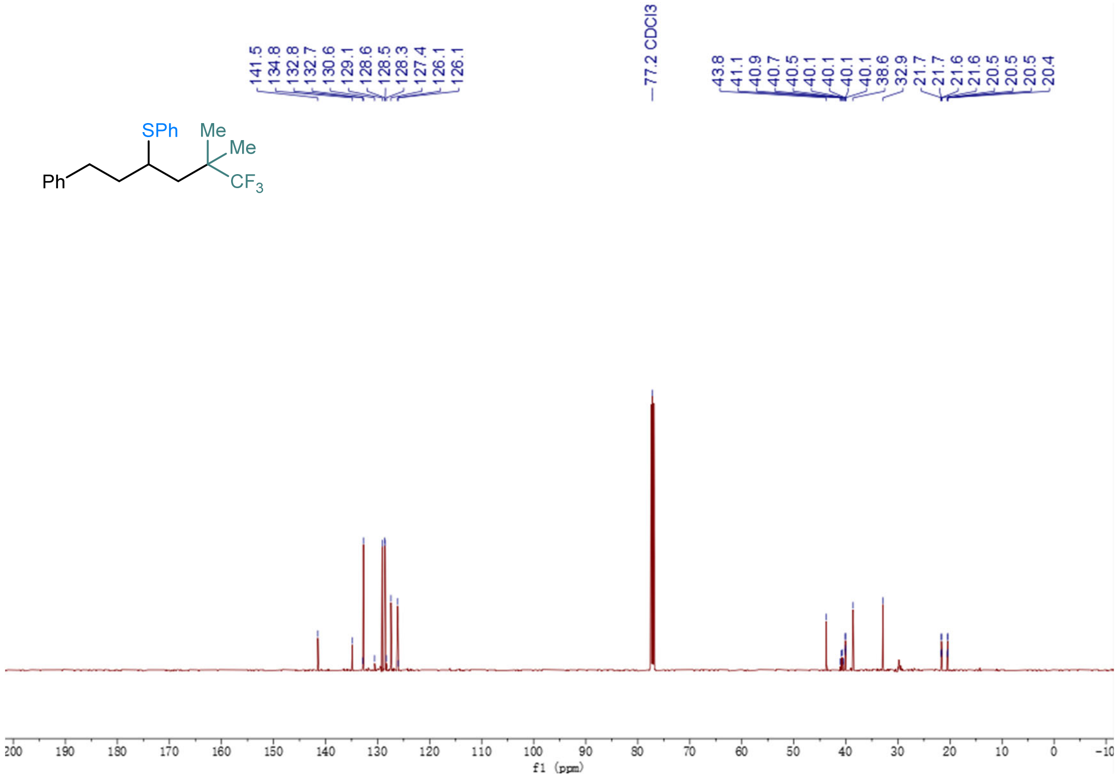

^{13}C NMR spectrum (126 MHz, Chloroform-*d*) of **59**

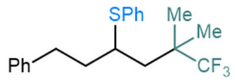

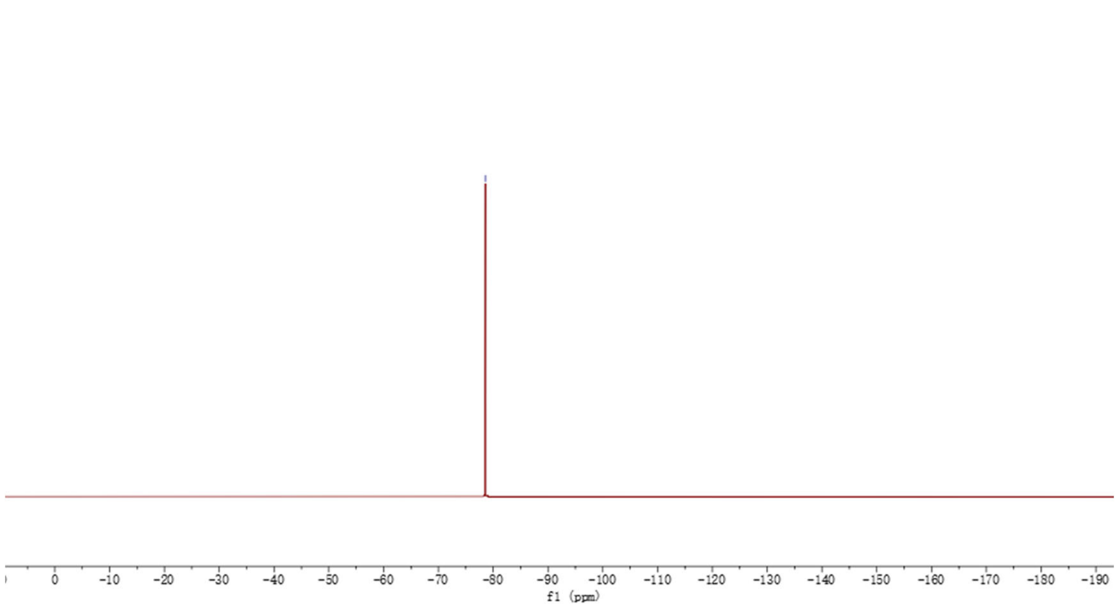

^{19}F NMR spectrum (471 MHz, Chloroform-*d*) of **59**

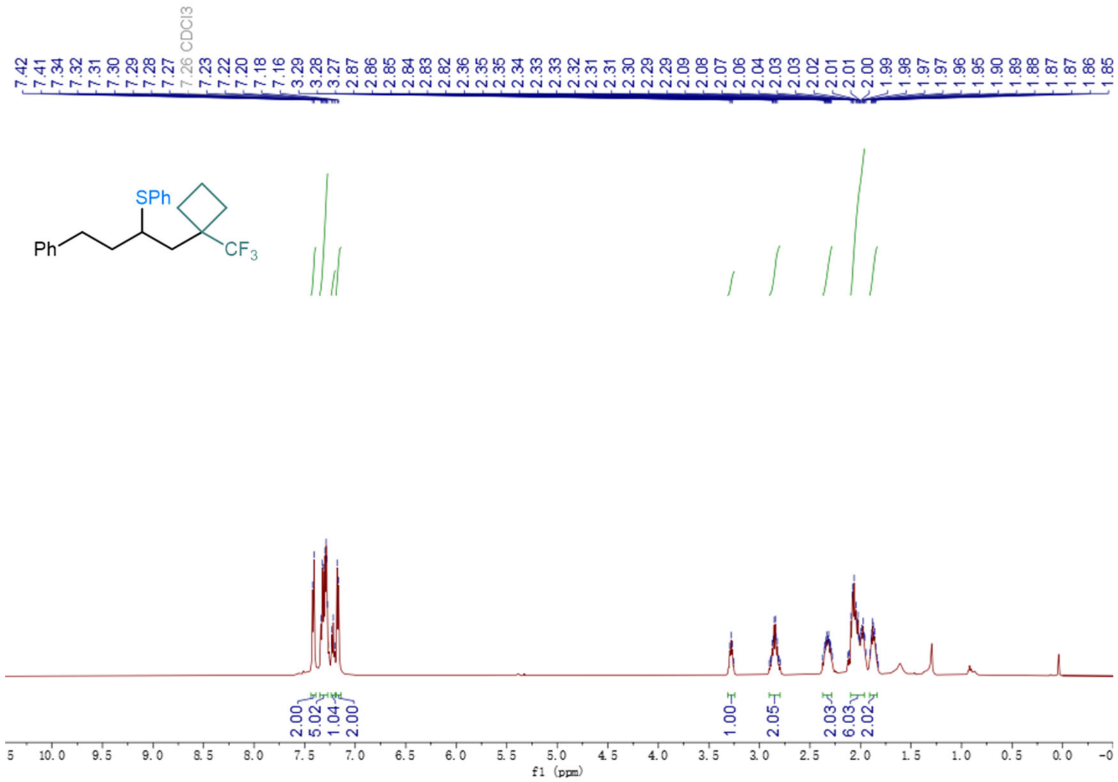

^{1}H NMR spectrum (500 MHz, Chloroform-*d*) of **60**

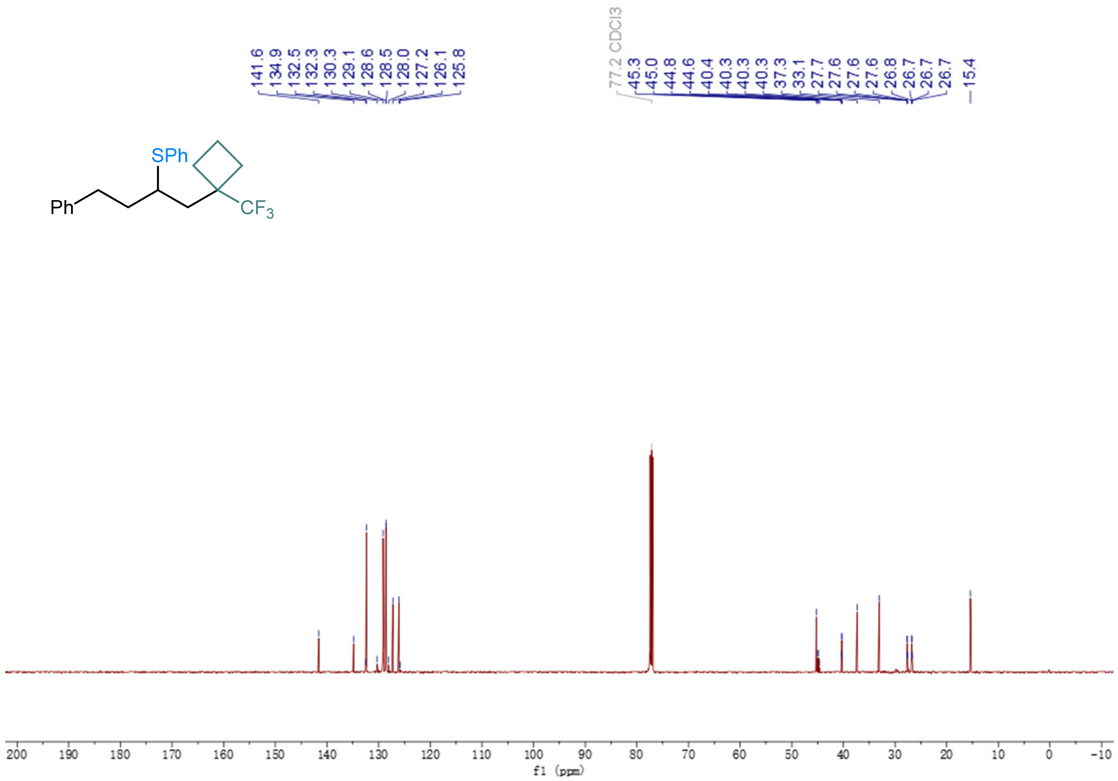

^{13}C NMR spectrum (126 MHz, Chloroform-*d*) of **60**

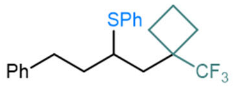

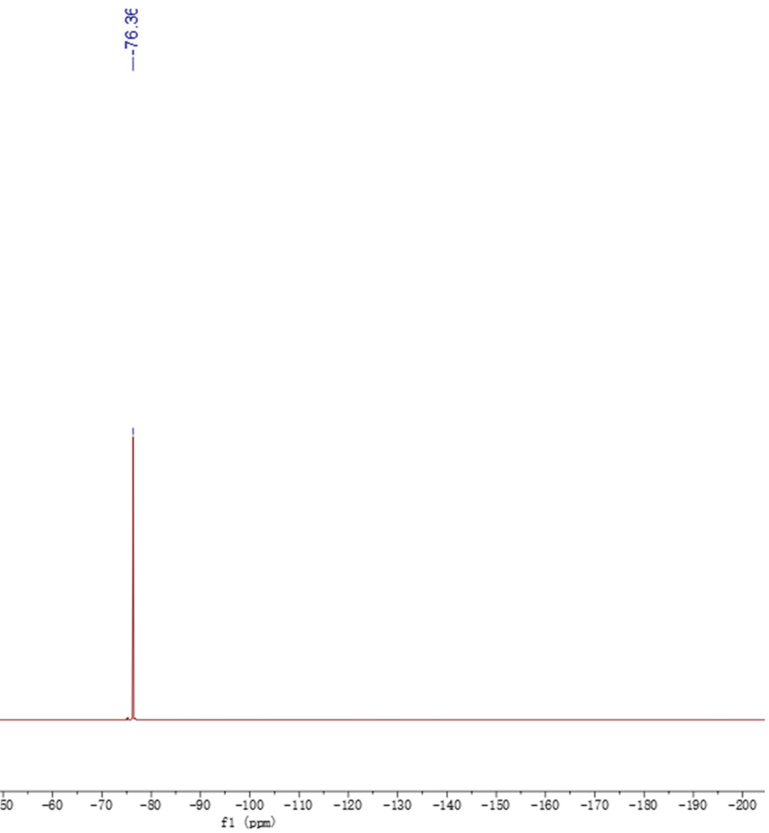

^{19}F NMR spectrum (471 MHz, Chloroform-*d*) of **60**

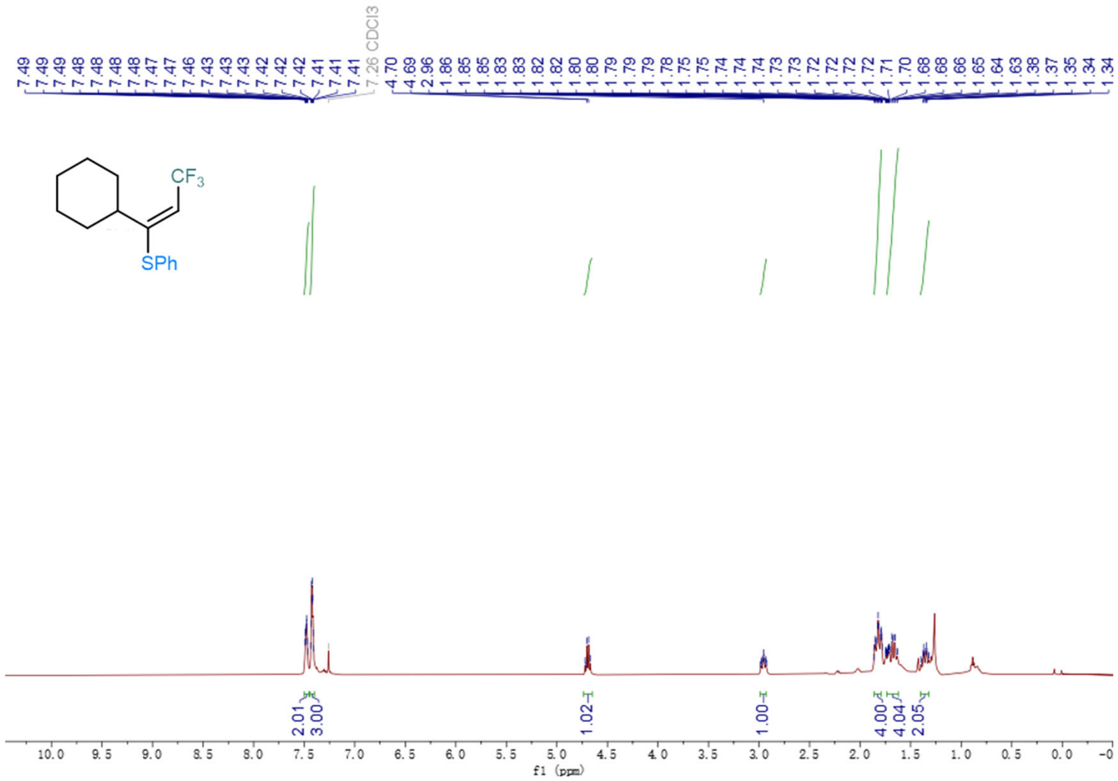

^{1}H NMR spectrum (500 MHz, Chloroform-*d*) of **61**

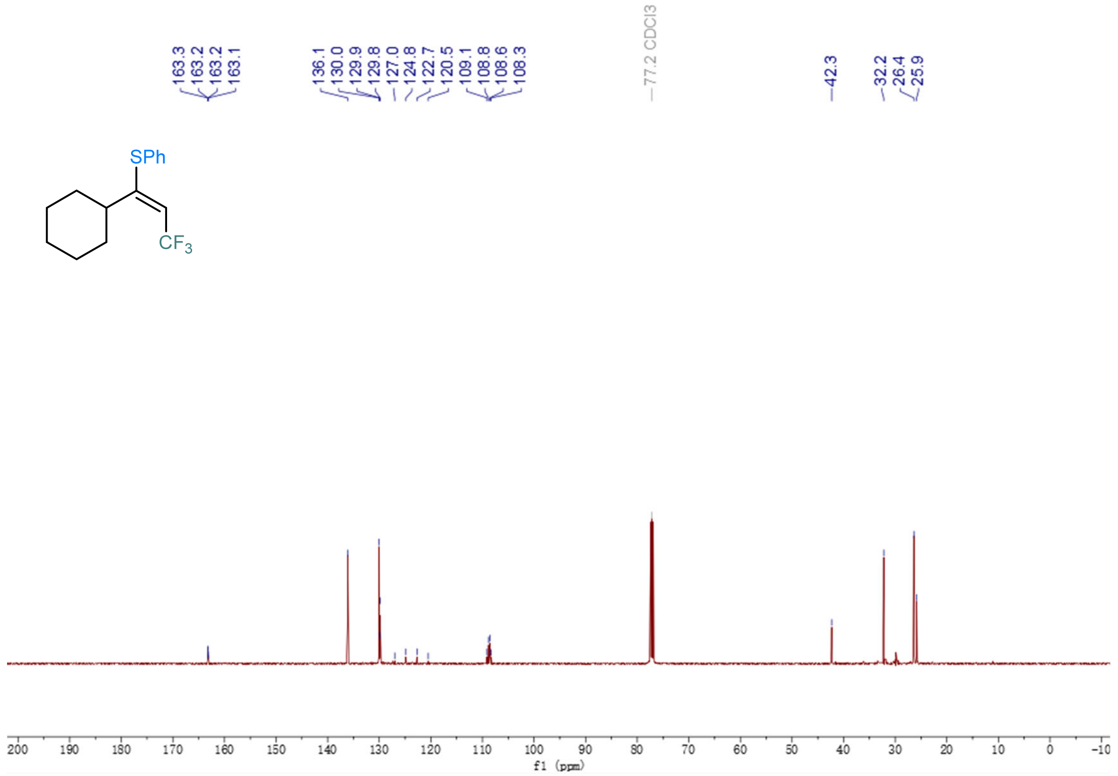

^{13}C NMR spectrum (126 MHz, Chloroform-*d*) of **61**

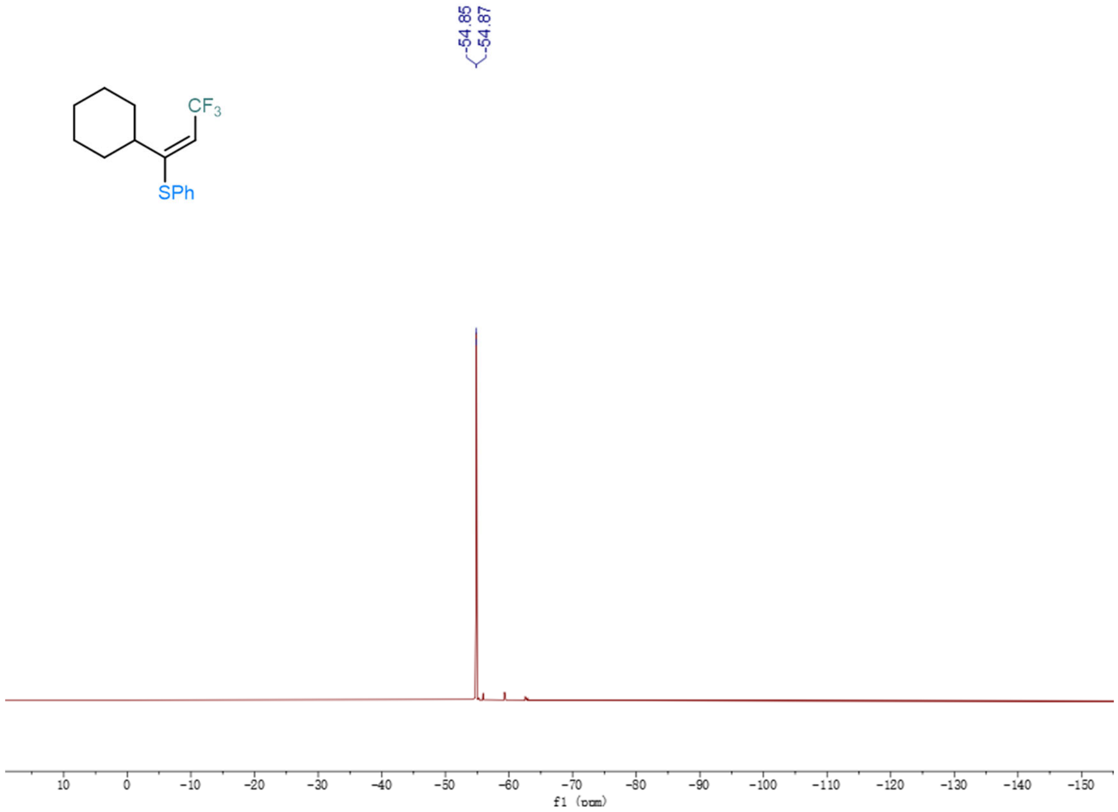

^{19}F NMR spectrum (471 MHz, Chloroform-*d*) of **61**

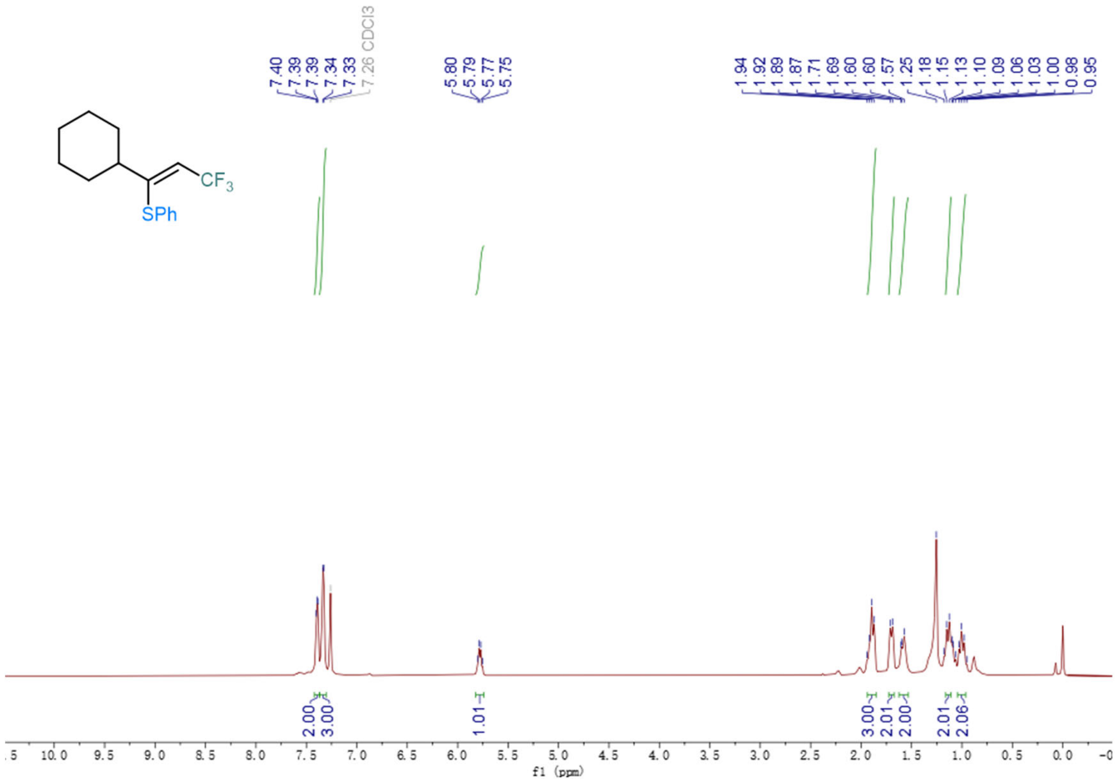

^{1}H NMR spectrum (500 MHz, Chloroform-*d*) of **61'**

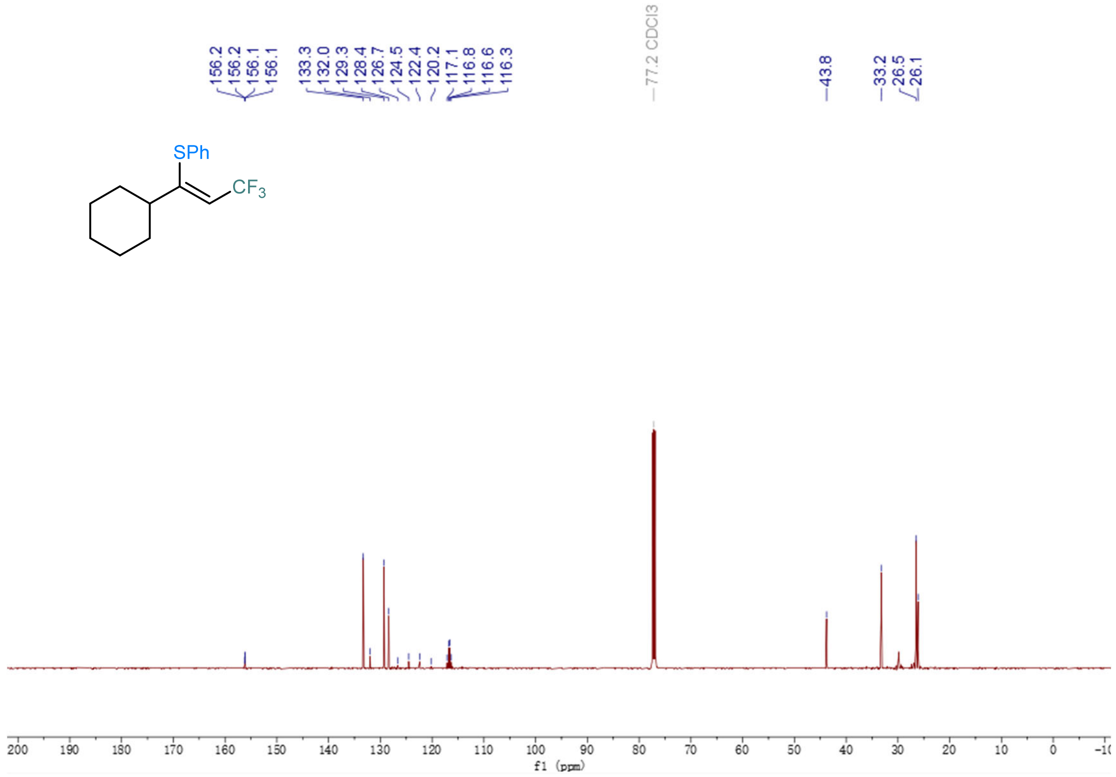

^{13}C NMR spectrum (126 MHz, Chloroform-*d*) of **61'**

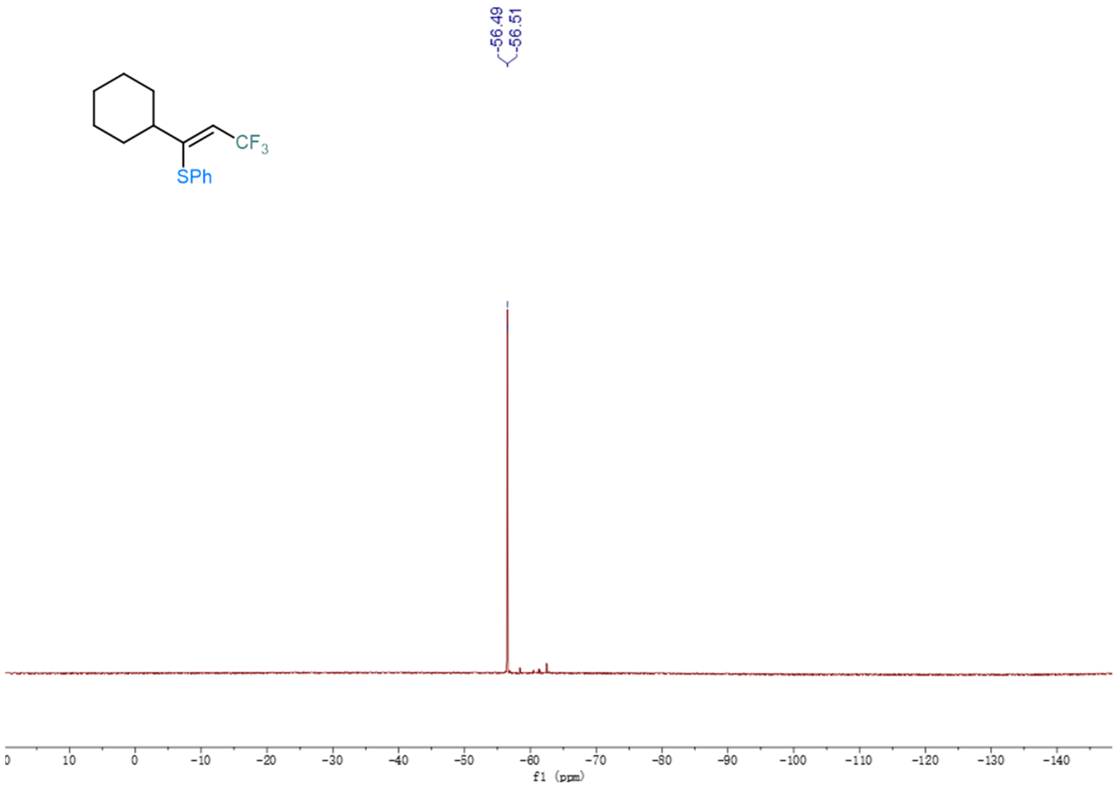

^{19}F NMR spectrum (471 MHz, Chloroform-*d*) of **61'**

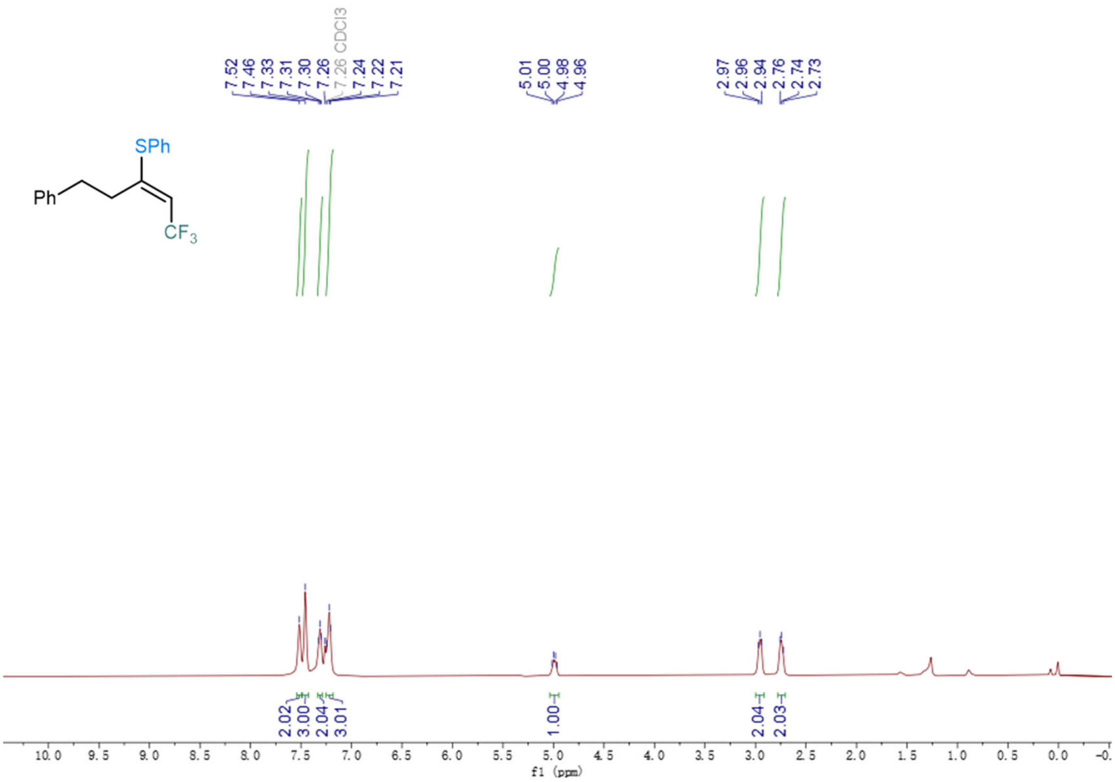

^{1}H NMR spectrum (500 MHz, Chloroform-*d*) of **62**

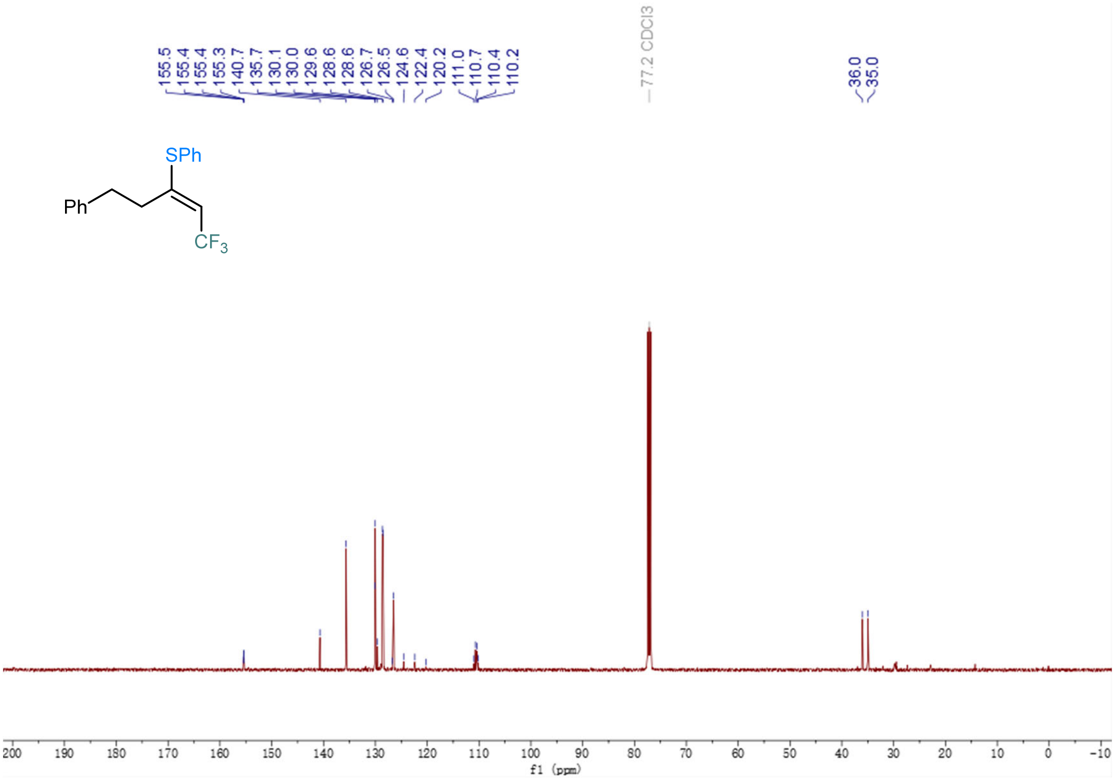

^{13}C NMR spectrum (126 MHz, Chloroform-*d*) of **62**

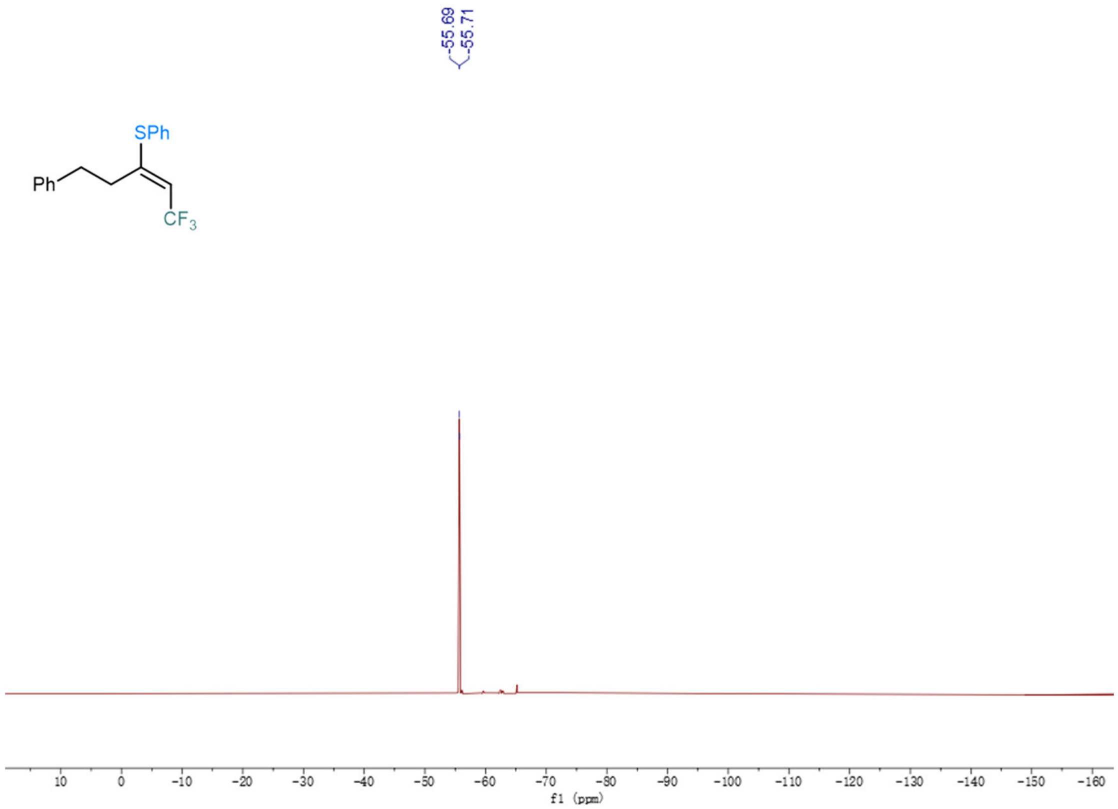

^{19}F NMR spectrum (471 MHz, Chloroform-*d*) of **62**

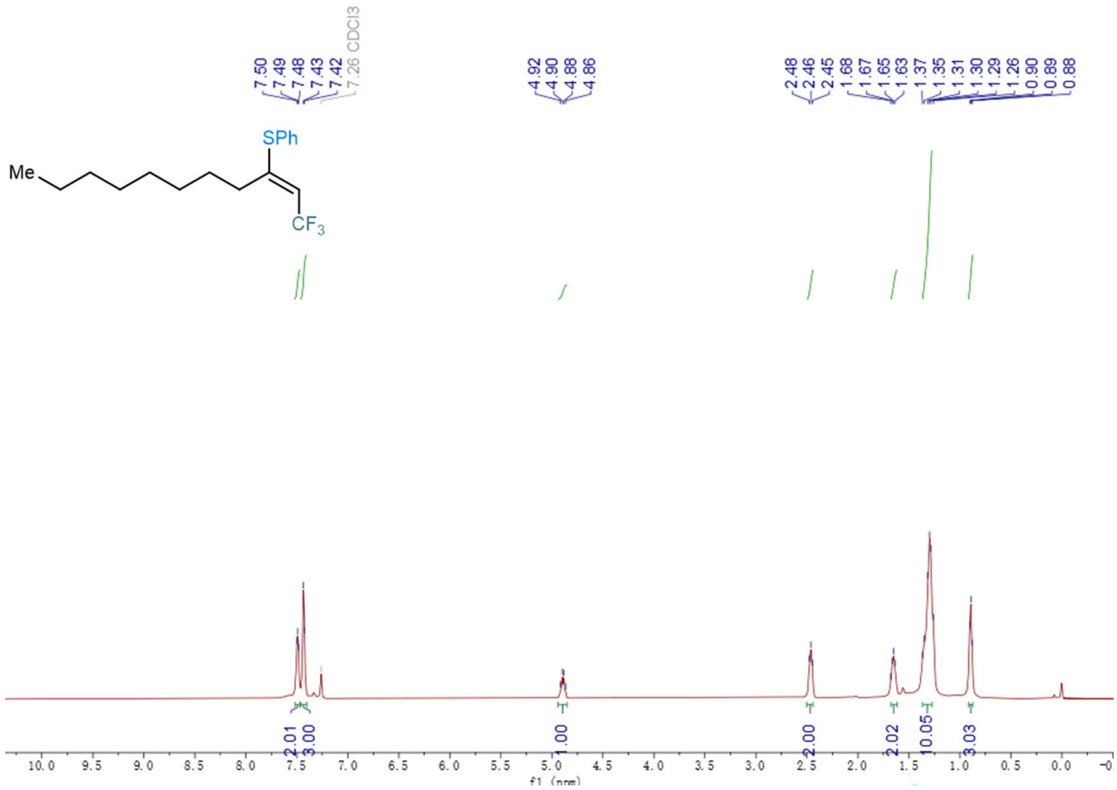

^{1}H NMR spectrum (500 MHz, Chloroform-*d*) of **63**

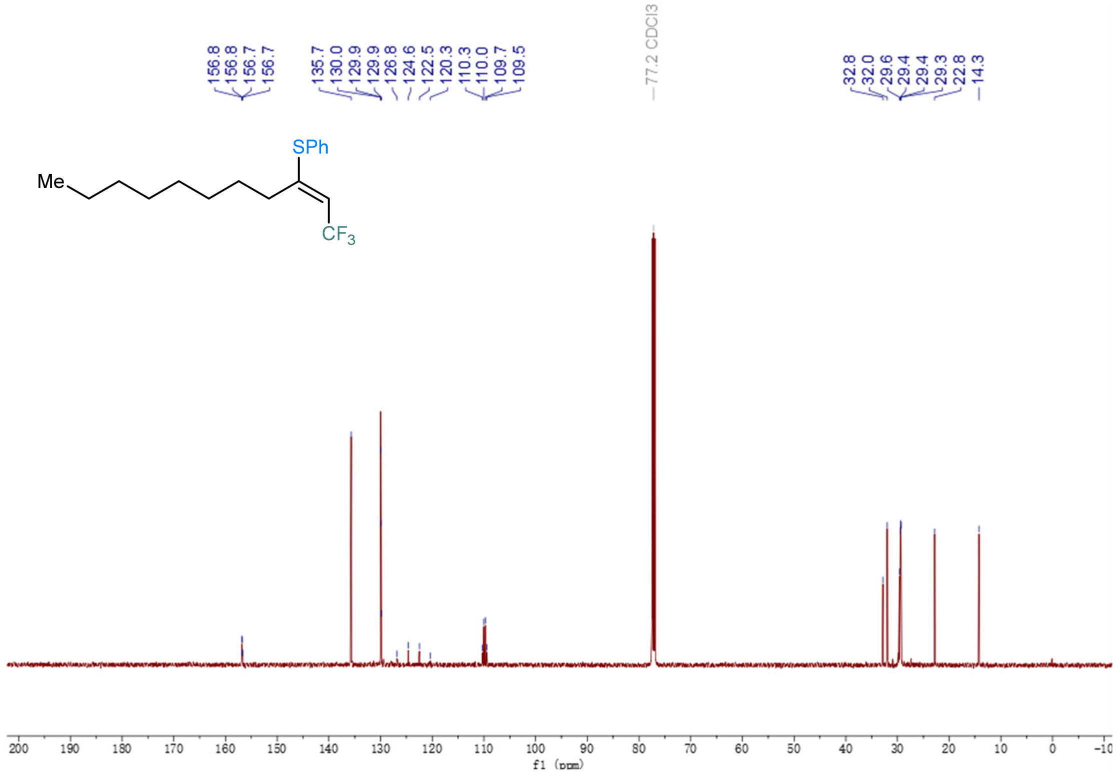

^{13}C NMR spectrum (126 MHz, Chloroform-*d*) of **63**

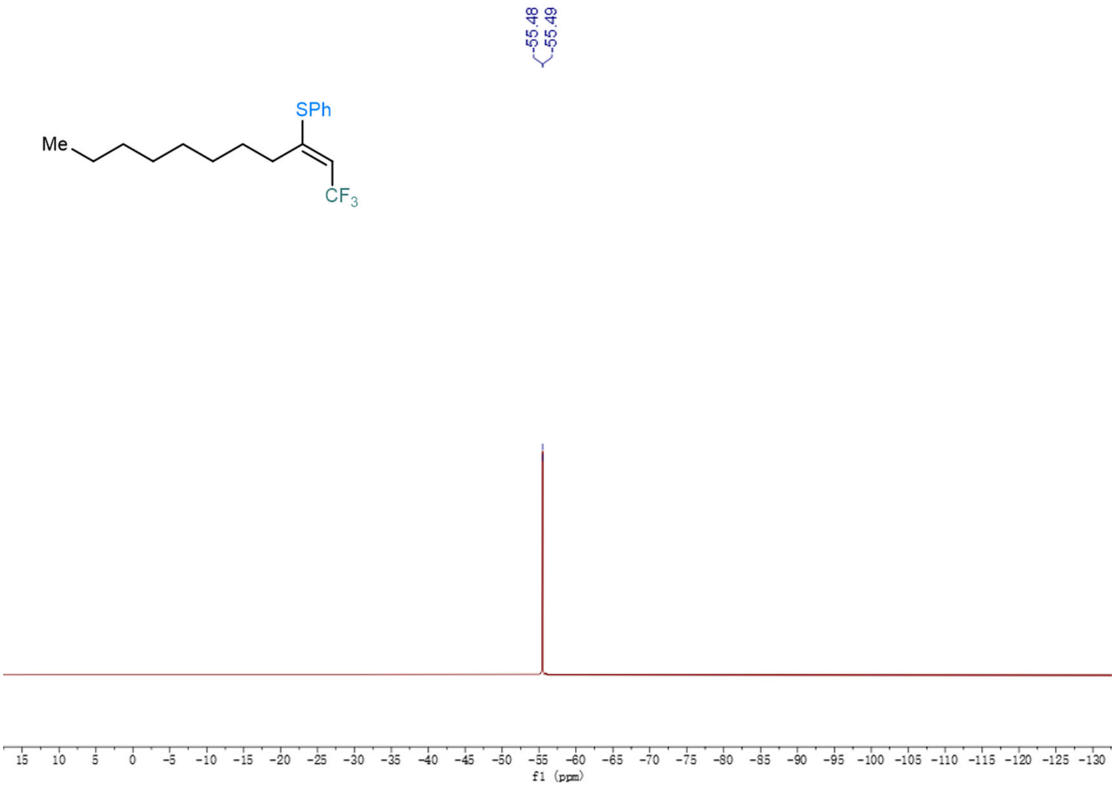

^{19}F NMR spectrum (471 MHz, Chloroform-*d*) of **63**

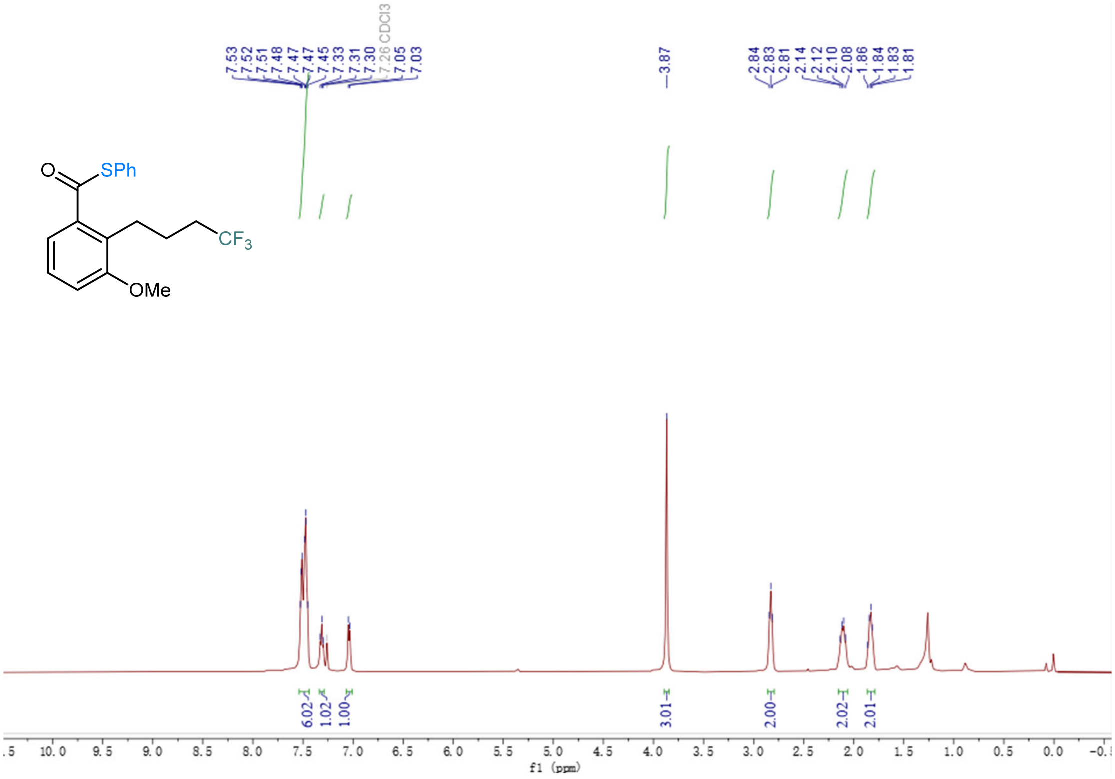

^{1}H NMR spectrum (500 MHz, Chloroform-*d*) of **64**

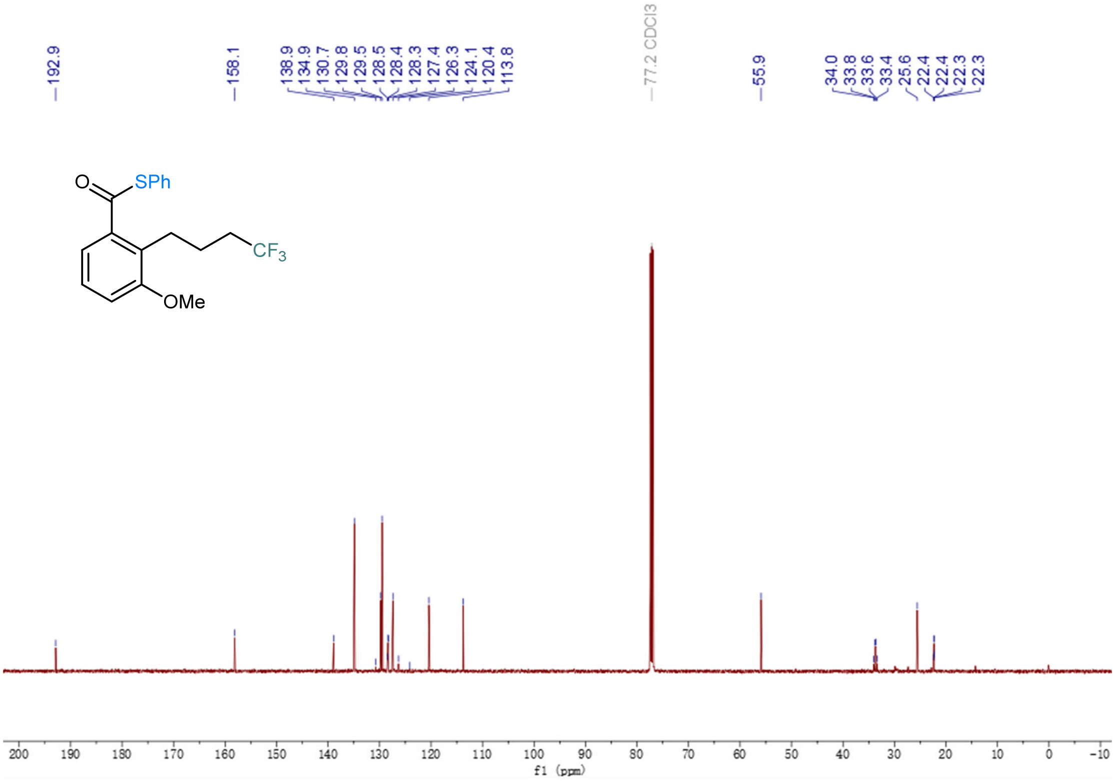

^{13}C NMR spectrum (126 MHz, Chloroform-*d*) of **64**

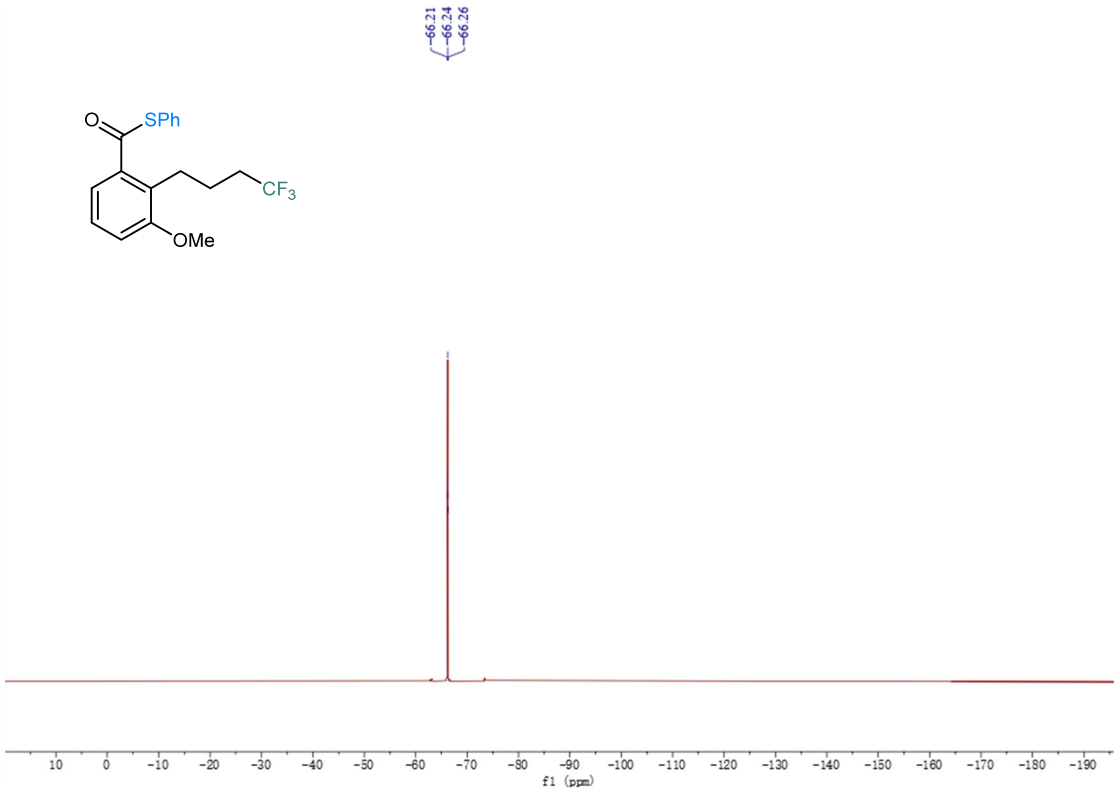

^{19}F NMR spectrum (471 MHz, Chloroform-*d*) of **64**

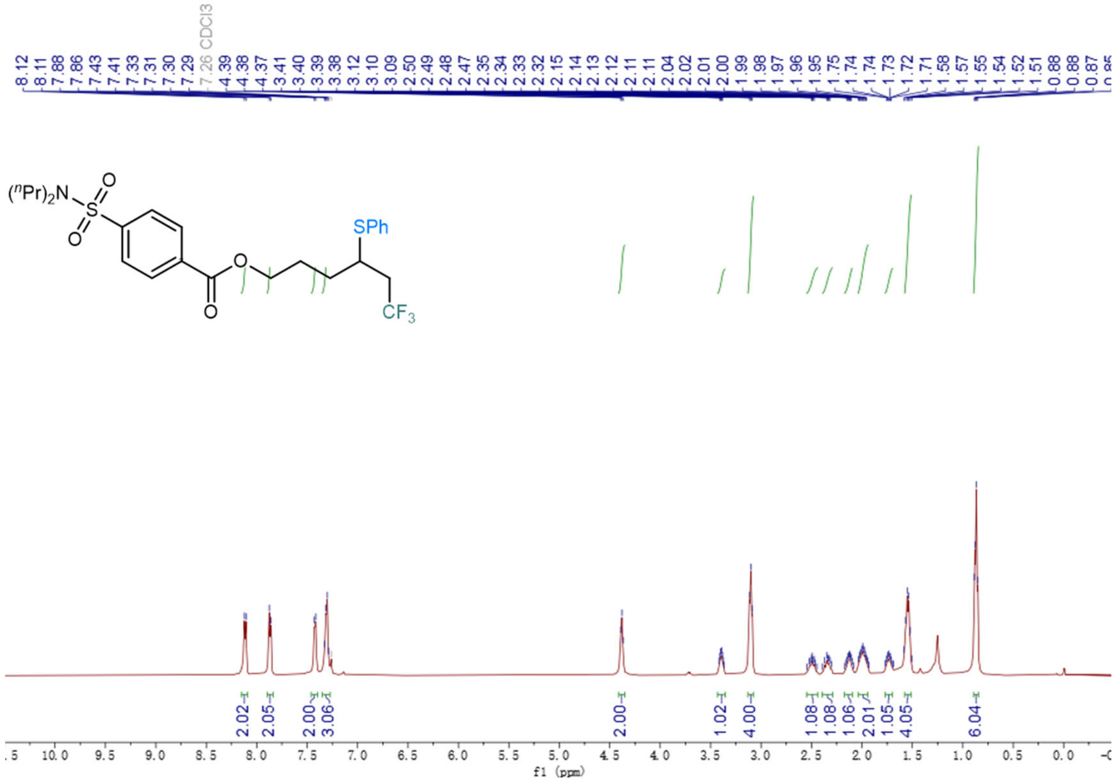

^{1}H NMR spectrum (500 MHz, Chloroform-*d*) of **65**

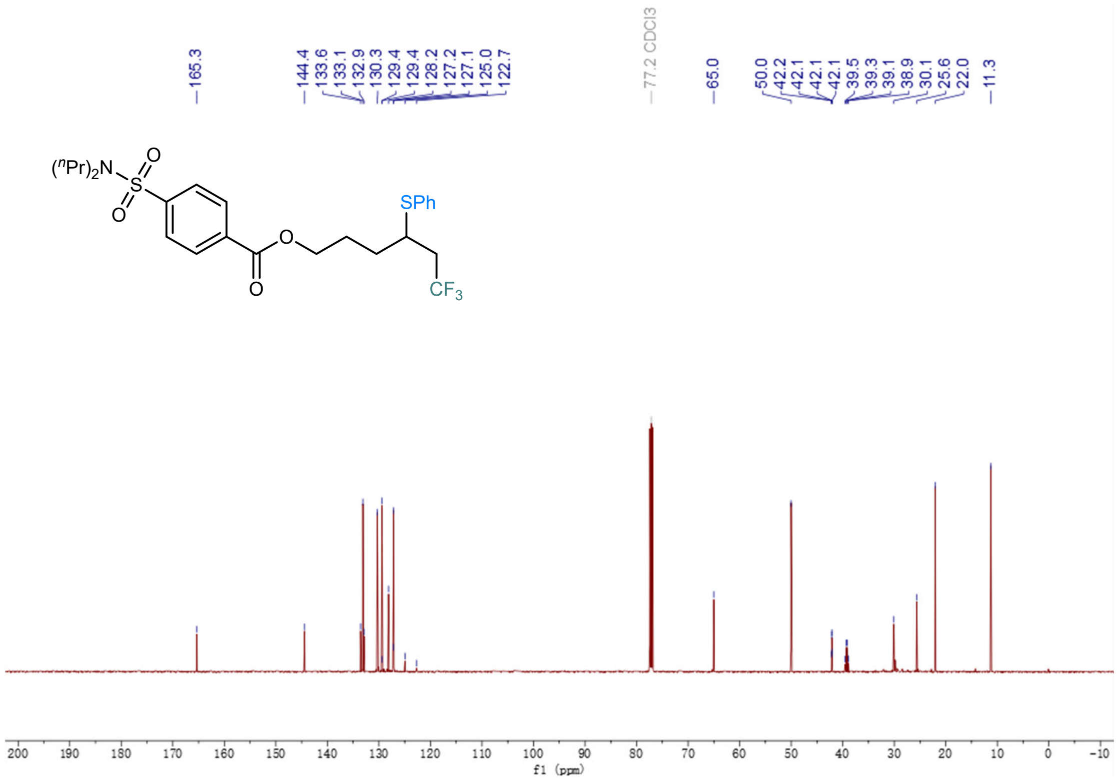

^{13}C NMR spectrum (126 MHz, Chloroform-*d*) of **65**

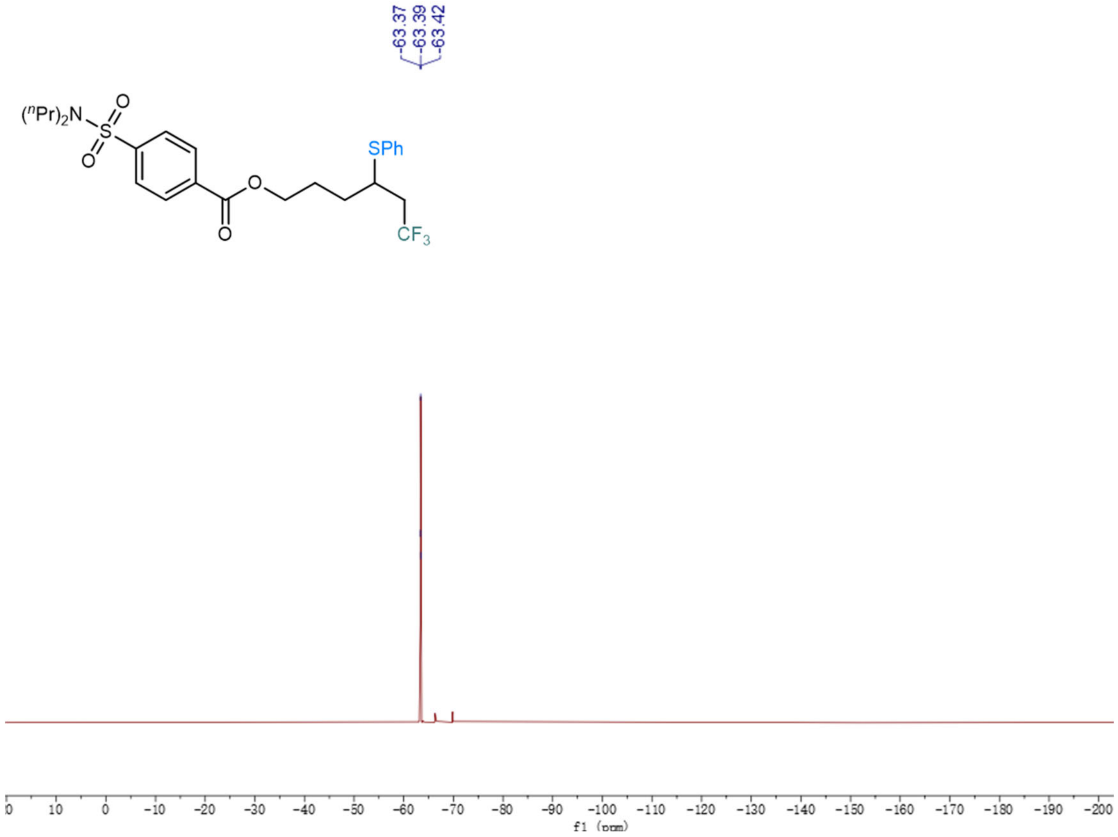

^{19}F NMR spectrum (471 MHz, Chloroform-*d*) of **65**

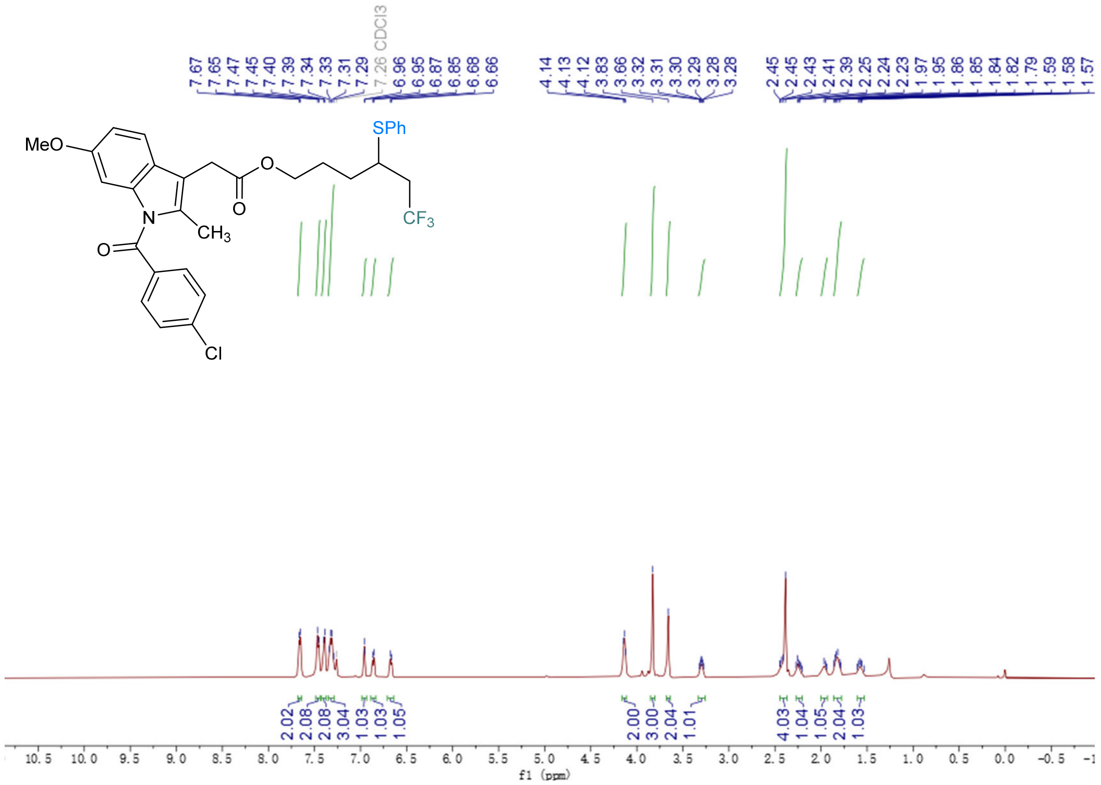

^{1}H NMR spectrum (500 MHz, Chloroform-*d*) of **66**

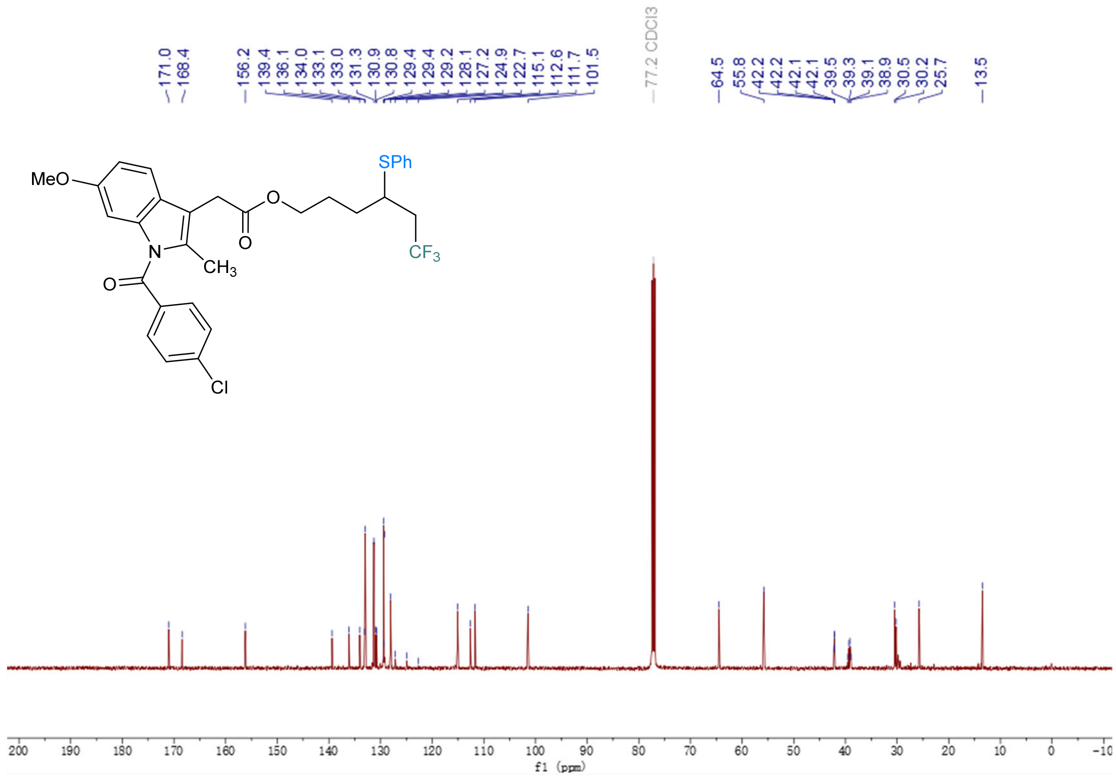

^{13}C NMR spectrum (126 MHz, Chloroform-*d*) of **66**

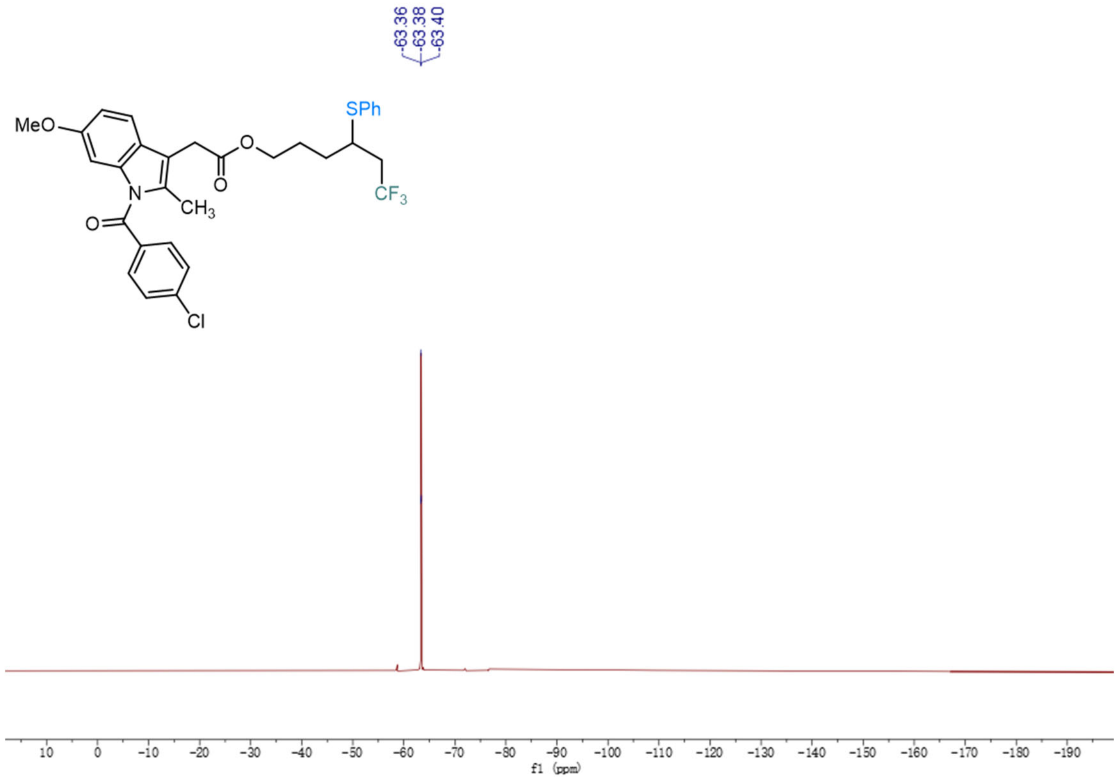

^{19}F NMR spectrum (471 MHz, Chloroform-*d*) of **66**

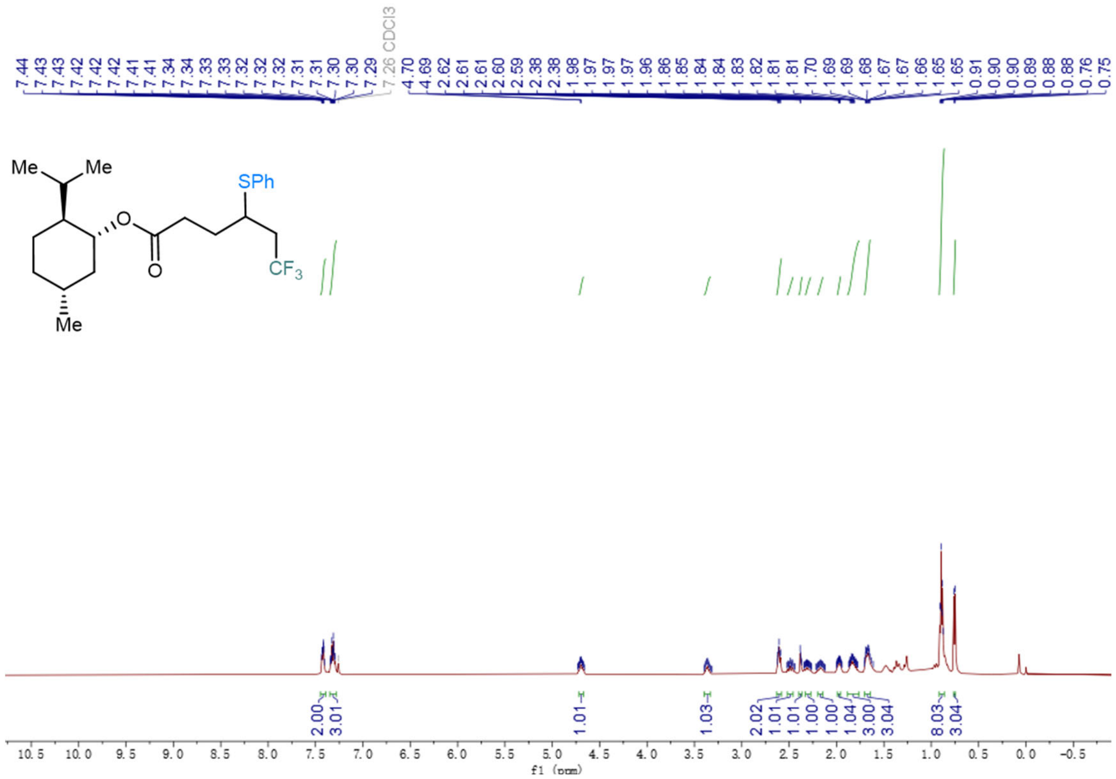

^{1}H NMR spectrum (500 MHz, Chloroform-*d*) of **67**

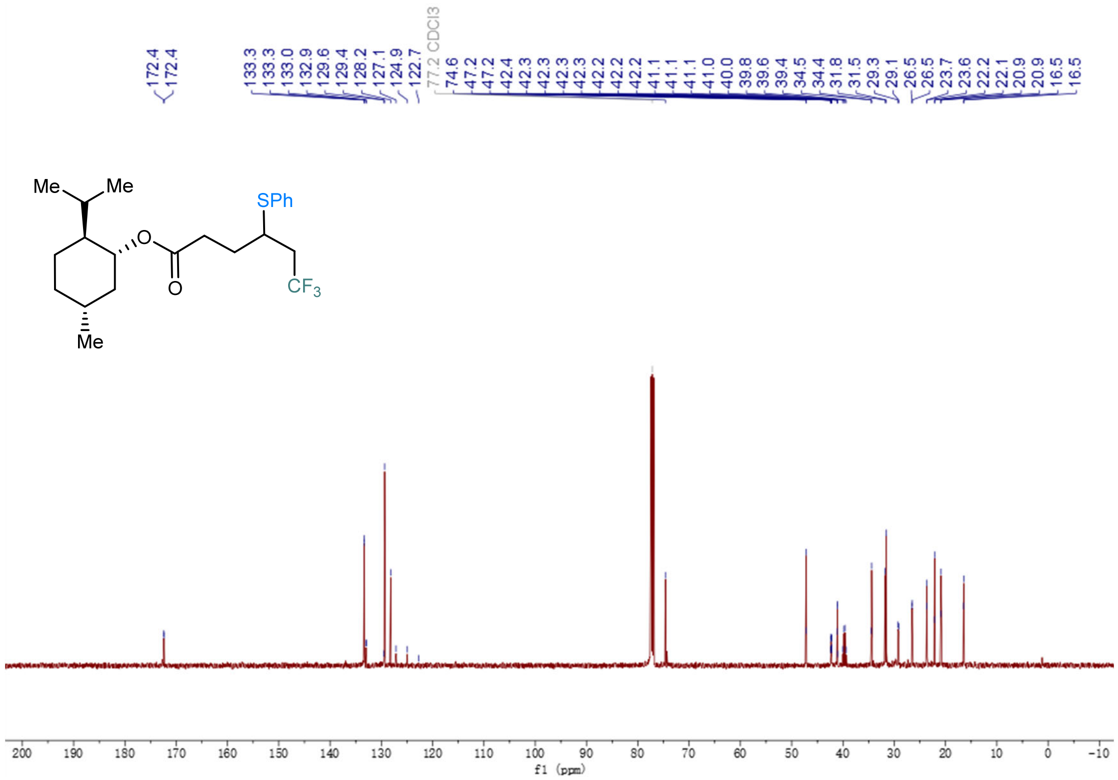

^{13}C NMR spectrum (126 MHz, Chloroform-*d*) of **67**

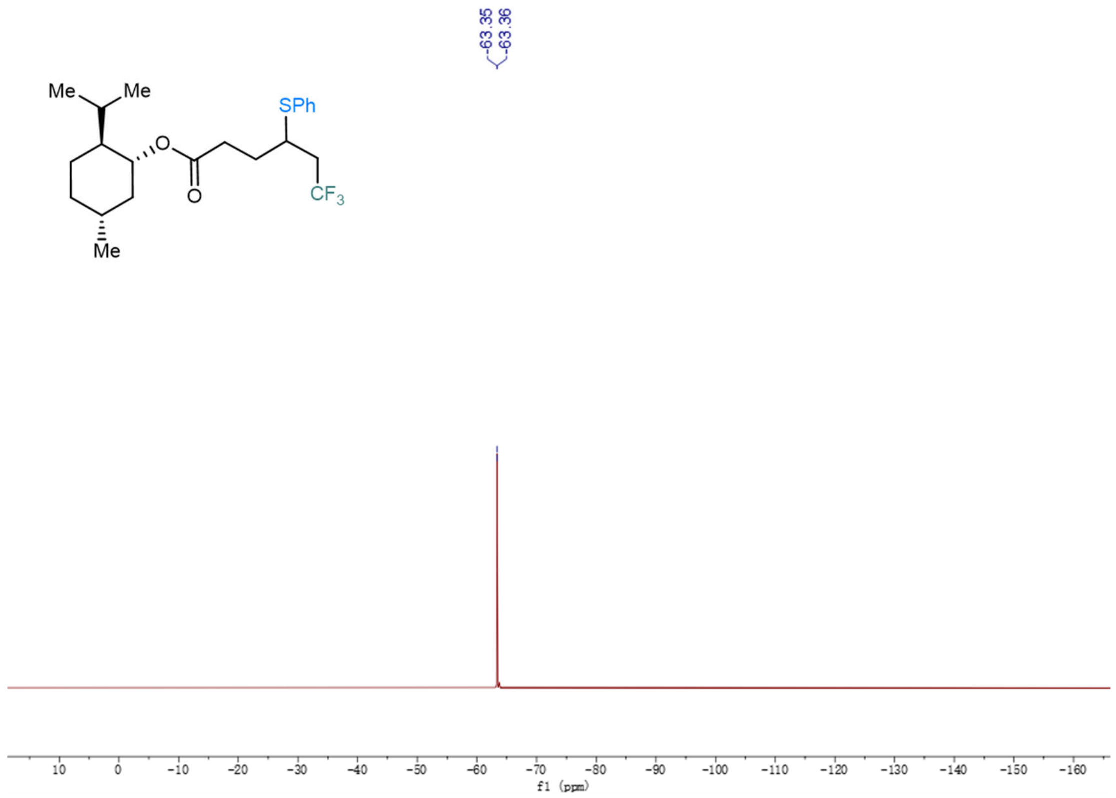

^{19}F NMR spectrum (471 MHz, comp. pulse decoupling, Chloroform-*d*) of **67**

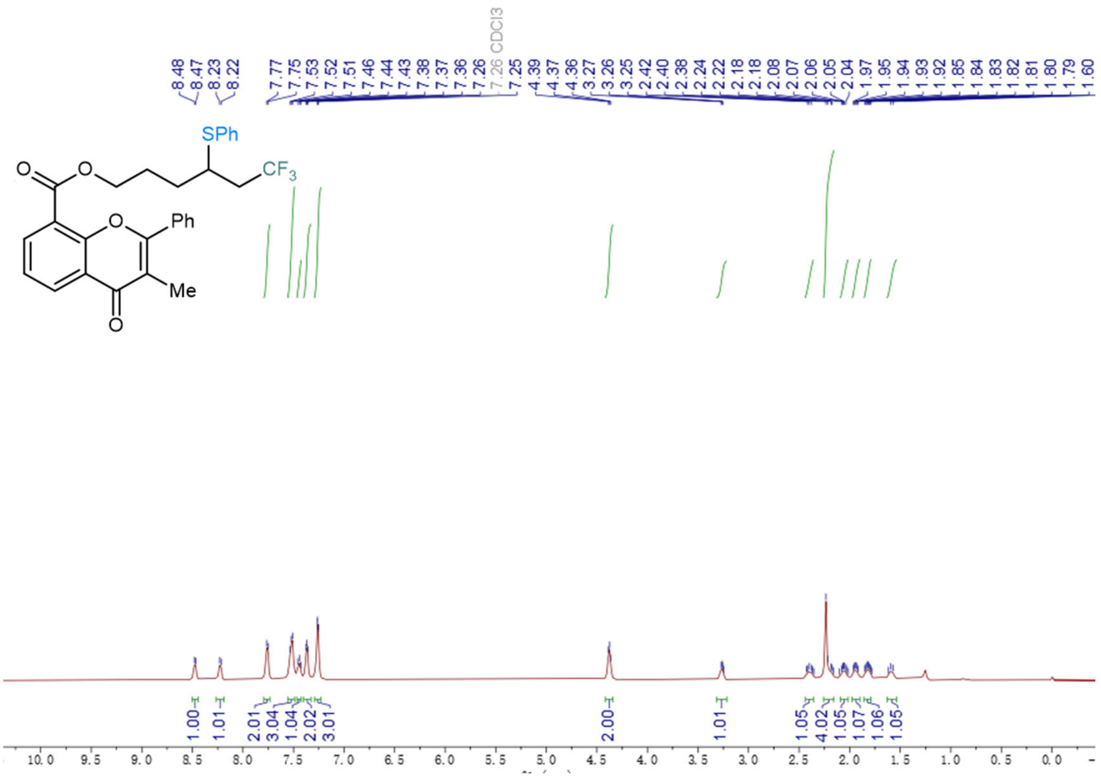

^{1}H NMR spectrum (500 MHz, Chloroform-*d*) of **68**

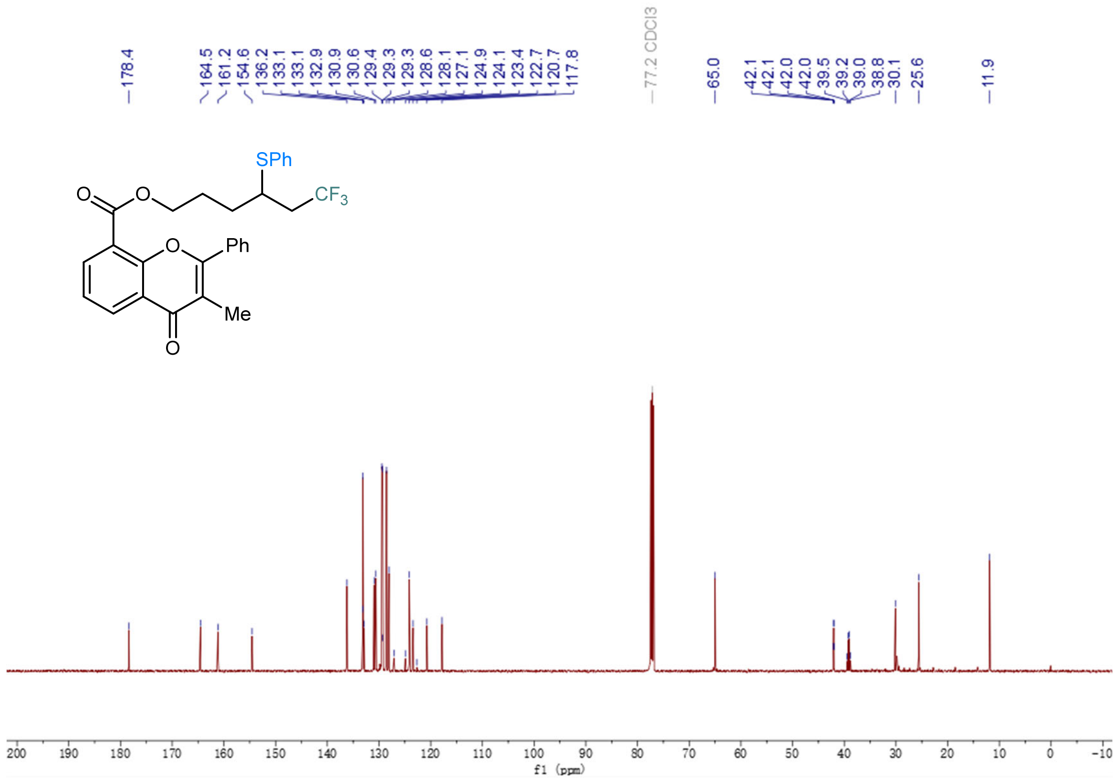

^{13}C NMR spectrum (126 MHz, Chloroform-*d*) of **68**

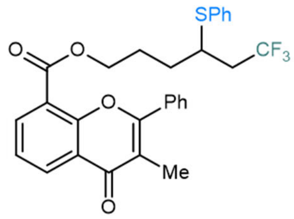

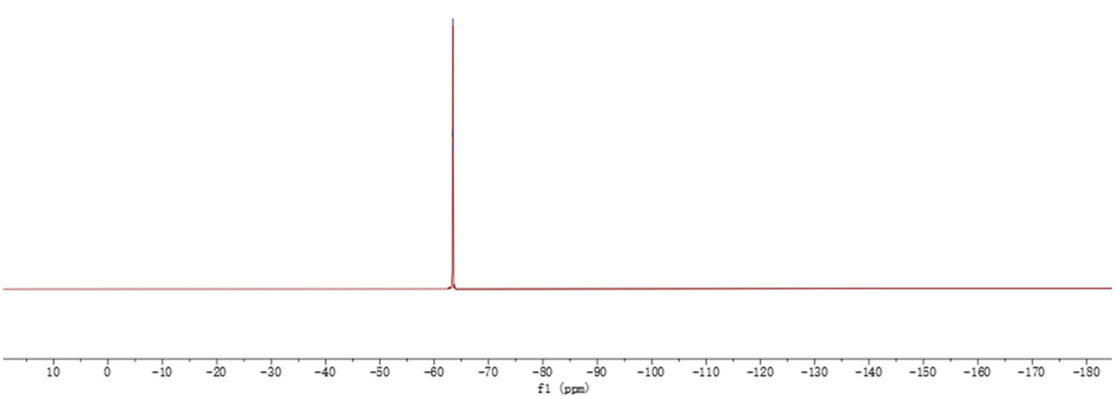

^{19}F NMR spectrum (471 MHz, Chloroform-*d*) of **68**

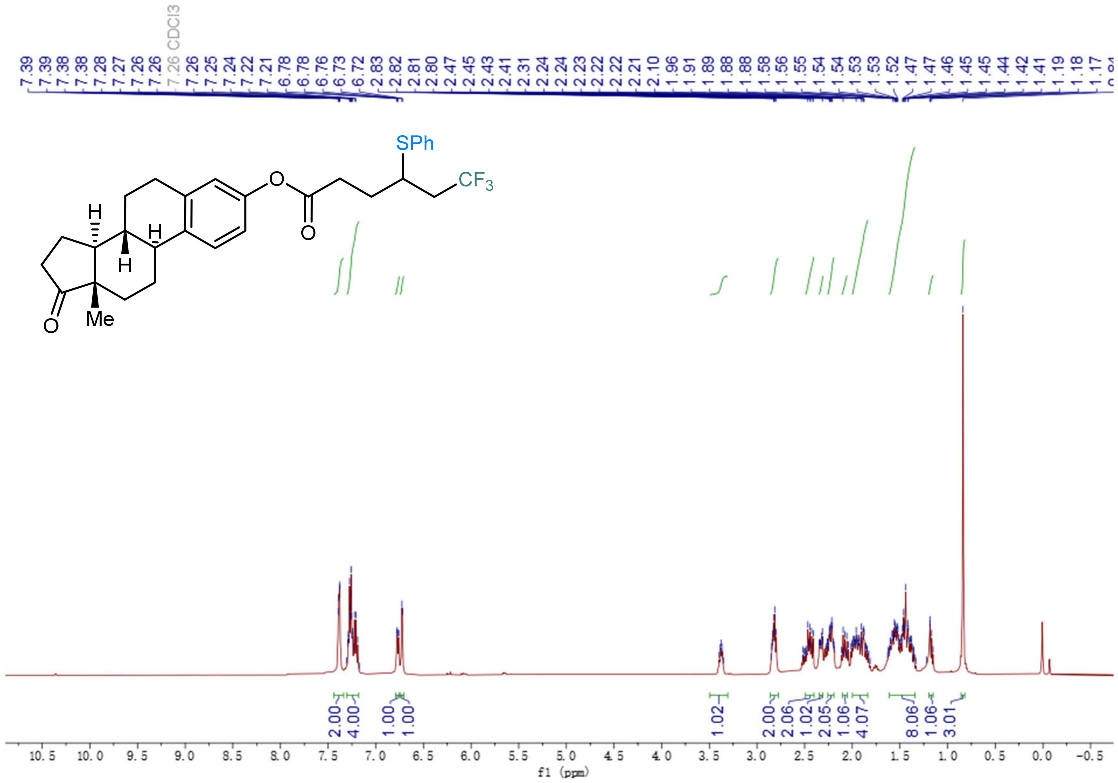

^{1}H NMR spectrum (500 MHz, Chloroform-*d*) of **69**

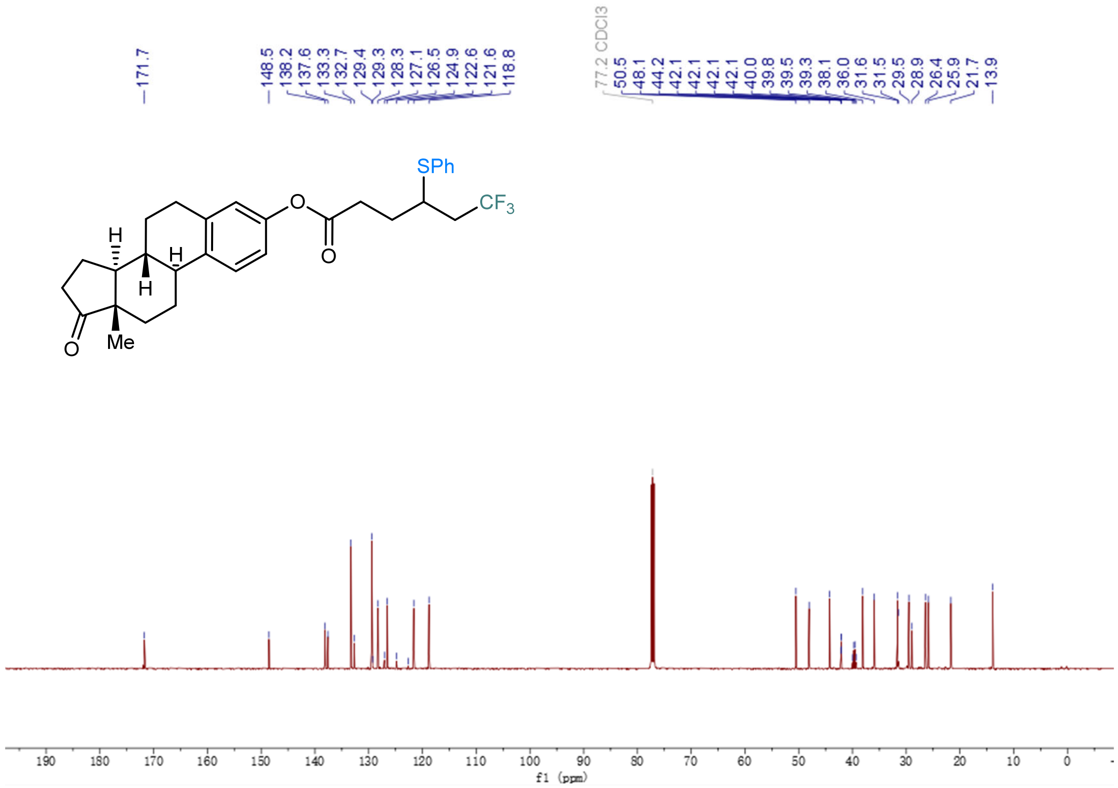

^{13}C NMR spectrum (126 MHz, Chloroform-*d*) of **69**

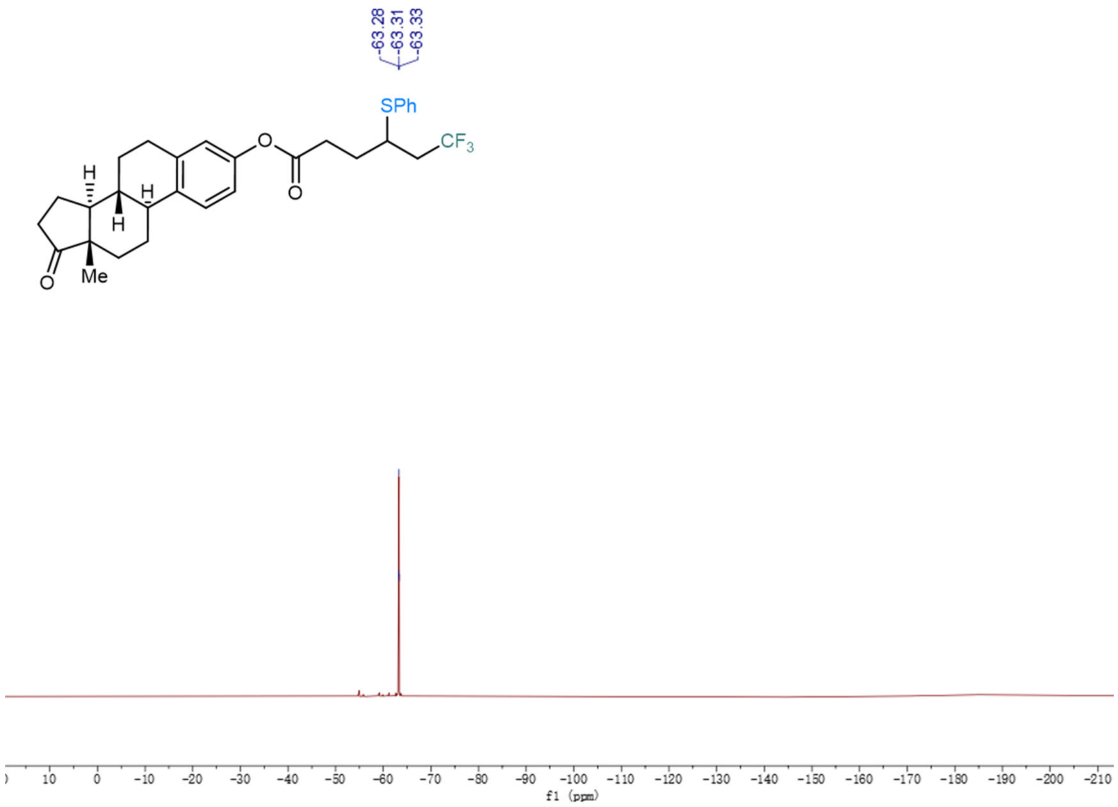

^{19}F NMR spectrum (471 MHz, Chloroform-*d*) of **69**

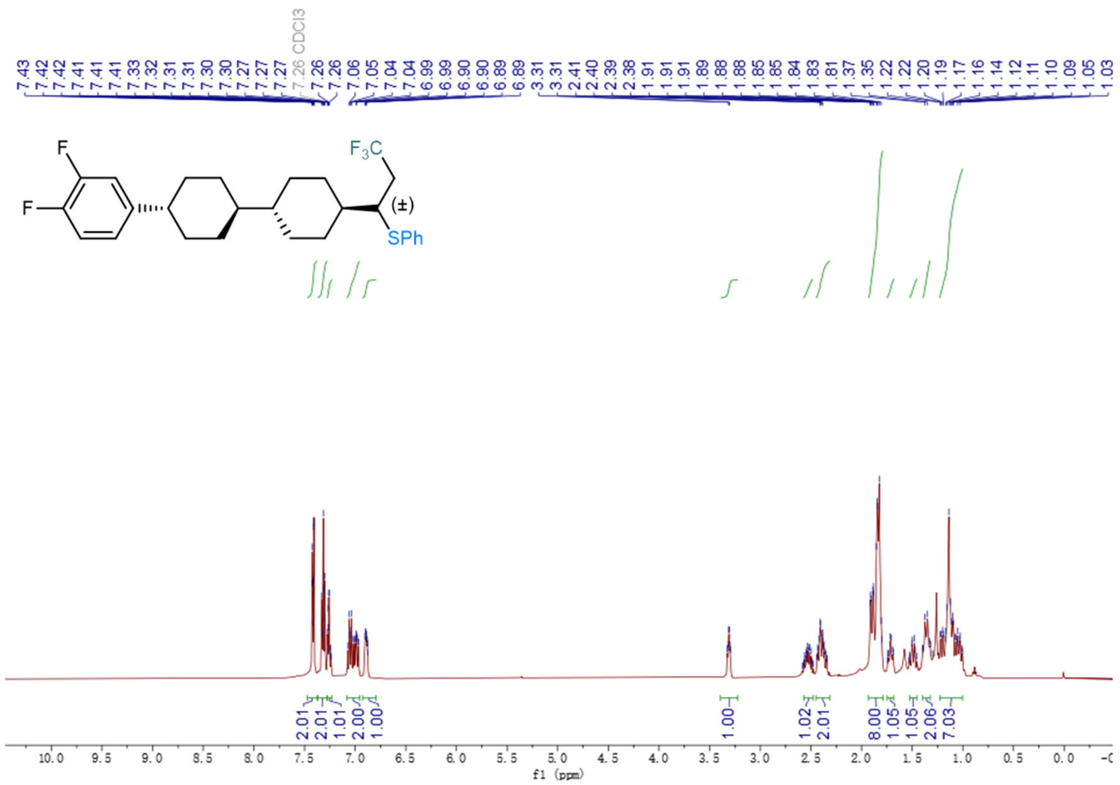

^{1}H NMR spectrum (500 MHz, Chloroform-*d*) of **70**

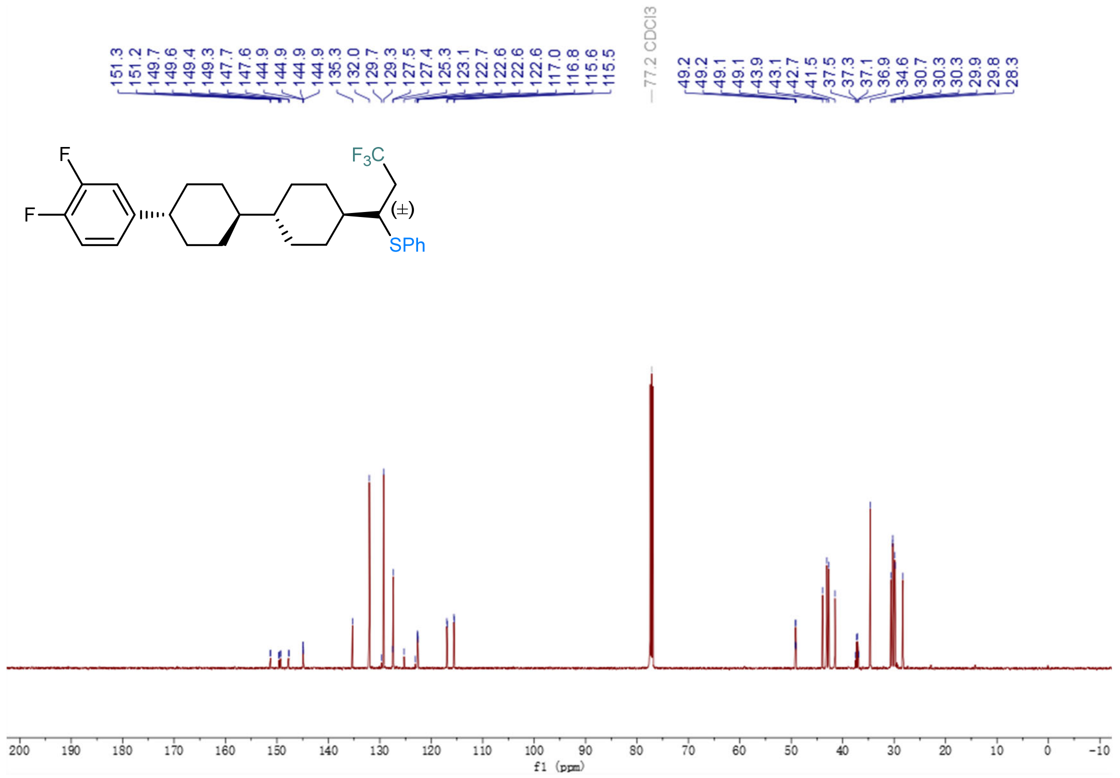

^{13}C NMR spectrum (126 MHz, Chloroform-*d*) of **70**

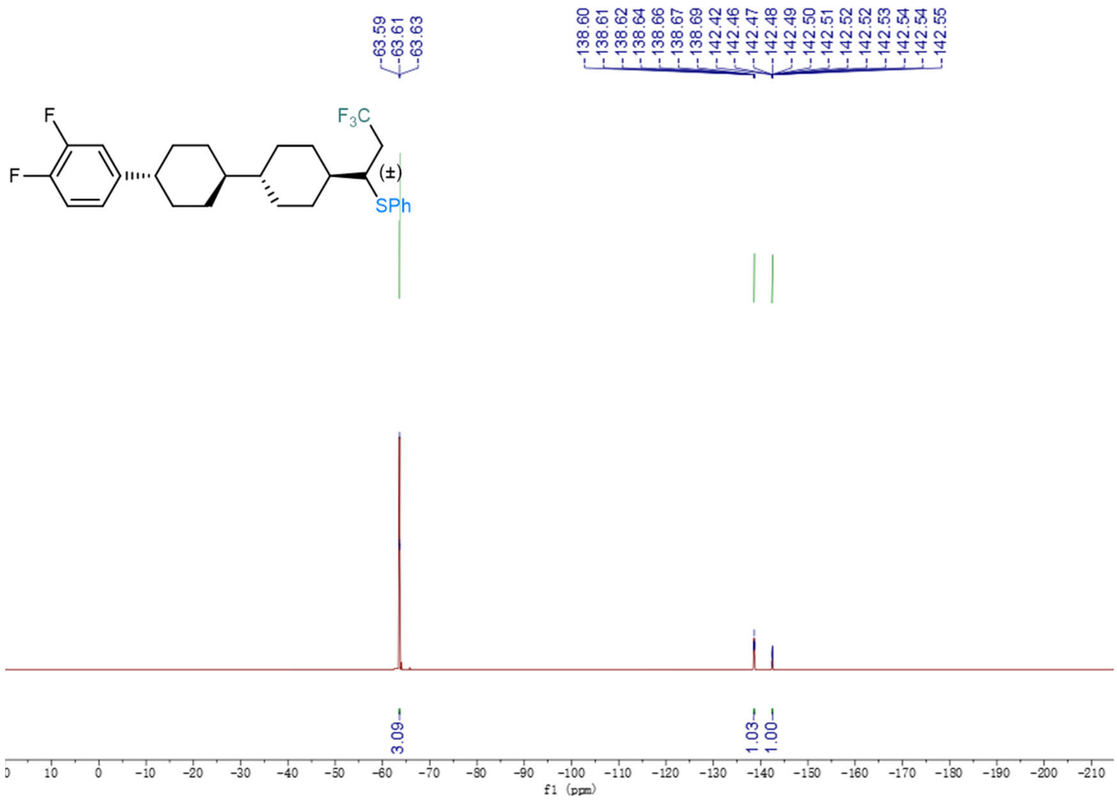

^{19}F NMR spectrum (471 MHz, Chloroform-*d*) of **70**

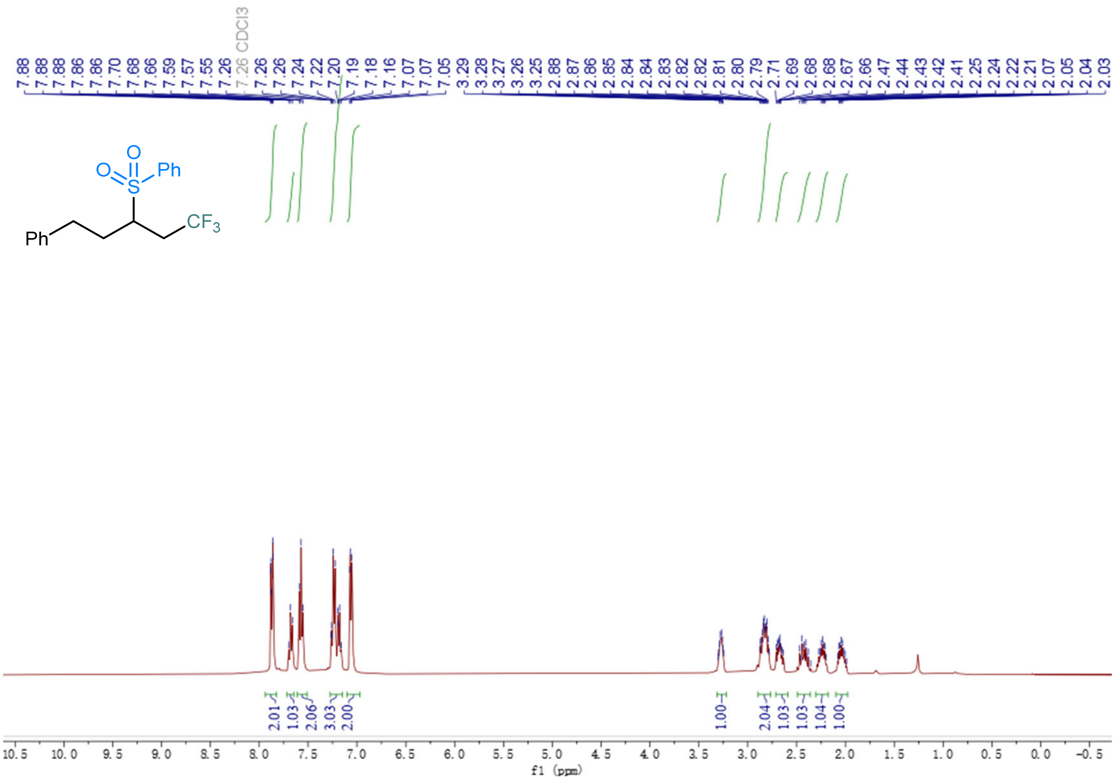

^{1}H NMR spectrum (500 MHz, Chloroform-*d*) of **71**

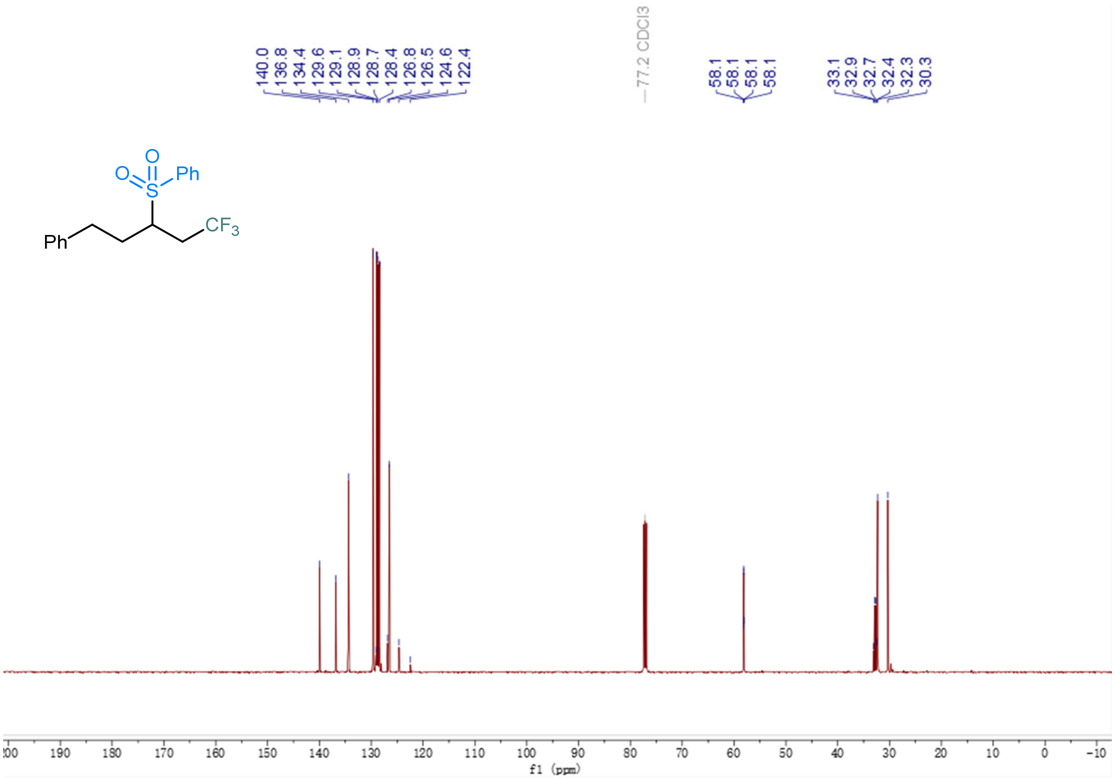

^{13}C NMR spectrum (126 MHz, Chloroform-*d*) of **71**

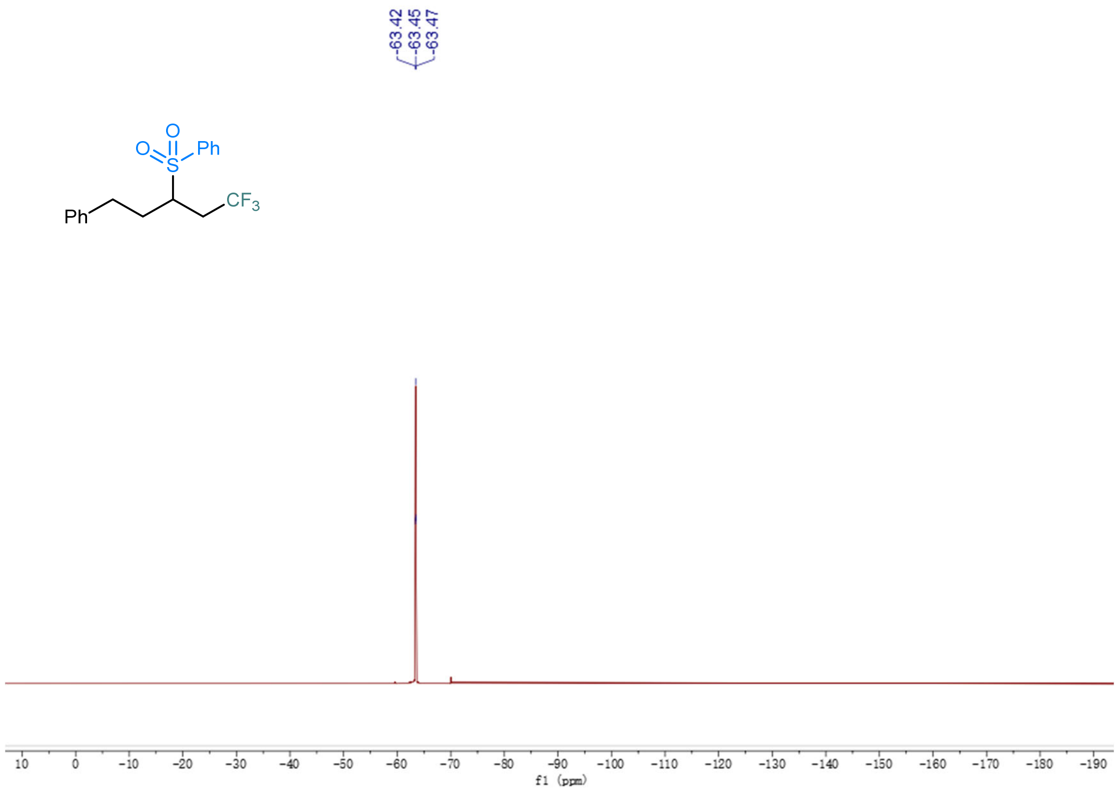

^{19}F NMR spectrum (471 MHz, Chloroform-*d*) of **71**

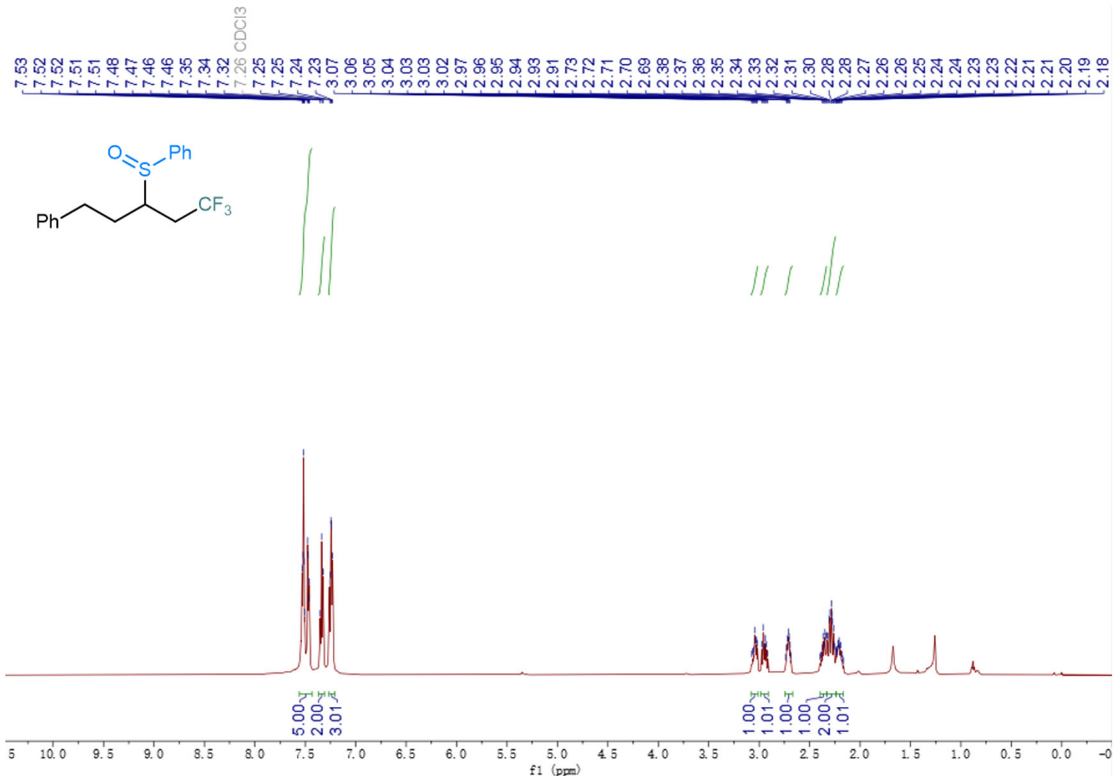

^{1}H NMR spectrum (500 MHz, Chloroform-*d*) of **72**

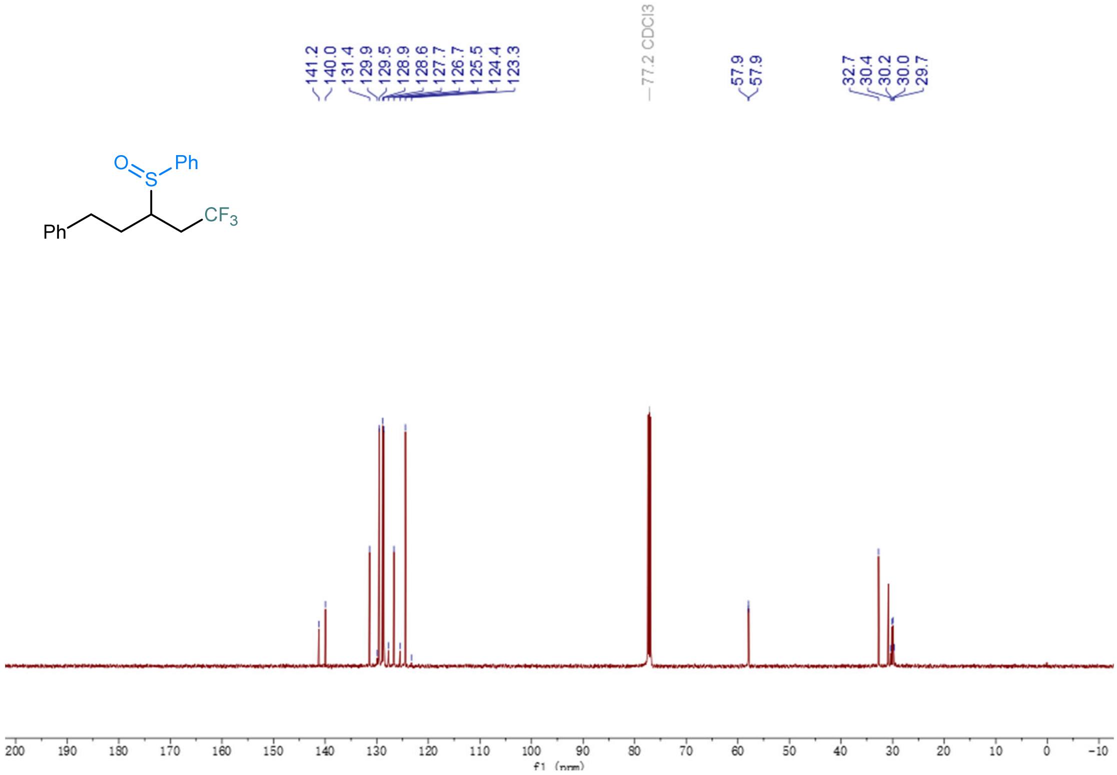

^{13}C NMR spectrum (126 MHz, Chloroform-*d*) of **72**

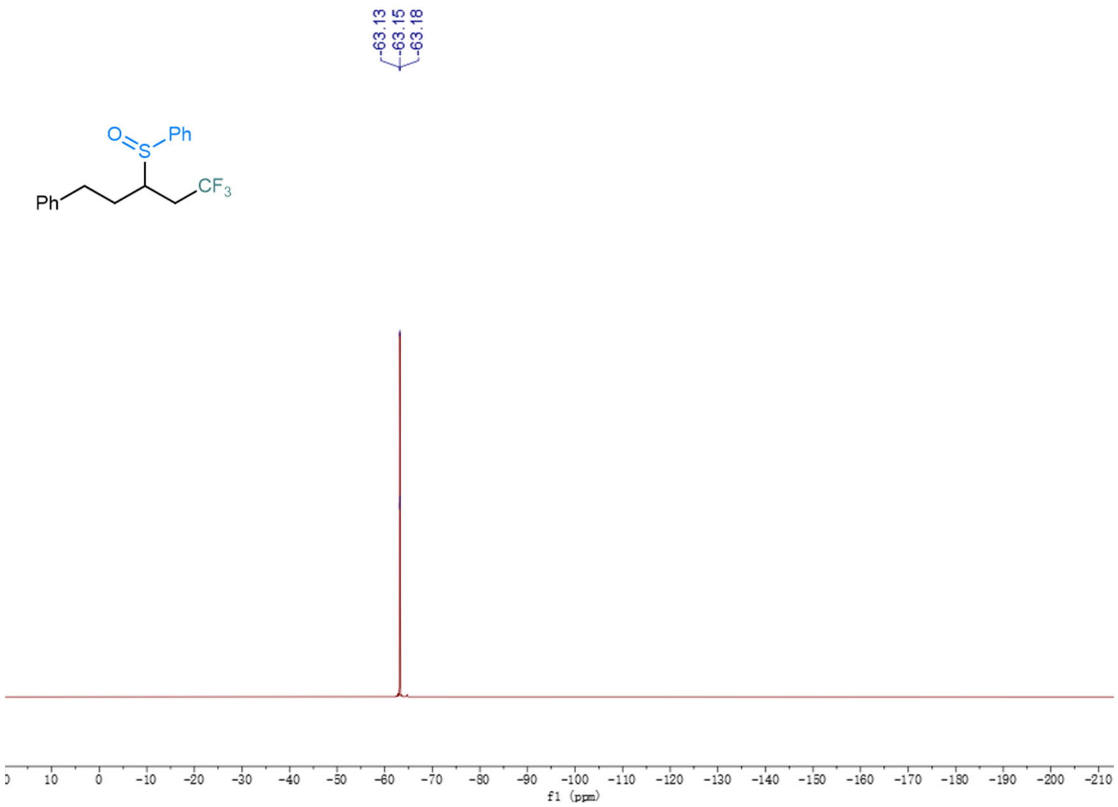

^{19}F NMR spectrum (471 MHz, Chloroform-*d*) of **72**

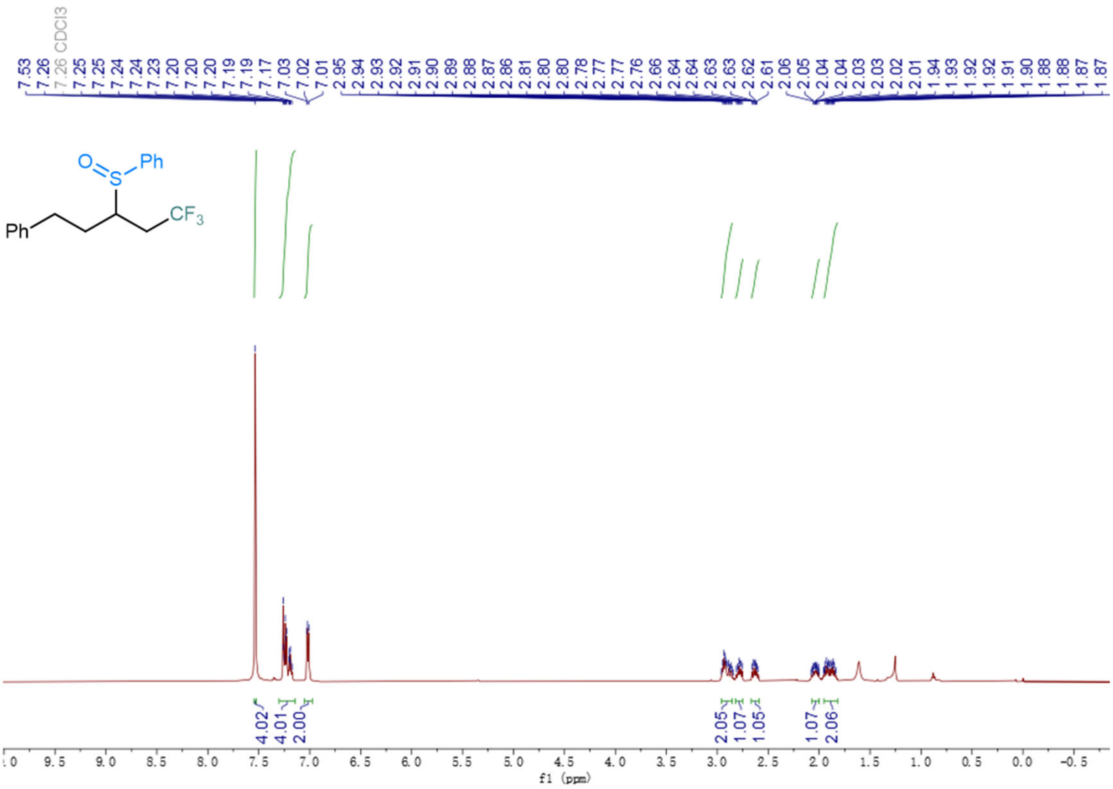

^{1}H NMR spectrum (500 MHz, Chloroform-*d*) of **72'**

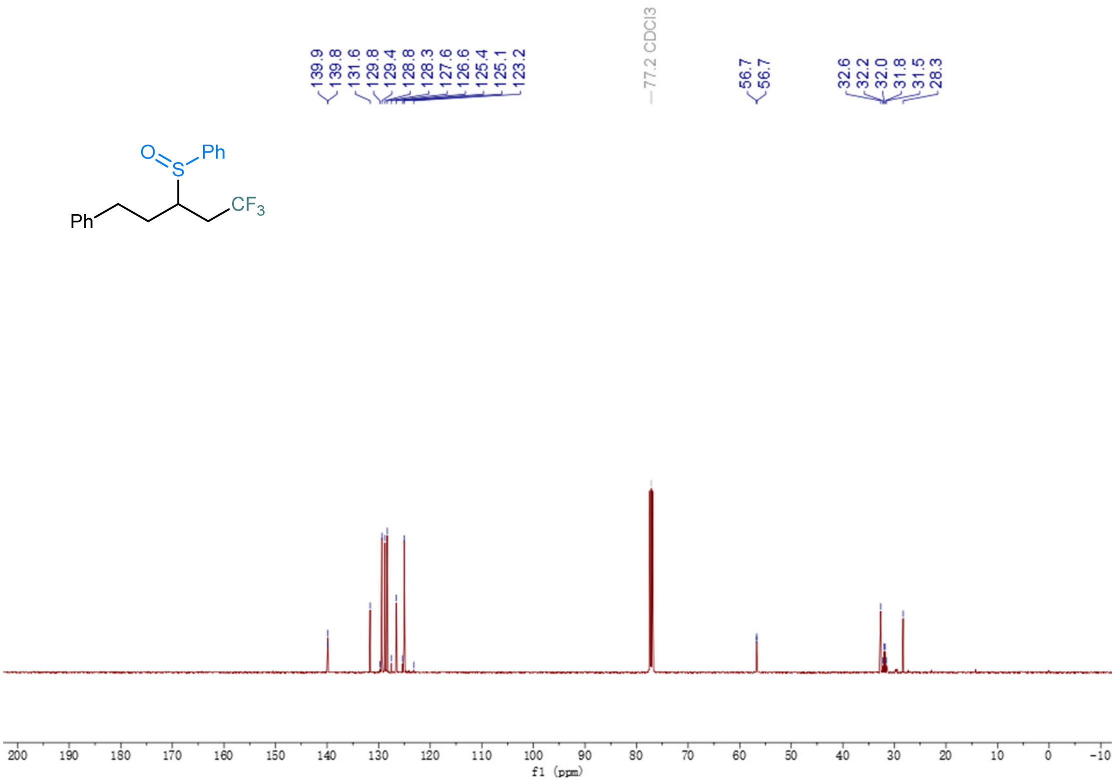

^{13}C NMR spectrum (126 MHz, Chloroform-*d*) of **72'**

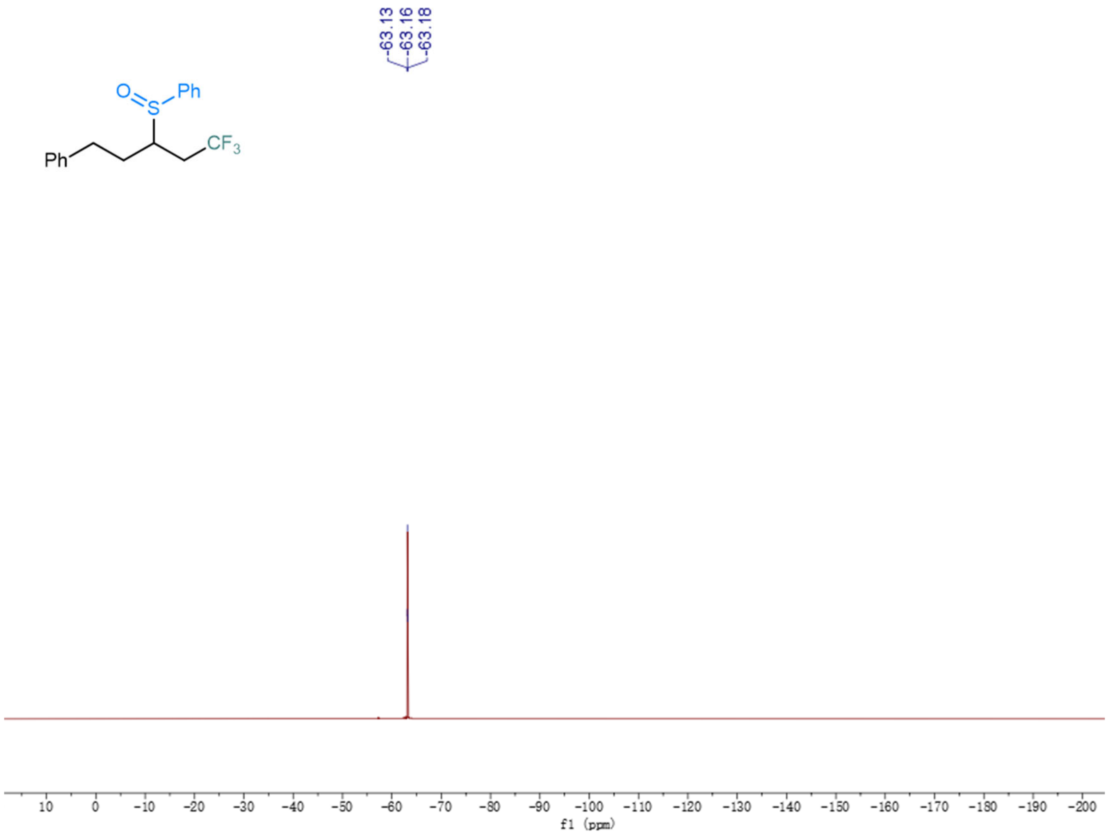

^{19}F NMR spectrum (471 MHz, Chloroform-*d*) of **72'**

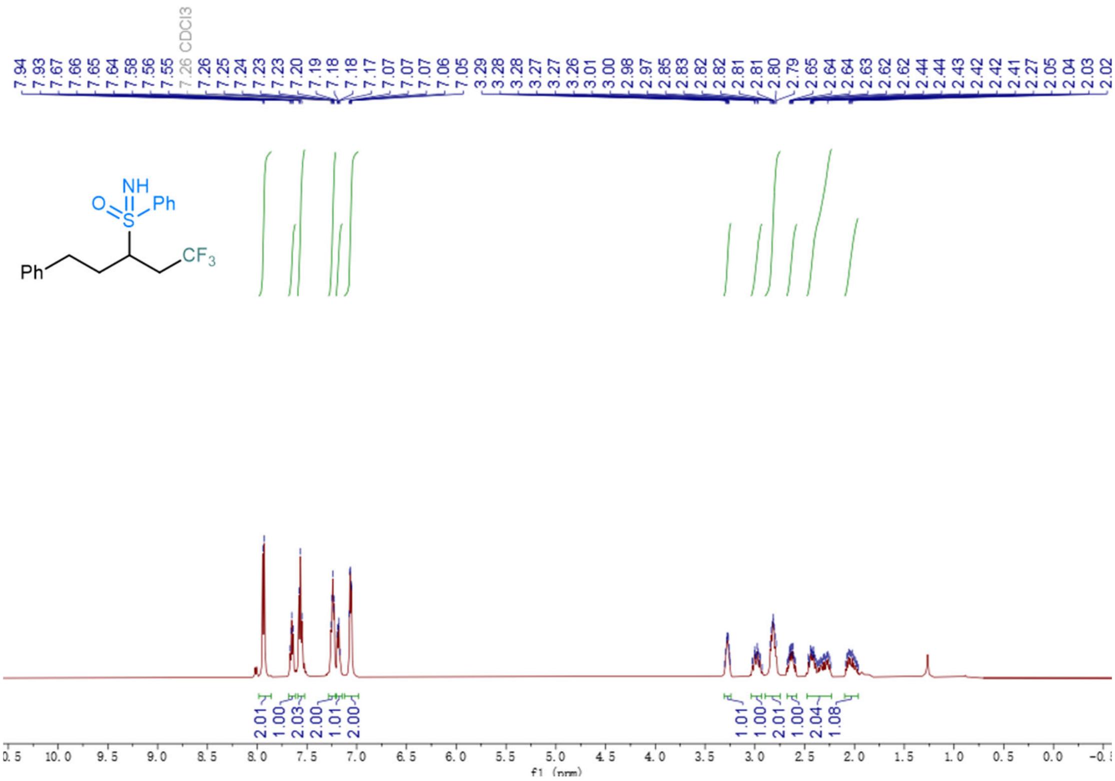

^{1}H NMR spectrum (500 MHz, Chloroform-*d*) of **73**

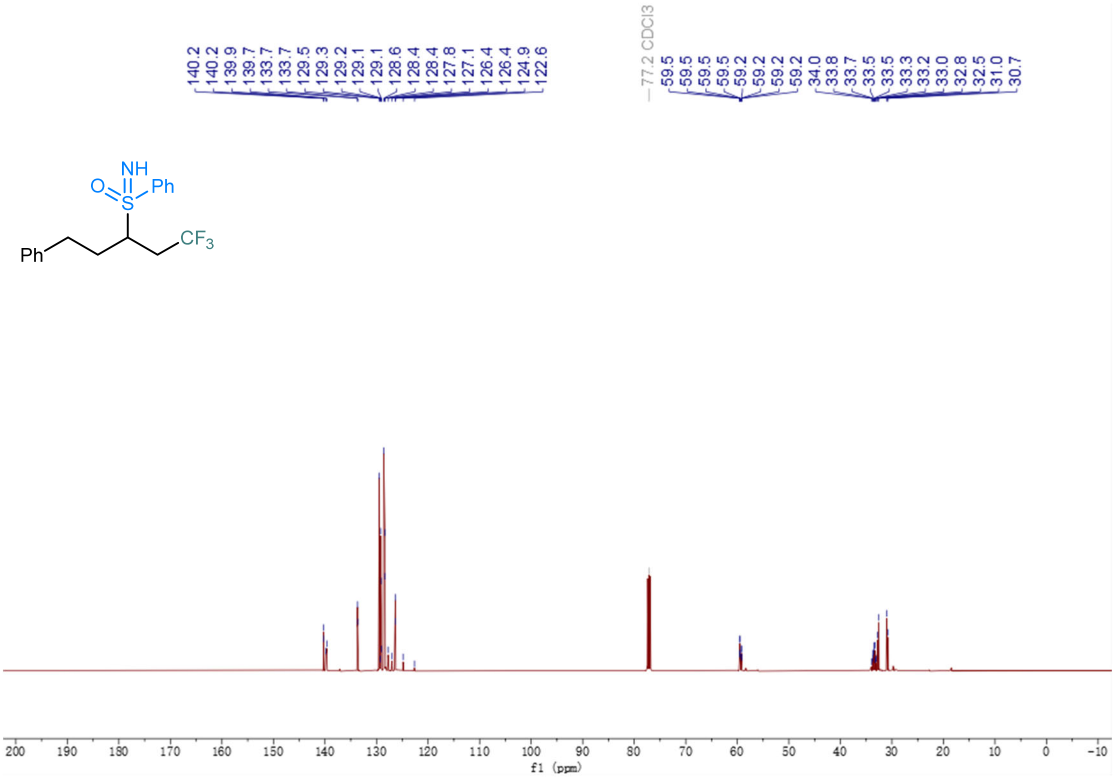

^{13}C NMR spectrum (126 MHz, Chloroform-*d*) of **73**

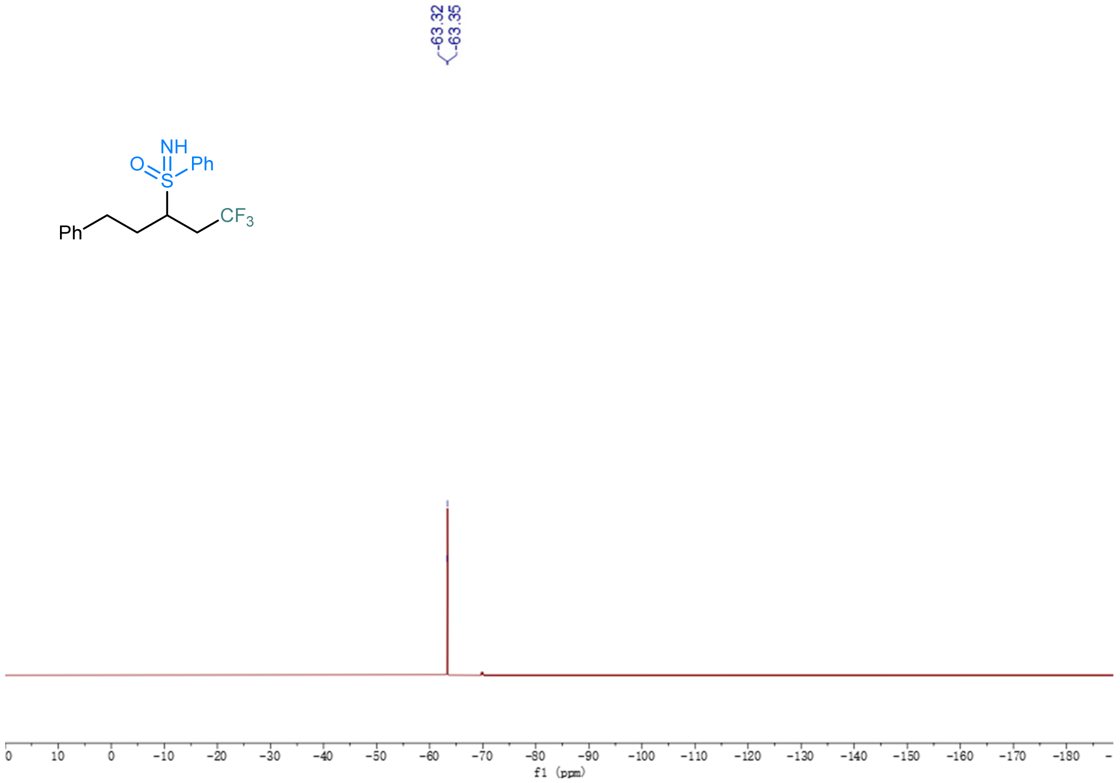

^{19}F NMR spectrum (471 MHz, comp. pulse decoupling, Chloroform-*d*) of **73**

References

(1) Kong, W.; An, H.; Song, Q. Visible-light-induced thiotrifluoromethylation of terminal alkenes with sodium triflinate and benzenesulfonothioates. *Chem. Commun.* **2017**, *53*, 8968–8971.

(2) Huang, Q.; Lou, C.; Lv, L.; Li, Z. Photoinduced fluoroalkylation–peroxidation of alkenes enabled by ligand-to-iron charge transfer mediated decarboxylation. *Chem. Commun.* **2024**, *60*, 12389–12392.

(3) Yang, G.-F.; Liu, Z.; Liu, K.; Wu, X.; Zhu, C.; Li, W.; Xie, J. Modular three-component radical fluoroalkyl-sulfuration of unactivated alkenes. *Chin. J. Catal.* **2025**, *69*, 249–258.

(4) Mampuys, P.; Zhu, Y.; Sergeyev, S.; Ruijter, E.; Orru, R. V.; Van Doorslaer, S.; Maes, B. U. Iodide-catalyzed synthesis of secondary thiocarbamates from isocyanides and thiosulfonates. *Org. Lett.* **2016**, *18*, 2808–2811.

(5) Milzarek, T. M.; Waser, J. Synthesis of Trifluoromethylated Alkenes: Hypervalent Iodine Meets High-Valent Copper. *Angew. Chem. Int. Ed.* **2023**, *62*, e202306128.

(6) Natho, P.; Colella, M.; Luisi, R. A Decade of NH-Transfer via Iodonitrene: A Journey of NH-Transfer from Sulfur and Nitrogen to Alkenes. *Synlett* **2026**, DOI: 10.1055/a-2845-8119.
